# Combining Photocatalytic Oxidation of β‐Chlorohydrins with Carbonyl Bioreduction in a Deracemization Approach

**DOI:** 10.1002/cssc.202500683

**Published:** 2025-06-29

**Authors:** Sara Filgueira, Laura Rodríguez‐Fernández, Iván Lavandera, Vicente Gotor‐Fernández

**Affiliations:** ^1^ Organic and Inorganic Chemistry Department Instituto Universitario de Química Organometálica “Enrique Moles” Universidad de Oviedo Avenida Julián Clavería, 8 33006 Oviedo Spain

**Keywords:** biocatalysis, deracemizations, halohydrins, oxidations, photocatalysis

## Abstract

Enantiopure β‐chlorohydrins are valuable intermediates in organic synthesis, acting as chiral building blocks for obtaining biologically active compounds. This work presents a one‐pot two‐step linear sequence for the efficient deracemization of β‐chlorohydrins by combining a photocatalytic alcohol oxidation with a stereoselective carbonyl bioreduction using alcohol dehydrogenases. Despite the thermodynamic challenges of the oxidation step for this family of substrates, three efficient photochemical conditions under white light irradiation are found using catalytic 2,4,6‐triphenylpyrylium tetrafluoroborate or 2,3‐dichloro‐5,6‐dicyano‐1,4‐benzoquinone in this case in stoichiometric or by in situ regeneration of the catalyst. In addition, its sunlight‐driven applicability and its implementation in flow chemistry are demonstrated. After testing the scope of these methodologies, 2‐chloro‐1‐arylethanols are found as suitable substrates. For those β‐halohydrins oxidized with complete conversion, the deracemization strategy is successfully achieved, yielding 12 enantiopure (*R*)‐ and (*S*)‐β‐halohydrins. Furthermore, 2‐chloro‐1‐phenylethanol enantiomers are subjected to an additional chemical cyclization under basic conditions, providing both (*R*)‐ and (*S*)‐styrene oxide in a three‐step one‐pot process and without loss of the optical activity.

## Graphical abstract

The oxidation of β‐chlorohydrins is thermodynamically unfavourable. To solve this, different photocatalytic systems are explored under white light irradiation. Best results are achieved using 2,3‐dichloro‐5,6‐dicyano‐1,4‐benzoquinone in stochiometric or catalytic loadings including *tert*‐butyl nitrite/O_2_ as regeneration system. Thus, the synthesis of several α‐haloketones is accomplished, which are employed as intermediates in multistep chemoenzymatic cascades.

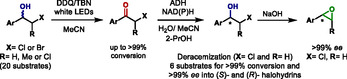

## 1 Introduction

Enantiopure β‐chlorohydrins are valuable intermediates in organic synthesis as chiral building blocks for obtaining biologically active compounds.^[^ ^1^, ^2^ ^]^ In recent years, enzymatic methods have emerged as valuable tools for the synthesis of this family of compounds with high (stereo)selectivity.^[^ ^3^ ^]^ The traditional approach involves the enzymatic kinetic resolution of racemic alcohol or acetate forms using lipases that has allowed the asymmetric preparation of valuable drug precursors.^[^ ^4^, ^5^, ^6^, ^7^, ^8^, ^9^ ^]^ However, this method has a major drawback, its inherent 50% maximum yield, since the unwanted enantiomer is discarded, thus reducing the overall efficiency of the process. To overcome this limitation, lipases have been implemented in chemoenzymatic dynamic kinetic resolutions.^[^ ^10^, ^11^, ^12^ ^]^ Alternatively, the asymmetric bioreduction of prochiral α‐haloketones using alcohol dehydrogenases (ADHs) overcomes this synthetic limitation.^[^ ^3^, ^6^, ^13^, ^14^, ^15^, ^16^, ^17^ ^]^ However, the synthesis of α‐haloketones can be challenging, for example, via halogenation of ketones^[^ ^18^ ^]^ or oxyhalogenation of alkenes and alkynes.^[^ ^19^, ^20^ ^]^

**Scheme 1:**
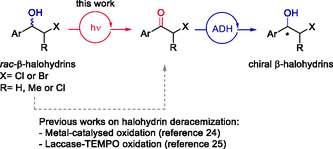
Oxidation of β‐halohydrins using different methodologies followed by carbonyl bioreduction for the design of chemoenzymatic halohydrin deracemizations.

The enzymatic reduction of α‐haloketones, in combination with a previous oxidation step, can be applied to deracemize β‐chlorohydrins. However, the high stability of β‐halohydrins, caused by several factors, complicates their oxidation. On the one hand, the presence of an intramolecular interaction between the alcohol group and the chlorine atom.^[^ ^21^ ^]^ On the other hand, the chlorine atom has a strong inductive electron‐withdrawing effect, creating an induced partial positive charge on the carbon bearing the hydroxyl group, reducing the nucleophilicity and reactivity of the hydroxyl group.^[^ ^22^ ^]^ Our research group correlated the reactivity of this family of compounds based on their intrinsic chemical structure,^[^ ^23^ ^]^ observing a great influence of their infrared frequencies on their reactivity that emphasizes a thermodynamic control over the kinetic component. As a result, the deracemization of β‐halohydrins is scarcely reported, with a few examples where the first oxidative step has been achieved by employing either an iridium catalyst^[^ ^24^ ^]^ or a system formed by a laccase and the 2,2,6,6‐tetramethylpiperidine‐1‐oxyl (**Scheme** **1**).^[^ ^25^ ^]^

In this context, methodologies employing metal‐free catalysts, green oxidants, and mild reaction conditions are highly appealing for large‐scale applications. Thus, photobiocatalysis has emerged as an elegant tool for achieving the synthesis of optically active compounds,^[^ ^26^, ^27^, ^28^, ^29^, ^30^, ^31^, ^32^ ^]^ including, among others, the disclosure of redox strategies. Unfortunately, current reports on the photobiocatalytic deracemization of *sec*‐alcohols lack the capability to efficiently oxidize β‐chlorohydrins due to their mentioned stability. In this study, we propose a one‐pot two‐step photobiocatalytic methodology for the efficient deracemization of β‐chlorohydrins that consists of an initial photo‐oxidation to their corresponding α‐haloketones using an organophotocatalyst under white light (Scheme 1). Subsequently, in an one‐pot fashion, the generated α‐haloketones undergo asymmetric bioreduction catalyzed by an ADH, allowing the synthesis of the desired enantiopure β‐chlorohydrins. Our approach could represent a significant advance in the synthesis of β‐chlorohydrins, introducing a greener and efficient alternative for the challenging oxidation step.

## 2 Results and Discussion

### 2.1 Photo‐Oxidation of β‐Chlorohydrins

First, we focused on studying the initial photo‐oxidation of racemic β‐chlorohydrins considering that for deracemization purposes it is crucial to proceed with full conversion in this oxidative step. Otherwise, the presence of nonreacted racemic alcohol may reduce the enantiomeric excess (*ee*) of the resulting optically active product in the overall strategy, even if the subsequent asymmetric bioreduction occurred with perfect stereoselection.^[^ ^33^, ^34^, ^35^, ^36^, ^37^, ^38^ ^]^ Thus, the reported difficulty to oxidize these substrates combined with this requirement remarks on the particular challenge of this contribution. Up to now, the photo‐oxidation of β‐chlorohydrins has only been briefly reported for 2‐chloro‐1‐phenylethanol (**1a**) using 2‐bromoanthraquinone (40% yield)^[^ ^39^ ^]^ and 2‐bromo‐6‐nitropyridine (82% yield).^[^ ^40^ ^]^ However, these results are not sufficient for our strategy to meet the demanding criteria.

Taking all of this into account, a screening of photosensitizers was performed using 2‐chloro‐1‐phenylethanol (**1a**) as a model substrate (Table S1, Supporting Information) to display a comprehensive study, while in **Table** **1** the most remarkable results have been highlighted. Initial tests conducted with 9‐fluorenone (entry 1) and sodium anthraquinone 2‐sulfonate (SAS, entry 2), which are reported to be successful in the photobiocatalytic deracemization of 1‐arylethanols,^[^ ^41^, ^42^ ^]^ gave ketone **2a** in low to moderate conversions (3–65%) leading us to explore other photocatalytic candidates. Other single electron transfer (SET) photosensitizers with higher excited‐state reduction potentials (*E* ^T1^_red_) were tested as stronger oxidants: 2,4,6‐triphenylpyrylium tetrafluoroborate (TPPT, entry 3) and 2,3‐dichloro‐5,6‐dicyano‐1,4‐benzoquinone (DDQ, entry 4).^[^ ^43^, ^44^, ^45^, ^46^ ^]^ Satisfyingly, both photosensitizers catalyzed the formation of ketone **2a** with complete conversion under indistinctly blue or white irradiation. Additionally, tetrabutylammonium decatungstate (TBADT) was tested, as it can operate not only via a SET mechanism but also through hydrogen atom transfer (HAT) depending on the redox properties of the substrate.^[^ ^47^ ^]^ This resulted in a complex reaction mixture since together with **1a** and **2a** the corresponding dehalogenation product **3a** was observed in significant amounts (entry 5).

**Table 1:** Screening of photocatalysts and irradiation source in the oxidation reaction of halohydrin **1a** to haloketone **2a**.

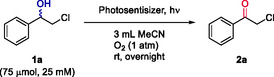				
Entry	Photosensitizer	*E* ^T1^_red_ (V vs. SCE)a)	Irradiation	**2a** [%]b)
1	9‐Fluorenone (20 mol%)	+0.96	Blue	3
2	SAS (20 mol%)	+1.80	Blue	65
3	TPPT (20 mol%)	+2.02	Blue or White	>99
4c)	DDQ (1 equiv.)	+3.18	Blue or White	>99
5d)	TBADT (30 mol%)	+2.44	390 nm	25

a) Excited triplet state reduction potentials versus saturated calomel electrode (SCE).^[^ ^43^, ^44^ ^]^

b) Conversion values were calculated by gas chromatography (GC) analysis.

c) The reaction mixture was carried out under atmospheric air.

d) The following mixture of products was obtained: **2a** (25%), **1a** (25%), and acetophenone (**3a**, 50%).

Once TPPT and DDQ were identified as efficient photocatalysts under white light irradiation, an optimization of the reaction conditions was undertaken, focusing on the later combination with the bioreduction step (Tables S2–S7, Supporting Information). Acetonitrile was selected as a solvent for its compatibility with the subsequent biocatalytic step as most enzymes, including ADHs, are tolerant to it. Also, the photo‐oxidation reactions were tested in both H_2_O and several MeCN:H_2_O mixtures (Table S18, Supporting Information). Unfortunately, both photosensitizers, TPPT and DDQ, were unable to efficiently work in the presence of water.

**Figure 1:**
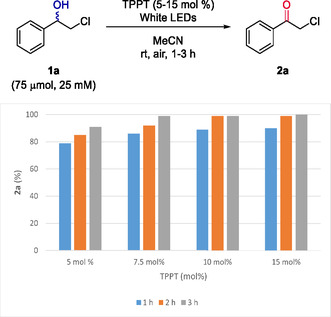
Optimization of the catalytic amount of TPPT and the reaction time in the photo‐oxidation of halohydrin **1a** (25 mM) to haloketone **2a**.

Optimization of the reaction conditions with each photosensitizer was carried out to ensure maximum efficiency. On the one hand, the photo‐oxidation reaction with TPPT relies on molecular oxygen to regenerate the photocatalytic action, confirming that atmospheric oxygen was sufficient for this process. To optimize the reaction conditions, studies were conducted monitoring the reaction progress over time at various catalytic loadings using a 25 mM concentration of **1a** (**Figure** **1** and Table S2, Supporting Information), attaining complete oxidation after 3 h under white light irradiation and air atmosphere using 10 or 15 mol% of TPPT. Interestingly, by increasing the substrate concentration to 75 mM it was possible to reduce the TPPT loading (7.5 mol%) and the reaction time (1 h) for the complete formation of α‐haloketone **2a** (Table S3, Supporting Information).

On the other hand, DDQ cannot be regenerated by molecular oxygen and requires more specific regeneration systems.^[^ ^46^ ^]^ An optimization study of the oxidation of **1a** at different concentrations, varying the amounts of DDQ and the reaction time can be found in Table S4, Supporting Information. Remarkably, a complete photo‐oxidation of **1a** (75 μmol, 75 mM) was achieved after 4 h under white light irradiation in air atmosphere using MeCN as solvent (**Table** **2**, entries 1–3). In addition, the regeneration of DDQ was carefully analyzed by evaluation of previously reported regeneration systems such as HNO_3_/O_2_^[^ ^48^ ^]^ (entries 4–6, further studies in Table S5, Supporting Information), MnO_2_/O_2_^[^ ^46^ ^]^ (entries 7 and 8, further studies in Table S6, Supporting Information), and *tert*‐butyl nitrite (TBN)/O_2_^[^ ^49^ ^]^ (entries 9–11, further studies in Table S7, Supporting Information). Above all, the combination of TBN and molecular oxygen proved to be the most suitable. After optimization, the loading of DDQ was reduced to 5 mol% employing 0.5 equivalents of TBN under air atmosphere which achieved complete oxidation of **1a** (75 μmol, 75 mM, entry 11).

**Table 2:** Regeneration systems of DDQ for the photo‐oxidation of halohydrin **1a**.

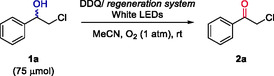					
Entry	DDQ (equiv.)	Regeneration System	t [h]	MeCN [mL]a)	**2a** [%]b)
1	0.8	none	16	3 (25 mM)	82
2	1.2	none	8	3 (25 mM)	>99
3	1	none	4	1 (75 mM)	>99
4	0.5	HNO_3_ (0.27 equiv.)c)	16	3 (25 mM)	26
5	0.5	HNO_3_ (0.53 equiv.)c)	16	3 (25 mM)	14
6	0.5	HNO_3_ (0.8 equiv.)c)	16	3 (25 mM)	5
7	0.5	MnO_2_ (0.5 equiv.)	16	3 (25 mM)	60
8	0.5	MnO_2_ (1 equiv.)	16	3 (25 mM)	62
9	0.5	TBN (1.5 equiv.)	4	1 (75 mM)	>99
10	0.05	TBN (1.5 equiv.)	4	1 (75 mM)	>99
11	0.05	TBN (0.5 equiv.)	4	1 (75 mM)	>99

a) Concentration of halohydrin **1a** in parentheses.

b) Conversion values were calculated by GC analysis.

c) HNO_3_ 1 N was used.

To sum up, three photochemical conditions for the oxidation of chlorohydrin **1a** were reported, which include the use of catalytic TPPT (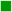 **Method A**), stoichiometric DDQ (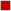 **Method B**), or catalytic DDQ coupled with its regeneration system TBN/O_2_ (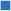 **Method C**).

### 2.2 Scope of the Photo‐Oxidation of β‐Chlorohydrins

Racemic β‐halohydrins **1a**–**t** were considered for broadening the oxidation study to have a clear overview about the possibilities of the photochemical approach previously optimized with substrate **1a** using different photosensitizers and conditions. The set of substrates includes 2‐halo‐1‐arylethanols considering chlorine and bromine as halogen atoms (**1a** and **1c**), the presence of a methyl substitution or an additional chlorine at the C‐2 position of the aliphatic chain (**1b** and **1d**), and both electron‐withdrawing or electron donor substituents at different positions of the aryl ring (**1e**–**t**). The study of 2‐halo‐1‐heteroarylethanols was discarded due to the difficulties in their synthesis when considering, for instance, pyridyl, thienyl, and furyl derivatives.

**Scheme 2:**
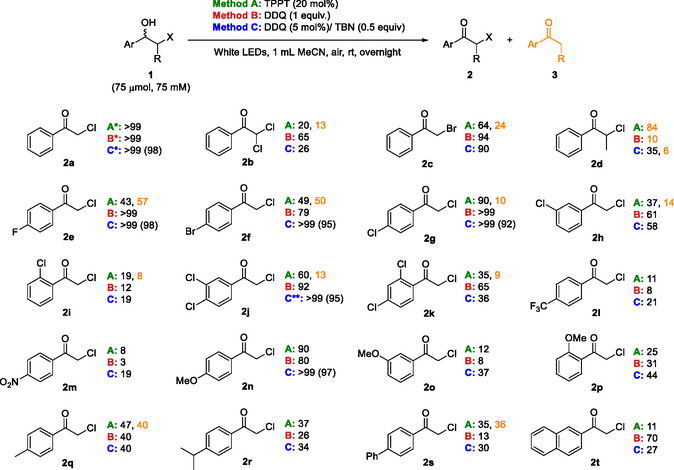
Photo‐oxidation of halohydrins **1a–t** using three different oxidative systems: 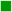 **Method A:** TPPT (20 mol%), 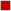 **Method B:** DDQ (1 equiv.), or 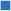 **Method C:** DDQ (5 mol%) and TBN (0.5 equiv.), under white light irradiation, air atmosphere, and room temperature overnight. Conversion toward haloketone **2** is reported. Conversion toward dehalogenation byproduct **3** is reported 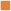. *Deviations for **1a**: TPPT 7.5 mol% and 1 h in **Method A**; 4 h in **Methods B** and **C**. **Deviations for **1j**: DDQ 50 mol% and TBN 1.5 equiv. in **Method C**. Additional information can be found in Table S8, Supporting Information.

The results obtained using the three oxidative methodologies **A**, **B,** and **C** are disclosed in **Scheme** **2**. First, the use of TPPT as photosensitizer (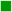 **Method A**) was assessed. Compared to the model substrate **1a**, it was necessary to increase the catalytic loading to 20 mol% and extend overnight the reaction time to achieve synthetically useful results. Unfortunately, dehalogenation by‐product formation was detected in most cases, especially with substrate **1d** (84% conversion to **3d**). Notably, *para*‐halogenated chlorohydrins **1e**–**g** yielded the desired ketones **2e**–**g** without traces of alcohol, being the fluorinated one **2e** the most successful with the lowest by‐product formation. Other substrates, especially the *para*‐methoxy **2n** gave good conversions, but traces of alcohol were also detected.

Second, the applicability of stoichiometric DDQ as photosensitizer was evaluated and compared to the model substrate **1a**; the reaction time was prolonged overnight (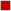 **Method B**). On the one hand, DDQ serves as a stronger oxidant than TPPT due to its higher excited‐state reduction potentials,^[^ ^44^ ^]^ leading to better results in most cases, as complete conversion to the ketone was observed with substrates **1e** and **1g**, while promising results were also achieved with substrates **1c**,**f**,**j**,**n**. Satisfyingly, DDQ did not generate dehalogenation by‐products, with the exception of compound **1d**. This enhancement underscores the advantages of utilizing DDQ in the oxidation process compared to TPPT.

Finally, the applicability of catalytic DDQ as a photosensitizer in combination with the TBN/O_2_ regeneration system was evaluated (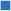 **Method C**), observing a further improvement as TBN/O_2_ enhances the oxidative power of DDQ. Compared to the model substrate **1a**, the reaction time needed to be extended overnight to achieve optimal outcomes. Complete conversions were observed with *para*‐halosubstituted substrates **1e–g** and for the *para*‐methoxy substituted **1j**. Also, good conversions were achieved with bromohydrin **1c** and *para*,*meta*‐dichlorosubstituted **1j**. Given its relevance in pharmaceutical chemistry,^[^ ^50^ ^]^ substrate **1j** was further optimized, achieving full conversion using 50 mol% DDQ and 1.5 equivalents of TBN (see Table S11, Supporting Information). Additionally, the use of other catalytic loadings was studied using **1l** as a model substrate to improve the oxidation of highly electron‐deficient alcohols. However, no significant improvement was observed under these conditions (see Table S10, Supporting Information). It is worth mentioning that no conversion was found when attempting the oxidation of an aliphatic derivative such as 1‐chlorooctan‐2‐ol.

Based on the so‐obtained results, catalytic DDQ with the TBN/O_2_ regeneration system (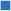 **Method C**) was selected as the best approach. This method successfully yielded six chloroketones **2a**,**e**,**f**,**g**,**j**,**n** (R = H, 4‐F, 4‐Br, 4‐Cl, 3,4‐Cl_2_, and 4‐OMe) with complete conversion, making it the most suitable choice for further application in the desired deracemization methodology.

**Figure 2:**
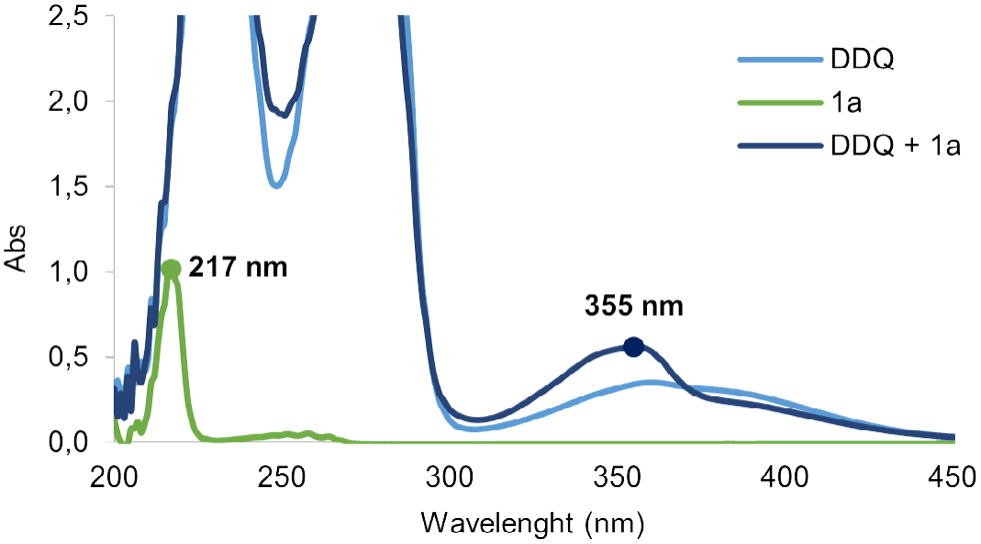
UV–VIS spectrometry studies on the formation of an EDA complex between substrate **1a** and DDQ; a bathochromic shift was observed denoting an EDA complex formation.

UV–VIS spectrometry studies uncovered the formation of an electron donor–acceptor (EDA) complex through the reaction outcome only when employing DDQ (**Figure** **2**); this was not observed when using TPPT (Figure S7, Supporting Information).^[^ ^51^, ^52^ ^]^ Substrate **1a** exhibits an absorption band at *λ*_max_ = 218 nm. The addition of 1 equivalent of DDQ resulted in a bathochromic shift toward *λ*_max_ = 355 nm. These data are consistent with the formation of an EDA complex between substrate **1a** and DDQ. EDA complexes are characterized by the appearance of an absorption band that is redshifted compared to the absorption bands of the individual components. We propose that the excitation of this charge transfer band promotes the initial electron transfer from **1a** to DDQ. The proposed mechanisms for both catalytic systems are discussed in detail in the Supporting Information (Section 4.6) and are illustrated in (Schemes S2 and S3, Supporting Information).

Both catalytic systems, TPPT and DDQ, proceed through an initial SET step, followed by an acid–base reaction and a HAT step. We hypothesize that the involvement of DDQ in forming an EDA complex and its subsequent participation in both the acid–base and HAT steps may provide a more controlled mechanistic environment, limiting side reactions such as dehalogenation. In contrast, TPPT does not engage in these later steps and depends on external agents as superoxide (O_2_•^−^) specie or hydroperoxyl radical (HOO•), leading to a more open radical pathway that could increase the chances of dehalogenation. However, this hypothesis was not experimentally demonstrated to prevent dehalogenation; therefore, it is still not fully clear why dehalogenation is more pronounced with TPPT than with DDQ.

Additionally, we hypothesize that the likelihood of dehalogenation correlates with the stability of the radical intermediate, particularly at the C‐2 position, as it was clearly observed with substrate **1d**.

### 2.3 Applications of the Photo‐Oxidation of β‐Chlorohydrins

Building on the successful achievement of finding mild reaction conditions for oxidizing β‐chlorohydrins through a photochemical approach, the previously reported methodologies were further investigated to expand their applicability. The following subsections will address, not only the goal of developing a linear deracemization process combining photo‐ and biocatalysis, but also exploring sustainable approaches such: as 1) the use of sunlight as irradiation source; 2) the implementation of flow experiments; and 3) applications in organic synthesis to facilitate the oxidation of additional compounds, thereby broadening the scope of the presented methodology.

### 2.4 Solar‐Driven Photo‐Oxidation Reaction

**Figure 3:**
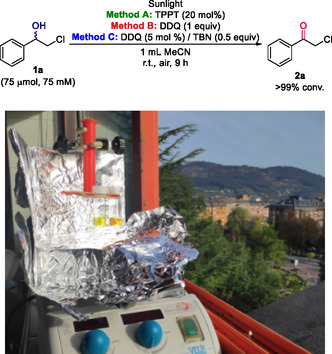
Setting of the sunlight‐driven oxidation of 2‐chloro‐1‐phenyletanol (**1a**) in the window of our laboratory at the University of Oviedo.

The sun is the most sustainable light source available on our planet; therefore, the direct use of sunlight for photochemistry is extremely appealing when looking for more economical and less energy‐consuming transformations. The sun‐driven oxidation of 2‐chloro‐1‐phenylethanol (**1a**) was studied by setting our reactions in the window of our laboratory for 9 h, achieving in all cases complete conversion to ketone **2a** (**Figure** **3**).

### 2.5 Continuous Flow Experiment

**Figure 4:**
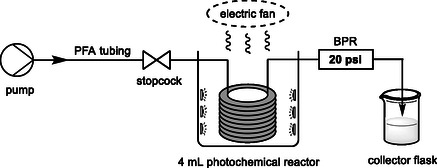
Setting of the continuous flow system for the photo‐oxidation of 2‐chloro‐1‐phenyletanol (**1a**).

Next, the design of a continuous flow system for the oxidation of **1a**–**2a** was accomplished. Although further details can be found in Section 5 of the Supporting Information, a diagram of the equipment has been represented in **Figure** **4**. Given that our continuous flow system lacks support for a gas inlet and since O_2_ is crucial for the reported catalytic systems, only stoichiometric DDQ (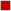 **Method B**) led to full oxidation at a flow rate (Q) of 1 mL h^−1^. The space‐time yield (STY) is a measurement of the productivity of the reaction and is given by the equation STY= P/(V·t), where *P* is the amount of product formed (in μmol), *V* is the reaction volume (in mL), and *t* is the reaction time (in hours). The photo‐oxidation reaction of **1a** under the best batch conditions (Table S23, Supporting Information entry 6) corresponds to STY of 50 μmol mL^−1^ h^−1^. The implementation of the continuous flow system resulted in a significant increase in the STY value to 300 μmol mL^−1^ h^−1^ (Table S15, Supporting Information entry 3).

### 2.6 Photo‐Oxidation of (2‐Chloroethyl)Benzene

At this point, trying to give additional value to our methodology on the oxidation of alkyl halides, our methodologies were applied to the photo‐oxidation of (2‐chloroethyl)benzene (**4a**). The best results were achieved when using TPPT (20 mol%) under blue light irradiation overnight, yielding a moderate although significant 51% conversion (**Table** **3**, entry 1). In comparison, similar results were reported by Wu and coworkers when describing the oxyfunctionalization of a series of benzylic substrates, obtaining ketone **2a** in 53% yield with SAS (20 mol%) in a water‐MeCN (70:30 v/v) system after 18 h and under blue light.^[^ ^53^ ^]^

**Table 3:** Oxidation of chlorohydrin **1a** using catalytic DDQ.

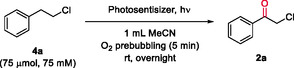			
Entry	Photosensitizer	Irradiation	**2a** [%]a)
1	TPPT (20 mol%)	Blue	51
2	TPPT (20 mol%)	White	34
3	DDQ (1 equiv.)	Blue	11
4	DDQ (1.1 equiv.)	White	24
5	DDQ (5 mol%)/TBN (0.5 equiv.)	Blue	5
6	DDQ (5 mol%)/TBN (0.5 equiv.)	White	20

a) Conversion values were calculated by high performance liquid chromatography analysis.

### 2.7 Photobiocatalytic One‐Pot Two‐Step Deracemization of β‐Chlorohydrins

Multistep cascade processes avoid intermediate isolation, generally leading to higher yields in comparison with stepwise approaches. Furthermore, the complete oxidation of halohydrins allows the possibility to obtain them in enantiopure form by performing a one‐pot oxidation‐reduction sequence, process that is known as deracemization.^[^ ^33^, ^34^, ^35^, ^36^, ^37^, ^38^, ^54^, ^55^, ^56^, ^57^ ^]^ Up to now, two photobiocatalytic deracemizations of secondary alcohols have been reported in the literature by performing a photo‐oxidation and bioreduction sequence. Wu and coworkers described the use of SAS as photocatalyst for the oxidation of 1‐arylethanols including the successful use of a halohydrin such as 2‐fluoroacetophenone,^[^ ^41^ ^]^ while Borowieki and coworkers described the use of 9‐fluorenone for the deracemization of (het)aryl, allylic and aliphatic substrates.^[^ ^42^ ^]^ In our attempts to employ these methodologies, both failed on deracemizing β‐chlorohydrins due to the difficulty of oxidizing these substrates. Having in our hands six substrates that were completely oxidized with the disclosed photochemical strategy, a photobiocatalytic deracemization was envisioned by the selection of highly stereoselective ADHs. Two ADHs overexpressed in *E. coli* were selected for the stereoselective bioreduction step due to their capability to reduce halohydrins with complete selectivity: ADH from *Rhodococcus ruber* (ADH‐A)^[^ ^58^ ^]^ and the one from *Lactobacillus brevis* (*Lb*ADH).^[^ ^59^ ^]^ Bioreduction experiments, under standard conditions, using a Tris HCl buffer, 50 mM and pH 7.5 with isopropanol (2‐PrOH, 5% vol) for cofactor recycling purpose were employed, observing the complete consumption of ketones **2a**,**e**,**f**,**g**,**j**,**n** and total selectivity toward the formation of alcohols (*R*)‐ and (*S*)‐**2a**,**e**,**f**,**g**,**j**,**n**, respectively (Table S17, Supporting Information).

At this point, the key challenge was how to merge the initial photochemical oxidation and the subsequent bioreduction in a one‐pot process, particularly since hydroxyl oxidation was performed in acetonitrile (MeCN) while the enzymatic carbonyl reduction requires an aqueous medium. To address this, bioreductions of **2a** with each enzyme were tested at varying acetonitrile concentrations, with the maximum MeCN tolerated without loss of activity and selectivity being 7.5% vol for ADH‐A (Table S19, Supporting Information) and 70% vol for *Lb*ADH (Table S20, Supporting Information). Moreover, to fulfill pH and ketone concentration requirements in the bioreduction step, the photochemical oxidation was reoptimized by reducing the acetonitrile volume to 250 μL (**1a** 302 mM), achieving complete conversion in 6 h (Table S23, Supporting Information entry 6).

**Scheme 3:**
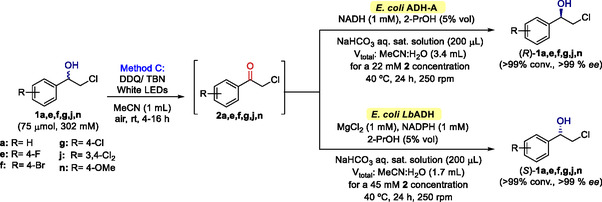
Photobiocatalytic one‐pot sequential deracemization strategy for chlorohydrins **1a,e,f,g,j,n**.

Ultimately, the deracemization of racemic **1a**,**e**,**f**,**g**,**j**,**n** β‐chlorohydrins was successfully achieved using a two‐step, one‐pot strategy. First, the corresponding racemic alcohol **1** was photo‐oxidized using DDQ (5–50 mol%) with TBN (0.5–1.5 equivalents)/O_2_ as a regeneration system in 250 μL MeCN and irradiated with white light for 6–12 h. Then, the pH and ketone concentration of the resulting mixture were adjusted using a NaHCO_3_‐saturated aqueous solution, requiring dilution to 45 mM for *Lb*ADH and 22 mM for ADH‐A (**Scheme** **3**). Finally, after adding 2‐PrOH (5% vol), cofactors (1 mM), and the ADH, bioreduction was carried out at 250 rpm and 40 °C for 24 h, achieving full conversion and total enantiomeric excess of (*R*)‐ and (*S*)‐β‐chlorohydrins. Semipreparative experiments were successfully performed using 0.753 mmol of racemic chlorohydrin **1a** (118 mg), yielding the chlorohydrins (*R*)‐ and (*S*)‐**1a** with quantitative yield and enantiopurity after liquid–liquid extraction using the ADH‐A and the *Lb*ADH, respectively.

### 2.8 Photo‐Bio‐Chemoenzymatic Sequential Cascade for the Production of Styrene Oxide Enantiomers

**Scheme 4:**
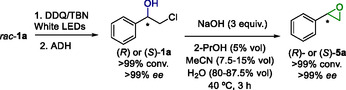
One‐pot three‐step stereoselective photo–bio–chemo sequence for the synthesis of styrene oxide (**5a**) enantiomers from racemic 2‐chloro‐1‐phenylethanol (**1a**).

As mentioned, β‐halohydrins are highly valuable compounds due to their potential for transformation into various important derivatives, so at this point, the derivatization into enantiopure styrene oxides (**5a**) was envisioned by coupling an additional chemical epoxidation step by the addition of a base.^[^ ^16^ ^]^ Preliminary studies were performed using racemic halohydrin **1a** to find the minimum amount of NaOH to form **5a** with full conversion after 3 h at 40 °C (Table S25, Supporting Information). Then, the desired photo‐bio‐chemo sequence was studied in a one‐pot three‐step fashion as depicted in **Scheme** **4**, requiring in this case 3 equiv. of NaOH to form **5a** enantiomers with complete conversion and without loss of their optical purity. Therefore, both styrene oxide (**5a**) enantiomers were obtained in high purity depending on the enzyme of choice (Table S26, Supporting Information for more details of the cascade optimization), (*S*)‐**5a** with *Lb*ADH (>99% conversion and >99% *ee*) and (*R*)‐**5a** for ADH‐A (>99% conversion and >99% *ee*). This simple protocol highlights the importance of our deracemization process, disclosing new synthetic pathways for accessing valuable compounds.

## 3 Conclusion

A photobiocatalytic sequential approach has been accomplished by means of light irradiation synthesis of α‐haloketones from the corresponding racemic halohydrins, finding the use of TPPT in catalytic amounts, DDQ in stochiometric loading or alternatively DDQ in catalytic doses using TBN/O_2_ as regeneration system. The generality of the oxidative reaction has been highly dependent on the pattern substitutions at the aromatic ring when considering 2‐halo‐1‐arylethanols, producing six α‐haloketones in quantitative yield. The search for sustainable methods to obtain the desired α‐haloketones has been deeply explored by also describing the performance of a sunlight‐driven oxidation process, the design of a continuous flow oxidative process, and the possibility to oxidize alkyl halides.

Taking advantage of merging different types of catalysts, and once several 2‐halo‐1‐arylethanones were produced in quantitative conversions, the design of a one‐pot two‐step deracemization process was demonstrated by sequentially coupling a bioreduction step after the photocatalytic oxidative process. ADH‐A from *Rhodococcus ruber* and the one from *Lactobacillus brevis* have allowed the photoenzymatic synthesis of the 12 possible 2‐chloro‐1‐arylethanols in enantiopure form. Finally, a three‐step one‐pot sequential cascade was described, which includes a final epoxidation reaction controlling the halohydrin concentration and the equivalents of NaOH, thus selectively leading to enantiopure styrene oxide enantiomers depending on the ADH of choice for the bioreduction step.

## Conflict of Interest

The authors declare no conflict of interest.

## Supporting information

Supplementary Material

## Data Availability

The data that support the findings of this study are available from the corresponding author upon reasonable request.
