# Supplementary material for: Combining Photocatalytic Oxidation of β‐Chlorohydrins with Carbonyl Bioreduction in a Deracemization Approach
Source: ChemSusChem. 2025 Jun 29;18(15):e202500683. doi: 10.1002/cssc.202500683 (PMC12302324; doi:10.1002/cssc.202500683)

Combining photocatalytic oxidation of β-chlorohydrins with carbonyl bioreduction in a deracemization approach

Sara Filgueira, Laura Rodríguez-Fernández, Iván Lavandera and Vicente Gotor-Fernández*

Organic and Inorganic Chemistry Department. Instituto Universitario de Química Organometálica "Enrique Moles". University of Oviedo. Avenida Julián Clavería 8, 33006 Oviedo. Spain.

E-mail: vicgotfer@uniovi.es (V.G.-F.).

Supporting Information

Index

1. Compounds studied in this contribution S3

2. General considerations S4

3. Synthesis and characterization of racemic halohydrins S5

3.1. Experimental protocol for the synthesis of haloketones 2d,h,i,l,m,o-t S5

3.2. Experimental protocol for the synthesis of racemic halohydrins 1a-t S7

4. Photo-oxidation of halohydrins S12

4.1. Description of the photochemistry setups S12

4.2. Optimizing the photo-oxidation reaction S14

4.2.1. Method A: using TPPT in catalytic amount S16

4.2.2. Method B: using DDQ in stoichiometric amount S17

4.2.3. Method C: using DDQ in catalytic amount S18

4.3. Substrate scope of the photo-oxidation reaction S20

4.4. Experimental protocol for the photo-oxidation of halohydrins 1a-t S24

4.5. Solar-driven photo-oxidation reaction S26

4.6. Mechanistic studies S27

5. Photo-oxidation reaction in continuous flow S31
5.1. Description of the continuous flow photochemistry system S31
5.2. Optimizing the photo-oxidation reaction in continuous flow S33
5.3. Experimental protocol for the photo-oxidation in continuous flow of halohydrin 1a S33
6. Photo-oxidation of (2-chloroethyl)benzene to obtain the corresponding haloketone 2a S34
7. Asymmetric biocatalytic reduction of haloketones S35
7.1. Experimental protocol for the biocatalytic reduction of haloketones 2a,e-g,j,n using ADH-A S36
7.2. Experimental protocol for the biocatalytic reduction of haloketones 2a,e-g,j,n using *Lb*ADH S36
8. One-pot two-step sequential photo-biocatalytic deracemization of halohydrins S37
8.1. Optimizing the photo-biocatalytic deracemization S37
8.2. Substrate scope of the photo-biocatalytic deracemization S41
8.3. Experimental protocol for the photo-biocatalytic deracemization of 2a,e-g,j,n S42
8.4. General procedure for the deracemization of halohydrin 1a in batch and semipreparative scale S42
9. Enantiopure epoxide formation sequence S43
9.1. Experimental protocol for the enantiopure epoxide formation sequence S44
10. Analytical data S45
10.1. GC analyses S45
10.2. HPLC analyses S44
11. References S55
12. NMR spectra S57

1. Compounds studied in this contribution

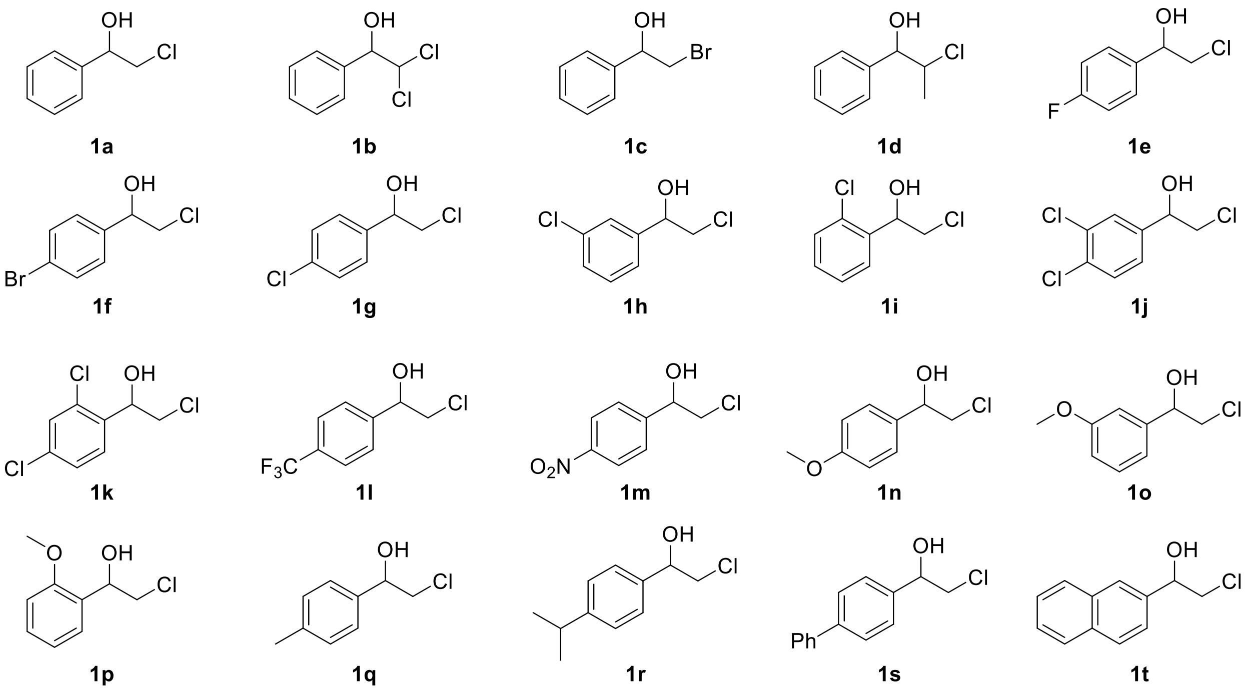

Figure S1. Structure of halohydrins **1a-t** studied in this contribution.

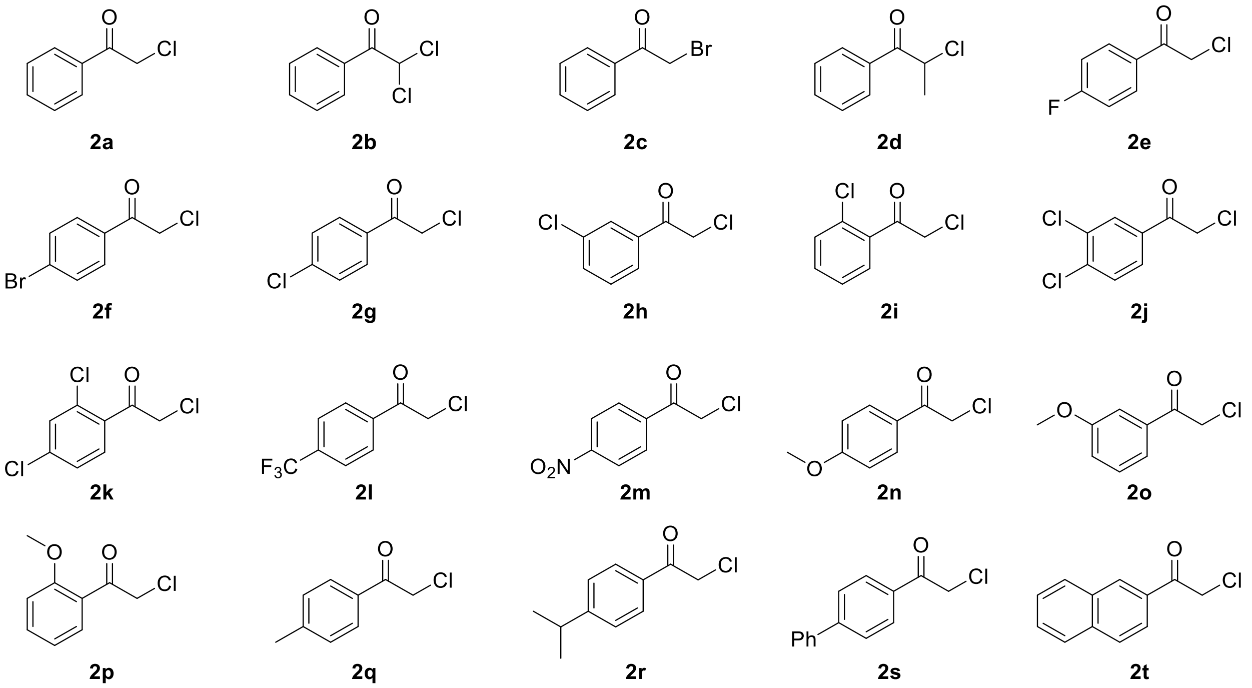

Figure S2. Structure of haloketones **2a-t** studied in this contribution.

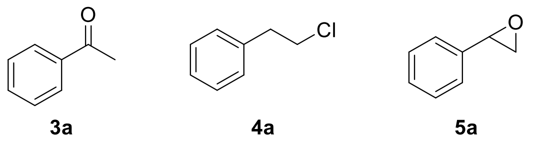

Figure S3. Other compounds in this contribution.

2. General considerations

Chemical reagents, organic solvents and nicotinamide cofactors (NAD^+ and $NADP^+$) were purchased from different commercial sources and used as received. Racemic halohydrins **1a-t** were chemically synthesised, exhibiting spectral data in agreement with those reported in the literature, see Section 3 for synthetic procedures and characterisation data.

In-house alcohol dehydrogenases (ADHs) were obtained as previously reported in the literature from lyophilised *E. coli* cells heterologously overexpressing ADHs from *Rhodococcus ruber* (ADH-A), *Lactobacillus brevis* (*Lb*ADH) or *Thermoanaerobacter* sp. (ADH-T).[1–3]

The photochemistry setup was composed by an irradiation source, a cooling fan and a magnetic stirrer. The irradiation source consisted of either a white LED (Light Emitting Diode) strip lights or two Kessil® blue LED lamps (H150W-blue and H160 Tuna Flora). Photochemical reactions were performed in 4 or 25-mL glass vials with screw cap. The detailed description of the setup is described in Section 4.1.

The continuous flow system was built with perfluoroalkoxy (PFA) tubing. The reaction solution was pumped into the flow system by a syringe pump and passed through a stopcock, a photochemical reactor and a 20 psi back pressure regulator (BPR) with its outlet connected to the collector flask. The detailed description of the setup is described in Section 5.1.

Nuclear magnetic resonance (NMR) spectra were recorded on a Bruker AV-300. All chemical shifts (δ) are reported in parts per million (ppm) and properly referenced to the residual solvent peak of ($CDCl_3$). Coupling constants (*J*) are specified in hertz (Hz). Chemical shifts are displayed as follows: chemical shift (multiplicity, coupling constant(s) when applicable, number of protons). The assignment of the ^{13}C-NMR spectra has been carried out by means of DEPT-135 experiments.

Thin-layer chromatography (TLC) analyses for the monitorization of reactions were conducted with silica gel 60 F254 precoated plates and visualised with both UV lamp and vanillin stain. Purification of synthesized compounds were carried out by column chromatographies using silica gel 60 (230-240 mesh) as stationary phase.

3. Synthesis and characterization of racemic halohydrins

Racemic β-halohydrins **1a**,**c-t** and β,β-dichlorohydrin **1b** studied in this work (Figure S1) were prepared by reducing the corresponding haloketones **2a-t** (Figure S2). Some of these α-haloketones were already commercially available, while others (**2d**,**h**,**i**,**l**,**m**,**o-t**) needed to be synthesized by chlorination of their ketone precursors.

3.1. Experimental protocol for the synthesis of haloketones 2d,h,i,l,m,o-t

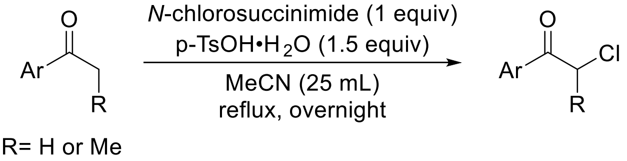

To a solution of the corresponding ketone precursor (1 equiv, 5 mmol) in MeCN (25 mL), *N*-chlorosuccinimide (1 equiv) and *p*-toluenesulfonic acid monohydrate (1.5 equiv) were added. The reaction solution was stirred under reflux overnight. Then, the solvent was concentrated under vacuum and the residue purified by column chromatography on silica gel leading to the corresponding α-haloketone **2**.[4]

For compound **2q**, a modified method was used. In this protocol, the solvent used was MeOH instead of MeCN, thiourea (0.02 equiv) instead of *p*-toluenesulfonic acid monohydrate and the reaction was carried out at room temperature.

For compound **2t**, CH_2Cl_2 (5 mL) was also added to the reaction medium improving the substrate solubility.

Spectroscopic data of the obtained compounds agreed with those previously reported.[5-10]

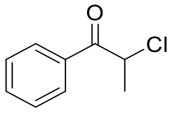

2-Chloro-1-phenylpropanone (2d):[5] Colorless oil (24% isolated yield). R_f (Hexane/CH_2Cl_2 8:5) 0.56. **^{1}H-NMR** (300 MHz, $CDCl_3$) δ 8.04–8.00 (m, 2H), 7.60 (tt, J = 6.7, 1.3 Hz, 1H), 7.52–7.46 (m, 2H), 5.26 (q, J = 6.7 Hz, 1H), 1.74 (d, J = 6.7 Hz, 3H). **^{13}C-NMR** (75 MHz, $CDCl_3$) δ 193.7 (C), 134.2 (C), 133.9 (CH), 129.1 (2CH), 128.9 (2CH), 52.9 (CH), 20.1 (CH_3).

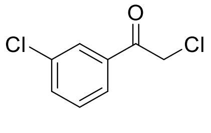

2-Chloro-1-(3-chlorophenyl)ethanone (2h):[6] Colorless oil (66% isolated yield). R_f (Hexane/CH_2Cl_2 8:5) 0.38. **^{1}H-NMR** (300 MHz, $CDCl_3$) δ 7.92 (t, J = 1.8 Hz, 1H), 7.82 (ddd, J = 7.7, 1.7, 1.1 Hz, 1H), 7.58 (ddd, J = 8.0, 2.1, 1.1 Hz, 1H), 7.44 (t, J = 7.9 Hz, 1H), 4.68 (s, 2H). **^{13}C-NMR** (75 MHz, $CDCl_3$) δ 190.5 (C), 136.2 (C), 135.8 (C), 134.5 (CH), 130.8 (CH), 129.1 (CH), 127.1 (CH), 46.3 (CH_2).

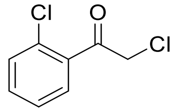

2-Chloro-1-(2-chlorophenyl)ethanone (2i):[6] Colorless oil (48% isolated yield). R_f (Hexane/CH_2Cl_2 8:5) 0.40. **^{1}H-NMR** (300 MHz, $CDCl_3$) δ 7.56–7.52 (m, 1H), 7.45–7.42 (m, 2H), 7.38–7.32 (m, 1H), 4.69 (s, 2H). **^{13}C-NMR** (75 MHz, $CDCl_3$) δ 194.3 (C), 136.2 (C), 132.9 (CH), 131.4 (C), 130.7 (CH), 130.0 (CH), 127.3 (CH), 48.8 (CH_2).

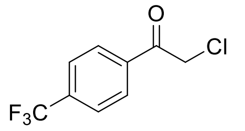

2-Chloro-1-[4-(trifluoromethyl)phenyl]ethanone (2l):[5] White solid (42% isolated yield). R_f (Hexane/CH_2Cl_2 8:5) 0.45. **^{1}H-NMR** (300 MHz, $CDCl_3$) δ 8.07 (d, J = 8.1 Hz, 2H), 7.76 (d, J = 8.1 Hz, 2H), 4.71 (s, 2H). **^{13}C-NMR** (75 MHz, $CDCl_3$) δ 190.5 (C), 136.9 (C), 135.3 (q, J = 32.8 Hz, CF_3), 129.1 (2CH), 126.1 (q, J = 3.8 Hz, 2CH), 121.7 (C), 45.9 (CH_2). **^{19}F{^{1}H}-NMR** (282 MHz, $CDCl_3$) δ –62.9.

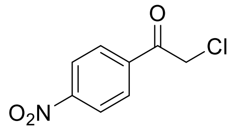

2-Chloro-1-(4-nitrophenyl)ethanone (2m):[7] Colorless oil (36% isolated yield). R_f (Hexane/EtOAc/CH_2Cl_2 8:1:5) 0.22. **^{1}H-NMR** (300 MHz, $CDCl_3$) δ 8.38–8.34 (m, 2H), 8.16–8.12 (m, 2H), 4.71 (s, 2H). **^{13}C-NMR** (75 MHz, $CDCl_3$) δ 190.0 (C), 150.9 (C), 138.7 (C), 129.9 (2CH), 124.2 (2CH), 45.7 (CH_2).

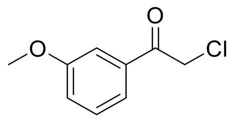

2-Chloro-1-(3-methoxyphenyl)ethanone (2o):[6] White solid (52% isolated yield). R_f (Hexane/CH_2Cl_2 8:5) 0.47. **^{1}H-NMR** (300 MHz, $CDCl_3$) δ 7.53–7.48 (m, 2H), 7.40 (t, J = 7.9 Hz, 1H), 7.16 (ddd, J = 8.2, 2.7, 1.0 Hz, 1H), 4.71 (s, 2H), 3.86 (s, 3H). **^{13}C-NMR** (75 MHz, $CDCl_3$) δ 191.0 (C), 160.1 (C), 135.7 (C), 130.0 (CH), 121.1 (CH), 120.6 (CH), 112.9 (CH), 55.7 (CH_3), 46.3 (CH_2).

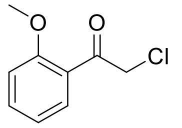

2-Chloro-1-(2-methoxyphenyl)ethanone (2p):[7] White solid (13% isolated yield). R_f (Hexane/CH_2Cl_2 8:5) 0.15. **^{1}H-NMR** (300 MHz, $CDCl_3$) δ 7.83 (dd, J = 7.8, 1.9 Hz, 1H), 7.49 (ddd, J = 8.4, 7.3, 1.9 Hz, 1H), 7.02–6.95 (m, 2H), 4.75 (s, 2H), 3.90 (s, 3H). **^{13}C-NMR** (75 MHz, $CDCl_3$) δ 192.1 (C), 159.0 (C), 134.9 (CH), 131.2 (CH), 124.7 (C), 121.0 (CH), 111.6 (CH), 55.7 (CH_3), 51.3 (CH_2).

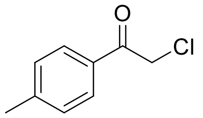

2-Chloro-1-(4-methylphenyl)ethanone (2q):[6] White solid (61% isolated yield). R_f (Hexane/CH_2Cl_2 4:1) 0.16. **^{1}H-NMR** (300 MHz, $CDCl_3$) δ 7.86 (d, J = 8.3 Hz, 2H), 7.29 (d, J = 8.0 Hz, 2H), 4.69 (s, 2H), 2.43 (s, 3H). **^{13}C-NMR** (75 MHz, $CDCl_3$) δ 190.8 (C), 145.2 (C), 131.9 (C), 129.7 (2CH), 128.8 (2CH), 46.1 (CH_2), 21.9 (CH_3).

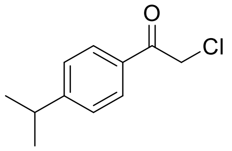

2-Chloro-1-(4-isopropylphenyl)ethanone (2r):[8] Colorless oil (36% isolated yield). R_f (Hexane/EtOAc/CH_2Cl_2 8:1:5) 0.67. **^{1}H-NMR** (300 MHz, $CDCl_3$) δ 7.86 (d, J = 8.4 Hz, 2H), 7.31 (d, J = 8.4 Hz, 2H), 4.68 (s, 2H), 2.94 (hept, J = 6.9 Hz, 1H), 1.24 (d, J = 6.9 Hz, 6H). **^{13}C-NMR** (75 MHz, $CDCl_3$) δ 190.7 (C), 155.7 (C), 132.0 (C), 128.7 (2CH), 127.0 (2CH), 46.1 (CH_2), 34.3 (CH), 23.5 ($2CH_3$).

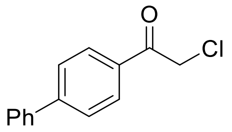

1-[(1,1'-Biphenyl)-4-yl]-2-chloroethanone (2s):[9] White solid (25% isolated yield). R_f (Hexane/CH_2Cl_2 1:1) 0.49. **^{1}H-NMR** (300 MHz, $CDCl_3$) δ 8.04 (d, J = 8.4 Hz, 2H), 7.72 (d, J = 8.4 Hz, 2H), 7.65–7.62 (m, 2H), 7.52–7.39 (m, 3H), 4.74 (s, 2H). **^{13}C-NMR** (75 MHz, $CDCl_3$) δ 190.8 (C), 146.8 (C), 139.6 (C), 133.0 (C), 129.3 (2CH), 129.2 (2CH), 128.6 (CH), 127.6 (2CH), 127.4 (2CH), 46.1 (CH_2).

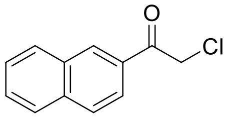

2-Chloro-1-(naphthalen-2-yl)ethanone (2t):[10] White solid (47% isolated yield). R_f (Hexane/CH_2Cl_2 1:1) 0.51. **^{1}H-NMR** (300 MHz, $CDCl_3$) δ 8.47 (s, 1H), 8.03–7.88 (m, 4H), 7.67–7.56 (m, 2H), 4.85 (s, 2H). **^{13}C-NMR** (75 MHz, $CDCl_3$) δ 191.2 (C), 136.0 (C), 132.5 (C), 131.7 (C), 130.6 (CH), 129.8 (CH), 129.2 (CH), 129.0 (CH), 128.0 (CH), 127.3 (CH), 124.0 (CH), 46.2 (CH_2).

3.2. Experimental protocol for the synthesis of racemic halohydrins 1a-t

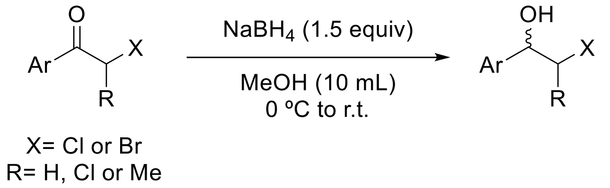

The corresponding α-haloketone **2** (1 equiv, 1 mmol) was dissolved in MeOH (10 mL). Next, sodium borohydride (1.5 equiv) was added in portions at 0 ºC while stirring. The reaction was allowed to reach room temperature and stirred until complete consumption of the starting material observed by TLC analysis (mixtures of Hexane/CH_2Cl_2). The resulting solution was quenched with a few drops of an HCl 6 N aqueous solution and then extracted with EtOAc (2 x 20 mL). The organic phases were combined, dried over Na_2SO_4,

filtered and concentrated under reduced pressure, obtaining the racemic halohydrins **1** which could be used without further purification:[4,6,11,12]

- For compound **1r**, just 1 equiv of sodium borohydride was used to minimise the formation of the corresponding epoxide as side-reaction product.[4]
- For compound **1s**, just 1 equiv of sodium borohydride was used in order to minimise the epoxidation side-reaction and additional THF (10 mL) was added to improve the solubility. Purification by column chromatography on silica gel (Hexane/ CH_2Cl_2 1:1) was required for this compound.
- For compound **1t**, just 1 equiv of sodium borohydride was used in order to minimise the epoxidation side-reaction and purification by column chromatography on silica gel (Hexane/ CH_2Cl_2 1:1) was required.

Spectroscopic data of the obtained compounds agreed with those previously reported.[6,11-18]

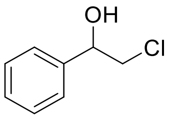

2-Chloro-1-phenylethanol (1a):[6] Colorless oil (98% isolated yield). R_f (Hexane/CH_2Cl_2 8:5) 0.39. **^{1}H-NMR** (300 MHz, $CDCl_3$) δ 7.40–7.30 (m, 5H), 4.90 (dd, *J* = 8.7, 3.5 Hz, 1H), 3.75 (dd, *J* = 11.2, 3.5 Hz, 1H), 3.65 (dd, *J* = 11.2, 8.7 Hz, 1H), 2.77 (br s, 1H). **^{13}C-NMR** (75 MHz, $CDCl_3$) δ 140.0 (C), 128.8 (CH), 128.6 (2CH), 126.2 (2CH), 74.2 (CH), 51.0 (CH_2).

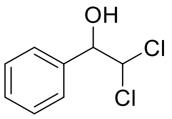

2,2-Dichloro-1-phenylethanol (1b):[11] Colorless oil (94% isolated yield). R_f (Hexane/CH_2Cl_2 8:5) 0.45. **^{1}H-NMR** (300 MHz, $CDCl_3$) δ 7.44–7.38 (m, 5H), 5.83 (dd, *J* = 5.5, 2.0 Hz, 1H), 4.99 (dd, *J* = 5.4, 3.6 Hz, 1H), 2.92 (t, *J* = 3.0 Hz, 1H). **^{13}C-NMR** (75 MHz, $CDCl_3$) δ 137.4 (C), 129.2 (CH), 128.6 (2CH), 127.2 (2CH), 78.9 (CH), 76.5 (CH).

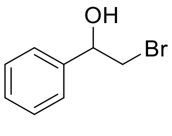

2-Bromo-1-phenylethanol (1c):[6] Colorless oil (75% isolated yield). R_f (Hexane/CH_2Cl_2 8:5) 0.35. **^{1}H-NMR** (300 MHz, $CDCl_3$) δ 7.40–7.30 (m, 5H), 4.93 (dd, *J* = 8.9, 3.4 Hz, 1H), 3.64 (dd, *J* = 10.5, 3.4 Hz, 1H), 3.55 (dd, *J* = 10.5, 8.9 Hz, 1H), 2.72 (br s, 1H). **^{13}C-NMR** (75 MHz, $CDCl_3$) δ 140.4 (C), 128.8 (2CH), 128.6 (CH), 126.1 (2CH), 73.9 (CH), 40.3 (CH_2).

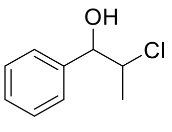

2-Chloro-1-phenylpropan-1-ol (1d):[13] Colorless oil (89% isolated yield). R_f (Hexane/CH_2Cl_2 8:5) 0.28. **^{1}H-NMR** (300 MHz, CDCl3) δ 7.41–7.30 (m, 5H), 4.58 (d, J = 7.6 Hz, 1H), 4.21 (dq, J = 7.7, 6.7 Hz, 1H), 2.93 (s, 1H), 1.37 (d, J = 6.7 Hz, 3H). **^{13}C-NMR** (75 MHz, $CDCl_3$) δ 139.7 (C), 128.6 (2CH), 128.6 (CH), 127.0 (2CH), 79.1 (CH), 64.5 (CH_2), 21.6 (CH_3).

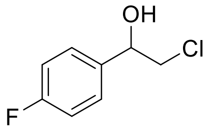

2-Chloro-1-(4-fluorophenyl)ethanol (1e):[6] Colorless oil (95% isolated yield). R_f (Hexane/CH_2Cl_2 8:5) 0.19. **^{1}H-NMR** (300 MHz, $CDCl_3$) δ 7.40–7.33 (m, 2H), 7.10–7.02 (m, 2H), 4.88 (dt, J = 8.8, 2.8 Hz, 1H), 3.71 (dd, J = 11.2, 3.6 Hz, 1H), 3.61 (dd, J = 11.3, 8.6 Hz, 1H), 2.75 (d, J = 2.8 Hz, 1H). **^{13}C-NMR** (75 MHz, $CDCl_3$) δ 162.8 (d, J = 246.9 Hz, C), 135.8 (d, J = 3.1 Hz, C), 127.9 (d, J = 8.2 Hz, 2CH), 115.7 (d, J = 21.6 Hz, 2CH), 73.5 (CH), 50.9 (CH_2). **^{19}F{^{1}H}-NMR** (282 MHz, $CDCl_3$) δ –113.5.

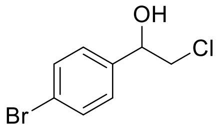

1-(4-Bromophenyl)-2-chloroethanol (1f):[6] Colorless oil (85% isolated yield). R_f (Hexane/CH_2Cl_2 8:5) 0.13. **^{1}H-NMR** (300 MHz, $CDCl_3$) δ 7.51 (d, J = 8.5 Hz, 2H), 7.27 (d, J = 8.3, 2H), 4.87 (dt, J = 8.6, 3.3 Hz, 1H), 3.72 (dd, J = 11.2, 3.5 Hz, 1H), 3.60 (dd, J = 11.3, 8.6 Hz, 1H), 2.71 (d, J = 3.2 Hz, 1H). **^{13}C-NMR** (75 MHz, $CDCl_3$) δ 139.0 (C), 131.9 (2CH), 127.9 (2CH), 122.5 (C), 73.5 (CH), 50.8 (CH_2).

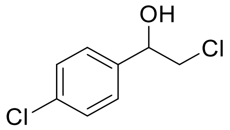

2-Chloro-1-(4-chlorophenyl)ethanol (1g):[6] Colorless oil (87% isolated yield). R_f (Hexane/CH_2Cl_2 8:5) 0.12. **^{1}H-NMR** (300 MHz, $CDCl_3$) δ 7.38–7.32 (m, 4H), 4.89 (dt, J = 8.6, 3.3 Hz, 1H), 3.72 (dd, J = 11.3, 3.5 Hz, 1H), 3.60 (dd, J = 11.3, 8.6 Hz, 1H), 2.69 (d, J = 3.2 Hz, 1H). **^{13}C-NMR** (75 MHz, $CDCl_3$) δ 138.4 (C), 134.4 (C), 129.0 (2CH), 127.6 (2CH), 73.5 (CH), 50.9 (CH_2).

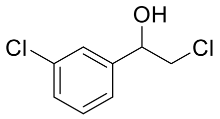

2-Chloro-1-(3-chlorophenyl)ethanol (1h):[6] Colorless oil (83% isolated yield). R_f (Hexane/CH_2Cl_2 8:5) 0.13. **^{1}H-NMR** (300 MHz, $CDCl_3$) δ 7.41–7.40 (m, 1H), 7.32–7.24 (m, 3H), 4.88 (dt, J = 8.7, 3.2 Hz, 1H), 3.74 (dd, J = 11.3, 3.4 Hz, 1H), 3.61 (dd, J = 11.3, 8.6 Hz, 1H), 2.73 (d, J = 3.2 Hz, 1H). **^{13}C-NMR** (75 MHz, $CDCl_3$) δ 142.0 (C), 134.8 (C), 130.1 (CH), 128.7 (CH), 126.4 (CH), 124.4 (CH), 73.5 (CH), 50.8 (CH_2).

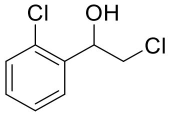

2-Chloro-1-(2-chlorophenyl)ethanol (1i):[6] Colorless oil (84% isolated yield). R_f (Hexane/CH_2Cl_2 8:5) 0.22. **^{1}H-NMR** (300 MHz, $CDCl_3$) δ 7.62 (dd, J = 7.6, 1.9 Hz, 1H), 7.37–7.23 (m, 3H), 5.30 (dd, J = 8.6, 2.8 Hz, 1H), 3.89 (dd, J = 11.3, 2.8 Hz, 1H), 3.55 (dd, J = 11.3, 8.6 Hz, 1H), 2.96 (s, 1H). **^{13}C-NMR** (75 MHz, $CDCl_3$) δ 137.3 (C), 132.0 (C), 129.6 (CH), 129.5 (CH), 127.6 (CH), 127.3 (CH), 70.8 (CH), 49.5 (CH_2).

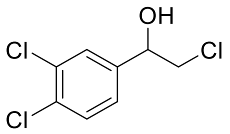

2-Chloro-1-(3,4-dichlorophenyl)ethanol (1j):[6] White solid (89% isolated yield. R_f (Hexane/CH_2Cl_2 8:5) 0.14. **^{1}H-NMR** (300 MHz, $CDCl_3$) δ 7.49 (d, J = 2.1 Hz, 1H), 7.43 (d, J = 8.3 Hz, 1H), 7.20 (dd, J = 8.3, 2.0 Hz, 1H), 4.85 (dd, J = 8.5, 3.5 Hz, 1H), 3.71 (dd, J = 11.3, 3.5 Hz, 1H), 3.58 (dd, J = 11.3, 8.5 Hz, 1H), 2.95 (s, 1H). **^{13}C-NMR** (75 MHz, $CDCl_3$) δ 140.1 (C), 133.0 (C), 132.5 (C), 130.7 (CH), 128.3 (CH), 125.5 (CH), 72.9 (CH), 50.5 (CH_2).

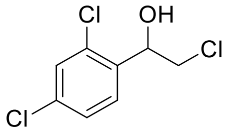

2-Chloro-1-(2,4-dichlorophenyl)ethanol (1k):[12] White solid (91% isolated yield). R_f (Hexane/CH_2Cl_2 8:5) 0.26. **^{1}H-NMR** (300 MHz, $CDCl_3$) δ 7.56 (d, J = 8.4 Hz, 1H), 7.37 (d, J = 2.0 Hz, 1H), 7.30 (dd, J = 8.4, 2.0 Hz, 1H), 5.25 (dt, J = 8.6, 2.8 Hz, 1H), 3.86 (dd, J = 11.3, 2.8 Hz, 1H), 3.51 (dd, J = 11.3, 8.4 Hz, 1H), 2.92 (d, J = 3.3 Hz, 1H). **^{13}C-NMR** (75 MHz, $CDCl_3$) δ 136.0 (C), 134.7 (C), 132.6 (C), 129.4 (CH), 128.6 (CH), 127.7 (CH), 70.4 (CH), 49.2 (CH_2).

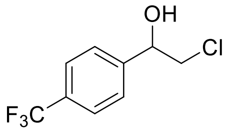

2-Chloro-1-(4-(trifluoromethyl)phenyl)ethanol (1l):[14] White solid (93% isolated yield). R_f (Hexane/CH_2Cl_2 8:5) 0.18. **^{1}H-NMR** (300 MHz, $CDCl_3$) δ 7.64 (d, J = 8.2 Hz, 2H), 7.52 (d, J = 8.1 Hz, 2H), 4.97 (dt, J = 8.4, 3.1 Hz, 1H), 3.76 (dd, J = 11.3, 3.4 Hz, 1H), 3.63 (dd, J = 11.3, 8.5 Hz, 1H), 2.82 (d, J = 3.2 Hz, 1H). **^{13}C-NMR** (75 MHz, $CDCl_3$) δ 143.9 (C), 130.8 (q, J = 32.5 Hz, CF_3), 126.6 (2CH), 125.8 (q, J = 3.7 Hz, 2CH), 122.3 (C), 73.5 (CH), 50.7 (CH_2). **^{19}F{^{1}H}-NMR** (282 MHz, $CDCl_3$) δ –62.6.

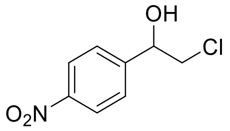

2-Chloro-1-(4-nitrophenyl)ethanol (1m):[14] Yellowish oil (85% isolated yield). R_f (Hexane/EtOAc/CH_2Cl_2 16:5:10) 0.31. **^{1}H-NMR** (300 MHz, $CDCl_3$) δ 8.22 (d, J = 8.6 Hz, 2H), 7.58 (d, J = 8.5 Hz, 2H), 5.02 (d, J = 7.2 Hz, 1H), 3.78 (dd, J = 11.4, 3.6 Hz, 1H), 3.64 (dd, J = 11.4, 8.1 Hz, 1H), 2.97 (s, 1H). **^{13}C-NMR** (75 MHz, $CDCl_3$) δ 147.9 (C), 147.1 (C), 127.1 (2CH), 123.9 (2CH), 73.1 (CH), 50.4 (CH_2).

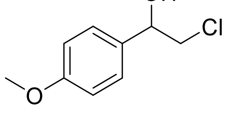

2-Chloro-1-(4-methoxyphenyl)ethanol (1n):[6] Colorless oil (97% isolated yield). R_f (Hexane/CH_2Cl_2 8:5) 0.28. **^{1}H-NMR** (300 MHz, $CDCl_3$) δ 7.33–7.29 (m, 2H), 6.93–6.88 (m, 2H), 4.84 (dd, J = 8.6, 3.7 Hz, 1H), 3.81 (s, 3H), 3.70 (dd, J = 11.2, 3.8 Hz, 1H), 3.62 (dd, J = 11.2, 8.6 Hz, 1H), 2.72 (s, 1H). **^{13}C-NMR** (75 MHz, $CDCl_3$) δ 159.8 (C), 132.2 (C), 127.4 (2CH), 114.1 (2CH), 73.8 (CH), 55.4 (CH_3), 51.0 (CH_2).

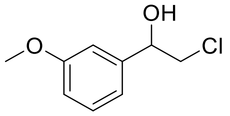

2-Chloro-1-(3-methoxyphenyl)ethanol (1o):[6] Colorless oil (80% isolated yield). R_f (Hexane/CH_2Cl_2 8:5) 0.15. **^{1}H-NMR** (300 MHz, $CDCl_3$) δ 7.29 (t, J = 8.2 Hz, 1H), 6.96-6.94 (m, 2H), 6.87 (ddd, J = 8.3, 2.6, 1.1 Hz, 1H), 4.89 (dd, J = 8.8, 3.4 Hz, 1H), 3.82 (s, 3H), 3.75 (dd, J = 11.2, 3.4 Hz, 1H), 3.64 (dd, J = 11.2, 8.8 Hz, 1H), 2.67 (br s, 1H). **^{13}C-NMR** (75 MHz, $CDCl_3$) δ 160.0 (C), 141.7 (C), 129.9 (CH), 118.4 (CH), 114.1 (CH), 111.6 (CH), 74.1 (CH), 55.4 (CH_3), 51.0 (CH_2).

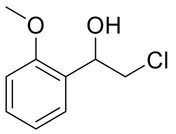

2-Chloro-1-(2-methoxyphenyl)ethanol (1p):[15] Colorless oil (78% isolated yield). R_f (Hexane/CH_2Cl_2 8:5) 0.05. **^{1}H-NMR** (300 MHz, $CDCl_3$) δ 7.44 (dd, J = 7.4, 1.7 Hz, 1H), 7.30 (td, J = 8.2, 1.6 Hz, 1H), 7.00 (td, J = 7.5, 1.1 Hz, 1H), 6.89 (dd, J = 8.2, 1.0 Hz, 1H), 5.14 (d, J = 9.0 Hz, 1H), 3.88-3.83 (m, 4H), 3.64 (dd, J = 11.0, 8.3 Hz, 1H), 3.03 (br s, 1H). **^{13}C-NMR** (75 MHz, $CDCl_3$) δ 156.3 (C), 129.4 (CH), 127.9 (C), 127.3 (CH), 121.0 (CH), 110.5 (CH), 70.5 (CH), 55.4 (CH_3), 49.7 (CH_2).

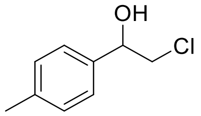

2-Chloro-1-(*p*-tolyl)ethanol (1q):[6] White solid (94% isolated yield). R_f (Hexane/CH_2Cl_2 4:1) 0.01. **^{1}H-NMR** (300 MHz, $CDCl_3$) δ 7.27 (d, J = 8.1 Hz, 2H), 7.19 (d, J = 7.9 Hz, 2H), 4.86 (dd, J = 8.7, 3.6 Hz, 1H), 3.72 (dd, J = 11.3, 3.6 Hz, 1H), 3.63 (dd, J = 11.2, 8.7 Hz, 1H), 2.64 (br s, 1H), 2.36 (s, 3H). **^{13}C-NMR** (75 MHz, $CDCl_3$) δ 138.4 (C), 137.1 (C), 129.5 (2CH), 126.1 (2CH), 74.1 (CH), 51.0 (CH_2), 21.3 (CH_3).

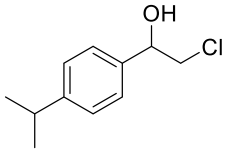

2-Chloro-1-(4-isopropylphenyl)ethanol (1r):[16] Colorless oil (93% isolated yield). R_f (Hexane/EtOAc/CH_2Cl_2 8:1:5) 0.52. **^{1}H-NMR** (300 MHz, $CDCl_3$) δ 7.32 (d, J = 8.3 Hz, 2H), 7.25 (d, J = 8.3 Hz, 2H), 4.87 (dd, J = 8.7, 3.6 Hz, 1H), 3.74 (dd, J = 11.2, 3.6 Hz, 1H), 3.65 (dd, J = 11.2, 8.7 Hz, 1H), 2.92 (hept, J = 6.9 Hz, 1H), 1.26 (d, J = 6.9 Hz, 6H). **^{13}C-NMR** (75 MHz, $CDCl_3$) δ 149.4 (C), 137.4 (C), 126.9 (2CH), 126.2 (2CH), 74.1 (CH), 51.0 (CH_2), 34.0 (CH), 24.1 (2CH_3).

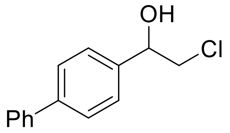

1-[(1,1'-Biphenyl)-4-yl]-2-chloroethanol (1s):[17] Colorless oil (72% isolated yield). R_f (Hexane/CH_2Cl_2 1:1) 0.26. **^{1}H-NMR** (300 MHz, $CDCl_3$) δ 7.64-7.58 (m, 4H), 7.49-7.43 (m, 4H), 7.40-7.35 (m, 1H), 4.96 (dd, J = 8.7, 3.5 Hz, 1H), 3.80 (dd, J = 11.2, 3.5 Hz, 1H), 3.70 (dd, J = 11.2, 8.7 Hz, 1H), 2.80 (br s, 1H). **^{13}C-NMR** (75 MHz, $CDCl_3$) δ 141.5 (C), 140.7 (C), 139.0 (C), 128.9 (2CH), 127.6 (CH), 127.5 (2CH), 127.2 (2CH), 126.6 (2CH), 74.0 (CH), 51.0 (CH_2).

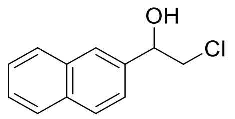

2-Chloro-1-(naphthalen-2-yl)ethanol (1t):[18] Brownish solid (98% isolated yield). R_f (Hexane/CH_2Cl_2 1:1) 0.29. **^{1}H-NMR** (300 MHz, $CDCl_3$) δ 7.88–7.82 (m, 4H), 7.54–7.46 (m, 3H), 5.08 (dd, J = 8.7, 3.5 Hz, 1H), 3.84 (dd, J = 11.3, 3.5 Hz, 1H), 3.73 (dd, J = 11.3, 8.7 Hz, 1H), 2.80 (br s, 1H). **^{13}C-NMR** (75 MHz, $CDCl_3$) δ 137.3 (C), 133.4 (C), 133.3 (C), 128.7 (CH), 128.2 (CH), 127.9 (CH), 126.5 (CH), 126.4 (CH), 125.4 (CH), 123.8 (CH), 74.3 (CH), 51.0 (CH_2).

4. Photo-oxidation of halohydrins

4.1. Description of the photochemistry setups

The photochemistry setup is composed by an irradiation source, a cooling fan, a magnetic stirrer and a vial-holder. The irradiation source consisted of either:

a) Common white LED (Light Emitting Diode) strip lights in a box (Figure S5). To a circular plastic box lined with aluminum foil, strip lights are attached to the walls using zip ties forming a 4 cm high coil.

b) Two Kessil® blue LED lamps in a box (Figure S6). The Kessil® lamps used are: H150W-blue (set at maximum light intensity) and H160 Tuna Flora (set at maximum light intensity and blue spectra). The box is lined with aluminum foil and the lamps are introduced throw two opposite holes.

Each of the described setups is covered to avoid external light contamination. Photochemical reactions are performed in 4 or 25-mL glass vials with screw cap and a magnetic stirrer. The vials are placed at a distance of 3 cm away from the white LED strip lights and 4.5 cm away from the blue LED lamps. 4 mL vials are held with a 3D-printed plastic holder whereas 24 mL vials are held with a clamp.

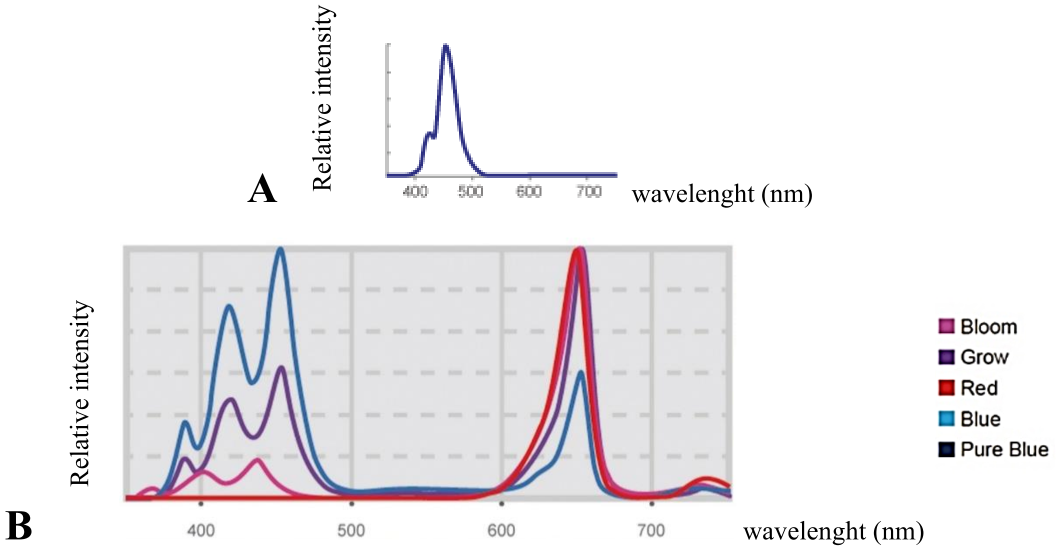

Figure S4. Emission spectrum of Kessil® H150W-blue (A) and Kessil® H160 Tuna Flora (B). The images were taken from the manufacturer's website (*Kessil.com*).

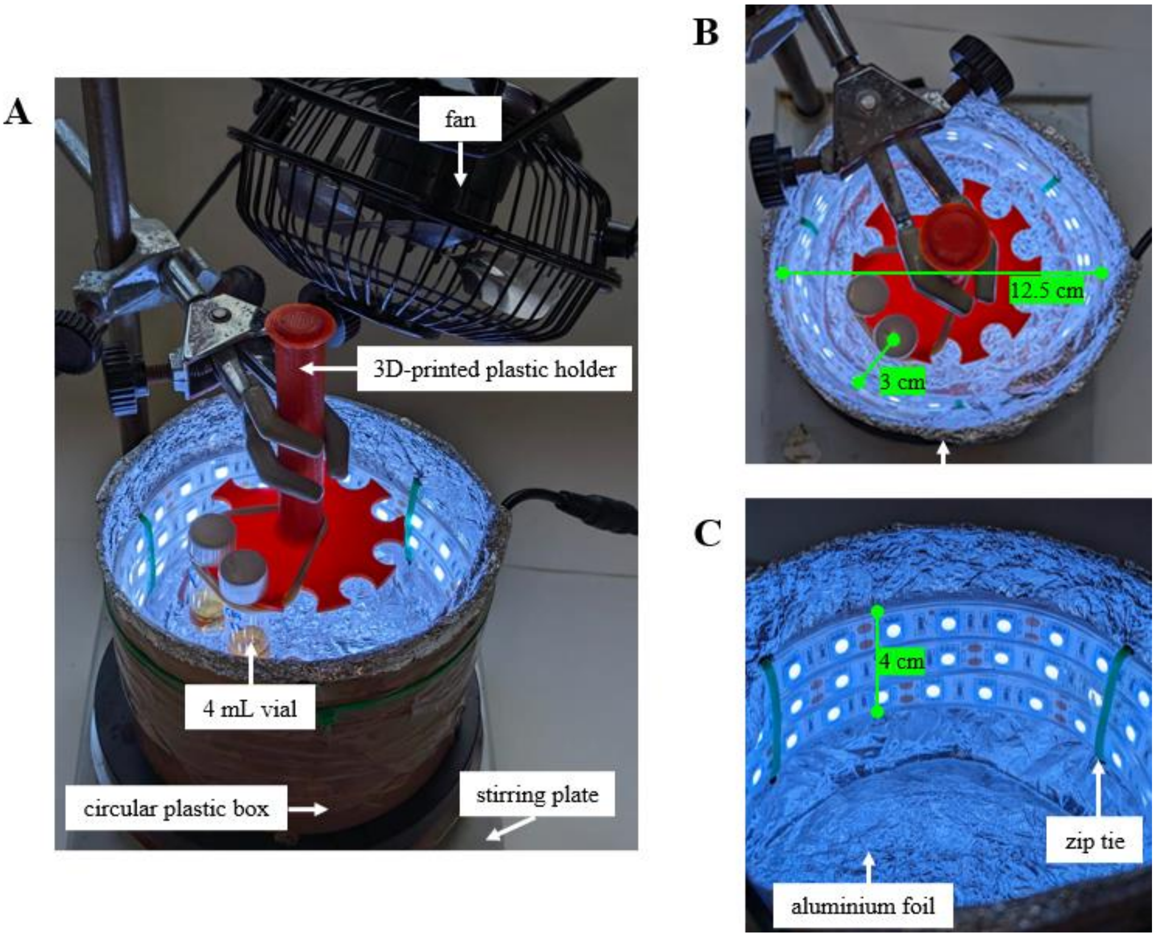

Figure S5. Photochemistry setup using white LED strip lights in a box. **A**) Front view. **B**) Top view. **C**) Interior view.

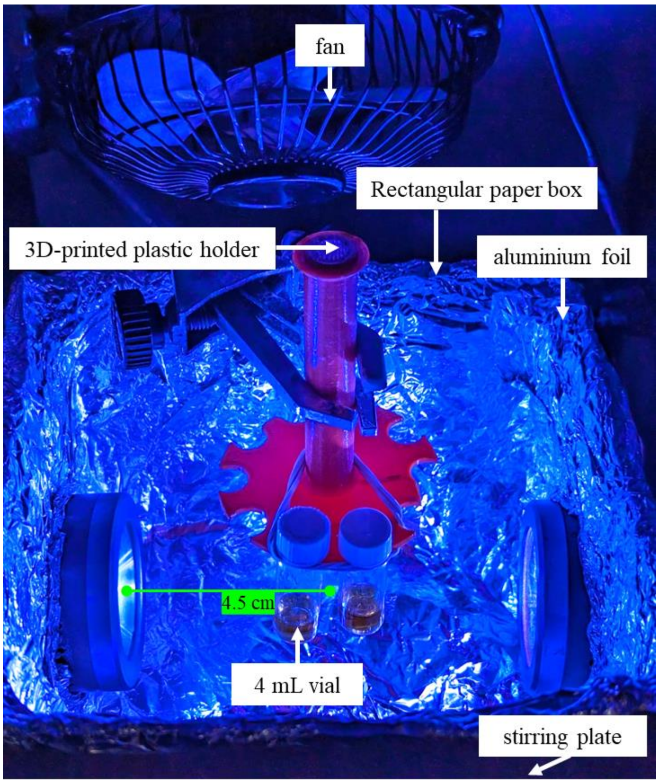

Figure S6. Photochemistry setup using two Kessil® lamps.

Ensuring the reproducibility of photochemical set-ups is crucial for obtaining consistent and reliable results. To achieve this, equal reactions were conducted at various positions within both setups (Scheme S1). The study was performed using 2-chloro-1-phenylethanol (**1a**) as model substrate. The findings revealed uniform results across all positions when using white LED strip lights. However, variations were observed in reactions conducted under the two blue LED lamps setup, with the Kessil® H150W-blue lamp yielding better conversions compared to the Kessil® H160 Tuna Flora lamp.

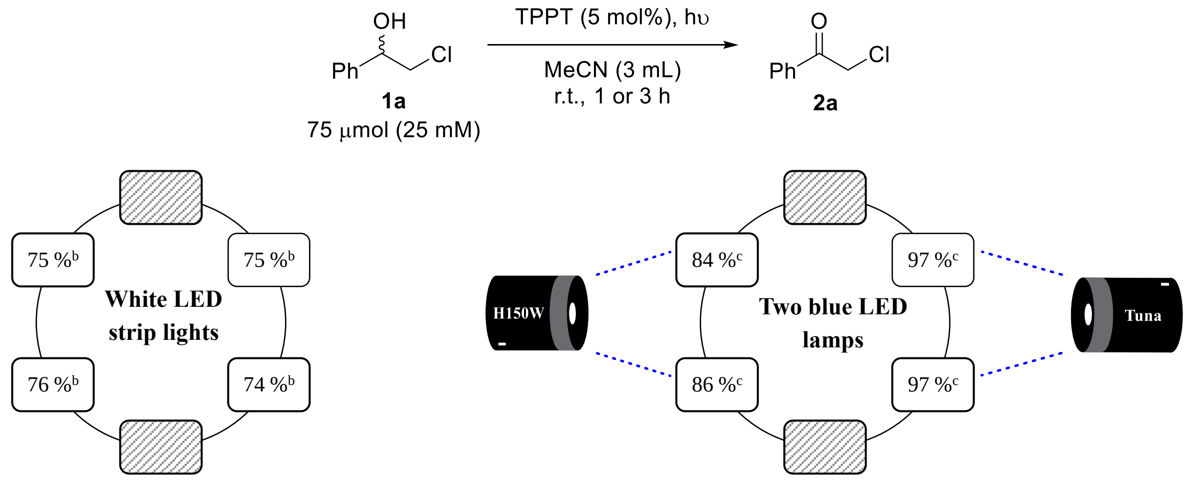

Conversion values were calculated by GC analysis. [b] 1 h reaction time. [c] 3 h reaction time.

Scheme S1. Reproducibility testing of the photochemical setups.

4.2. Optimizing the photo-oxidation reaction

Up to now, only two photobiocatalytic deracemizations of *sec*-alcohols have been reported, using 9-fluorenone or sodium anthraquinone 2-sulfonate (SAS) as photocatalysts.[19,20] However, the photochemical conditions in those works lack the capability to efficiently oxidize β-chlorohydrins because this transformation is thermodynamically disfavoured. Therefore, we propose a novel one-pot two-step methodology capable of efficiently deracemizing β-chlorohydrins.

In order to find a suitable photo-oxidizer, a screening was performed using 2-chloro-1-phenylethanol (**1a**) as model substrate (Table S1). Commonly used single-electron transfer (SET) photocatalysts were used: **9-fluorenone**, 9-mesityl-10-methylacridinium perchlorate (**[Mes-Acr-Me]ClO$_4$**), sodium anthraquinone 2-sulfonate (**SAS**), 2,4,6-triphenylpyrylium tetrafluoroborate (**TPPT**) and 2,3-dichloro-5,6-dicyano-1,4-benzoquinone (**DDQ**). Thus, the potential window studied ranged from +0.96 to +3.18 V versus saturated calomel electrode (SCE), contemplating the excited triplet state reduction potentials ($E_{red}^{T_1}$) of those phtosensitizers.[21,22]

Oxygen is able to regenerate photocatalysts such 9-fluorenone, [Mes-Acr-Me]ClO$_4$, SAS and TPPT. However, DDQ cannot be regenerated by oxygen and requires more specific regeneration systems. See Section 4.6 for mechanistic details.

Also, tetrabutylammonium decatungstate (**TBADT**) was tested, which is regenerated by oxygen. This photosensitizer can undergo via a single electron transfer (SET) mechanism and also hydrogen atom transfer (HAT), depending on the redox properties of the subtract.[23]

Table S1. Screening of photosensitizers and irradiation source in the oxidation reaction of halohydrin **1a** to haloketone **2a** in MeCN as solvent.

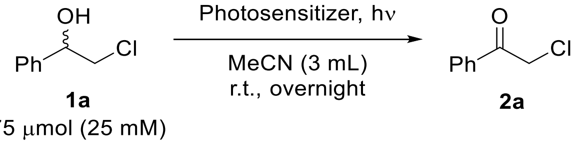

1a 75 μmol (25 mM) **2a**

Entry	Photosensitizer	$E_{red}^{T_1}$ (V vs SCE)	Irradiation	Atmosphere	2a (%)[a]
1	9-Fluorenone (20 mol%)	+0.96	Blue	O_2 (1 atm)[b]	3
2	[Mes-Acr-Me]ClO_4 (20 mol%)	+1.45	Blue	O_2 (1 atm)[b]	9
3	SAS (20 mol%)	+1.80	Blue	O_2 (1 atm)[b]	65
4	TPPT (20 mol%)	+2.02	Blue	O_2 (1 atm)[b]	>99
5			White	O_2 (1 atm)[b]	>99
6			White	Air	>99
7	DDQ (1.1 equiv)	+3.18	Blue	Air	>99
8			White	Air	>99
9[c]	TBADT (30 mol%)	+ 2.44	390 nm	O_2 (1 atm)[b]	25

[a] Conversion values were calculated by GC analysis.

[b] The reaction was pre-bubbled with O_2 for 5 min and a balloon filled with O_2 was connected to the reaction vial with a syringe adaptor.

[c] After the reaction time, the following product distribution was observed: **2a** (25%), **1a** (25%) and acetophenone (**3a**, 50%).

4.2.1. Method A: using TPPT in catalytic amount

Based on the preliminary results, the photo-oxidation reaction of **1a** using catalytic amounts of TPPT was optimised using **1a** as model substrate in terms of catalyst loading, reaction time and substrate concentrations (Tables S2 and S3).

Table S2. Optimisation of the catalytic amount of TPPT and the reaction time in the photo-oxidation reaction of halohydrin **1a** to haloketone **2a**.

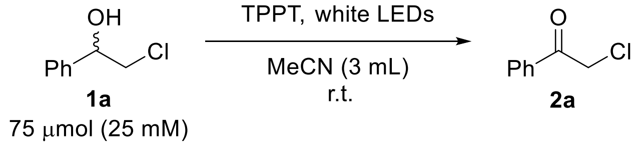

		2a (%)[a]			
**Entry**	**t (h)**	**5 mol% TPPT**	**7.5 mol% TPPT**	**10 mol% TPPT**	**15 mol% TPPT**
1	0.5	58	71	77	83
2	1	79	86	89	90
3	2	85	92	99	99
4	3	91	99	>99	>99
5	4	n.p.	>99	n.p.	n.p.

[a] Conversion values were calculated by GC analysis. n.p.: Not performed.

Table S3. Optimization of the volume of organic solvent (MeCN) and revaluation of the reaction time in the photo-oxidation reaction of halohydrin **1a** to haloketone **2a**.

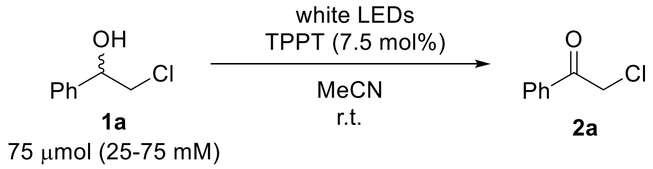

Entry	MeCN (mL)	time (h)	2a (%)[a]
1	3 (25 mM)	4	>99
2	2 (50 mM)	4	>99
3	1 (75 mM)	4	>99
4	1 (75 mM)	1	>99

[a] Conversion values were calculated by GC analysis.

4.2.2. Method B: using DDQ in stoichiometric amount

Based on the preliminary results, the photo-oxidation reaction with stoichiometric amounts of DDQ was optimized using **1a** as model substrate (Table S4).

Table S4. Optimization of the stoichiometric amount of DDQ and the reaction time in the photo-oxidation reaction of halohydrin **1a** to haloketone **2a**.

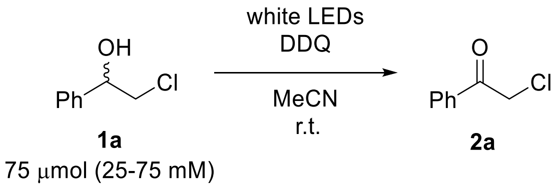

Entry	MeCN (mL)	DDQ (equiv)	Time	2a (%)[a]
1	3 (25 mM)	0.25	Overnight	51
2	3 (25 mM)	0.5	Overnight	72
3	3 (25 mM)	0.8	Overnight	82
4	3 (25 mM)	1	Overnight	>99
5	3 (25 mM)	1.2	Overnight	>99
6	3 (25 mM)	1.2	8 h	>99
7	3 (25 mM)	1.2	4 h	>99
8	1 (75 mM)	1.2	4 h	>99
9	1 (75 mM)	1	4 h	>99
10	1 (75 mM)	1	1 h	78

[a] Conversion values were calculated by GC analysis.

4.2.3. Method C: using DDQ in catalytic amount

Based on the preliminary results, the photo-oxidation reaction with catalytic amounts of DDQ was optimized using **1a** as model substrate (Tables S5 to S7). Therefore, a screening of regeneration systems for DDQ was performed using reported oxidizing agents such as *tert*-butyl nitrite (TBN), manganese dioxide (MnO_2) and nitric acid (HNO_3).[24]

Table S5. Study the use of the HNO_3/O_2 system for the regeneration for DDQ in the in the photo-oxidation reaction of halohydrin **1a** to haloketone **2a**.

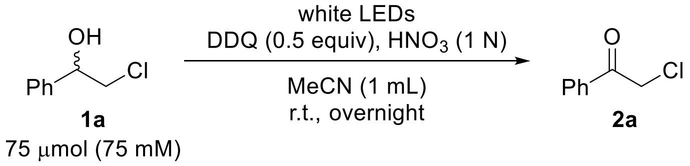

Entry	HNO_3 (equiv)	2a (%)[a]
1	-	72
2	0.27	26
3	0.53	14
4	0.80	5

[a] Conversion values were calculated by GC analysis.

Table S6. Study the use of the MnO_2/O_2 system for the regeneration of DDQ in the in the photo-oxidation reaction of alcohol **1a** to ketone **2a**.

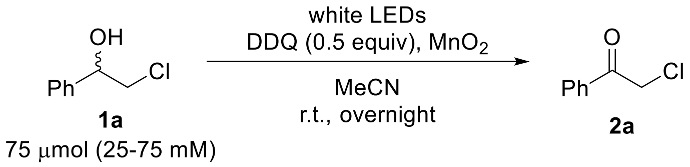

Entry	MnO_2 (equiv)	MeCN (mL)	2a (%)[a]
1	-	3 (25 mM)	72
2	0.5	3 (25 mM)	60
3	1	3 (25 mM)	62
4	3	1 (75 mM)	51
5	1	1 (75 mM)	50

[a] Conversion values were calculated by GC analysis.

Table S7. Study the use of the TBN/ O_2 system for the regeneration of DDQ in the photo-oxidation reaction of alcohol **1a** to ketone **2a**.

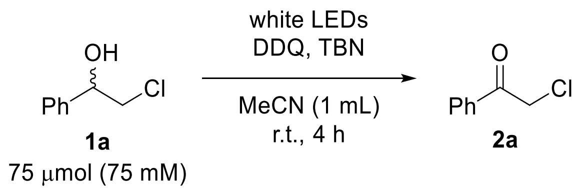

Entry	DDQ (mol%)	TBN (equiv)	2a (%)[a]
1	50	-	43
2	-	1.5	8
3	-	20	9
4	50	1.5	>99
5	40	1.5	>99
6	30	1.5	>99
7	20	1.5	>99
8	10	1.5	>99
9	5	1.5	>99
10	3	1.5	89
11	2	1.5	82
12	5	1	>99
13	5	0.5	>99

[a] Conversion values were calculated by GC analysis.

4.3. Substrate scope of the photo-oxidation reaction

The applicability of the photo-oxidation conditions optimized in Section 4.2 was studied with 19 additional substrates (alcohols **1a-t**, Table S8). Regarding **method A**, using TPPT as photocatalyst, the formation of dehalogenation by-products **3** was observed for several substrates, particularly with the α-substituted substrate **1d** (R^2= Me). However, **methods B** and **C**, employing DDQ as photocatalysts, did not yield dehalogenation by-products **3**, except for α-substituted substrate **1d** (R^2= Me).

Table S8. Substrate scope of the photo-oxidation reaction of halohydrins **1a-s** to haloketones **2a-s,** using either TPPT 20 mol% (**method A**), DDQ 1 equiv (**method B**) or DDQ 5 mol% (**method C**).

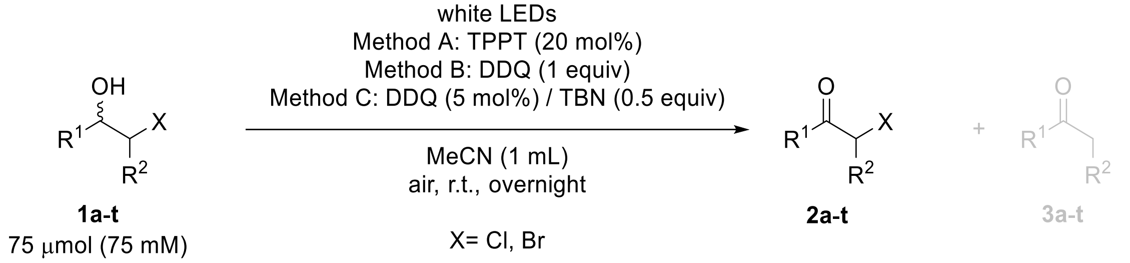

Entry	Product (2a-t)	Method	2a-t (%)[a,b]	3a-t (%)[a]
1[e]		A	**>99**	n.d.
2[f]	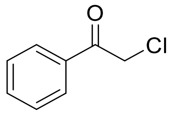	B	**>99**	n.d.
3[f]	**2a**	C	**>99 (98)**	n.d.
4[c]		A	**20**	13
5[c]	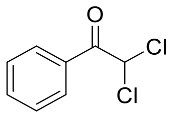	B	**65**	n.d.
6[c]	**2b**	C	**26**	n.d.
7[c]		A	**64**	24
8[c]	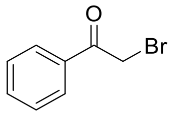	B	**94**	n.d.
9[c]	**2c**	C	**90**	n.d.
10[d]		A	**<3**	84
11[d]	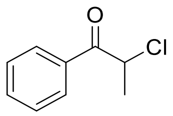	B	**<3**	10
12[d]	**2d**	C	**35**	6
13		A	**43**	57
14	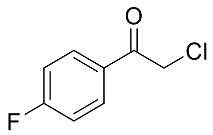	B	**>99**	n.d.
15	**2e**	C	**>99 (95)**	n.d.
16		A	**49**	50
17	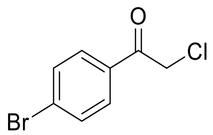	B	**79**	n.d.
18	**2f**	C	**>99 (96)**	n.d.

Entry	Product (2a-t)	Method	2a-t (%)[a,b]	3a-t (%)[a]
19	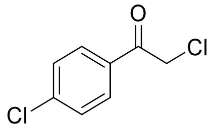	A	**90**	10
20		B	**>99**	n.d.
21	**2g**	C	**>99 (92)**	n.d.
22	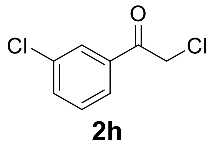	A	**37**	14
23		B	**61**	n.d.
24	**2h**	C	**58**	n.d.
25	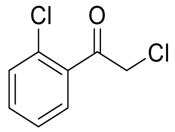	A	**19**	8
26		B	**12**	n.d.
27	**2i**	C	**19**	n.d.
28	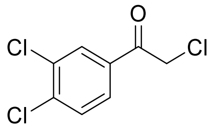	A	**60**	13
29		B	**92**	n.d.
30	**2j**	C	**80**	n.d.
31	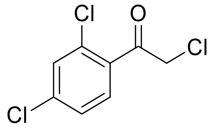	A	**61**	9
32		B	**65**	n.d.
33	**2k**	C	**37**	n.d.
34	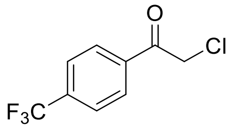	A	**11**	n.d.
35		B	**8**	n.d.
36	**2l**	C	**31**	n.d.
37	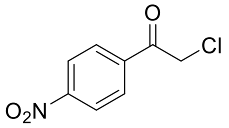	A	**8**	n.d.
38		B	**3**	n.d.
39	**2m**	C	**19**	n.d.
40	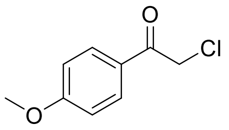	A	**90**	n.d.
41		B	**80**	n.d.
42	**2n**	C	**>99 (97)**	n.d.
43	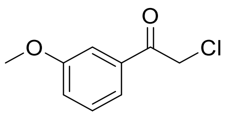	A	**12**	n.d.
44		B	**8**	n.d.
45	**2o**	C	**37**	n.d.

Entry	Product (2a-t)	Method	2a-t (%)[a,b]	3a-t (%)[a]
46	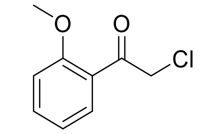	A	**25**	n.d.
47		B	**31**	n.d.
48	**2p**	C	**44**	n.d.
49	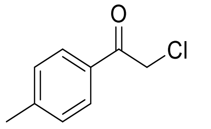	A	**47**	40
50		B	**40**	n.d.
51	**2q**	C	**40**	n.d.
52	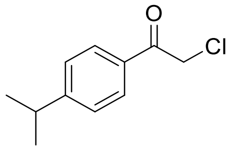	A	**37**	n.d.
53		B	**26**	n.d.
54	**2r**	C	**34**	n.d.
55	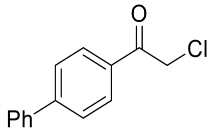	A	**35**	36
56		B	**13**	n.d.
57	**2s**	C	**30**	n.d.
58	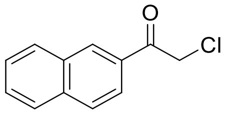	A	**11**	n.d.
59		B	**70**	n.d.
60	**2t**	C	**27**	n.d.

[a] Conversion values were calculated by GC analysis unless otherwise stated in footnotes c and d. [b] Isolated yields appear in parentheses after column chromatography purification. [c] Conversion values were calculated by HPLC analysis. [d] Conversion values were calculated by ^{1}H-NMR analysis of the reaction crudes. [e] 1 h reaction time and TPPT 7.5 mol%. [f] 4 h reaction time. n.d.: Not detected.

To understand the formation of the dehalogenation by-products **3**, 2-chloro-4'-haloacetophenones **2e-g** were incubated in the reaction medium (Table S9). Since acetophenones **3e-g** were not observed after white light irradiation with the presence of TPPT, we speculate that formation of **3e-g** in the photo-oxidation reactions of **1e-g** comes from a resonant structure of the corresponding radical intermediate.

Table S9. Incubation of 2-chloro-4'-haloacetophenones **2e-g** in the reaction media of **method A**.

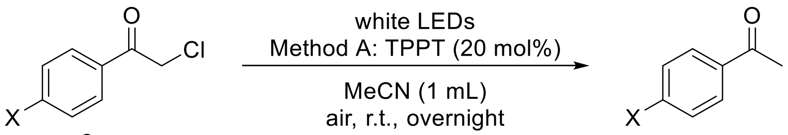

2e-g
75 μmol (75 mM)

3e-g

Entry	X	2e-g (%)[a]	3e-g (%)[a]
1	F (**e**)	>99	<1
2	Cl (**f**)	>99	<1
3	Br (**g**)	>99	<1

[a] Product percentages were calculated by GC analysis.

Table S10. Re-optimization of the photo-oxidation conditions in **method C** to improve the poor reactivity of highly electron-deficient alcohols containing electron-withdrawing groups in the aryl ring. The studies were performed using **1l** as model substrate.

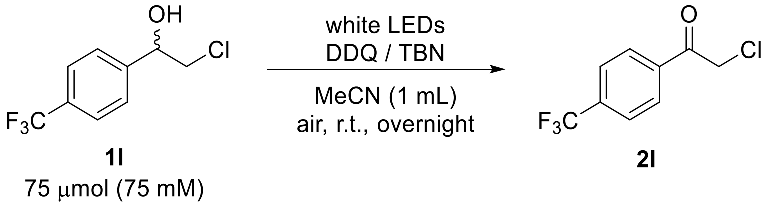

Entry	DDQ (equiv)	TBN (equiv)	Deviation from standard conditions	2l (%)[a]
1	0.05	1.5	-	21
2	0.5	1.5	-	26
3	1	1.5	-	26
4	2	1.5	-	31
5	2	-	-	30
6	0.5	3	-	25
7	0.5	30	-	29
8	0.5	1.5	30 h reaction time	30
9	0.5	1.5	CH_2Cl_2 instead of MeCN as solvent	36
10	0.5	1.5	O_2 (1 atm) instead of air atmosphere	31

[a] Conversion values were calculated by GC analysis.

Table S11. Re-optimization of the photo-oxidation conditions in **method C** for the conversion of halohydrin **1h** to haloketone **2h.**

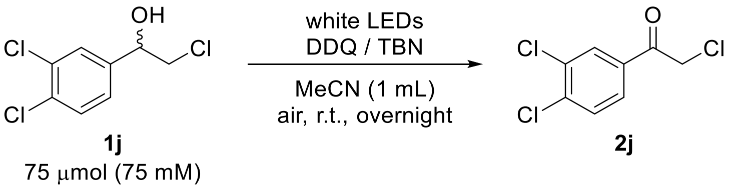

Entry	DDQ (equiv)	TBN (equiv)	2h (%)[a,b]
1	2	-	>99
2	0.5	1.5	>99 (95)
3	1	1.5	>99

[a] Conversion values were calculated by GC analysis.

[b] Yield reported in brackets refer to isolated and purified product **2h**.

Finally, **method C**, employing DDQ (5 mol%) and TBN (0.5 equiv), was selected as optimum for this transformation achieving 5 ketones (**2a**,**e-g**,**n**) with complete conversion. An alternative set of conditions

employing DDQ (0.5 mol%) and TBN (1.5 equiv) also yielded **2h** with complete conversion. Thus, these 6 substrates were selected for the photo-biocatalytic deracemization studies.

4.4. Experimental protocol for the photo-oxidation of halohydrins 1a-t

In a 4 mL vial, the β-halohydrin **1a-t** (75 µmol) was dissolved in MeCN (1 mL, 75 mM). The vial was closed with a screw cap, and then one of these three photo-oxidation methods were carried out:

- TPPT (20 mol%) was added (**method A**).
- DDQ (1.1 equiv) was added (**method B**).
- DDQ (5 mol%) and TBN (0.5 equiv) were added (**method C**).

Reactions were stirred overnight under white light irradiation at room temperature, using the photochemistry set-up described in Section 4.1. Then, the solvent was concentrated under vacuum and the residue was purified by column chromatography on silica gel, leading to the corresponding α-haloketone **2a-t**:

- For compound **1a**, 1 h of reaction time and TPPT (7.5 mol%) were used for **method A** and 4 h of reaction time were used for **methods B** and **C**.

- For compound **1j**, DDQ (50 mol%) and TBN (1.5 equiv) were employed.

Spectroscopic data of the obtained compounds agreed with those previously reported.[6]

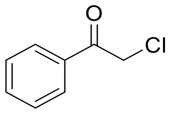

2-Chloro-1-phenylethanone (2a):[6] White solid (98% isolated yield). R_f (Hexane/CH_2Cl_2 8:5) 0.60. **^{1}H-NMR** (300 MHz, $CDCl_3$) δ 7.96–7.92 (m, 2H), 7.61 (td, J = 7.3, 1.8 Hz, 1H), 7.48 (td, J = 7.4, 1.9 Hz, 2H), 4.72 (s, 2H). **^{13}C-NMR** (75 MHz, $CDCl_3$) δ 191.1 (C), 134.3 (C), 134.1 (CH), 129.0 (2CH), 128.6 (2CH), 46.2 (CH_2).

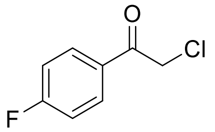

1-(4-Fluorophenyl)-2-chloroethanone (2e):[6] White solid (95% isolated yield). R_f (Hexane/CH_2Cl_2 8:5) 0.41. **^{1}H-NMR** (300 MHz, $CDCl_3$) δ 8.02–7.95 (m, 2H), 7.20–7.12 (m, 2H), 4.67 (s, 2H). **^{13}C-NMR** (75 MHz, $CDCl_3$) δ 189.7 (C), 166.3 (d, J = 256.5 Hz, C), 131.4 (d, J = 9.4 Hz, 2CH), 130.7 (d, J = 3.1 Hz, C), 116.2 (d, J = 22.0 Hz, 2CH), 45.8 (CH_2). **^{19}F-NMR** (282 MHz, $CDCl_3$) δ –103.1.

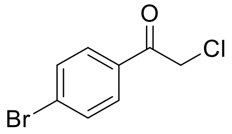

1-(4-Bromophenyl)-2-chloroethanone (2f):[6] White solid (96% isolated yield). R_f (Hexane/CH_2Cl_2 8:5) 0.37. **^{1}H-NMR** (300 MHz, $CDCl_3$) δ 7.85–7.80 (m, 2H), 7.67–7.62 (m, 2H), 4.66 (s, 2H). **^{13}C-NMR** (75 MHz, $CDCl_3$) δ 190.4 (C), 133.0 (C), 132.4 (2CH), 130.2 (2CH), 129.5 (C), 45.8 (CH_2).

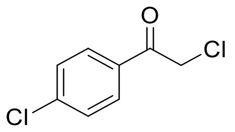

2-Chloro-1-(4-chlorophenyl)ethanone (2g):[6] White solid (92% isolated yield). R_f (Hexane/CH_2Cl_2 8:5) 0.34. **^{1}H-NMR** (300 MHz, $CDCl_3$) δ 7.93–7.88 (m, 2H), 7.50–7.45 (m, 2H), 4.66 (s, 2H). **^{13}C-NMR** (75 MHz, $CDCl_3$) δ 190.2 (C), 140.7 (C), 132.6 (C), 130.1 (2CH), 129.4 (2CH), 45.8 (CH_2).

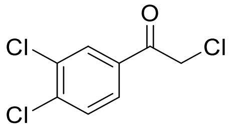

2-Chloro-1-(3,4-dichlorophenyl)ethanone (2j):[6] White solid (95% isolated yield). R_f (Hexane/CH_2Cl_2 8:5) 0.34. **^{1}H-NMR** (300 MHz, $CDCl_3$) δ 8.04 (d, J = 2.1 Hz, 1H), 7.78 (dd, J = 8.4, 2.1 Hz, 1H), 7.59 (d, J = 8.4 Hz, 1H), 4.64 (s, 2H). **^{13}C-NMR** (75 MHz, Chloroform-*d*) δ 189.3 (C), 138.9 (C), 133.9 (C), 133.7 (C), 131.2 (CH), 130.7 (CH), 127.7 (CH), 45.6 (CH_2).

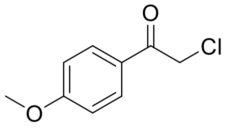

2-Chloro-1-(4-methoxyphenyl)ethan-1-one (2n):[6] White solid (97% isolated yield). R_f (Hexane/CH_2Cl_2 8:5) 0.47. **^{1}H-NMR** (300 MHz, $CDCl_3$) δ 7.93 (d, J = 8.9 Hz, 2H), 6.95 (d, J = 8.9 Hz, 2H), 4.65 (s, 2H), 3.87 (s, 3H). **^{13}C-NMR** (75 MHz, $CDCl_3$) δ 190.2 (C), 164.7 (C), 131.5 (2CH), 127.7 (C), 114.6 (2CH), 56.1 (CH_3), 46.3 (CH_2).

4.5. Solar-driven photo-oxidation reaction

The sun is the most sustainable light source available on our planet, therefore the direct use of sunlight for photochemistry is extremely appealing. Thus, we attempted this approach under previously studied oxidative conditions (Table S12) using the set-up described in Figure 3 of the manuscript.

Table S12. Solar-driven photo-oxidation reaction of halohydrin **1a** to haloketone **2a**, using either TPPT 20 mol% (**method A**), DDQ 1 equiv (**method B**) or DDQ 5 mol% (**method C**).

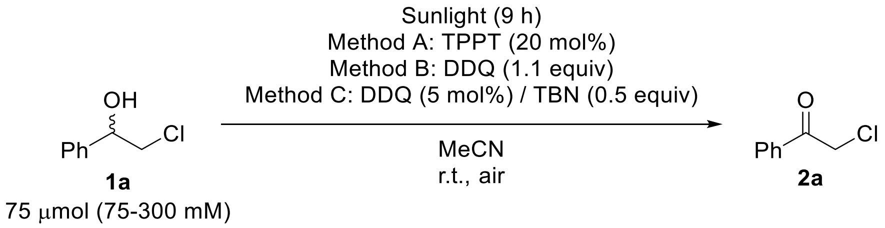

Entry	Method	MeCN (mL)	2a (%)[a]
1	A	1 (75 mM)	>99
2	B	1 (75 mM)	>99
3	C	1 (75 mM)	>99
4	C	0.25 (300 mM)	>99

[a] Conversion values were calculated by GC analysis.

To assess the method's reproducibility, we performed the photo-oxidation reactions on two different dates: 12th October 2023 and 12th March 2024. The reactions were set in the window of our laboratory for 9 h, starting at 09:00 CET.

12th October 2023:

Sunrise/Sunset: 08:25 / 19:54 CET

Total Daylight: Approximately 11.5 h

Average Sunshine Duration: Approximately 5.5 h

UV Index: Maximum of 4

Weather Conditions: Partly cloudy

Temperature: constant laboratory temperature 17 °C.

12th March 2024:

Sunrise/Sunset: 07:40 / 19:26 CET

Total Daylight: Approximately 11.8 h

Average Sunshine Duration: Approximately 7 h

UV Index: Maximum of 3

Weather Conditions: Clear and sunny

Temperature: constant laboratory temperature 17 °C.

4.6. Mechanistic studies

Here below the mechanisms for the photo-oxidation reaction involving both photocatalysts employed in this study are shown:

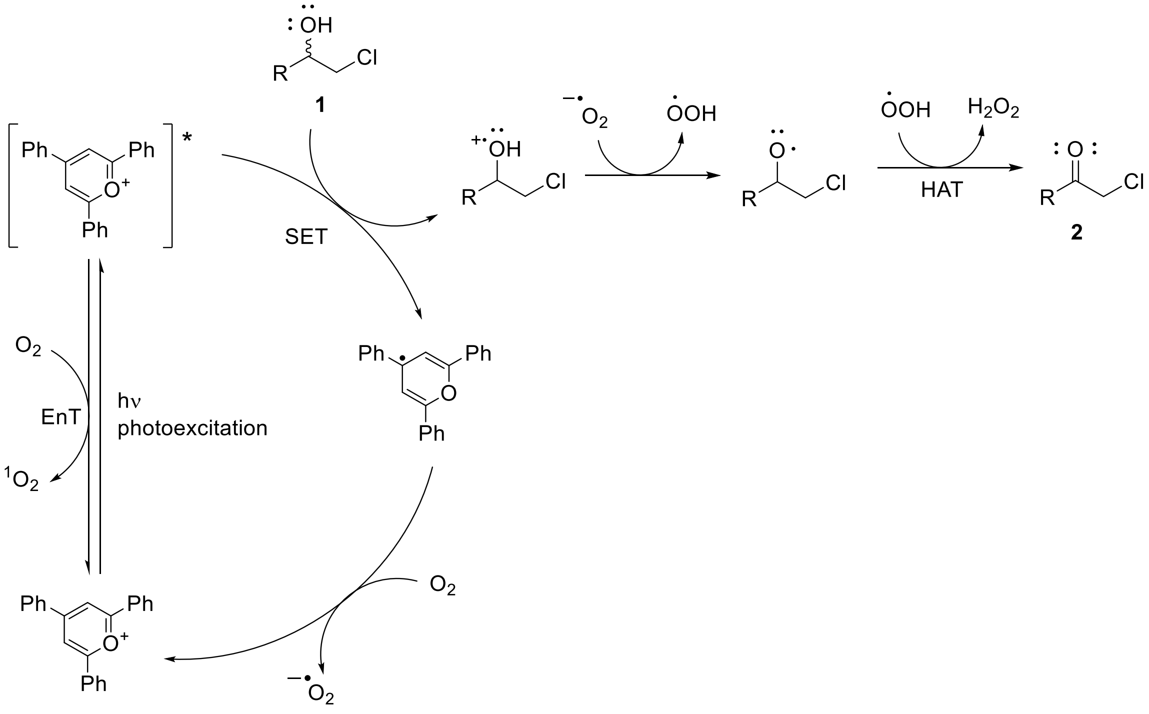

Scheme S2. Plausible mechanistic proposal for TPPT.

Mechanism 1: The 2,4,6-triphenylpyrylium ion (TPP^+) is excited by visible light irradiation to $TPP^{+}*$, which can undergo both photoinduced energy transfer (EnT) or photoinduced single electron transfer (SET) pathways. The EnT from $TPP^{+}*$ to molecular oxygen generates singlet oxygen ($^1O_2$), an excited state of O_2. In SET pathway, $TPP^{+}*$ is reductively quenched by SET due to **1** to give $\mathbf{1}^{\bullet+}$ and $TPP^{\bullet}$. Then, $TPP^{\bullet}$ reacts with O_2 generating the superoxide ($O_2^{\bullet-}$) species regenerating TPP^+. The proton abstraction of $O_2^{\bullet-}$ from $\mathbf{1}^{\bullet+}$ generates the oxyradical intermediate and hydroperoxyl radical ($HOO^{\bullet}$), whose subsequent hydrogen atom transfer (HAT) provides product **2** and hydrogen peroxide, respectively.[25]

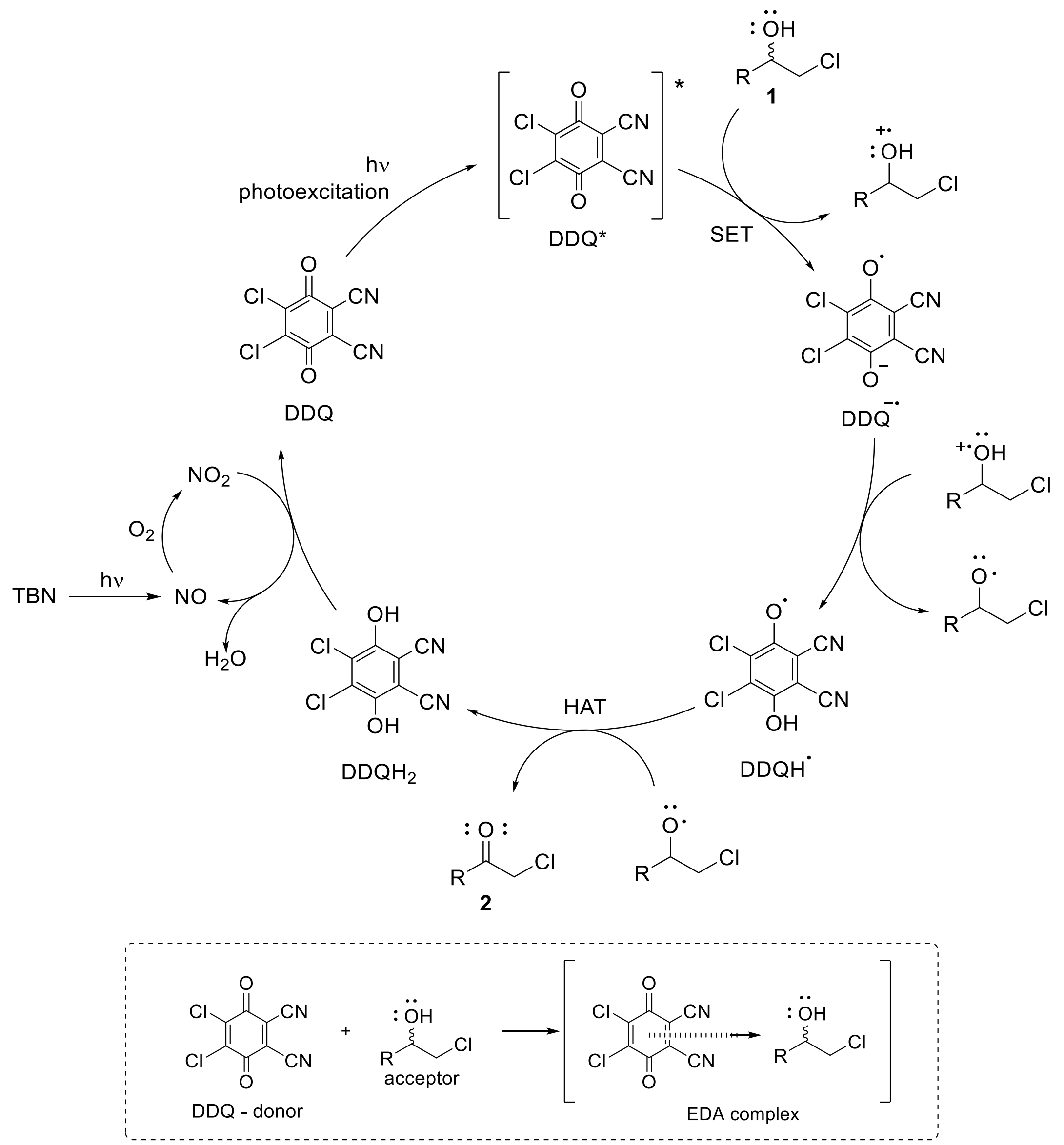

Scheme S3. Plausible mechanism proposal for DDQ.

Mechanism 2: Upon visible-light irradiation, DDQ is excited to its singlet excited state (^{1}DDQ*) that rapidly decays into a triplet state (^{3}DDQ*). The ^{3}DDQ* undergoes photoinduced single electron transfer (SET) pathway. Thus, ^{3}DDQ* is reductively quenched by **1** to give $\mathbf{1}^{\bullet +}$ and DDQ$^{\bullet -}$. Then, both species undergo a hydrogen atom transfer (HAT) process. During the process, DDQ acts as a two-electron acceptor and is reduced to $DDQH_2$, which is reoxidized to DDQ by the TBN•O_2 system to close the catalytic cycle.[24]

UV-Vis spectrometry studies uncovered the formation of an electron donor-acceptor (EDA) complex through the reaction outcome. Substrate **1a** exhibits an absorption band at λ_{max}= 218 nm. Addition of 1 equivalent of DDQ resulted in a bathochromic shift towards λ_{max}= 355 nm. These data are consistent with the formation of an EDA complex between substrate **1a** and DDQ. EDA complexes are characterized by the appearance of an absorption band that is redshifted compared to the absorption bands of the individual components. We propose that excitation of this charge transfer band promotes the initial electron transfer from **1a** to DDQ. The same UV-Vis spectroscopy experiment was conducted using an unsuitable photocatalyst for this oxidation, 9-fluorenone, and another suitable photocatalyst, TPPT.

A. Employing DDQ a bathochromic shift was observed denoting an EDA complex formation.

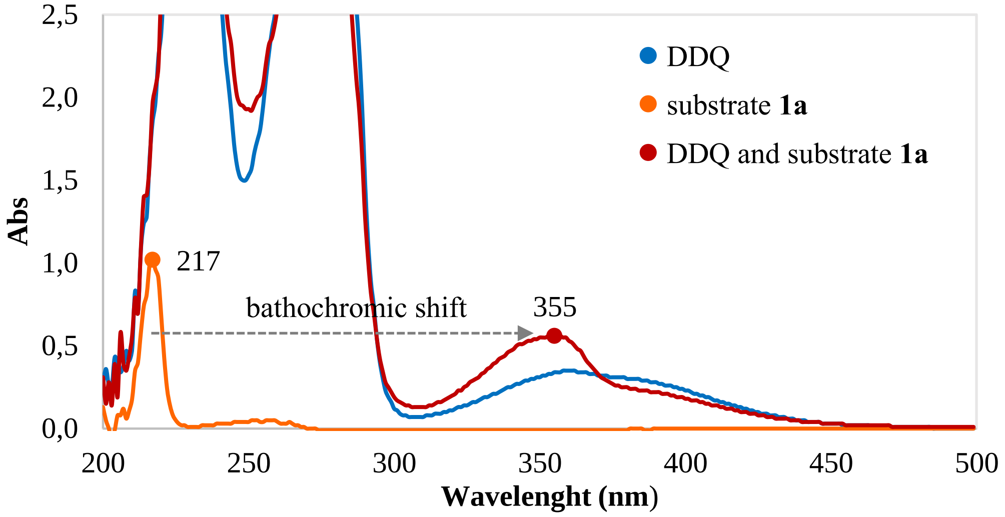

B. Employing TPPT no bathochromic shift was observed.

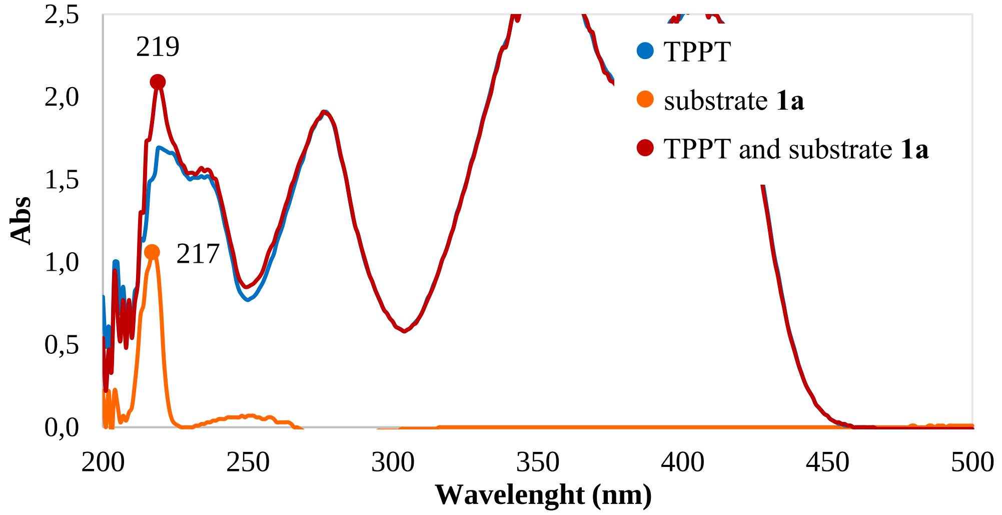

C. Employing 9-fluorenone no bathochromic shift was observed.

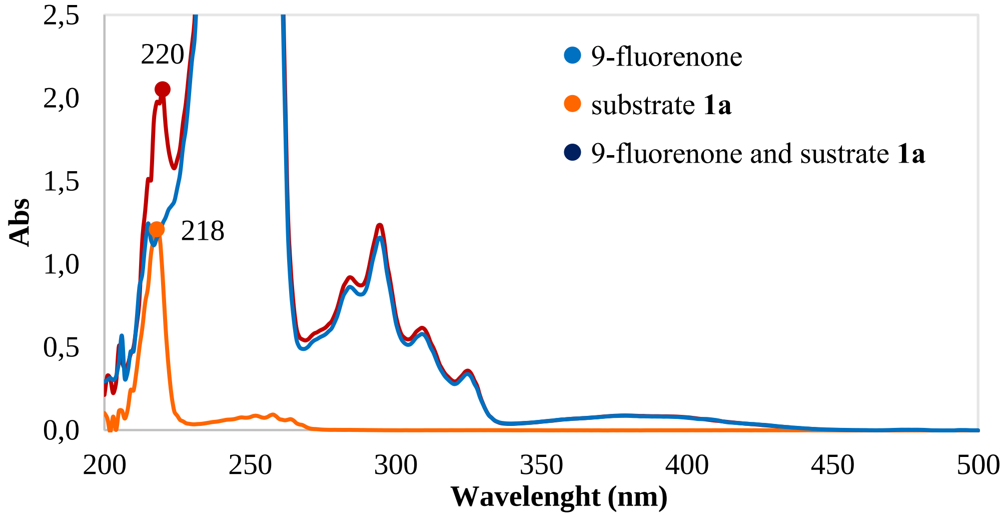

Figure S7. UV-Vis spectrometry studies in order to uncover the formation of an electron donor-acceptor (EDA) complex. For each measurement, the substrate concentration was 0.75 mM.

Table S13. Experiments to unravel the photo-oxidation reaction mechanisms of both photosensitizers studied in this work: TPPT and DDQ.

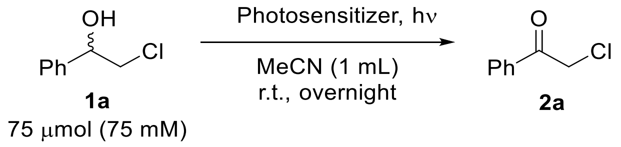

Entry	Photosensitiser	hν	Atmosphere	Temperature	2a (%)[a]
1	TPPT (20 mol%)	White	Air	r.t.	>99
2	TPPT (20 mol%)	White	N_2	r.t.	-
3	TPPT (20 mol%)	-	Air	r.t.	-
4	TPPT (20 mol%)	-	Air	40 ºC	-
5	DDQ (1 equiv)	White	Air	r.t.	>99
6	DDQ (1 equiv)	White	N_2	r.t.	>99
7	DDQ (1 equiv)	-	Air	r.t.	-
8	DDQ (1 equiv)	-	Air	40 ºC	-
9	-	White	Air	r.t.	-

[a] Conversion values were calculated by GC analysis.

Table S14. Radical trapping experiment using TEMPO to unravel the photo-oxidation reaction mechanisms of both photosensitizers studied in this work: TPPT and DDQ.

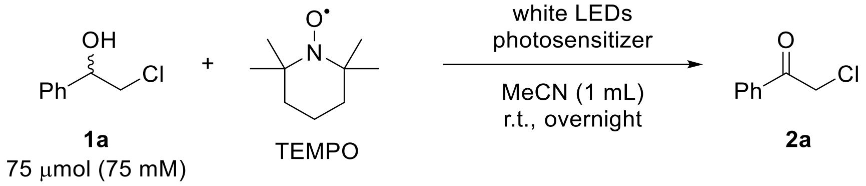

Entry	Radical trap	Photosensitizer	2a (%)[a]
1	-	TPPT (7.5 mol%)	>99
2	TEMPO (1 equiv)	TPPT (7.5 mol%)	43
3	-	DDQ (1 equiv)	>99
4	TEMPO (1 equiv)	DDQ (1 equiv)	38

[a] Conversion values were calculated by GC analysis.

5. Photo-oxidation reaction in continuous flow

5.1. Description of the continuous flow photochemistry system

The continuous flow system was built with PFA tubing (0.8 mm internal diameter, 1.6 mm outside diameter). All connection fittings are compatible with 1/4-28 flat-bottom ports. The reaction solution is pumped into the flow system by a syringe pump equipped with a 5-mL plastic syringe. The flow system is connected sequentially from its inlet to its exit to a stopcock, a photochemical reactor and a 20-psi back pressure regulator (BPR) with its outlet connected to the collector flask, see Figure S8.

The photochemical reactor consists of a coil made up with 8 m of the PFA tubing rolled around a metallic mesh tube (4 mL volume, 6 cm high, 8 cm diameter). The coil is illuminated, at a distance of 1 cm, by white LED strip lights in a box. A fan is used to avoid overheating of the reaction due to the radiation.

The continuous flow system is covered with a paper box to avoid external light contamination.

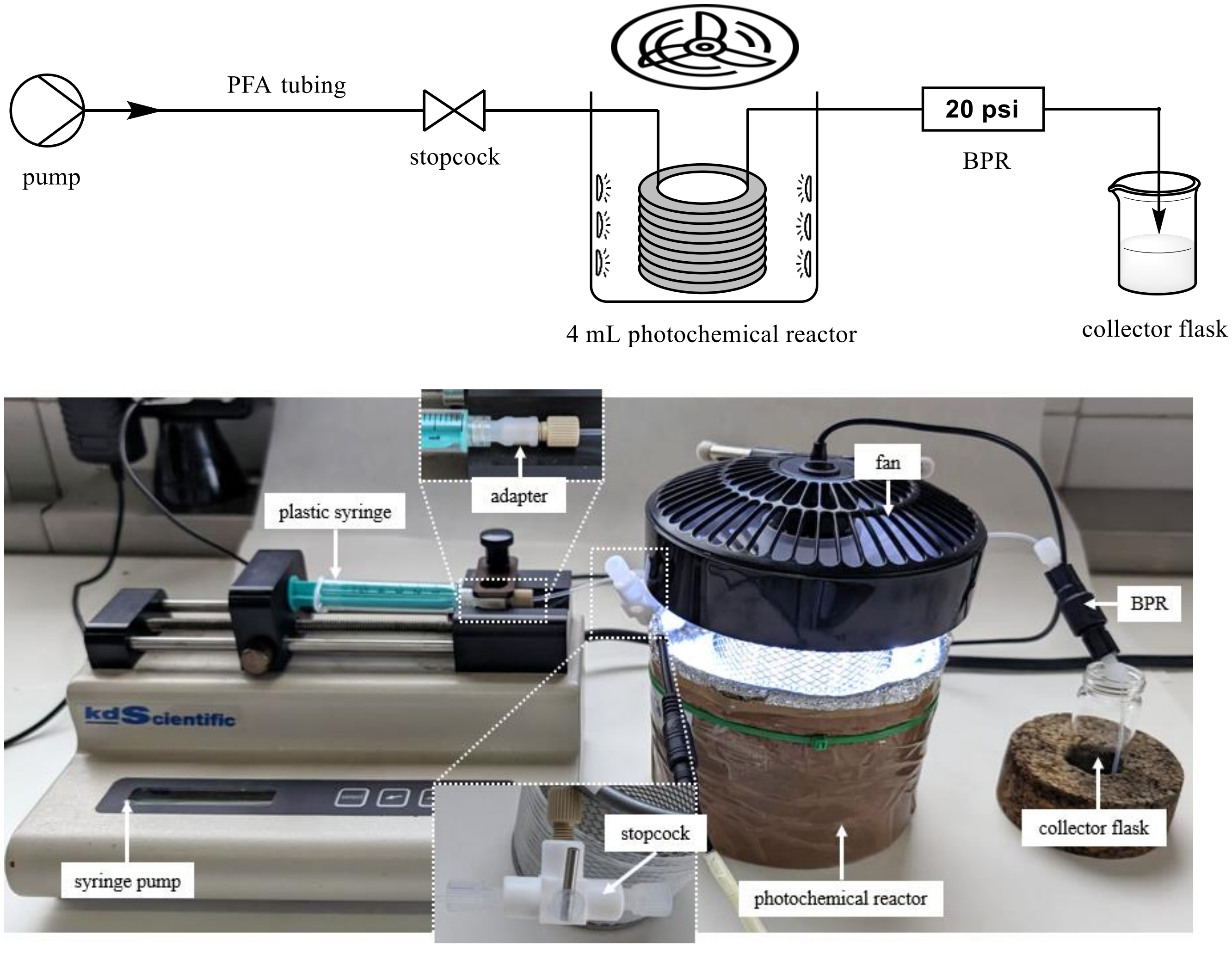

Figure S8. Continuous flow photochemistry system using white LED strip lights.

The detailed description of the materials is described here below:

- Syringe pump from KD Scientific (model No. KDS-100-CE).
- 5-mL plastic syringe with Luer lock.
- PFA tubing, 0.8 mm internal diameter, 1.6 mm outside diameter.

- Adapter female Luer to female 1/4-28 flat-bottom port, a 1.0 mm thru-hole, ETFE body.
- Flangeless fittings for 1/4-28 flat-bottom ports and 1.6 mm outside diameter tubing. Each fitting consists of a PFA nut and a ETFE ferrule.
- Miniature stopcock with three female 1/4-28 flat-bottom port connections, PTFE and FEP body. The unused port is closed with a plug.
- White LED strip lights in a box.
- Coil made up with 8 m of the PFA tubing rolled around a metallic mesh tube (4 mL volume, 6 cm high, 8 cm diameter).
- Miniature fan.
- Back pressure regulator for 20 psi (1.4 bar) with two female 1/4-28 flat-bottom port connections.
- Collector flask.

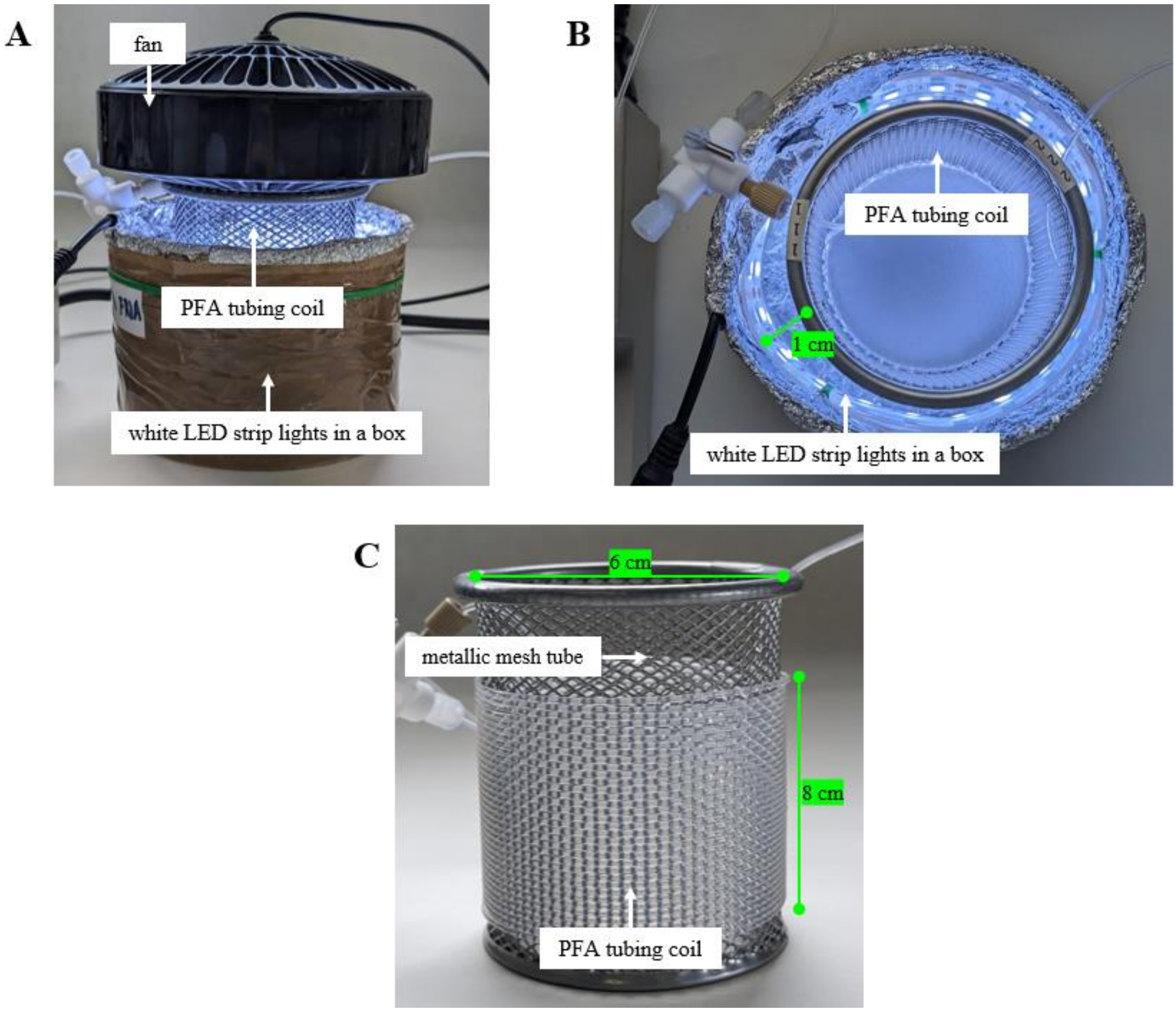

Figure S9. The photochemistry reactor. **A**) Front view; **B**) Top view; **C**) The PFA tubing coil.

5.2. Optimizing the photo-oxidation reaction in continuous flow

To optimize the photo-oxidation process in continuous flow, **1a** was used as model substrate. Given that our continuous flow system lacks support for a gas inlet and since O_2 is crucial for the catalytic system, the reaction solution is pre-bubbled with O_2. However, the reaction failed to achieve complete oxidation. Using stoichiometric DDQ led to full oxidation at a flow rate (**Q**) of 1 mL/h.

The space-time yield (STY) is a measurement of the productivity of the reaction and is given by the equation $\boldsymbol{STY = P/(V \cdot t)}$, where **P** is the amount of product formed (in µmol), **V** is the reaction volume (in mL) and **t** is the reaction time (in hours).

The photo-oxidation reaction of **1a** under the best batch conditions (see Table S22, entry 6), corresponds to STY of 50 µmol mL^{-1} h^{-1}. The implementation of the continuous flow system resulted in a significant increase on the STY value to 300 µmol mL^{-1} h^{-1} (Table S15, entry 3).

Table S15. Optimizing the photo-oxidation reaction in continuous flow of halohydrin **1a** to haloketone **2a** using DDQ as photosensitizer.

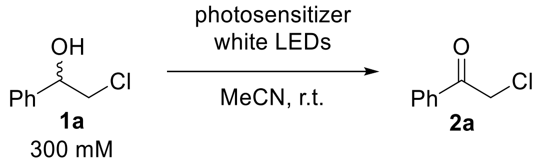

Entry	Photosensitizer	O_2 pre-bubbling[b]	Q (mL/h)	2a (%)[a]
1	DDQ (5 mol%)/ TBN (0.5 equiv)	Yes	0.5	28
2	DDQ (5 mol%)/ TBN (0.5 equiv)	Yes	1	29
3	DDQ (1.1 equiv)	No	1	>99
4	DDQ (1.1 equiv)	No	3	90
5	DDQ (1.1 equiv)	No	4	83

[a] Conversion values were calculated by GC analysis. [b] The reaction solution is pre-bubbled with O_2.

5.3. Experimental protocol for the photo-oxidation in continuous flow of halohydrin 1a

A solution containing **1a** (300 mM, 1 equiv) and DDQ (1.1 equiv) dissolved in MeCN is placed in a plastic syringe, placed in a syringe pump and connected to the inlet of the continuous flow system. Then, the stopcock is switched to allow the reaction solution to enter into the reactor. The lamps are turned on and the solution is pumped at a rate of 1 mL/h. Once all the desired reaction solution has been introduced into the flow reactor, the syringe is switched and MeCN is pumped (1 mL/h) into the reactor to push the reaction solution through the photochemistry reactor and into the collector flask.

6. Photo-oxidation of (2-chloroethyl)benzene to obtain the corresponding haloketone 2a

In order to extend the methodologies reported in this work, the photo-oxidation of (2-chloroethyl)benzene (**4a**) was studied to obtain the corresponding α-chloroketone (**2a**), similar to other reports in the literature with SAS as photocatalyst.[26]

Table S16. Photo-oxidation reaction of (2-chloroethyl)benzene **4a** to obtain the haloketone **2a**.

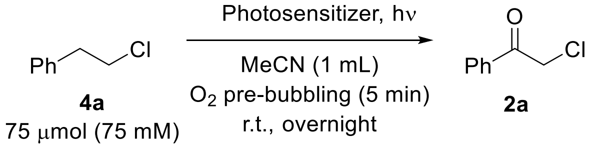

Entry	Photosensitizer	Irradiation	4a (%)[a]	2a (%)[a]
1	TPPT (20 mol%)	Blue	34	51
2	TPPT (20 mol%)	White	37	34
3	DDQ (1.1 equiv)	Blue	67	11
4	DDQ (1.1 equiv)	White	56	24
5	DDQ (5 mol%) / TBN (0.5 equiv)	Blue	71	5
6	DDQ (5 mol%) / TBN (0.5 equiv)	White	53	20

[a] Conversion values were calculated by HPLC analysis.

The general procedures for the photo-oxidation of (2-chloroethyl)benzene to obtain its corresponding α-chloroketone are the same as the ones described in Section 4.4.

7. Asymmetric biocatalytic reduction of haloketones

The study was performed on an analytical scale using 2-chloroacetophenone (**2a**) as model substrate. Our group previously screened various alcohol dehydrogenases (ADHs) for the asymmetric bioreduction of **2a**.[6] The most efficient enzymes identified were *E. coli*/ADH-A (Prelog enzyme leading to (*R*)-**1a**) and *E. coli*/*Lb*ADH (anti-Prelog enzyme leading to (*S*)-**1a**).

Table S17. Asymmetric biocatalytic reduction of haloketones **2a,e-g,j,n** to enantiopure halohydrins **1a,e-g,j,n** with in-house *E.coli* overexpressed ADHs.

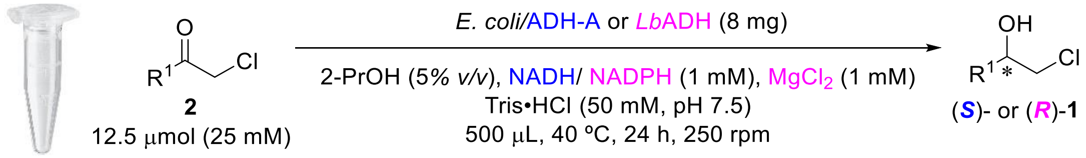

Entry	Product	Enzyme	**1** (%)[a]	*ee* (%)[a]
1	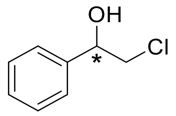 **1a**	ADH-A	>99	>99 (*R*)
2		*Lb*ADH	>99	>99 (*S*)
3	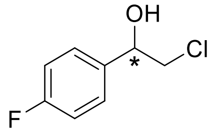 **1e**	ADH-A	>99	>99 (*R*)
4		*Lb*ADH	>99	>99 (*S*)
5	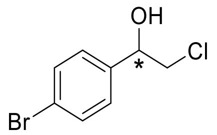 **1f**	ADH-A	>99	>99 (*R*)
6		*Lb*ADH	>99	>99 (*S*)
7	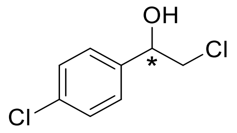 **1g**	ADH-A	>99	>99 (*R*)
8		*Lb*ADH	>99	>99 (*S*)
9	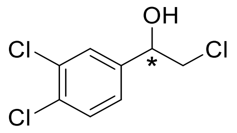 **1j**	ADH-A	>99	>99 (*R*)
10		*Lb*ADH	>99	>99 (*S*)
11	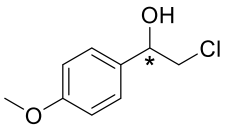 **1n**	ADH-A	>99	>99 (*R*)
12		*Lb*ADH	>99	>99 (*S*)

[a] Conversion and enantiomeric excess values were calculated by HPLC analysis.

7.1. Experimental protocol for the biocatalytic reduction of haloketones 2a,e-g,j,n using ADH-A

α-Chloroketone **2a**,**e**-**g**,**j**,**n** (12.5 μmol, 25 mM), 2-PrOH (5% *v/v*, 25 μL), NADH (1 mM, 25 μL from a 10 mM stock in an aqueous buffer Tris·HCl (50 mM, pH 7.5)), aqueous buffer Tris·HCl (50 mM, pH 7.5) (450 μL) and lyophilized cells of *E. coli* overexpressing ADH-A (8 mg) were successively added to a 1.5-mL Eppendorf tube. Then, the recipient was closed and kept under orbital shaking at 250 rpm and 40 °C for 24 h. After this time, the solution was extracted with MTBE (500 μL). The reaction conversion and the enantiomeric excess values were calculated by HPLC analysis (see Section 10.2).

7.2. Experimental protocol for the biocatalytic reduction of haloketones 2a,e-g,j,n using *Lb*ADH

α-Chloroketone **2a**,**e**-**g**,**j**,**n** (12.5 μmol, 25 mM), 2-PrOH (5% *v/v*, 25 μL), NADPH (1 mM, 25 μL from a 10 mM stock in aqueous buffer Tris·HCl (50 mM, pH 7.5)), $MgCl_2$ (1 mM, 25 μL from a 10 mM stock in aqueous buffer Tris·HCl (50 mM, pH 7.5)), Tris·HCl (50 mM, pH 7.5) (425 μL) and lyophilized cells of *E. coli* overexpressing *Lb*ADH (8 mg) were successively added to a 1.5 mL-Eppendorf tube. Then, the recipient was closed and kept under orbital shaking at 250 rpm and 40 °C for 24 h. After this time, the solution was extracted with MTBE (500 μL). The reaction conversion and the enantiomeric excess values were calculated by HPLC analysis (see Section 10.2).

8. One-pot two-step sequential photo-biocatalytic deracemization of halohydrins

8.1. Optimizing the photo-biocatalytic deracemization

The study was performed using **1a** as model substrate. Firstly, the photo-oxidation reaction was tested in the presence of water, Table S18. Both photosensitizers, TPPT and DDQ, were unable to work in the presence of water. Therefore, we propose a one-pot two-step sequential deracemization methodology, combining an initial photo-oxidation in organic solvent and a subsequent bioreduction in organic-aqueous medium.

Table S18. Influence of water in the photo-oxidation reaction of halohydrin **1a** to haloketone **2a** using either TPPT 20 mol% (**method A**) or DDQ 1 equiv (**method B**).

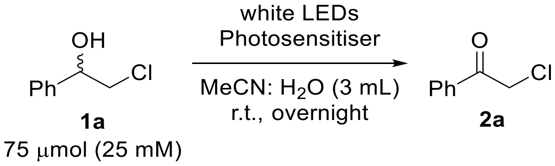

		2a (%)[a]	
**Entry**	**H_2O (% *v*/*v*)**	**TPPT (20 mol%)**	**DDQ (1 equiv)**
1	20	n.d.	n.d.
2	40	n.d.	n.d.
3	60	n.d.	n.d.
4	80	n.d.	n.d.

[a] Conversion values were calculated by GC analysis. n.d.: Not detected.

Secondly, a study on the organic-aqueous medium for the biocatalytic reduction was performed by studying the maximum % *v*/*v* of MeCN tolerated by each enzyme selected in Section 7.

Table S19. Study of the % *v*/*v* of MeCN tolerated by ADH-A in the asymmetric biocatalytic reduction of haloketone **2a** to enantiopure halohydrin **1a**.

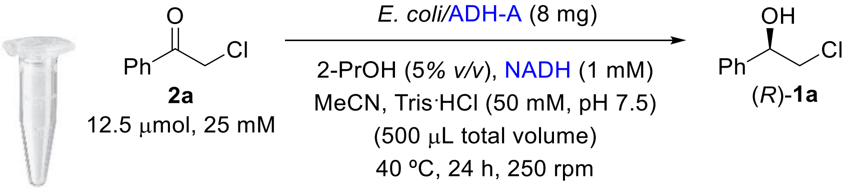

Entry	MeCN (% *v*/*v*)	1a (%)[a]	(*R*)-1a *ee* (%)[a]
1	5	>99	>99
2	7.5	>99	>99
3	10	>99	95
4	15	43	86

[a] Conversion and enantiomeric excess values were calculated by HPLC analysis.

Table S20. Study of the % *v*/*v* of MeCN tolerated by in the asymmetric biocatalytic reduction of haloketone **2a** to enantiopure halohydrin **1a**.

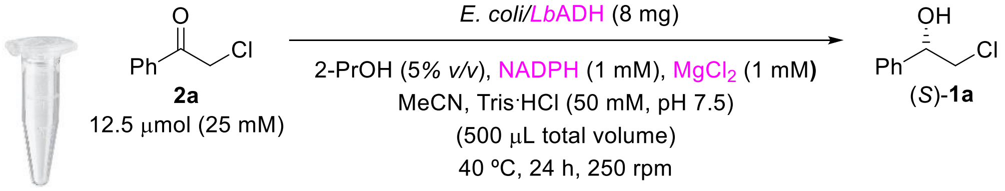

Entry	MeCN (% *v*/*v*)	1a (%)[a]	(*S*)-1a *ee* (%)[a]
1	5	>99	>99
2	10	>99	>99
3	15	>99	>99
4	20	>99	>99
5	30	>99	>99
6	50	>99	>99
7	70	>99	>99
8	80	17	>99
9	90	12	>99

[a] Conversion and enantiomeric excess values were calculated by HPLC analysis.

As shown in Table S20 entry 7, the bioreduction of ketone **2a** using lyophilized *Lb*ADH at 70% *v/v* MeCN resulted in complete conversion, demonstrating a successful example of high organic solvent tolerance. This outcome highlights the impressive performance of *Lb*ADH. To investigate enzyme stability and study long-term detrimental effects, we conducted a residual activity assay. Therefore, lyophilized LbADH was preincubated in 70% v/v MeCN in Tris·HCl buffer (50 mM, pH 7.5), with 5% v/v 2-propanol, NADPH (1 mM) and MgCl2 (1 mM) at 40 °C for various time intervals (0 to 24 hours), without substrate. After the incubation period, 2-chloroacetophenone 2a was added, and the reaction proceeded for 1 hour under standard conditions. The conversion to the corresponding alcohol **1a** was then measured, and the residual activity was calculated relative to the non-incubated sample.

Table S21. Residual activity assay for the study of *Lb*ADH enzyme stability and study long-term detrimental effects in asymmetric biocatalytic reduction of haloketone **2a** to enantiopure halohydrin **1a**.

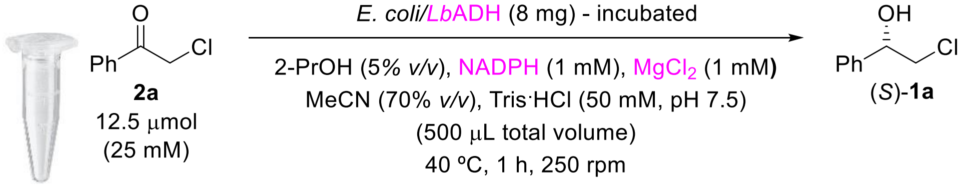

Entry	Preincubation time (h)	1a (%)[a]	*e.e.* (%)[a]	Relative residual activity (%)
1	0	99	>99 (*S*)	>99
2	1	98	>99 (*S*)	99
3	4	92	>99 (*S*)	93
4	8	90	>99 (*S*)	91
5	24	62	>99 (*S*)	63

[a] Lyophilized *E. coli*/ *Lb*ADH was preincubated, without substrate, in 70% v/v MeCN in Tris·HCl buffer (50 mM, pH 7.5), with 5% v/v 2-propanol, NADPH (1 mM) and $MgCl_2$ (1 mM) at 40 °C for various time intervals (0 to 24 h). After the incubation period, 2-chloroacetophenone (**2a**) was added, and the reaction proceeded for 1 h under standard conditions before measuring the conversion.

[b] Conversion values and enantiomeric excess values were calculated by HPLC analysis.

These results show that LbADH retains over 90% of its catalytic activity after up to 8 h in 70% v/v MeCN. While a decline in activity is observed with prolonged exposure the enzyme remains sufficiently stable and functional to support the one-pot deracemization cascade, which completes within a much shorter time frame.

This study also revealed that complete conversion in the reactions with the model substrate **2a** was achieved after only 1 h (entry 1), revealing that the bioreduction step proceeds significantly faster than expected. In our current project, the biocatalytic reactions were uniformly conducted over 24 h based on our experience with previous works. Thus, we do not focus on optimizing reaction time for the biocatalytic step. This unexpected result highlights the remarkable catalytic efficiency of *Lb*ADH under the reported conditions and suggests that future optimization could substantially shorten reaction times, increasing the overall practicality of the method.

The low % *v*/*v* of MeCN tolerated by ADH-A could complicate a one-pot strategy as this implies the use of an optimum flask for a low-volume photochemical step and a high-volume biochemical step. Therefore, studies were conducted with another Prelog enzyme, Table S22.

Table S22. Study of the % *v*/*v* of MeCN tolerated by ADH-T in the asymmetric biocatalytic reduction of haloketone **2a** to enantiopure halohydrin **1a**.

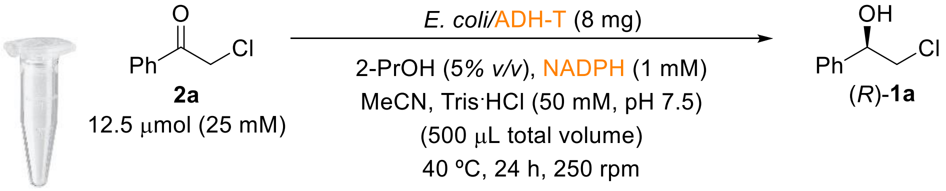

Entry	MeCN (% *v*/*v*)	1a (%)[a]	(*R*)-1a *ee* (%)[b]
1	5	96	81
2	10	66	58
3	15	37	30
4	20	37	20
5	30	10	10

[a] Conversion and enantiomeric excess values were calculated by HPLC analysis.

The volume of organic solvent and reaction time for the initial photocatalytic oxidation were re-optimized, selecting 250 µL of MeCN and a 6 h reaction time as optimum. This allowed the one-pot methodology with ADH-A implying 7.5% *v*/*v* of MeCN after dilution. Regarding the one-pot methodology with *Lb*ADH, lowering the volume of organic solvent in the bioreduction step must be compensated with an increase of water to maintain a suitable concentration of substrate. Therefore, a 15% *v*/*v* of MeCN was selected despite *Lb*ADH tolerated up to 70% *v*/*v*.

Table S23. Re-optimization of the organic solvent employed in the photo-oxidation reaction of halohydrin **1a** to haloketone **2a** using the DDQ 5 mol% (**method C**).

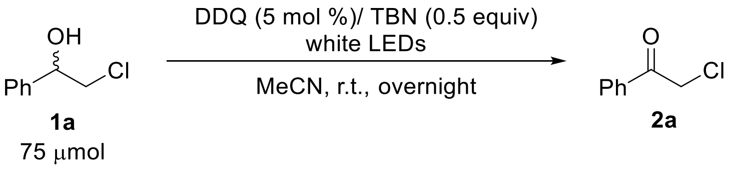

Entry	MeCN (µL)	Conc. 1a (mM)	Time (h)	2a (%)[a]
1	1000	75	4	>99
2	700	108	4	90
3			6	95
4	500	151	4	70
5			6	99
6	250	302	6	>99

[a] Conversion values were calculated by GC analysis.

8.2. Substrate scope of the photo-biocatalytic deracemization

Finaly, both steps were combined in a one-pot two step sequential methodology which was able to yield 12 enantiopure chlorohydrins with either (*R*) or (*S*) configuration.

Table S24. One-pot two-step deracemization methodology of halohydrins **1a,e-g,j,n** combining an initial photocatalytic oxidation and a subsequent asymmetric biocatalytic reduction.

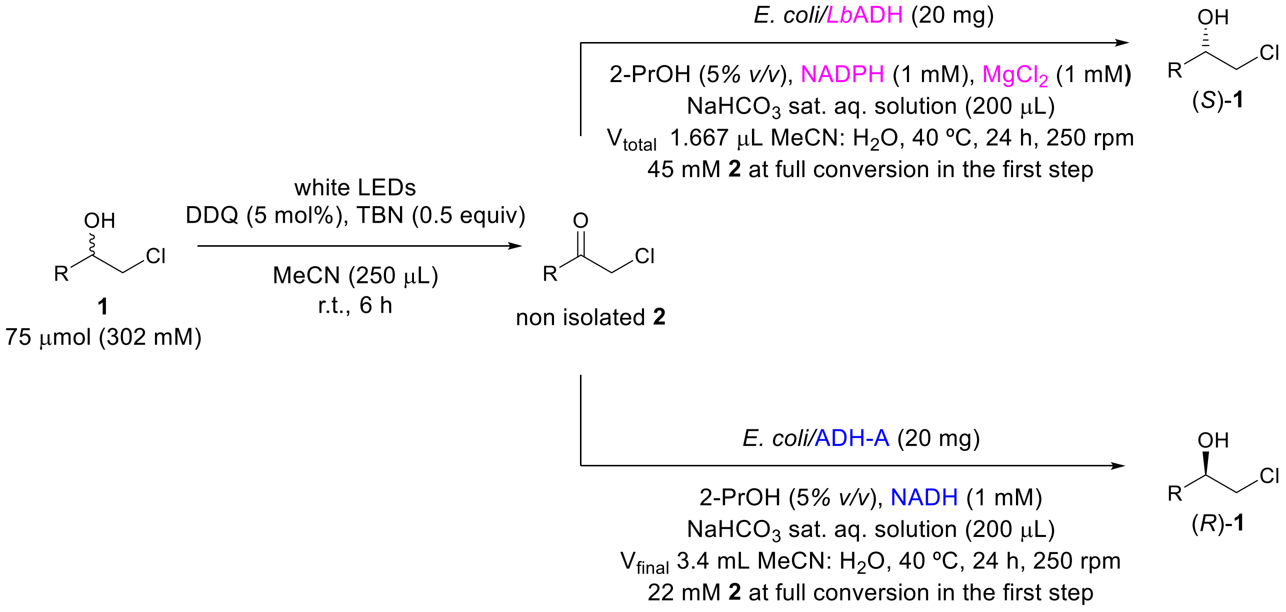

Entry	Product	Enzyme	**1** (%)[a]	*ee* (%)[a]
1	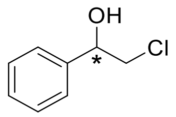	ADH-A	>99	>99 (*R*)
2	**1a**	*Lb*ADH	>99	>99 (*S*)
3	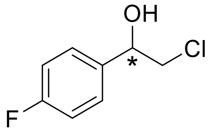	ADH-A	>99	>99 (*R*)
4	**1e**	*Lb*ADH	>99	>99 (*S*)
5	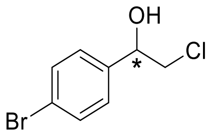	ADH-A	>99	>99 (*R*)
6	**1f**	*Lb*ADH	>99	>99 (*S*)
7	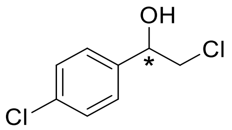	ADH-A	>99	>99 (*R*)
8	**1g**	*Lb*ADH	>99	>99 (*S*)

Entry	Product	Enzyme	1 (%)[a]	*ee* (%)[a]
9	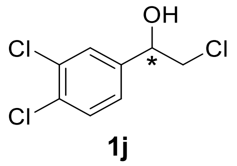 **1j**	ADH-A	>99	>99 (*R*)
10		*Lb*ADH	>99	>99 (*S*)
11	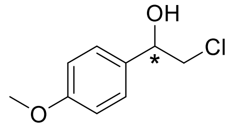 **1n**	ADH-A	>99	>99 (*R*)
12		*Lb*ADH	>99	>99 (*S*)

[a] Conversion values and enantiomeric excess values were calculated by HPLC analysis.

8.3. Experimental protocol for the photo-biocatalytic deracemization of 2a,e-g,j,n

In a 4-mL vial, the β-halohydrin **1** (75 μmol) was dissolved in MeCN (250 μL) and DDQ (5 mol%) and TBN (0.5 equiv) were added. For **1j**, DDQ (50 mol%) and TBN (1.5 equiv) were employed instead. The vial was closed with a screw cap. The reaction solution was stirred overnight under white light irradiation at room temperature, using the photochemistry set-up described in Section 4.4. For compound **1a**, 4 h of reaction time were used.

a) **To obtain (*S*)-1 enantiomer:** without organic solvent evaporation, distilled water (800 μL), $NaHCO_3$ (200 μL of a saturated aqueous solution), 2-PrOH (5% *v/v*, 83 μL), NADPH (1 mM, 167 μL from a 10 mM stock in Tris·HCl (50 mM, pH 7.5)), $MgCl_2$ (1 mM, 167 μL from a 10 mM stock in Tris·HCl (50 mM, pH 7.5)) and lyophilized cells of *E. coli* overexpressing *Lb*ADH (20 mg) were added. Then, the vial was closed with a screw cap and kept under orbital shaking at 250 rpm and 40 °C for 24 h. After this time, the solution was extracted with MTBE (500 μL). The reaction conversion and the enantiomeric excess values were calculated by HPLC analysis (see Section 10.2).

b) **To obtain (*R*)-1 enantiomer:** without organic solvent evaporation, distilled water (2.44 mL), $NaHCO_3$ (200 μL of a saturated aqueous solution), 2-PrOH (5% *v/v*, 170 μL), NADH (1 mM, 340 μL from a 10 mM stock in Tris·HCl (50 mM, pH 7.5)) and lyophilized cells of *E. coli* overexpressing ADH-A (20 mg) were added. Then, the vial was closed with a screw cap and kept under orbital shaking at 250 rpm and 40 °C for 24 h. After this time, the solution was extracted with MTBE (500 μL). The reaction conversion and the enantiomeric excess values were calculated by HPLC analysis (see Section 10.2).

8.4. General procedure for the deracemization of halohydrin 1a in batch and semipreparative scale

The study was performed using **1a** as model substrate. The semipreparative scale-up methodology was done employing *Lb*ADH in a one-pot fashion. The semipreparative scale-up methodology employing ADH-A was not in one-pot as the post-photoreaction crude must be transferred to a bigger flask due to final volume issues. To solve this problem, a continuous flow set-up is proposed, see Section 5.

In a 25-mL vial, the β-halohydrin **1** (753 μmol) was dissolved in MeCN (2.5 mL) and DDQ (5 mol%) and TBN (0.5 equiv) were added. The vial was closed with a screw cap. The reaction solution was stirred overnight under white light irradiation at room temperature, using the photochemistry setup described in Section 4.4.

a) **To obtain (*S*)-1 enantiomer:** without organic solvent evaporation, distilled water (11.34 mL), $NaHCO_3$ (2 mL of a saturated aqueous solution), 2-PrOH (5% *v/v*, 830 µL), NADPH (1 mM, 14 mg), $MgCl_2$ (1 mM, 3.4 mg) and lyophilized cells of *E. coli* overexpressing *Lb*ADH (200 mg) were added. Then, the vial was closed with a screw cap and kept under orbital shaking at 250 rpm and 40 °C for 24 h. After this time, the solution was extracted with MTBE (500 µL). The reaction conversion and the enantiomeric excess values were calculated by HPLC analysis (see Section 10.2).

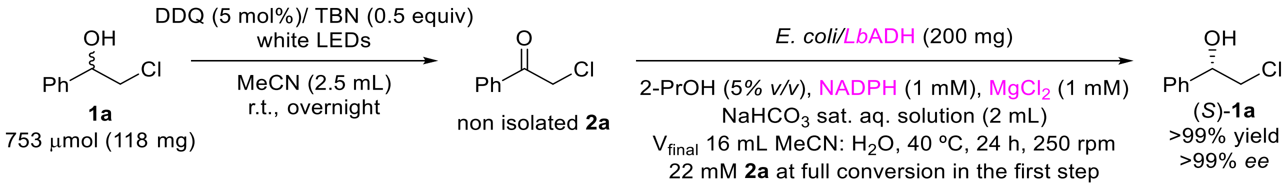

b) **To obtain (*R*)-1 enantiomer:** without organic solvent evaporation, the reaction mixture was poured into a 100-mL Erlenmeyer flask and distilled water (27.17 mL), $NaHCO_3$ (2 mL of a saturated aqueous solution), 2-PrOH (5% *v/v*, 1.67 mL), NADH (1 mM, 22.9 mg) and lyophilized cells of *E. coli* overexpressing ADH-A (200 mg) were added. Then, the vial was closed with a screw cap and kept under orbital shaking at 250 rpm and 40 °C for 24 h. After this time, the solution was extracted with MTBE (500 µL). The reaction conversion and the enantiomeric excess values were calculated by HPLC analysis (see Section 10.2).

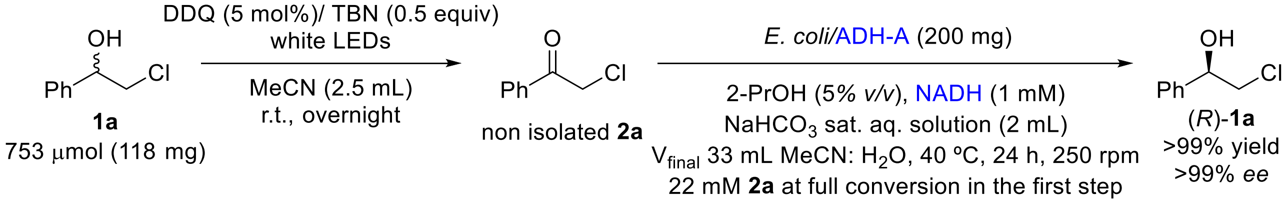

9. Enantiopure epoxide formation sequence

The optimization of the epoxide formation process from alcohol **1a**, was carried out considering the final conditions of the deracemization methodology with each enzyme.

Table S25. Optimizing the epoxide **5a** formation from alcohol **1a** in terms of base amount.

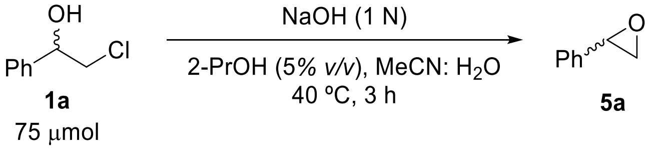

Entry	NaOH (equiv)	V_{final} (mL)	MeCN (% *v/v*)	5a (%)[a]
1	1	3.40 (22 mM)	7.5	>99
2	1.2	3.40 (22 mM)	7.5	>99
3	1.4	3.40 (22 mM)	7.5	>99
4	1	1.67 (45 mM)	15	96
5	1.2	1.67 (45 mM)	15	>99
6	1.4	1.67 (45 mM)	15	>99

[a] Conversion values were calculated by GC analysis.

Table S26. Optimizing the enantiopure epoxide **5a** formation from racemic alcohol **1a** in terms of base amount.

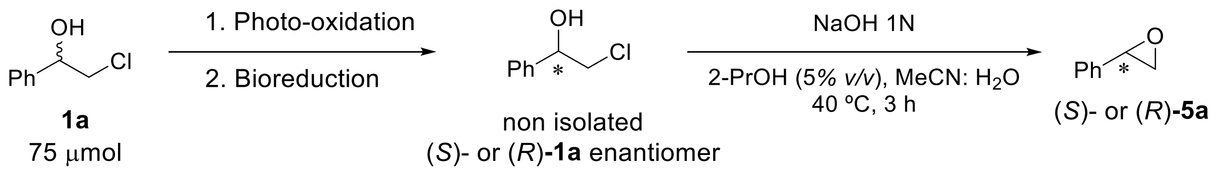

Entry	NaOH (equiv)	Enzyme	pH$_o$	pH$_{final}$	5a (%)[a]	5a *ee* (%)[b]
1	1.2	ADH-A	-	-	33	-
2	2	ADH-A	6	7.5	84	-
3	3	ADH-A	6	12	>99	>99 (*R*)
4	4	ADH-A	6	14	>99	>99 (*R*)
5	5	ADH-A	6	14	>99	>99 (*R*)
6	1.2	*Lb*ADH	-	-	51	-
7	2	*Lb*ADH	6	7.5	93	-
8	3	*Lb*ADH	6	12	>99	>99 (*S*)
9	5	*Lb*ADH	6	14	>99	>99 (*S*)

[a] Conversion values were calculated by GC analysis. [b] Enantiomeric excess values were calculated by HPLC analysis.

9.1. Experimental protocol for the enantiopure epoxide formation sequence

To obtain the desired **5a** enantiomer, the previously described one-pot two-step photo-biocatalytic deracemization protocol was followed using either *Lb*ADH or ADH-A, see Section 8.3. Then, NaOH (1 M, 3 equiv) was added, and the mixture kept under magnetic stirring at 40 °C for 3 h. After this time, the solution was extracted with MTBE (500 µL). The reaction conversion values were calculated by GC and the enantiomeric excess values were calculated by HPLC analysis.

10. Analytical data

10.1. GC analyses

Conversion values towards α-chloroketones (**2**) through photo-oxidation protocols, and the formation of epoxide **5a** through photo-oxidation sequences, or by direct epoxidation reaction, were determined by GC analyses using a chromatograph equipped with a flame ionization detector (FID) detector and an Agilent HP-1 column (30 m x 0.32 mm x 0.25 µm). The analytical conditions are indicated in Table S27.

Table S27. GC analytical conditions and retention times for the determination of conversion values.

Entry	Substrate	Program[a]	Retention time (min)
1	**1a**	110/0/2.5/120/0/10/200/1	3.4
2	**2a**	110/0/2.5/120/0/10/200/1	4.0
3	**3a**	110/0/2.5/120/0/10/200/1	2.0
4	**1e**	110/0/2.5/120/0/10/200/1	4.2
5	**2e**	110/0/2.5/120/0/10/200/1	3.4
6	**3e**	110/0/2.5/120/0/10/200/1	1.9
7	**1f**	110/0/2.5/120/0/10/200/1	8.2
8	**2f**	110/0/2.5/120/0/10/200/1	7.4
9	**3f**	110/0/2.5/120/0/10/200/1	4.5
10	**1g**	110/0/2.5/120/0/10/200/1	8.2
11	**2g**	110/0/2.5/120/0/10/200/1	7.4
12	**3g**	110/0/2.5/120/0/10/200/1	4.5
13	**1h**	110/0/2.5/120/0/10/200/1	11.2
14	**2h**	110/0/2.5/120/0/10/200/1	10.4
15	**3h**	110/0/2.5/120/0/10/200/1	6.0
16	**1i**	110/0/2.5/120/0/10/200/1	7.9
17	**2i**	110/0/2.5/120/0/10/200/1	6.4
18	**3i**	110/0/2.5/120/0/10/200/1	3.2
19	**1j**	110/0/2.5/120/0/10/200/1	9.6
20	**2j**	110/0/2.5/120/0/10/200/1	8.6
21	**3j**	110/0/2.5/120/0/10/200/1	5.3
22	**1k**	110/0/2.5/120/0/10/200/1	8.6
23	**2k**	110/0/2.5/120/0/10/200/1	7.5
24	**3k**	110/0/2.5/120/0/10/200/1	4.6
25	**1l**	110/0/2.5/120/0/10/200/1	4.2
26	**2l**	110/0/2.5/120/0/10/200/1	3.1

Entry	Substrate	Program[a]	Retention time (min)
27	**1m**	110/0/2.5/120/0/10/200/1	10.4
28	**2m**	110/0/2.5/120/0/10/200/1	8.9
29	**1n**	110/0/2.5/120/0/10/200/1	7.7
30	**2n**	110/0/2.5/120/0/10/200/1	8.5
31	**1o**	110/0/2.5/120/0/10/200/1	7.8
32	**2o**	110/0/2.5/120/0/10/200/1	7.2
33	**1p**	110/0/2.5/120/0/10/200/1	11.1
34	**2p**	110/0/2.5/120/0/10/200/1	12.1
35	**1q**	110/0/2.5/120/0/10/200/1	5.8
36	**2q**	110/0/2.5/120/0/10/200/1	5.2
37	**3q**	110/0/2.5/120/0/10/200/1	2.8
38	**1r**	110/0/2.5/120/0/10/200/1	11.9
39	**2r**	110/0/2.5/120/0/10/200/1	12.8
40	**1s**	110/0/20/175/0/5/180/0/1.5/190/0/20/200/0	6.6
41	**2s**	110/0/20/175/0/5/180/0/1.5/190/0/20/200/0	10.4
42	**3s**	110/0/20/175/0/5/180/0/1.5/190/0/20/200/0	3.8
43	**1t**	110/0/2.5/120/0/10/200/1	11.3
44	**2t**	110/0/2.5/120/0/10/200/1	11.8
18	**5a**	90/0/1/110/2/30/200/1	2.9

[a] GC program: initial temp. (ºC) / time (min) / ramp (ºC/min) / temp. (ºC) / time (min) / ramp (ºC/min) / final temp. (ºC) / time (min). Inlet: heater 250 ºC, total flow: 58.7 mL/ min. Column: 2.7 mL/min. FID detector: heater 300 ºC.

10.2. HPLC analyses

Conversion values of α-chloroketones **2a-c** and enantiomeric excess of chiral alcohols were determined by HPLC analyses employing an Agilent 1100 Series chromatograph equipped with a VIS-UV detector. Daicel columns Chiralcel OJ-H (25 cm x 4.6 mm, 5 µm particle size) or Chiralpak IA (25 cm x 4.6 mm, 5 µm particle size) were used as stationary phases. The experimental conditions are indicated in Tables S28 and S29.

Table S28. HPLC analytical conditions and retention times for the determination of conversion values.[a]

Entry	Substrate	*n*-Hexane/ 2-propanol (*v*/*v*)	Flow (mL/ min)	Rt (min)
1	**1a**	95:5	0.6	36.8 and 40.7
2	**2a**	95:5	0.8	24.6
3	**4a**	95:5	0.8	7.2
4	**1b**	90:10	1.0	22.5 and 23.6
5	**2b**	90:10	1.0	9.2
6	**1c**	95:5	0.8	27.3 and 29.8
7	**2c**	95:5	0.8	20.1
8	**2e**	95:5	0.6	30.0
9	**2f**	95:5	0.6	44.1
10	**2g**	90:10	0.8	17.9
11	**2j**	95:5	0.6	33.5
12	**2n**	95:5	0.6	17.3

[a] A Chiralcel OJ-H (4.6 cm diameter, 25 cm length and 5 µm particle size) was used at a temperature of 25 ºC.

Table S29. HPLC analytical conditions and retention times for the determination of enantiomeric excess values.

Entry	Substrate	Column	T (ºC)	*n*-Hexane/ 2-propanol (*v*/*v*)	Flow (mL/ min)	(*S*)- (min)	(*R*)- (min)
1	**1a**	Chiralcel OJ-H	25	95:5	0.6	40.7	36.8
2	**1e**	Chiralcel OJ-H	25	95:5	0.6	37.6	36.2
3	**1f**	Chiralcel OJ-H	25	95:5	0.6	37.9	33.8
4	**1g**	Chiralcel OJ-H	25	90:10	0.8	14.7	13.8
5	**1j**	Chiralcel OJ-H	25	95:5	0.6	32.0	28.1
6	**1n**	Chiralcel OJ-H	25	95:5	0.6	54.0	49.8
7	**5a**	Chiralpak IA	30	98:2	0.8	6.5	6.2

[a] A Chiralcel OJ-H (4.6 mm diameter, 25 cm length and 5 µm particle size) and Chiralpak IA (4.6 mm diameter, 25 cm length and 5 µm particle size) a were used.

HPLC separation for both enantiomers of racemic alcohol 1a

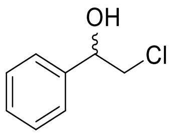

(±)-1a

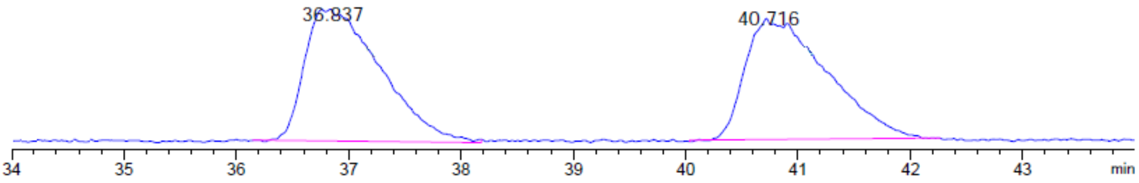

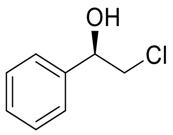

(*R*)-**1a**

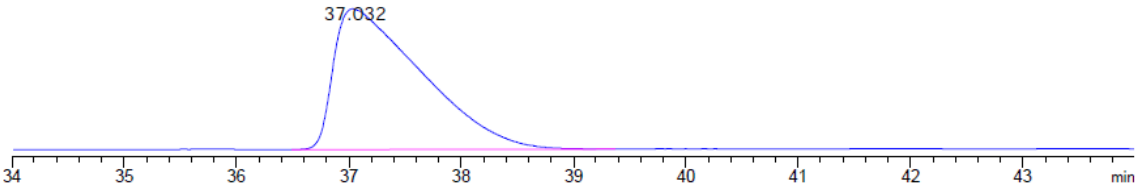

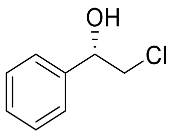

(*S*)-**1a**

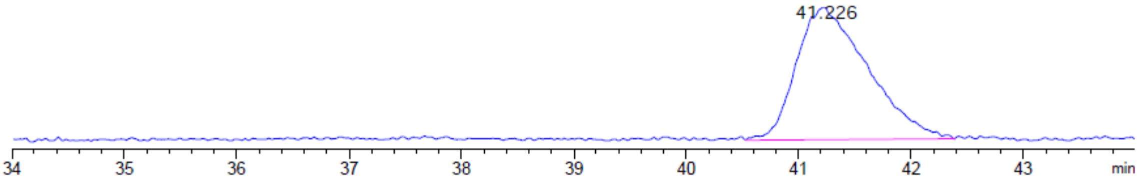

HPLC separation for both enantiomers of racemic alcohol 1e

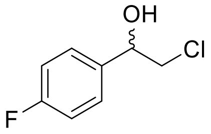

(±)-**1e**

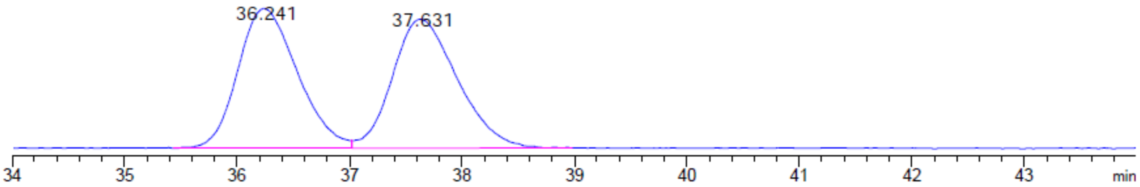

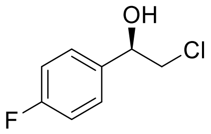

(*R*)-**1e**

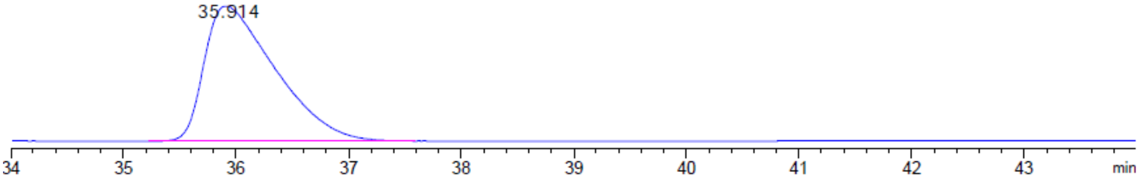

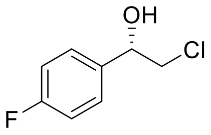

(*S*)-**1e**

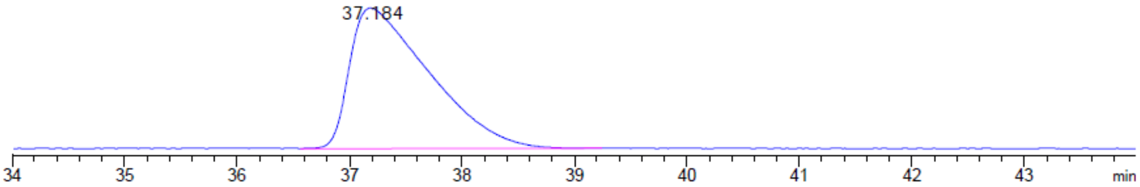

HPLC separation for both enantiomers of racemic alcohol 1f

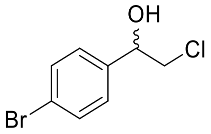

(±)-1f

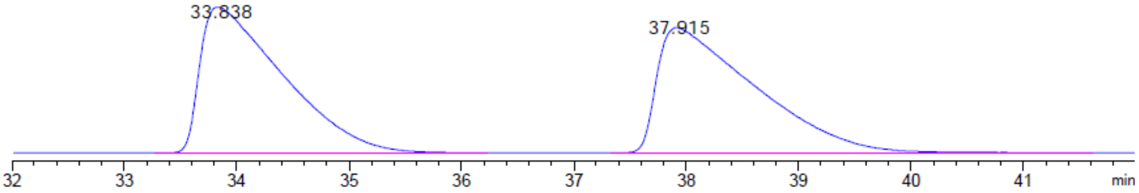

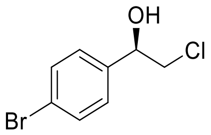

(*R*)-**1f**

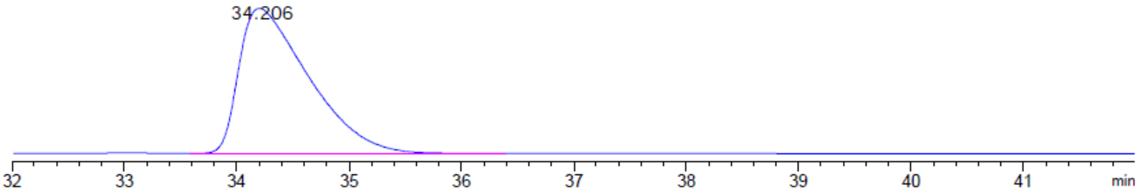

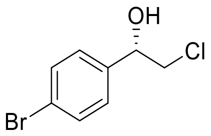

(*S*)-**1f**

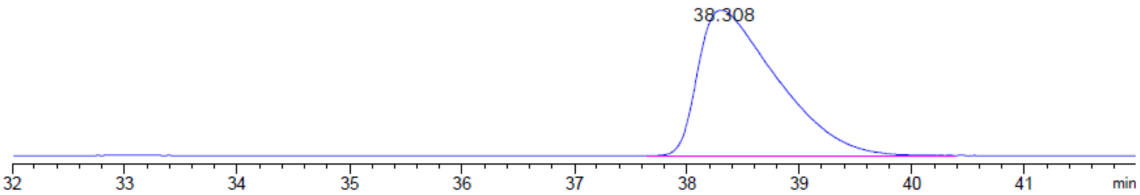

HPLC separation for both enantiomers of racemic alcohol 1g

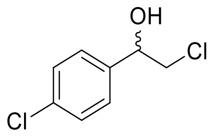

(±)-1g

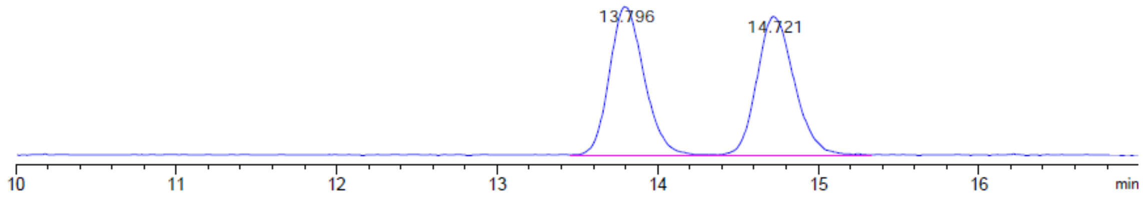

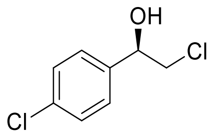

(*R*)-**1g**

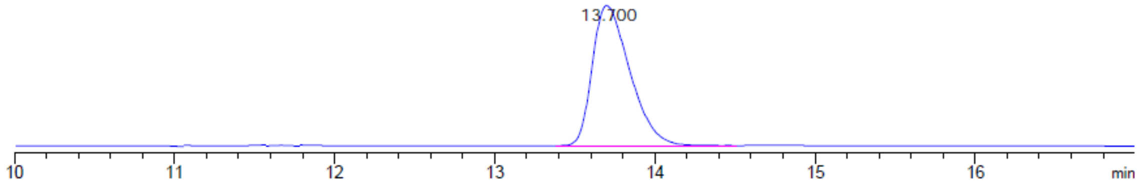

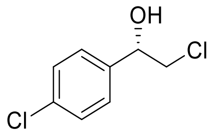

(*S*)-1g

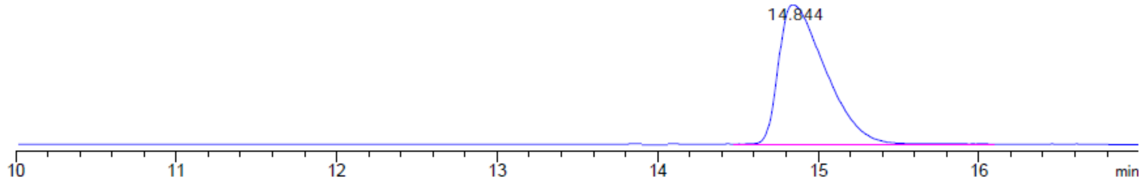

HPLC separation for both enantiomers of racemic alcohol 1j

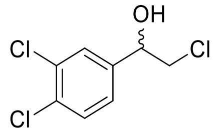

(±)-1j

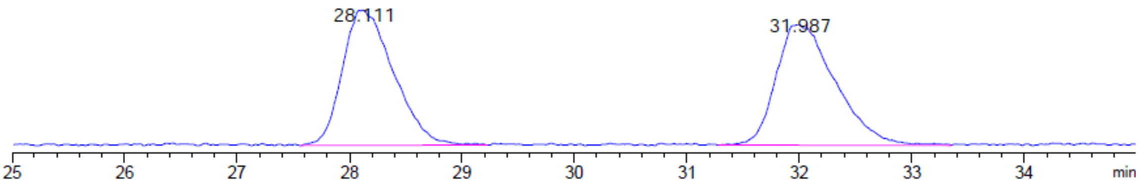

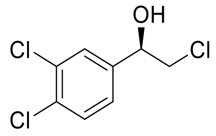

(*R*)-**1j**

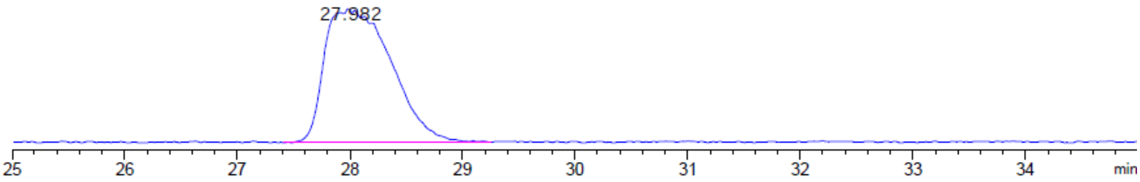

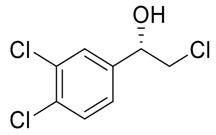

(*S*)-**1j**

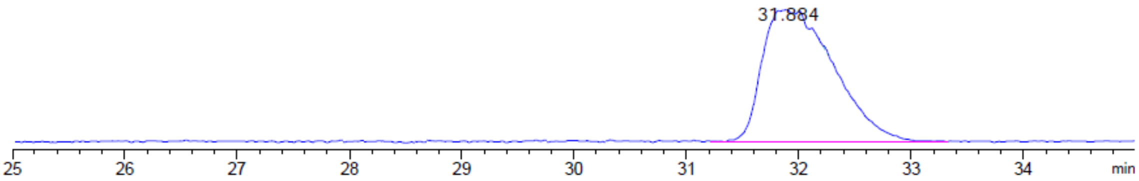

HPLC separation for both enantiomers of racemic alcohol 1n

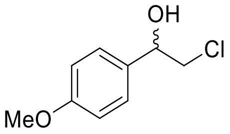

(±)-1n

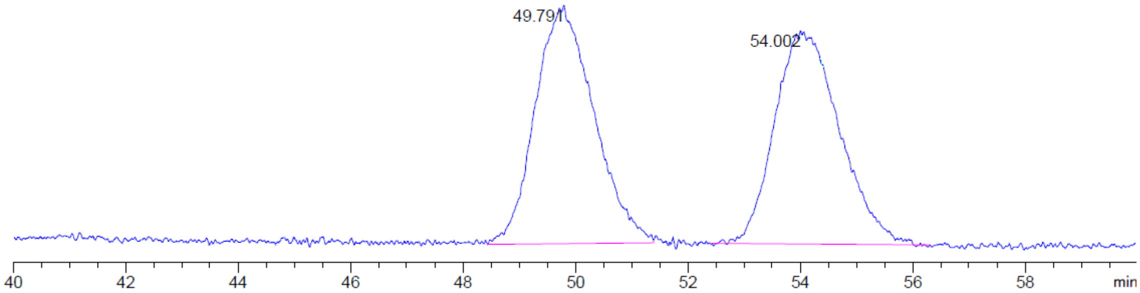

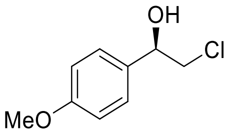

(*R*)-1n

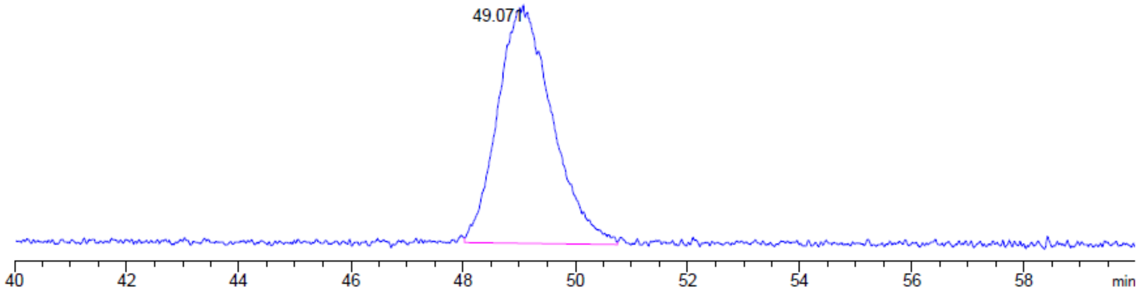

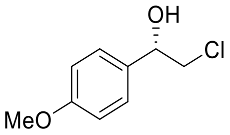

(*S*)-1n

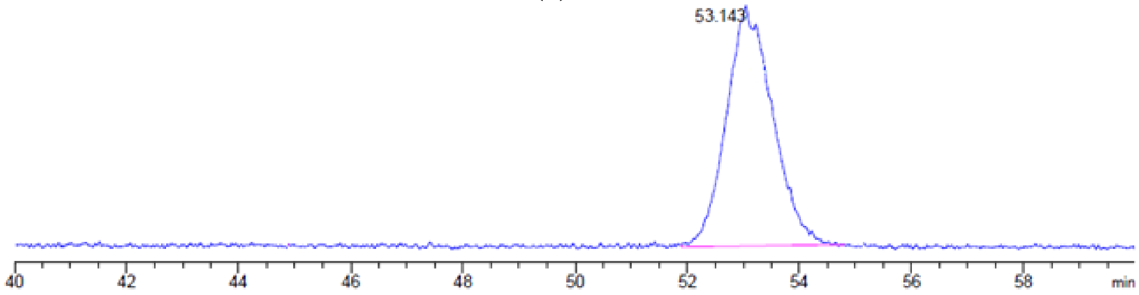

HPLC separation for both enantiomers of racemic epoxide 5a

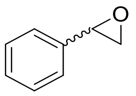

(±)-5a

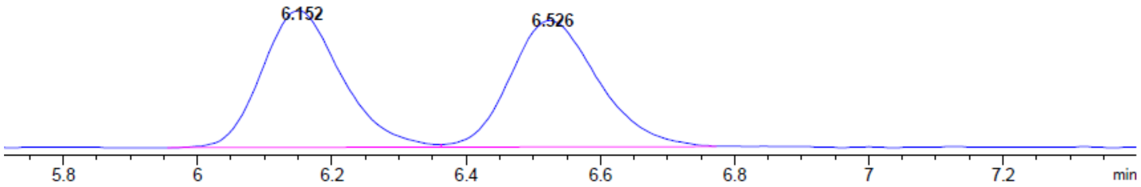

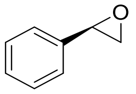

(*R*)-**5a**

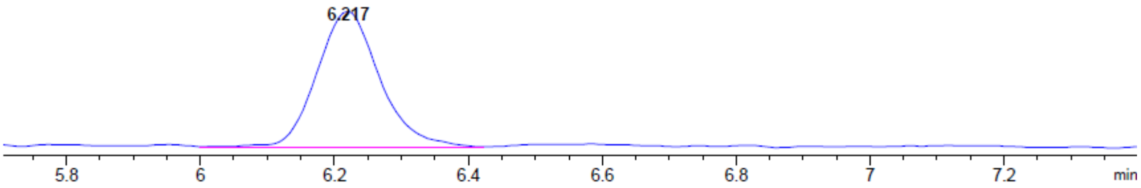

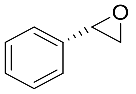

(*S*)-**5a**

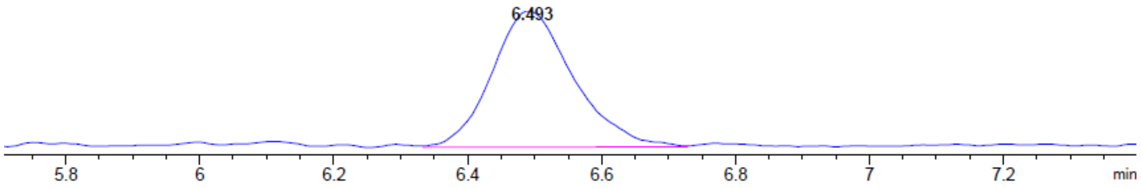

11. References

[1] Wolberg, M.; Hummel, W.; Wandrey, C.; Müller, M. Highly Regio- and Enantioselective Reduction of 3,5-Dioxocarboxylates. *Angew. Chem. Int. Ed.* **2000**, *39*, 4306-4308.

[2] Edegger, K.; Gruber, C. C.; Poessl, T. M.; Wallner, S. R.; Lavandera, I.; Faber, K.; Niehaus, F.; Eck, J.; Oehrlein, R.; Hafner, A.; Kroutil, W. Biocatalytic Deuterium- and Hydrogen-Transfer using Over-Expressed ADH-'A': Enhanced Stereoselectivity and ^{2}H-labeled Chiral Alcohols. *Chem. Commun.* **2006**, 2402-2404.

[3] Findrik, Z.; Vasic'-Rački, D.; Lütz, S.; Dauβmann, T.; Wandrey, C. Kinetic Modeling of Acetophenone Reduction Catalyzed by Alcohol Dehydrogenase from *Thermoanaerobacter* sp. *Biotechnol. Lett.* 2005, *27*, 1087-1095.

[4] Bisogno, F. R.; Lavandera, I.; Kroutil, W.; Gotor, V. Tandem Concurrent Processes: One-Pot Single-Catalyst Biohydrogen Transfer for the Simultaneous Preparation of Enantiopure Secondary Alcohols. *J. Org. Chem.* **2009**, *74*, 1730-1732.

[5] Tang, S.-Z.; Zhao, W.; Chen, T.; Liu, Y.; Zhang, X.-M.; Zhang, F.-M. A Simple and Efficient Method for the Preparation of α-Halogenated Ketones Using Iron(III) Chloride and Iron(III) Bromide as Halogen Sources with Phenyliodonium Diacetate as Oxidant. *Adv. Synth. Catal.* **2017**, *359*, 4177-4183.

[6] González-Granda, S.; Escot, L.; Lavandera, I.; Gotor-Fernández, V. Unmasking the Hidden Carbonyl Group Using Gold(I) Catalysts and Alcohol Dehydrogenases: Design of a Thermodynamically-Driven Cascade toward Optically Active Halohydrins. *ACS Catal.* **2022**, *12*, 2552-2560.

[7] Ram, R. N.; Manoj, T. P. Copper(I)-Promoted Synthesis of Chloromethyl Ketones from Trichloromethyl Carbinols. *J. Org. Chem.* **2008**, *73*, 5633-5635.

[8] Luo, Z.; Meng, Y.; Gong, X.; Wu, J.; Zhang, Y.; Ye, L. W.; Zhu, C. Facile Synthesis of α-Haloketones by Aerobic Oxidation of Olefins Using KX as Nonhazardous Halogen Source. *Chin. J. Chem.* **2020**, *38*, 173-177.

[9] Eshghi, H.; Bakavoli, M.; Ghasemzadeh, M.; Seyedi, S. M. Ionic Liquids Bis(2-N-Methylimidazoliumethyl)Ether Dichloroiodate/Dibromochlorate as an Efficient Halogenating Reagent for the Synthesis of α-Haloketones. *Res. Chem. Intermed.* **2015**, *41*, 1673-1682.

[10] Shi, X.; Zhang, L.; Yang, P.; Sun, H.; Zhang, Y.; Xie, C.; Ou-yang, Z.; Wang, M. Facile and Efficient Preparation of α-Halomethyl Ketones from α-Diazo Ketones Catalyzed by Iron(III) Halides and Silica Gel. *Tetrahedron Lett.* **2018**, *59*, 1200-1203.

[11] Kędziora, K.; Díaz-Rodríguez, A.; Lavandera, I.; Gotor-Fernández, V.; Gotor, V. Laccase/TEMPO-Mediated System for the Thermodynamically Disfavored Oxidation of 2,2-Dihalo-1-Phenylethanol Derivatives. *Green Chem.* **2014**, *16*, 2448-2453.

[12] Mangas-Sánchez, J.; Busto, E.; Gotor-Fernández, V.; Malpartida, F.; Gotor, V. Asymmetric Chemoenzymatic Synthesis of Miconazole and Econazole Enantiomers. the Importance of Chirality in Their Biological Evaluation. *J. Org. Chem.* **2011**, *76*, 2115-2122.

[13] Besse, P.; Renard, M. F.; Veschambre, H. Chemoenzymatic Synthesis of Chiral Epoxides. Preparation of 4-Phenyl-2,3-Epoxybutane and 1-Phenyl-1,2-Epoxypropane. *Tetrahedron Asymmetry* **1994**, *5*, 1249-1268.

[14] Haak, R. M.; Berthiol, F.; Jerphagnon, T.; Gayet, A. J. A.; Tarabiono, C.; Postema, C. P.; Ritleng, V.; Pfeffer, M.; Janssen, D. B.; Minnaard, A. J.; Feringa, B. L.; De Vries, J. G. Dynamic Kinetic Resolution of Racemic β-Haloalcohols: Direct Access to Enantioenriched Epoxides. *J. Am. Chem. Soc.* **2008**, *130*, 13508-13509.

[15] Blasius, C. K.; Vasilenko, V.; Gade, L. H. Ultrafast Iron-Catalyzed Reduction of Functionalized Ketones: Highly Enantioselective Synthesis of Halohydrines, Oxaheterocycles, and Aminoalcohols. *Angew. Chem. Int. Ed.* **2018**, *57*, 10231-10235.

[16] Gaviña, P.; Lavernia, N. L.; Mestres, R.; Miranda, M. A. Deacetalization by Photoinduced Electron Transfer with a Pyrylium Salt: Effect of Limiting the Amounts of Water, Oxygen and Sensitizer. *Tetrahedron* **1996**, *52*, 4911-4916.

[17] Lee, S. H.; Kim, S.; Yun, M. H.; Lee, Y. S.; Cho, S. N.; Oh, T.; Kim, P. Synthesis and Antitubercular Activity of Monocyclic Nitroimidazoles: Insights from Econazole. *Bioorg. Med. Chem. Lett.* **2011**, *21*, 1515-1518.

[18] Träft, A.; Bogár, K.; Warner, M.; Bäckvall, J. E. Highly Efficient Route for Enantioselective Preparation of Chlorohydrins via Dynamic Kinetic Resolution. *Org. Lett.* **2008**, *10*, 4807-4810.

[19] Wang, J.; Peng, Y.; Xu, J.; Wu, Q. Deracemization of Racemic Alcohols Combining Photooxidation and Biocatalytic Reduction. *Org. Biomol. Chem.* **2022**, *20*, 7765-7769.

[20] Rudzka, A.; Antos, N.; Reiter, T.; Kroutil, W.; Borowiecki, P. One-Pot Sequential Two-Step Photo-Biocatalytic Deracemization of Sec-Alcohols Combining Photocatalytic Oxidation and Bioreduction. *ACS Catal.* **2024**, *14*, 1808-1823.

[21] Romero, N. A.; Nicewicz, D. A. Organic Photoredox Catalysis. *Chem. Rev.* **2016**, *116*, 10075-10166.

[22] Aquino, A. M. The Synthesis and Photochemistry of Anthraquinone-2,6-disulfonyl-beta-cyclodextrin. *Dissertations, Theses, and Masters Projects.* **1989**, William & Mary. Paper 1539625517.

[23] Ravelli, D.; Fagnoni, M.; Fukuyama, T.; Nishikawa, T.; Ryu, I. Site-Selective C-H Functionalization by Decatungstate Anion Photocatalysis: Synergistic Control by Polar and Steric Effects Expands the Reaction Scope. *ACS Catal.* **2018**, *8*, 701-713.

[24] Natarajan, P.; König, B. Excited-State 2,3-Dichloro-5,6-Dicyano-1,4-Benzoquinone (DDQ*) Initiated Organic Synthetic Transformations under Visible-Light Irradiation. *Eur. J. Org. Chem.* **2021**, *2021*, 2145-2161.

[25] Tambe, S. D.; Cho, E. J. Organophotocatalytic Oxidation of Alcohols to Carboxylic Acids. *Bull. Korean Chem. Soc.* **2022**, *43*, 1226-1230.

[26] Peng, Y.; Li, D.; Fan, J.; Xu, W.; Xu, J.; Yu, H.; Lin, X.; Wu, Q. Enantiocomplementary C–H Bond Hydroxylation Combining Photo-Catalysis and Whole-Cell Biocatalysis in a One-Pot Cascade Process. *Eur. J. Org. Chem.* **2020**, *2020*, 821-825.

12. NMR spectra

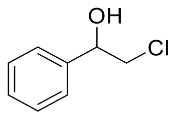

1a

^{1}H-NMR (300 MHz, $CDCl_3$)

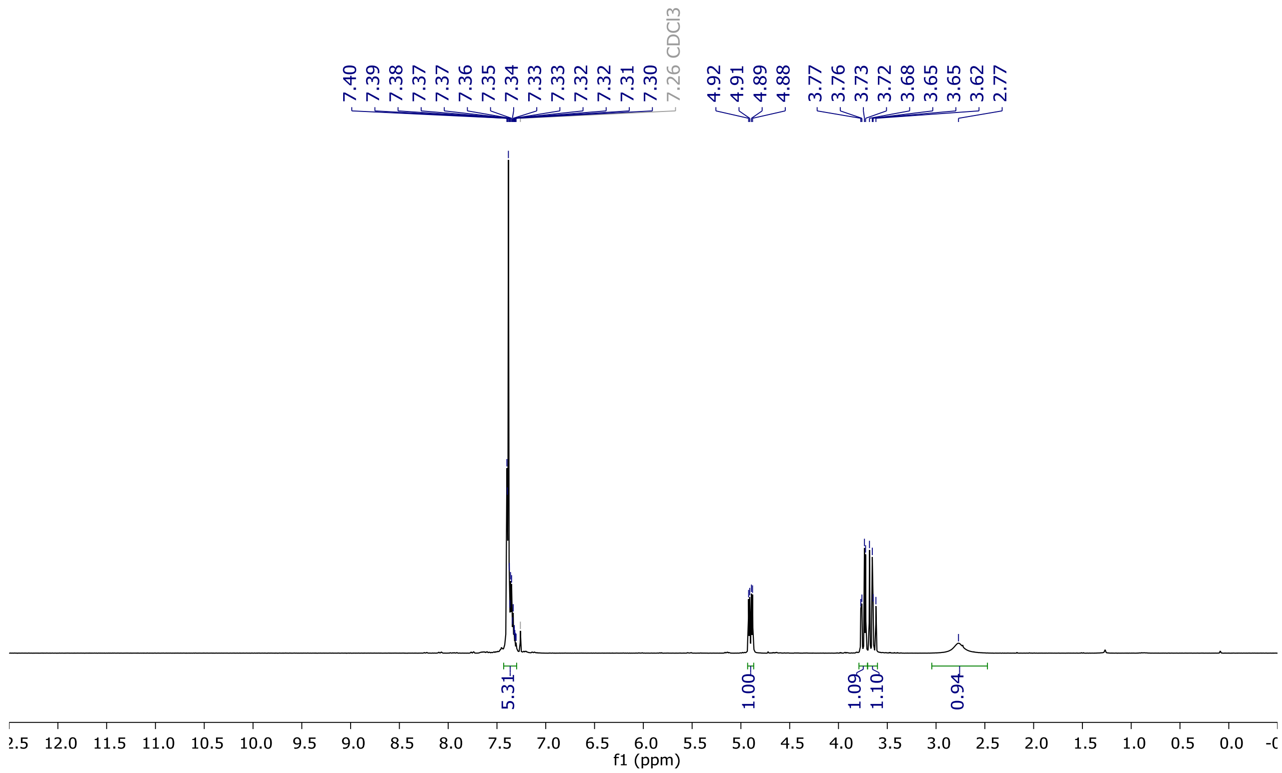

^{13}C-NMR (75 MHz, $CDCl_3$)

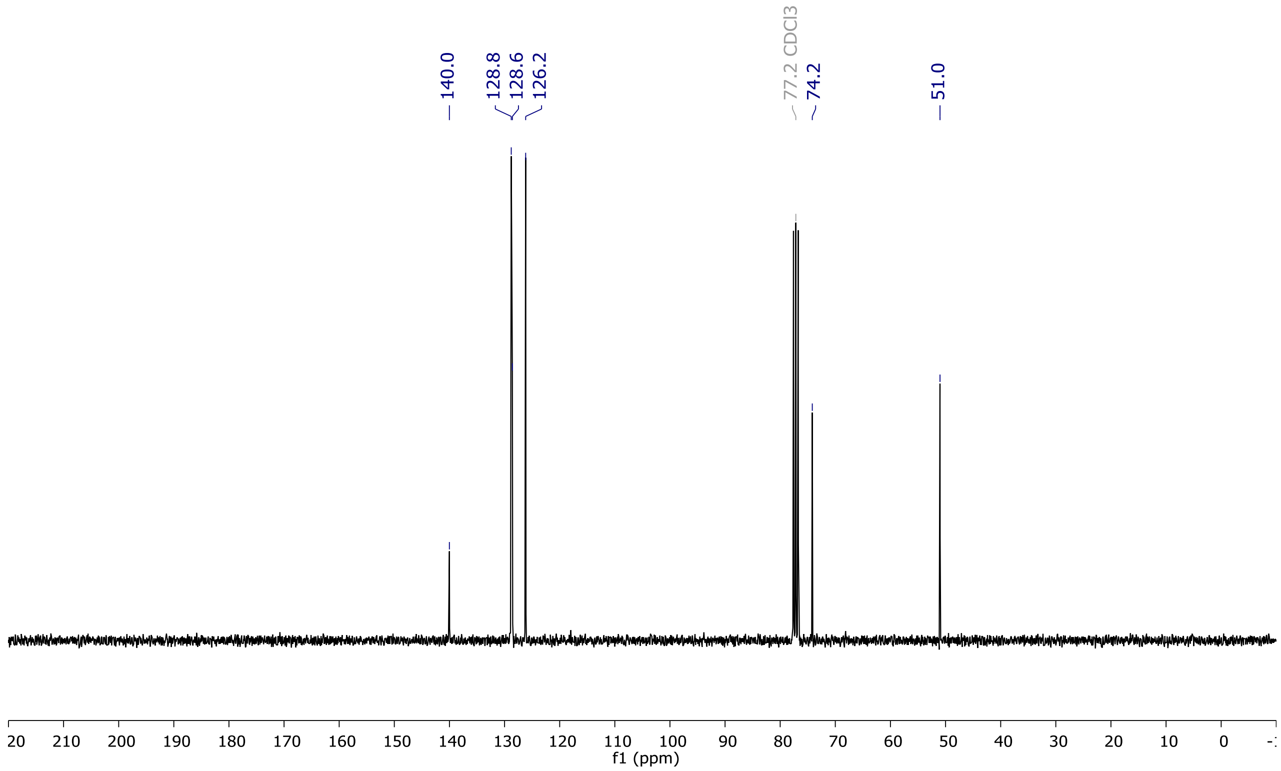

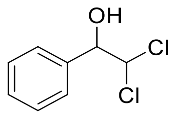

1b

^{1}H-NMR (300 MHz, $CDCl_3$)

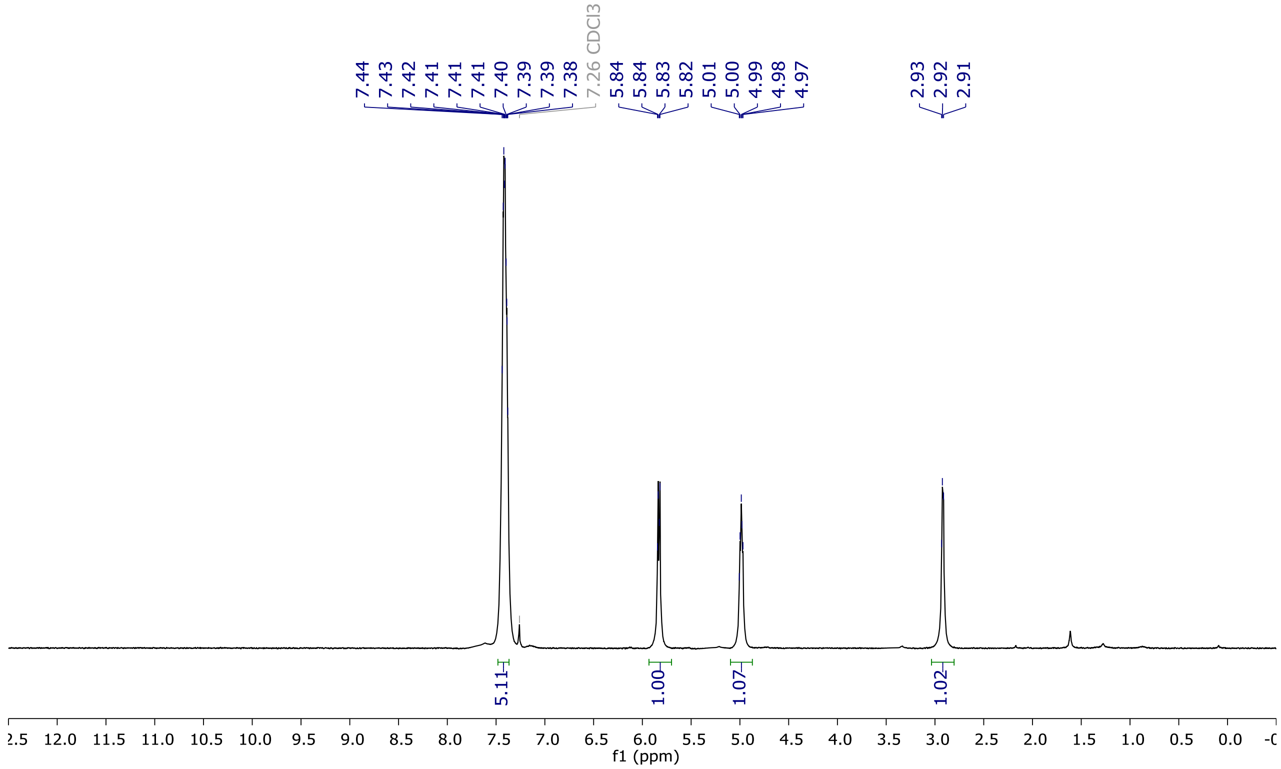

^{13}C-NMR (75 MHz, $CDCl_3$)

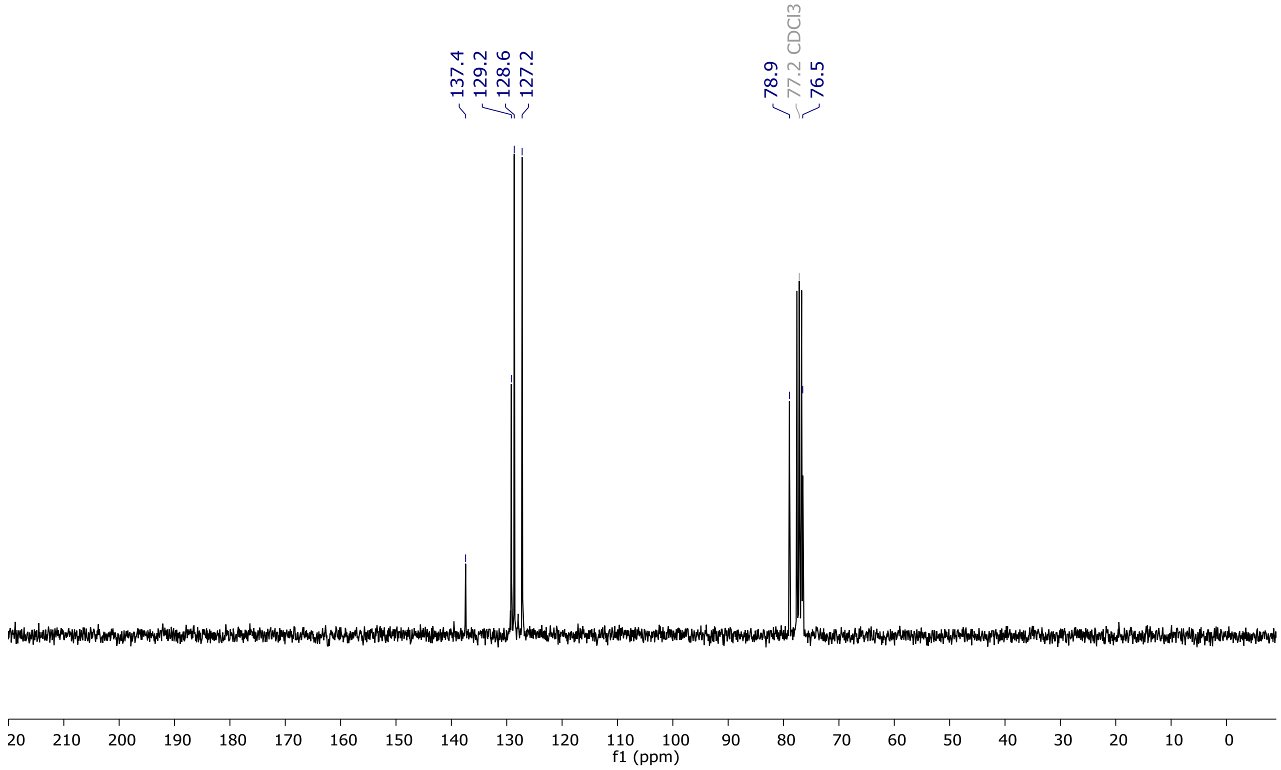

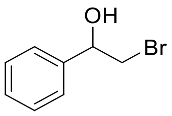

1c

^{1}H-NMR (300 MHz, $CDCl_3$)

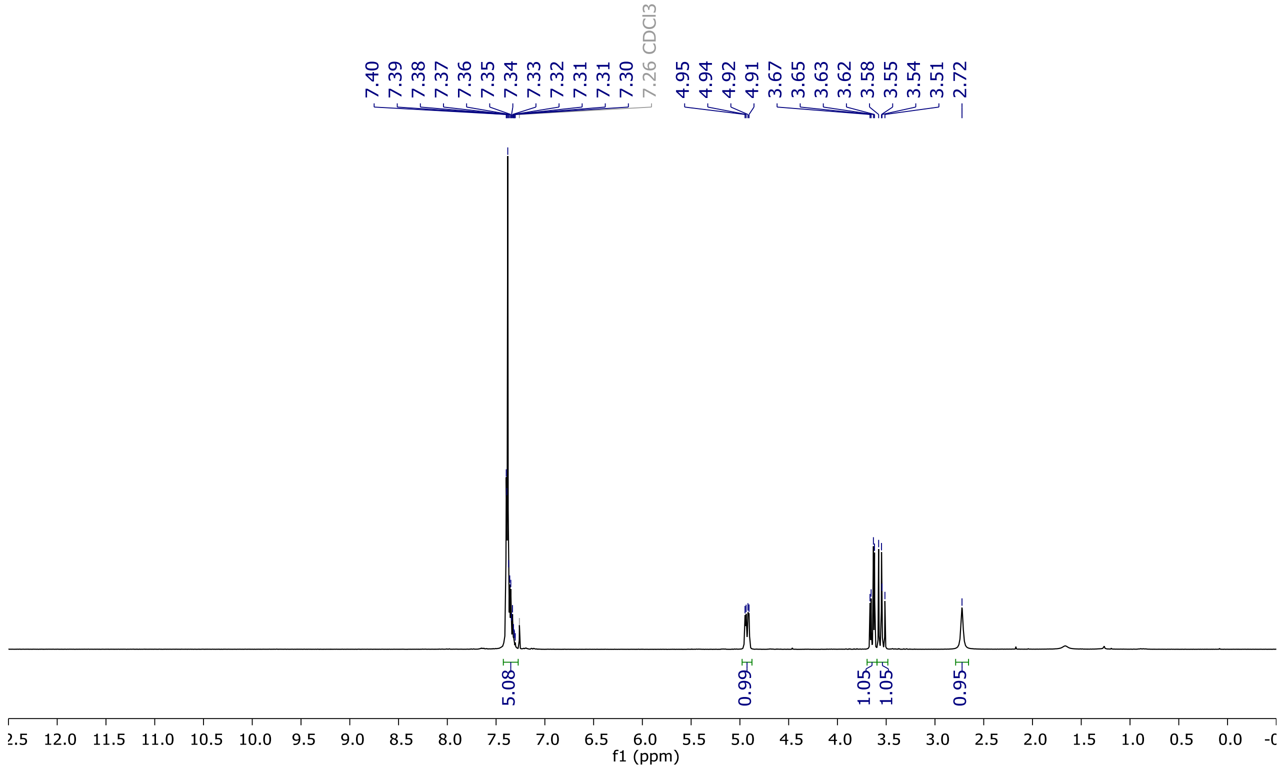

^{13}C-NMR (75 MHz, $CDCl_3$)

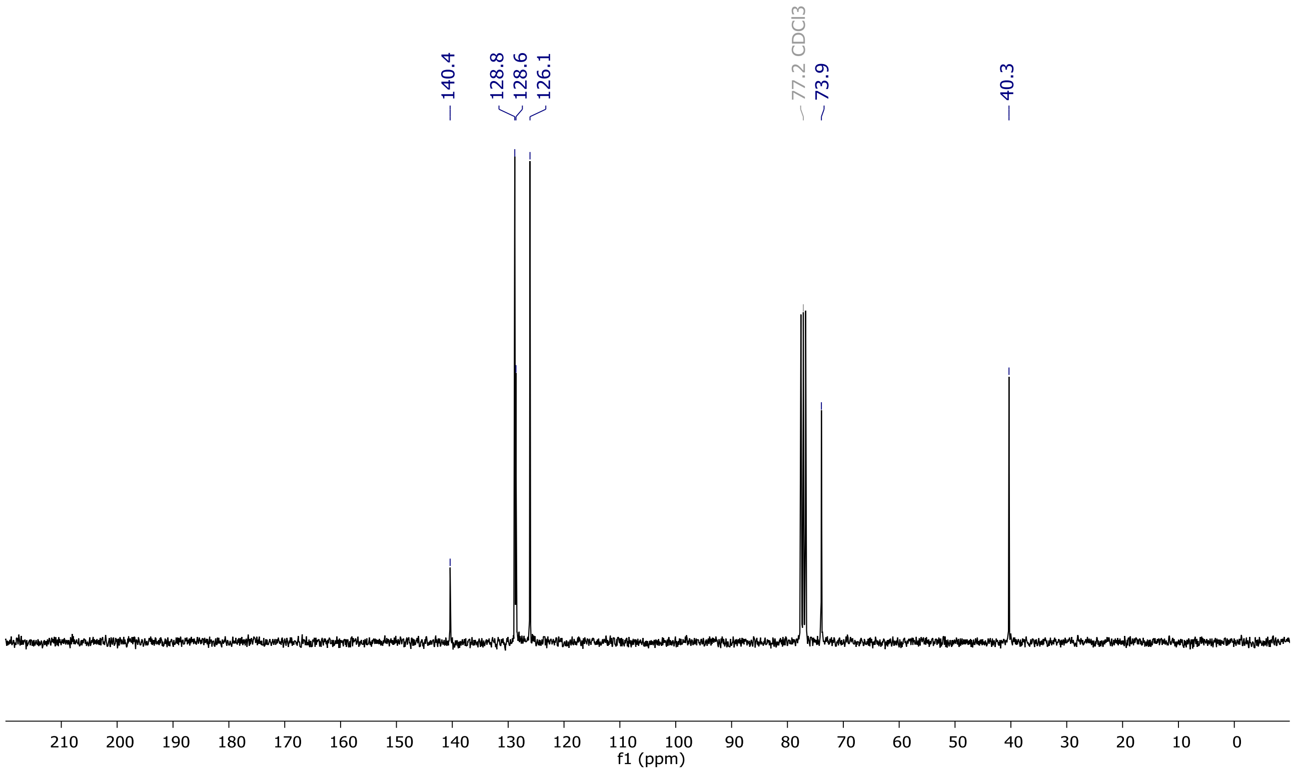

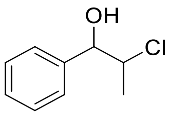

1d

^{1}H-NMR (300 MHz, $CDCl_3$)

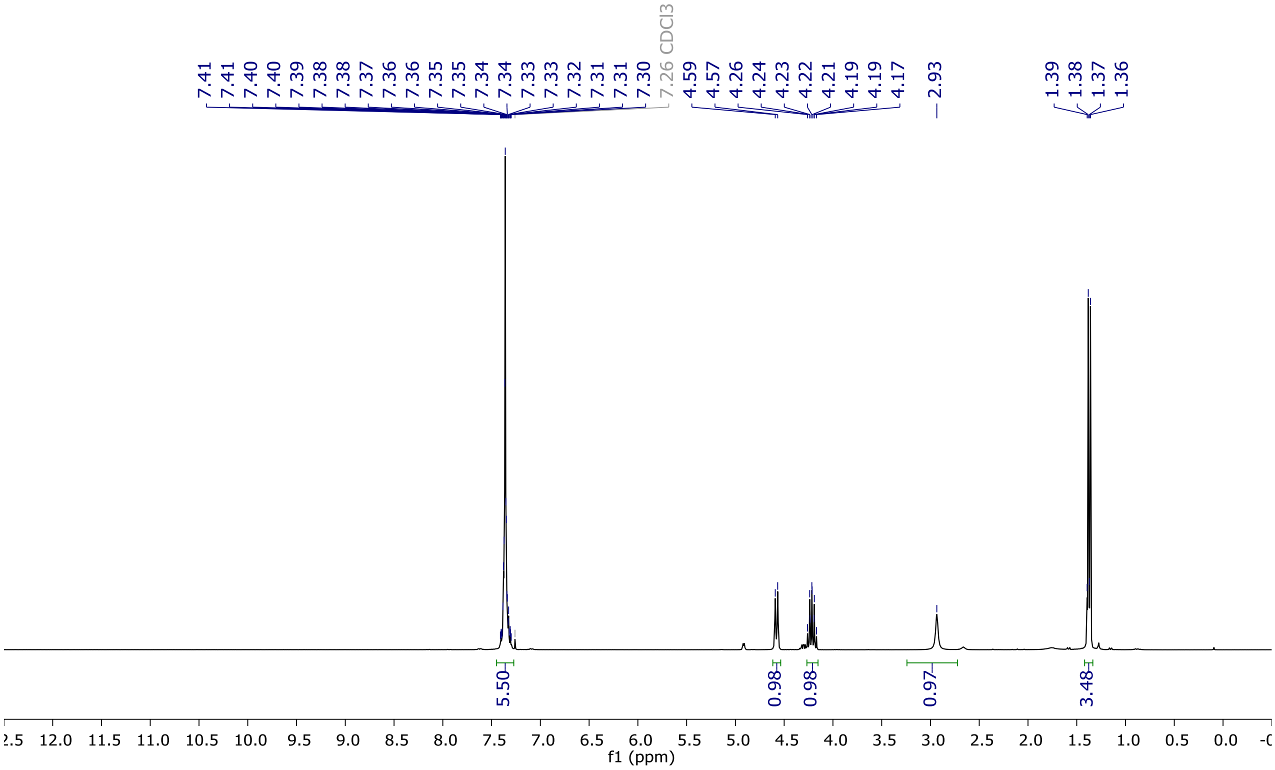

^{13}C-NMR (75 MHz, $CDCl_3$)

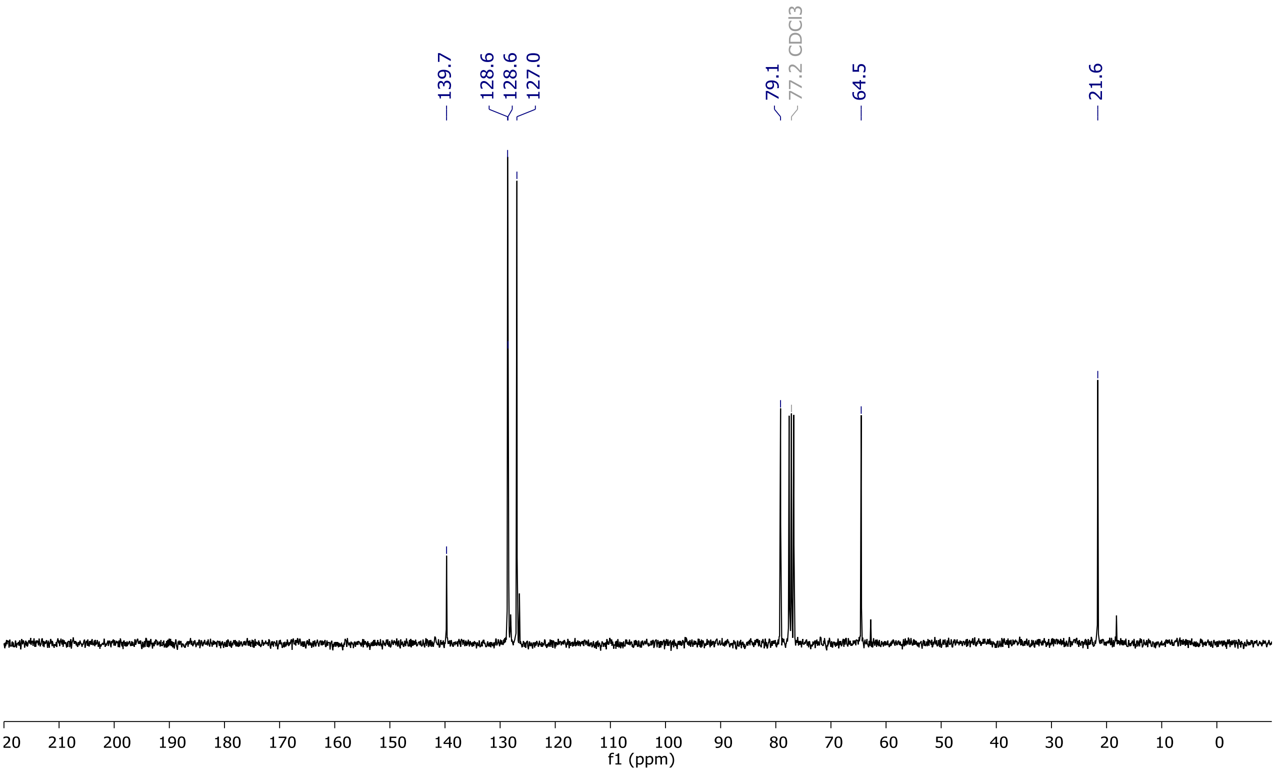

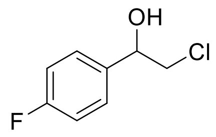

1e

^{1}H-NMR (300 MHz, $CDCl_3$)

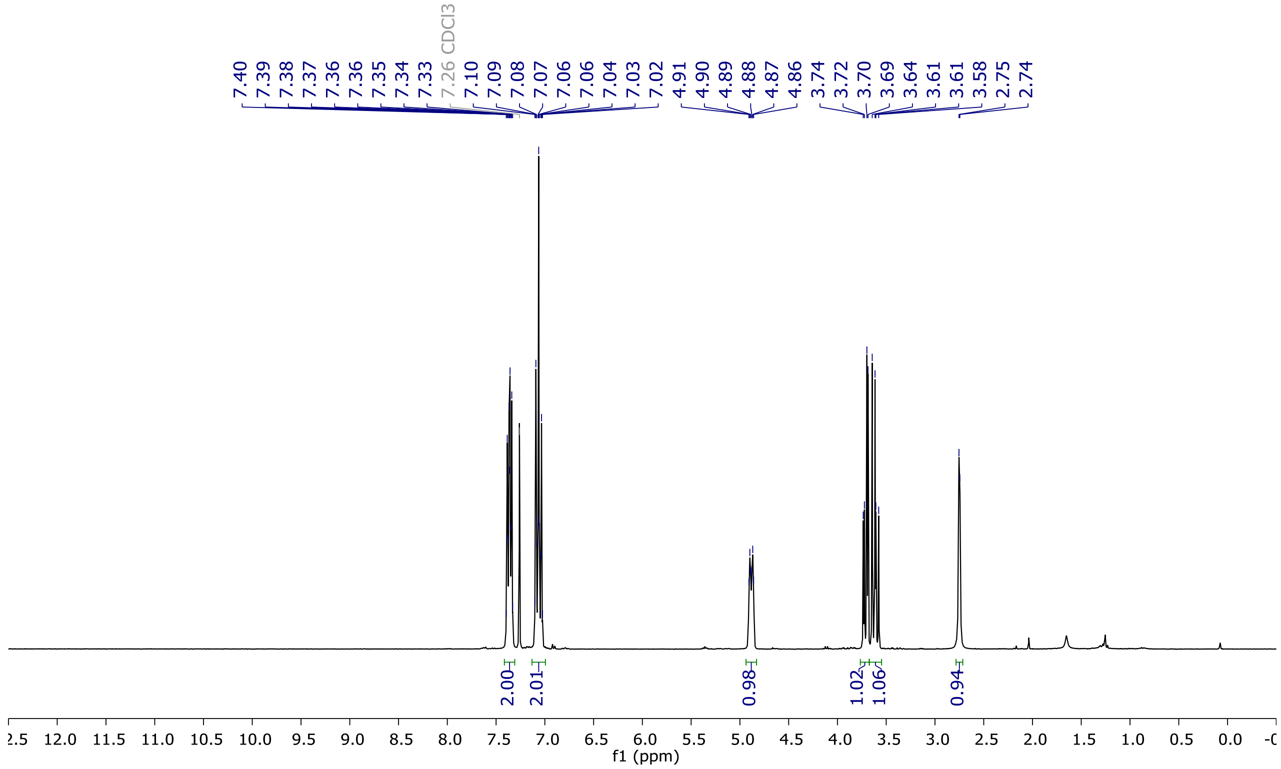

^{13}C-NMR (75 MHz, $CDCl_3$)

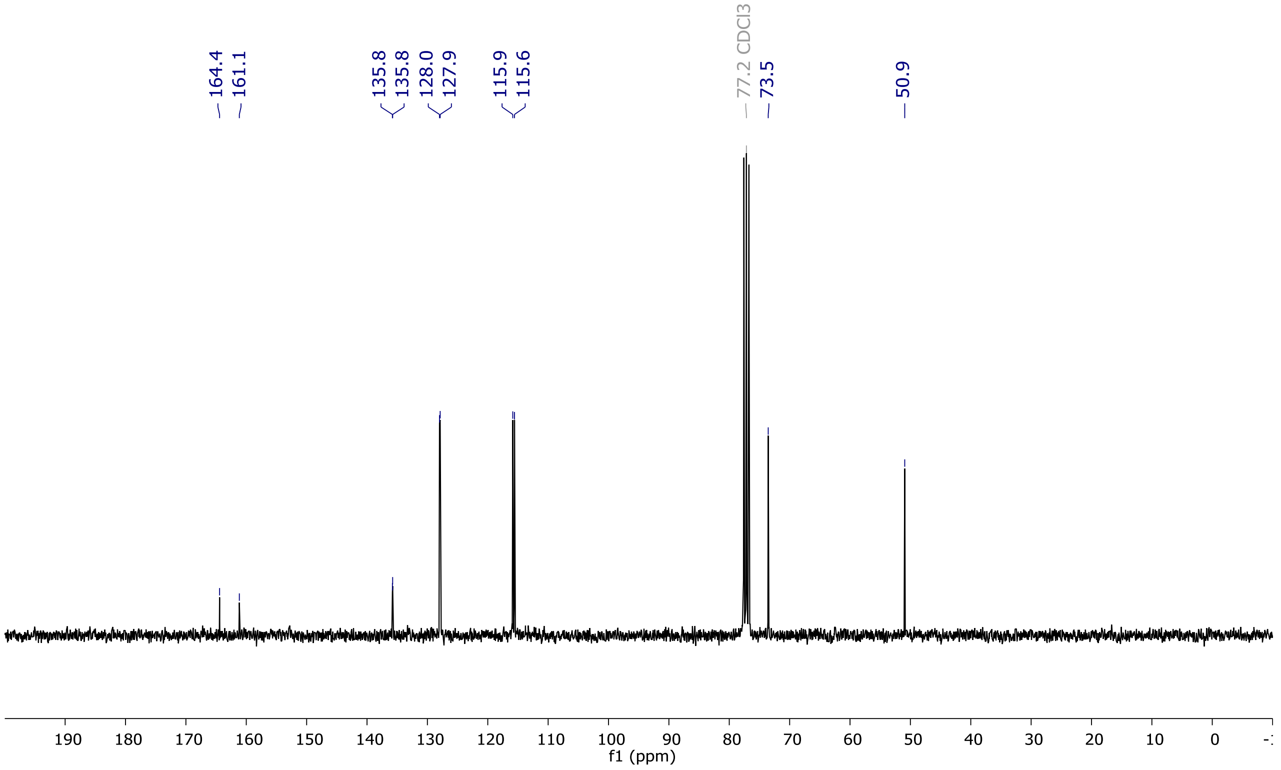

^{19}F{^{1}H}-NMR (282 MHz, $CDCl_3$)

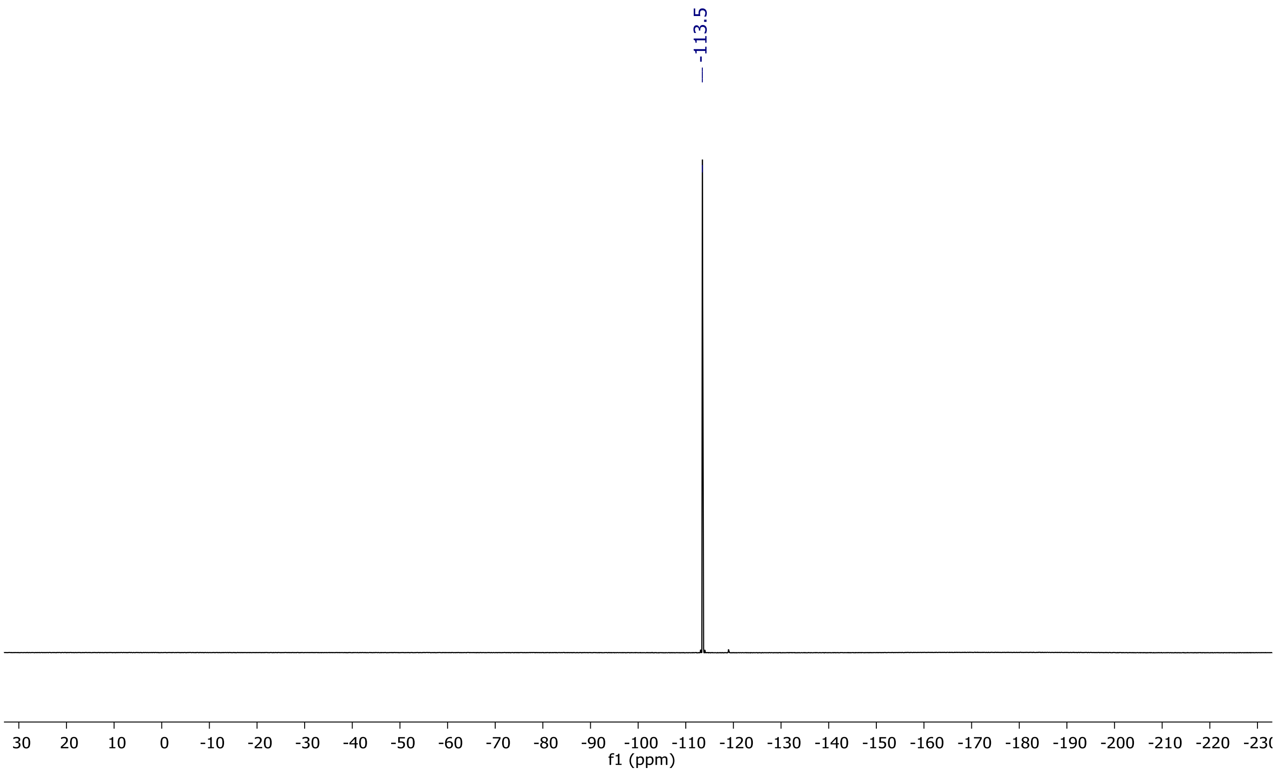

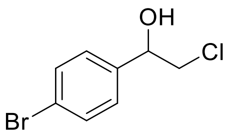

1f

^{1}H-NMR (300 MHz, $CDCl_3$)

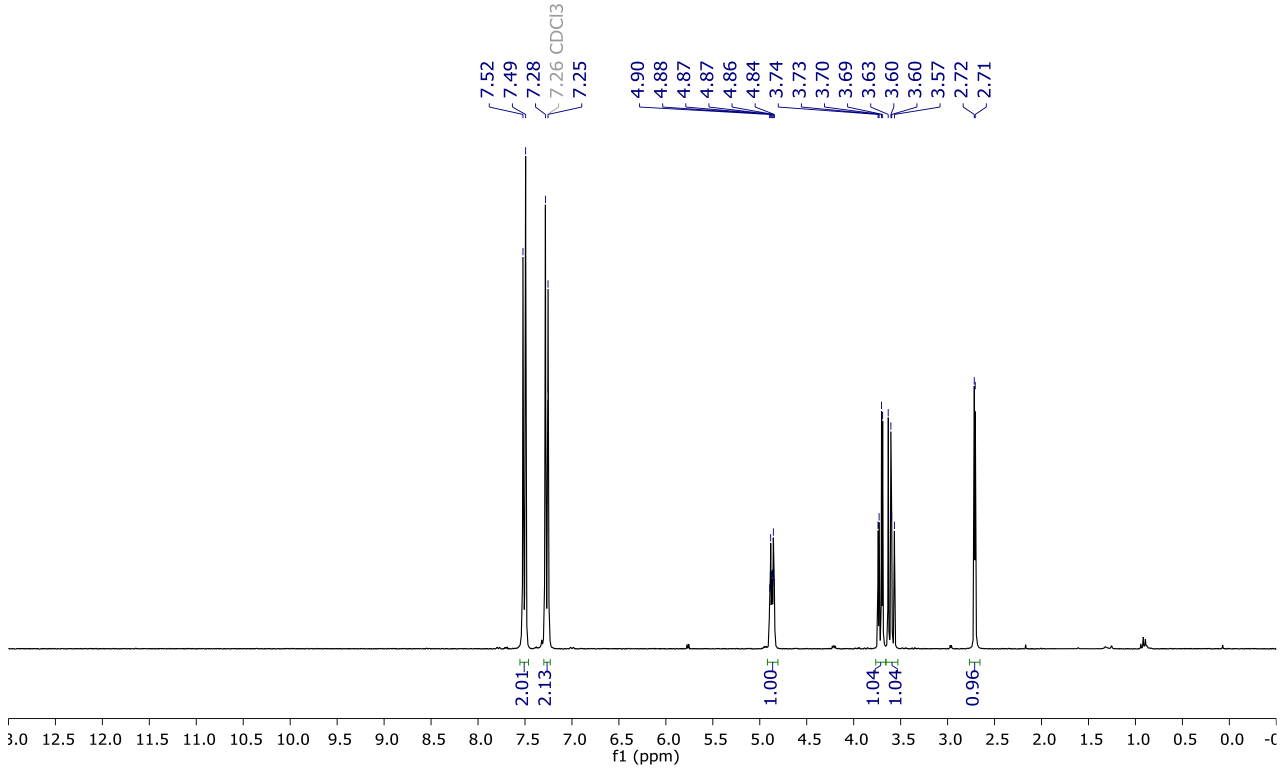

^{13}C-NMR (75 MHz, $CDCl_3$)

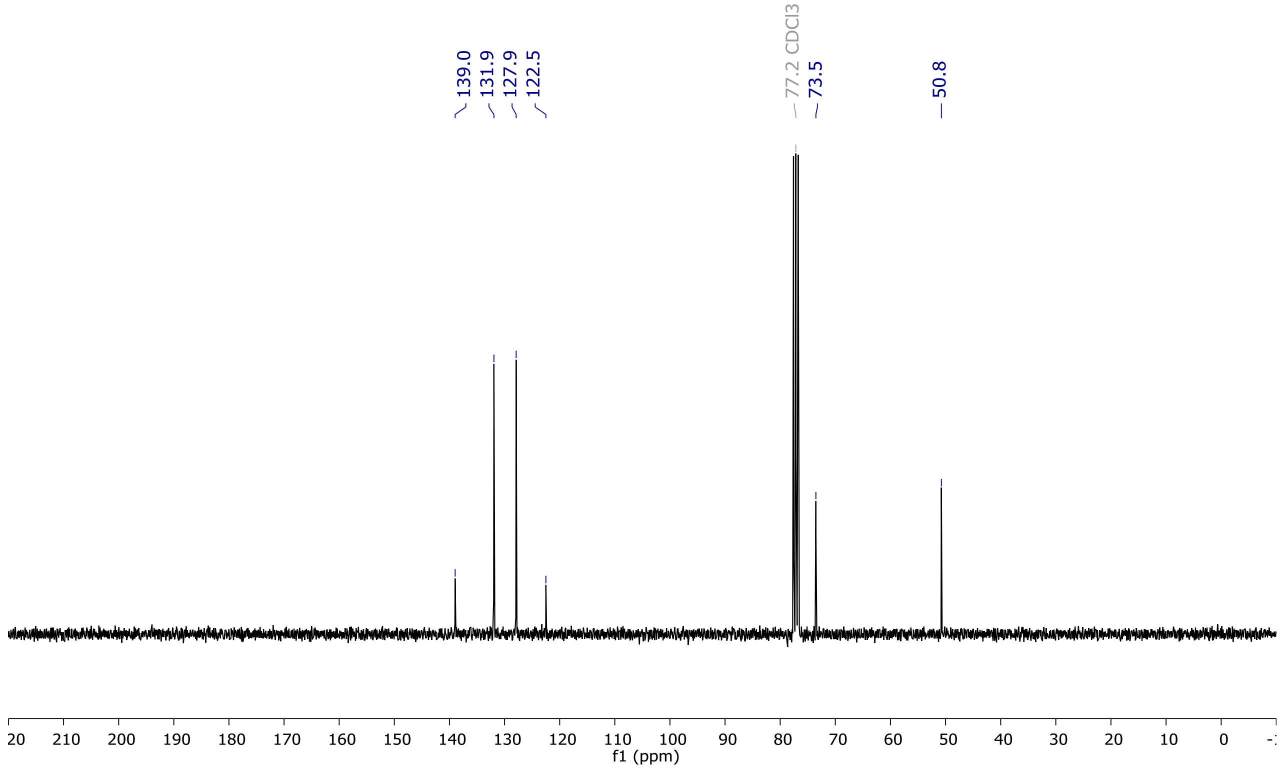

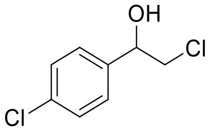

1g

^{1}H-NMR (300 MHz, $CDCl_3$)

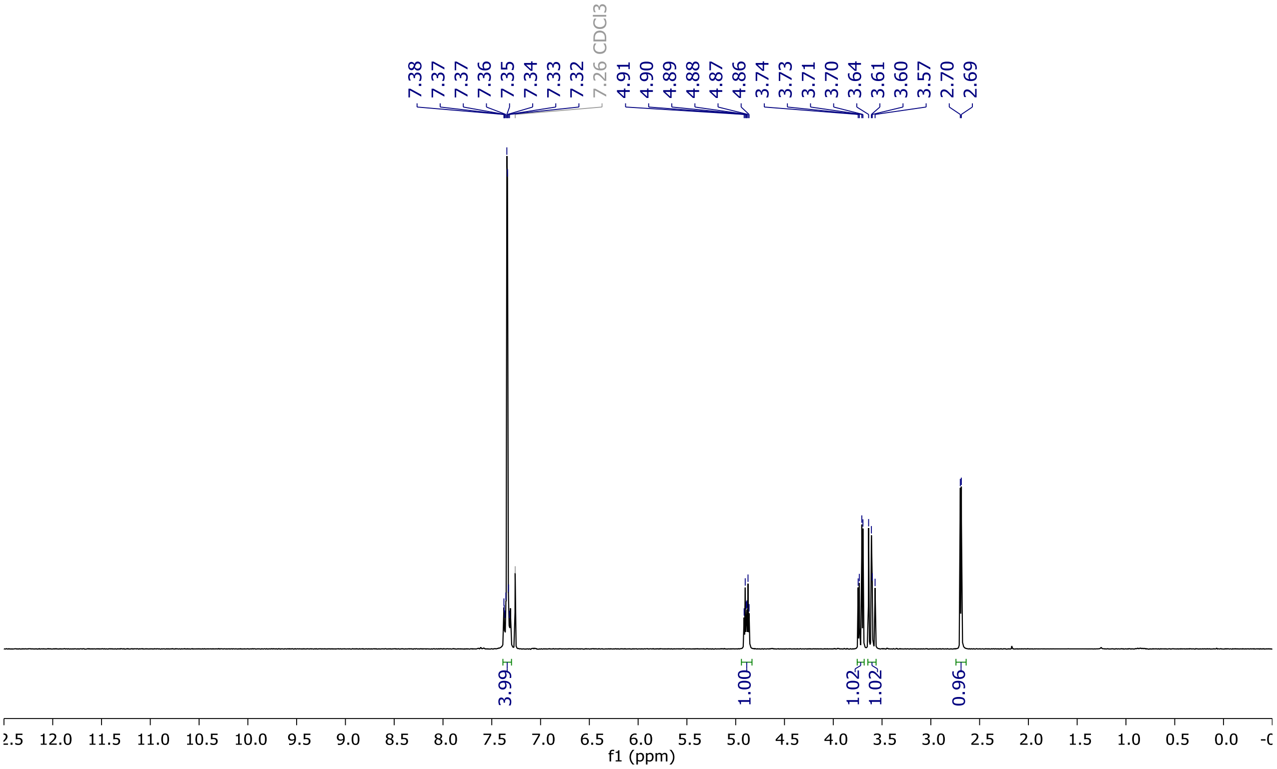

^{13}C-NMR (75 MHz, $CDCl_3$)

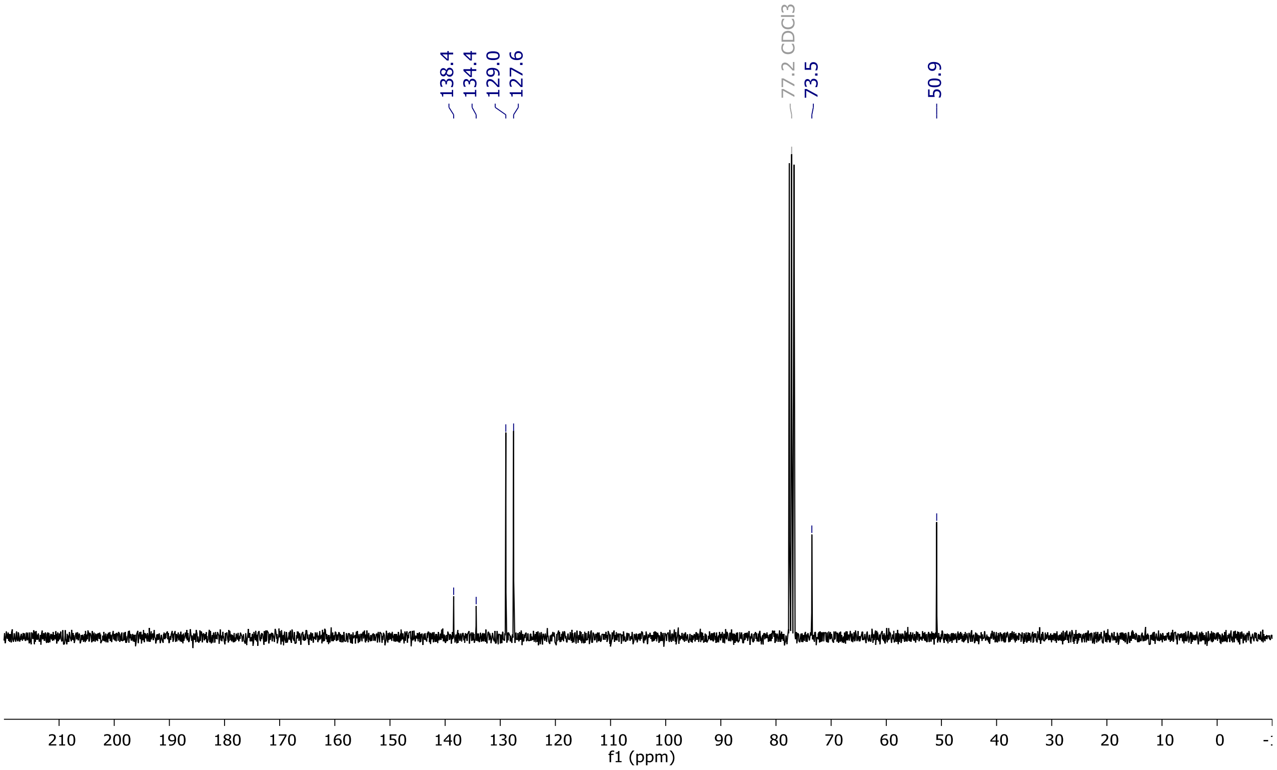

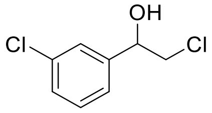

1h

^{1}H-NMR (300 MHz, $CDCl_3$)

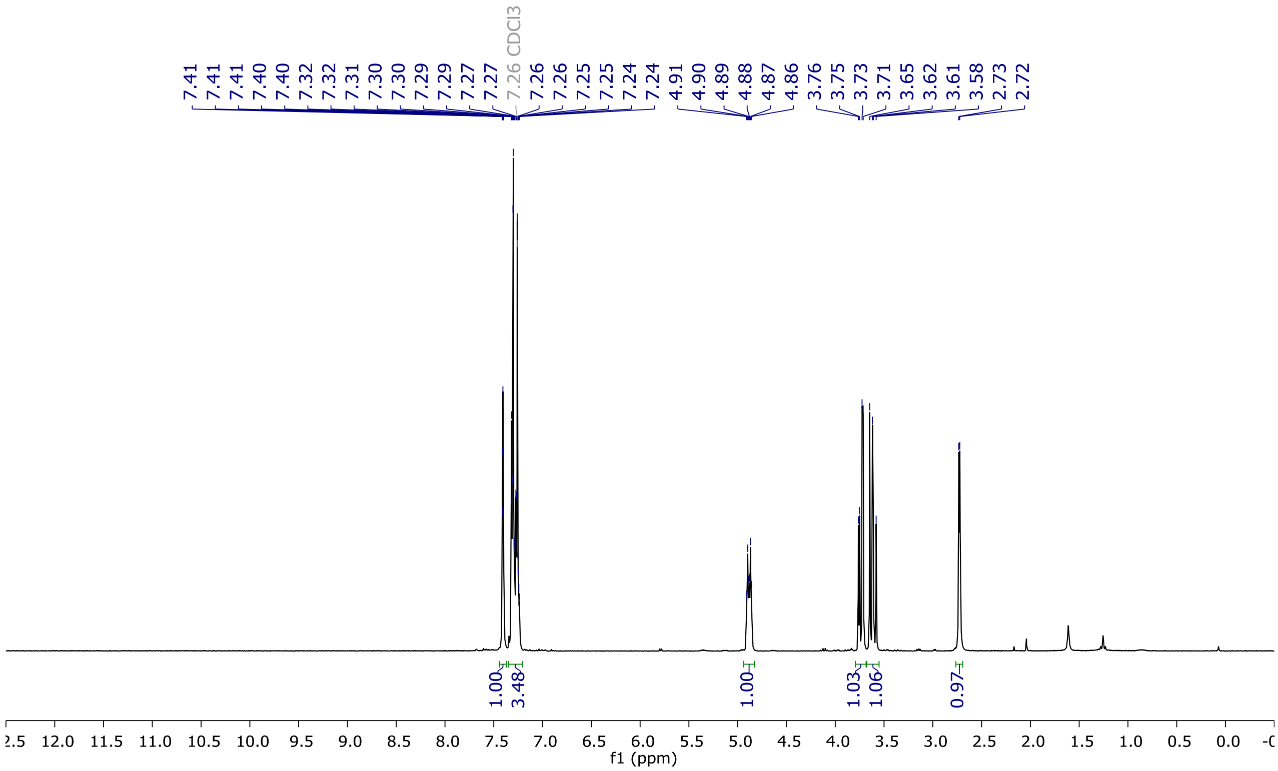

^{13}C-NMR (75 MHz, $CDCl_3$)

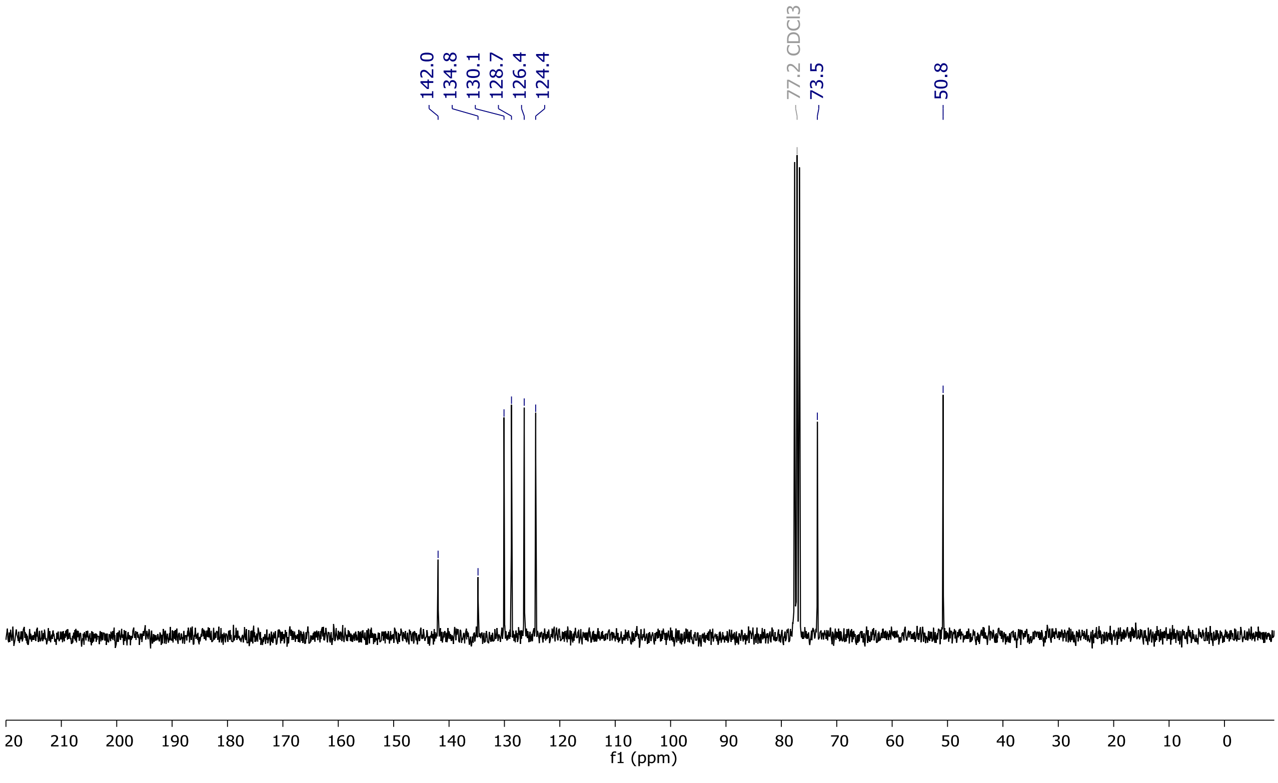

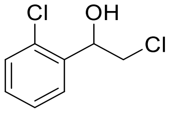

1i

^{1}H-NMR (300 MHz, $CDCl_3$)

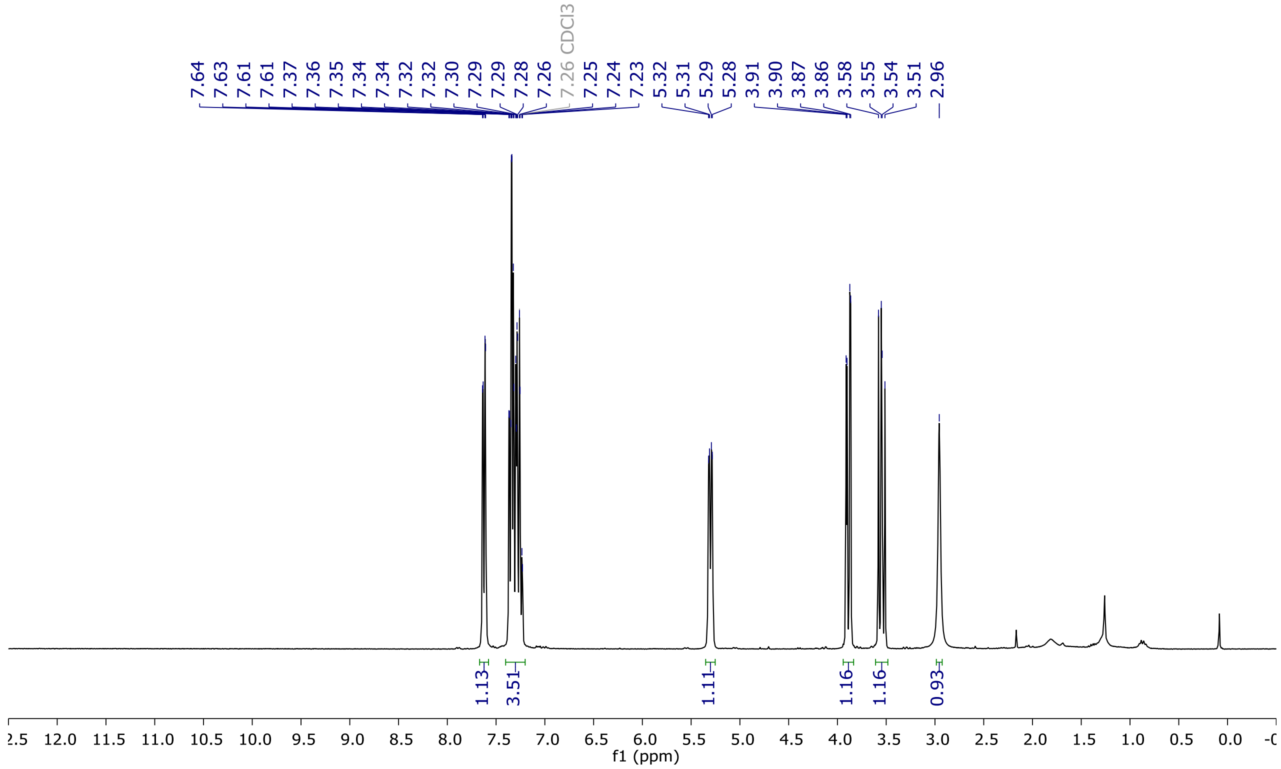

^{13}C-NMR (75 MHz, $CDCl_3$)

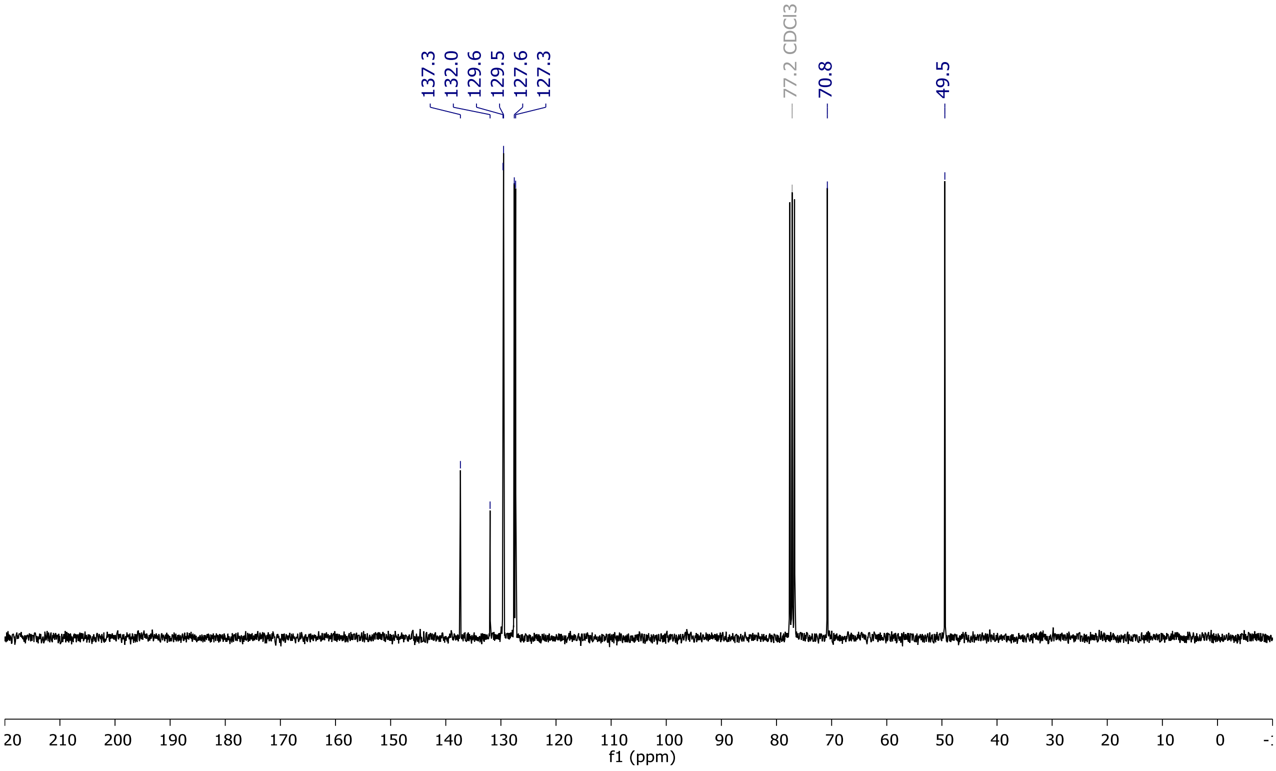

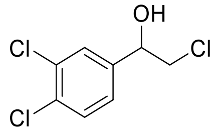

1j

^{1}H-NMR (300 MHz, $CDCl_3$)

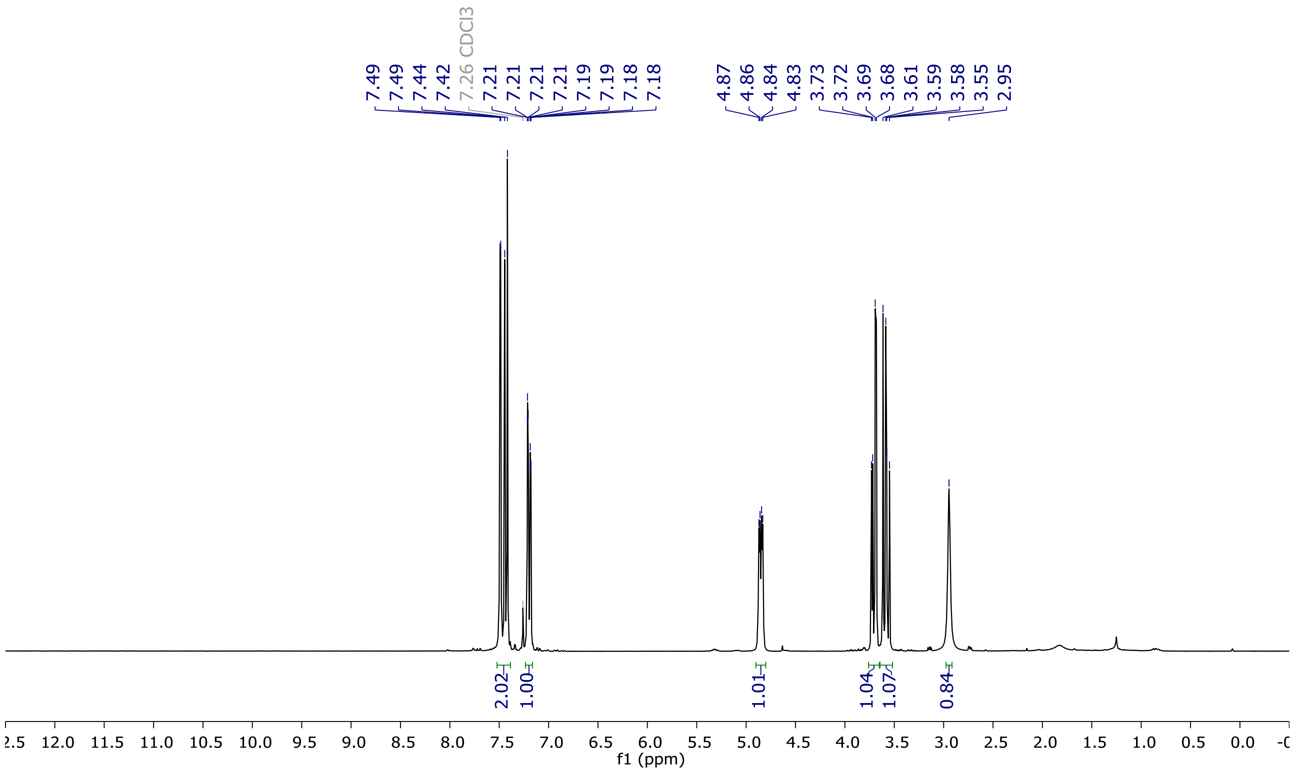

^{13}C-NMR (75 MHz, $CDCl_3$)

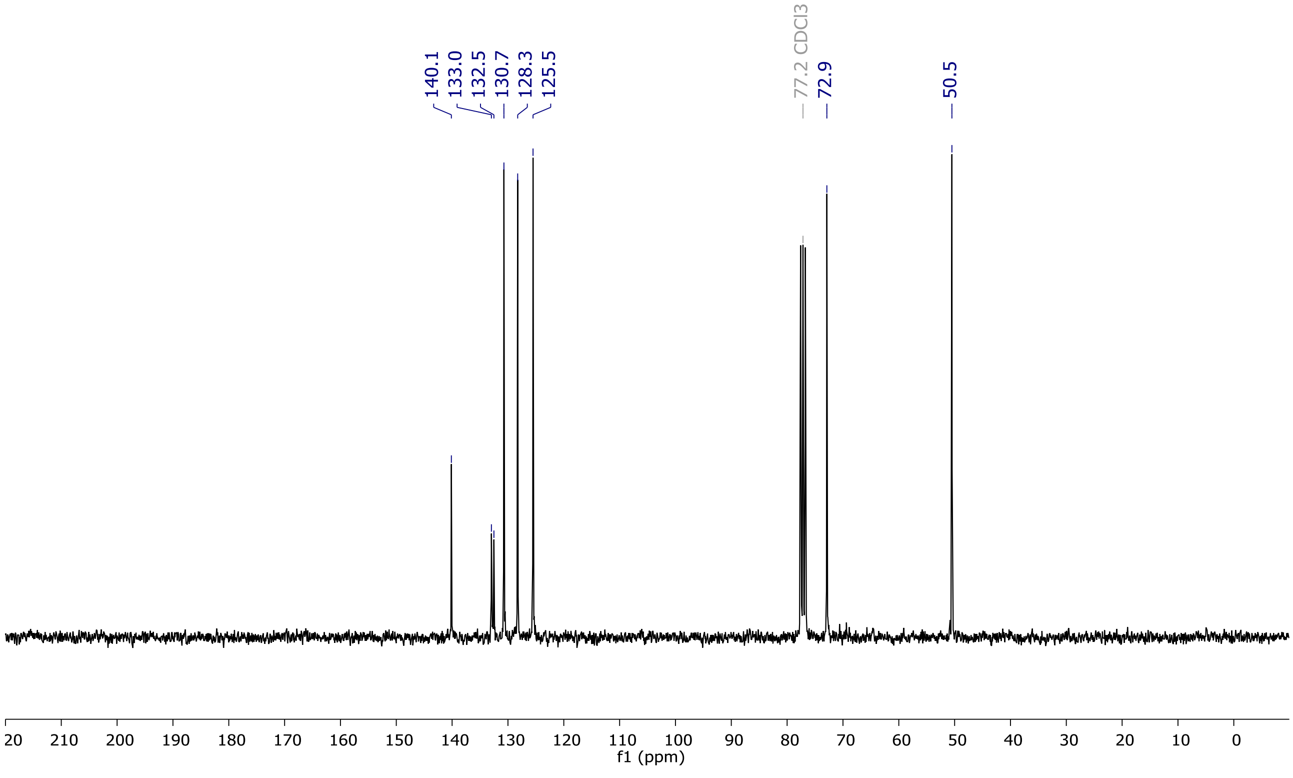

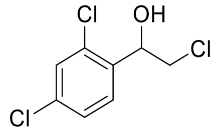

1k

^{1}H-NMR (300 MHz, $CDCl_3$)

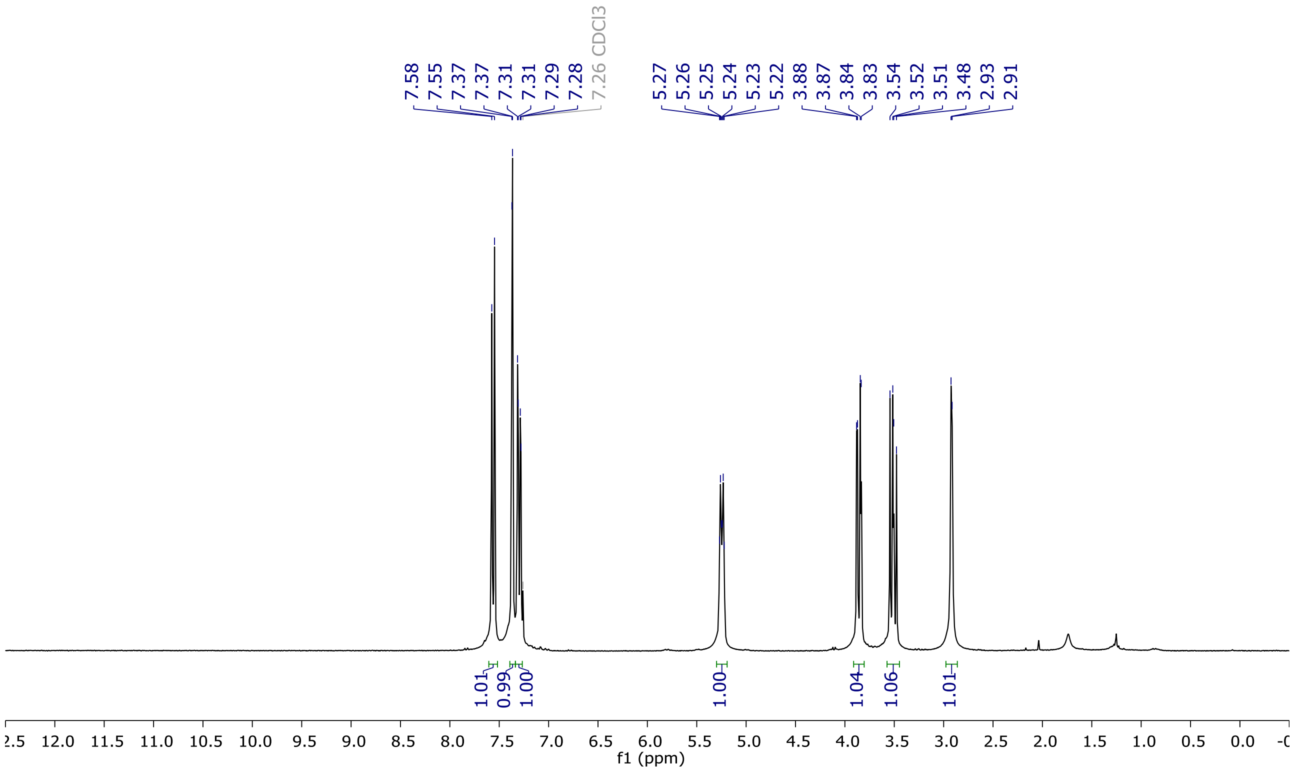

^{13}C-NMR (75 MHz, $CDCl_3$)

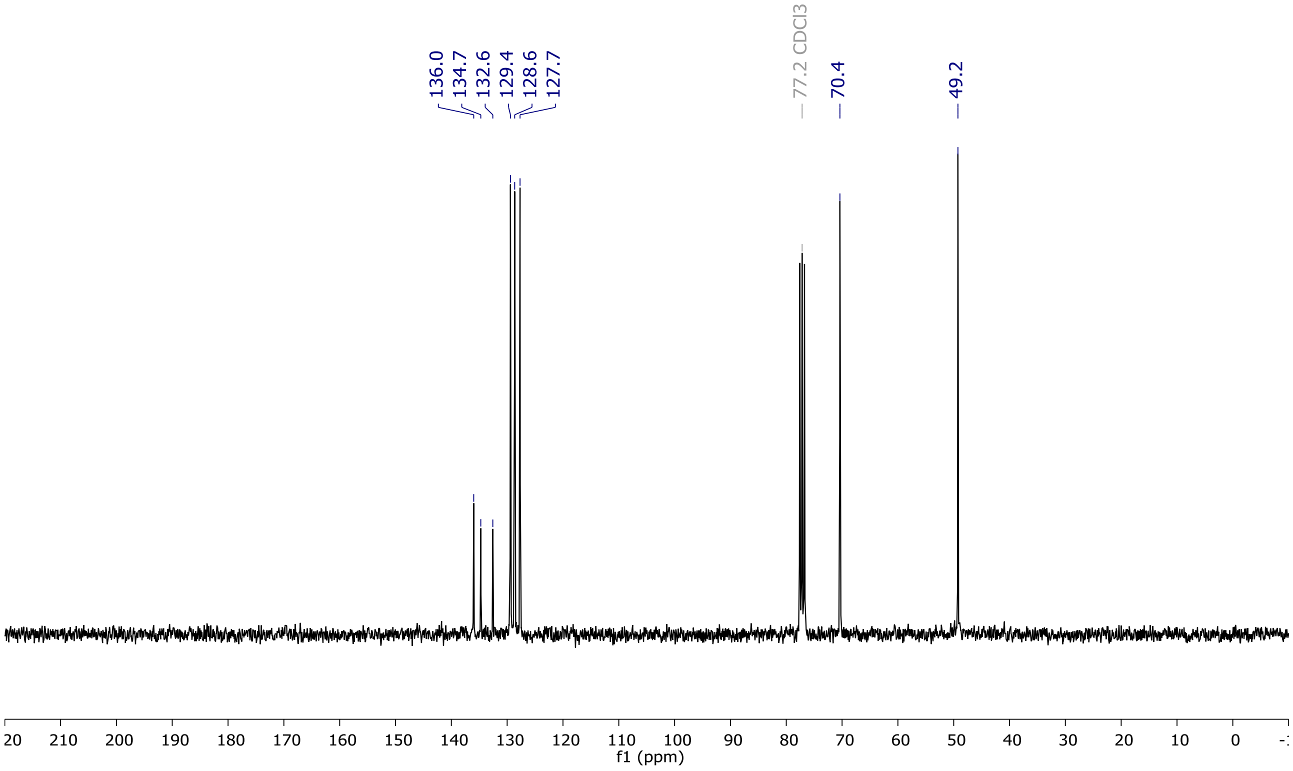

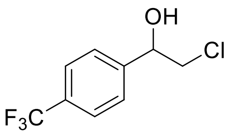

1l

^{1}H-NMR (300 MHz, $CDCl_3$)

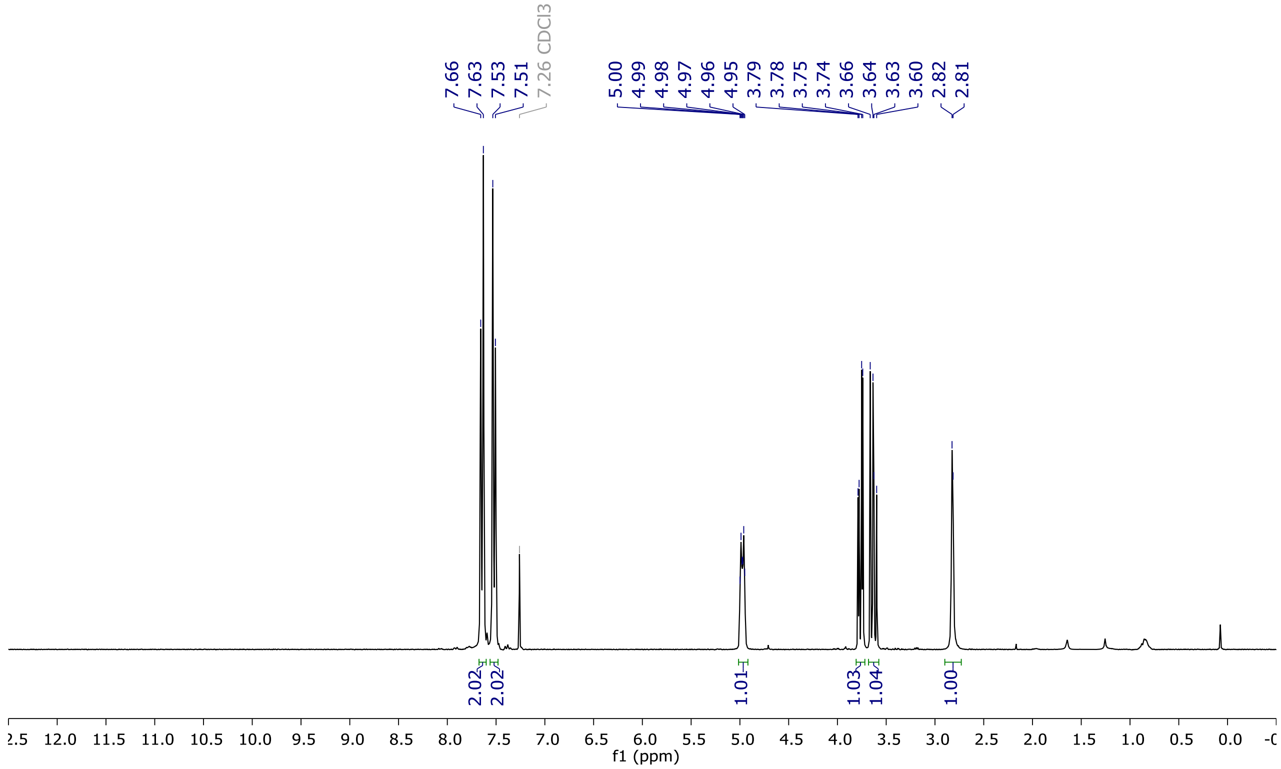

^{13}C-NMR (75 MHz, $CDCl_3$)

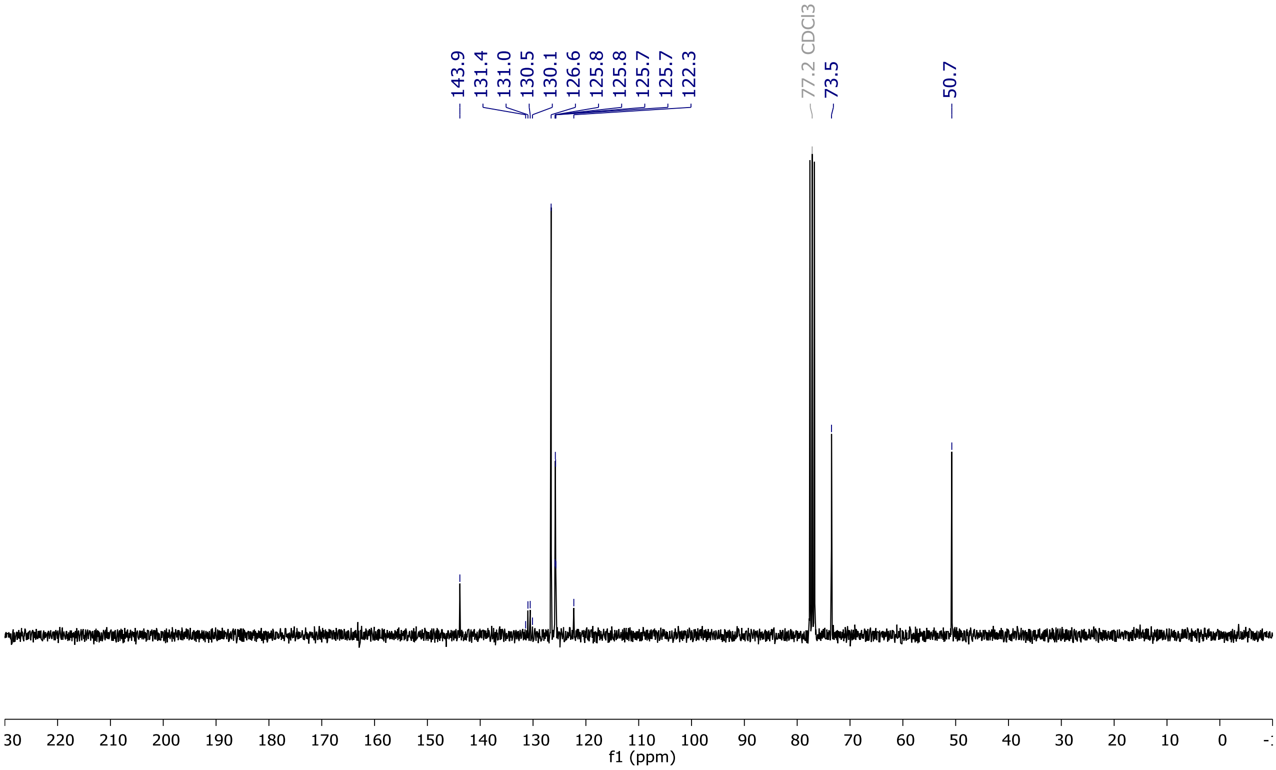

^{19}F{^{1}H}-NMR (282 MHz, $CDCl_3$)

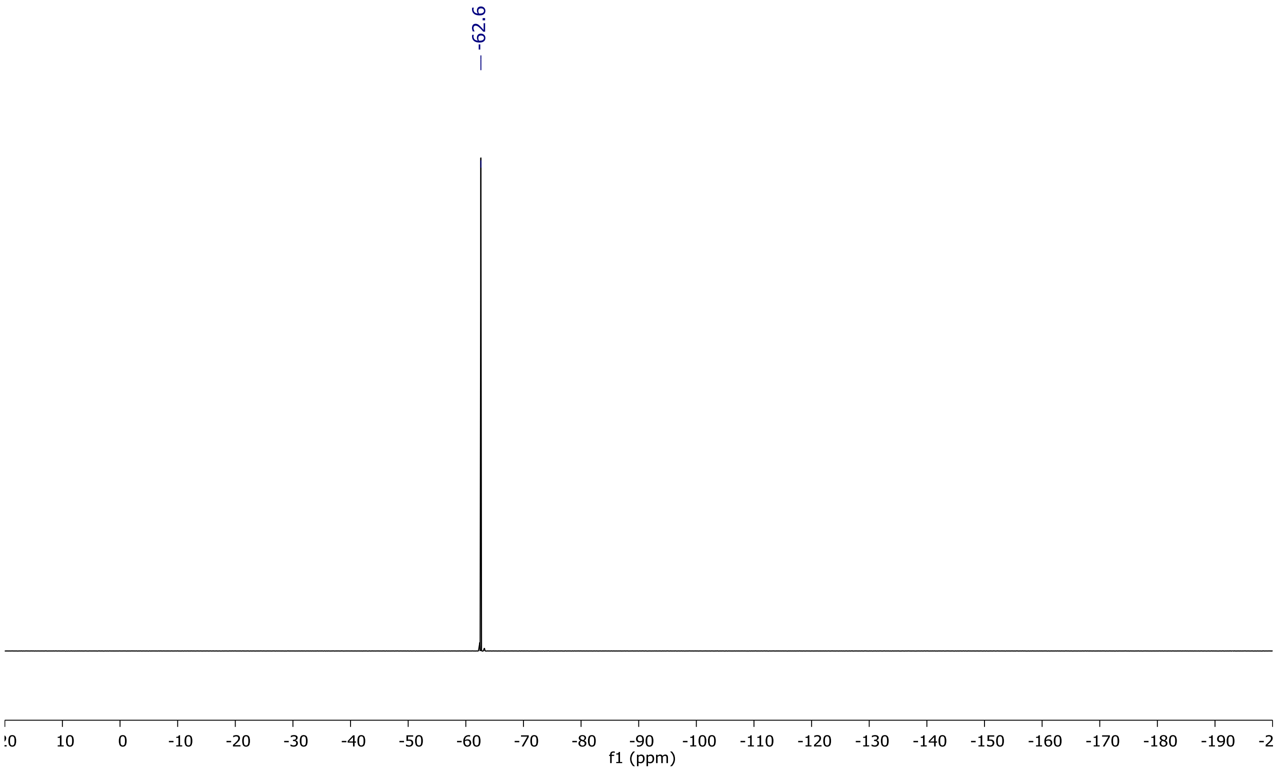

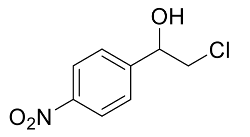

1m

^{1}H-NMR (300 MHz, $CDCl_3$)

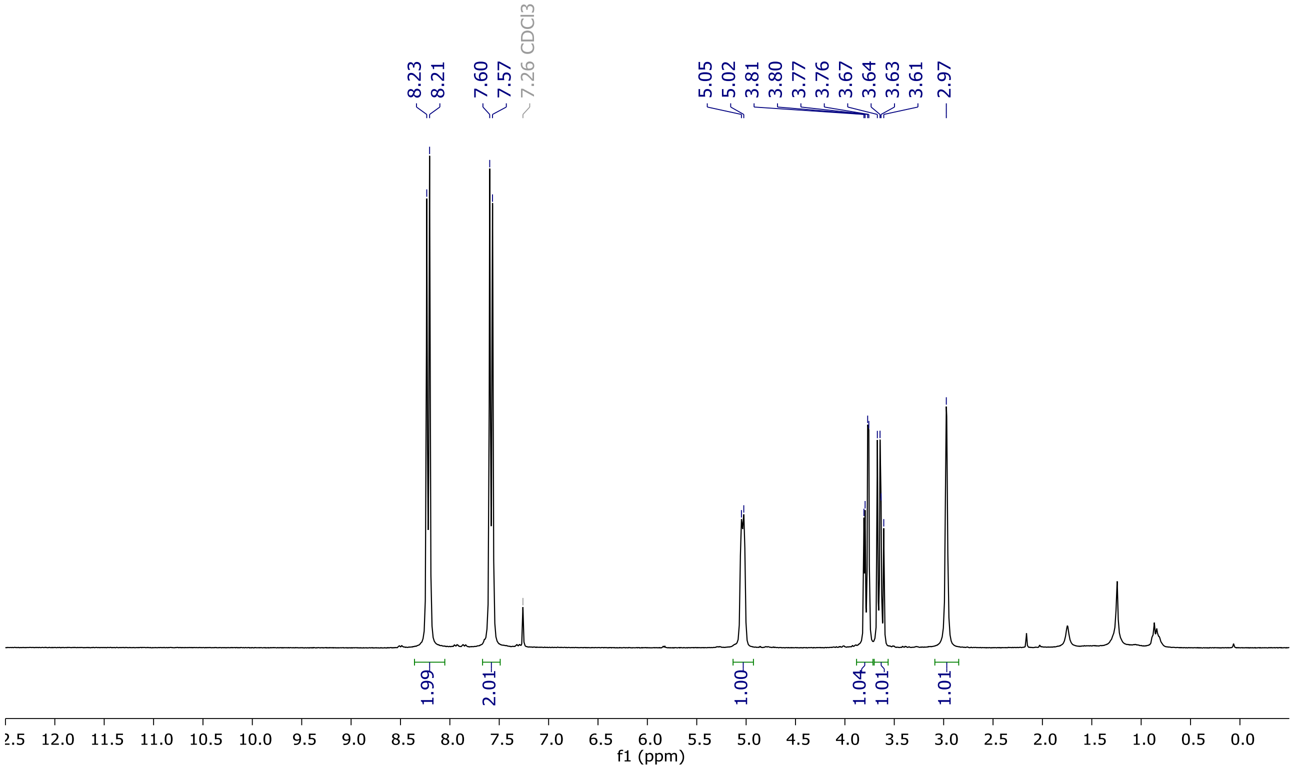

^{13}C-NMR (75 MHz, $CDCl_3$)

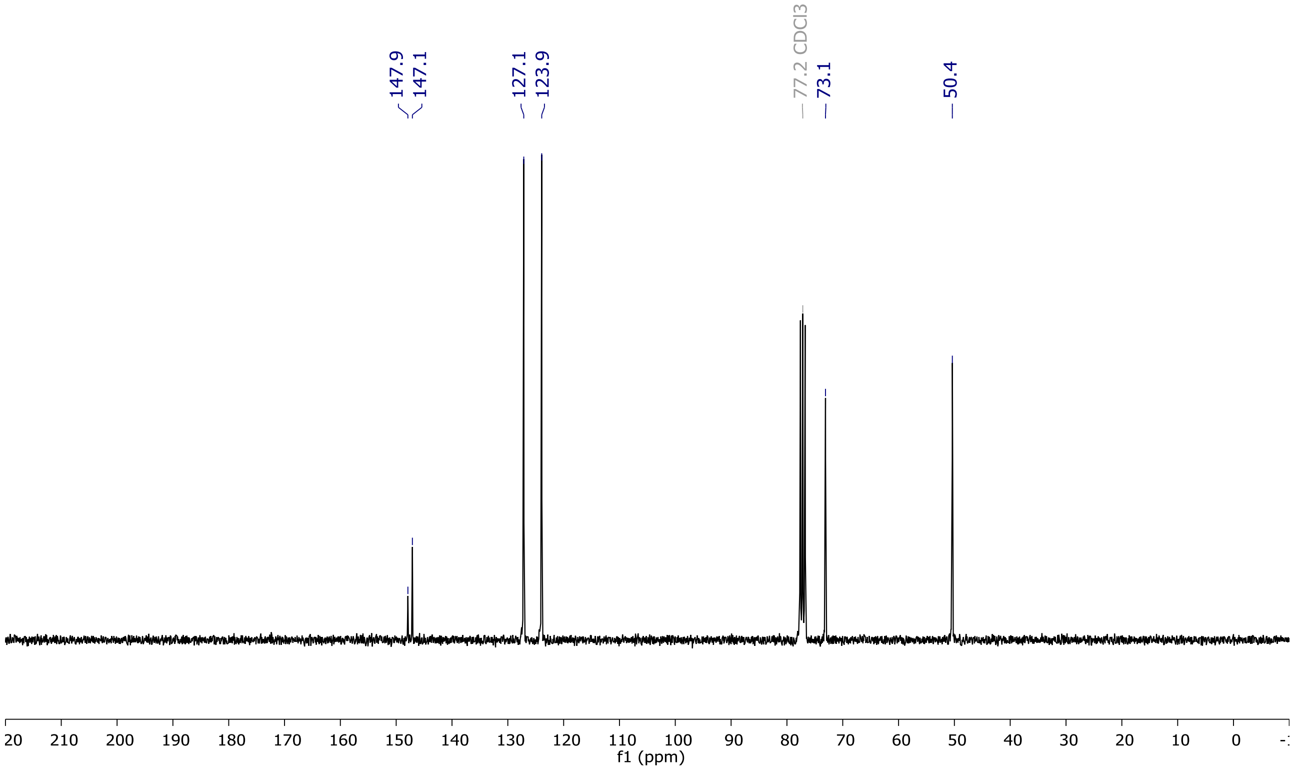

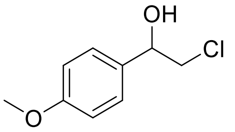

1n

^{1}H-NMR (300 MHz, $CDCl_3$)

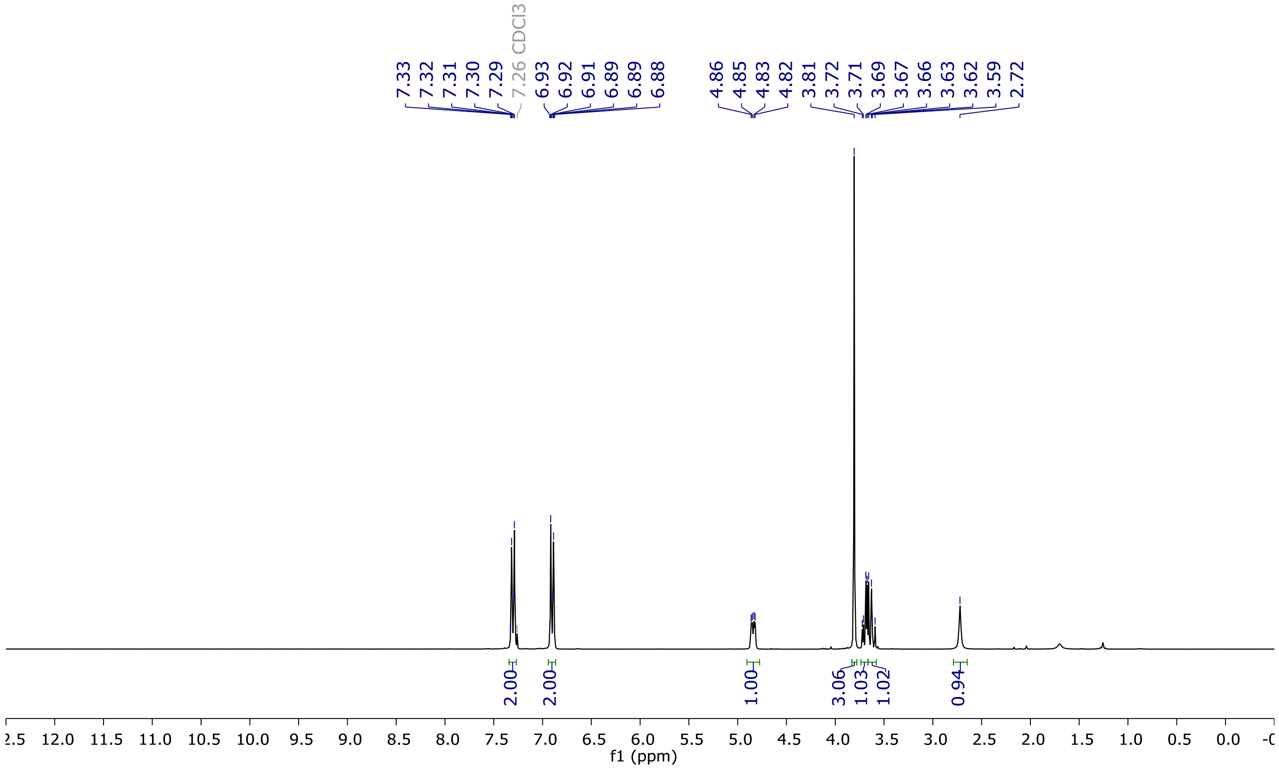

^{13}C-NMR (75 MHz, $CDCl_3$)

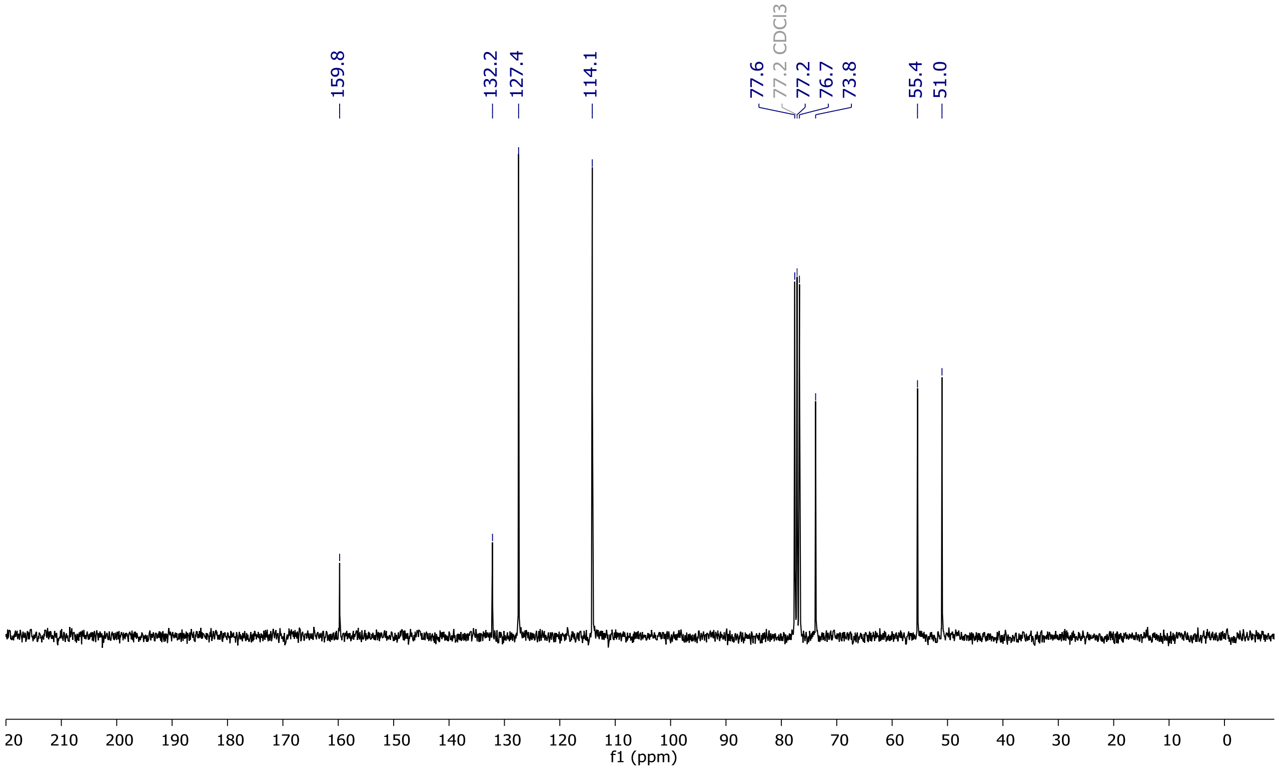

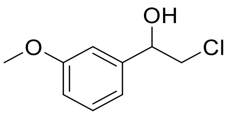

1o

^{1}H-NMR (300 MHz, $CDCl_3$)

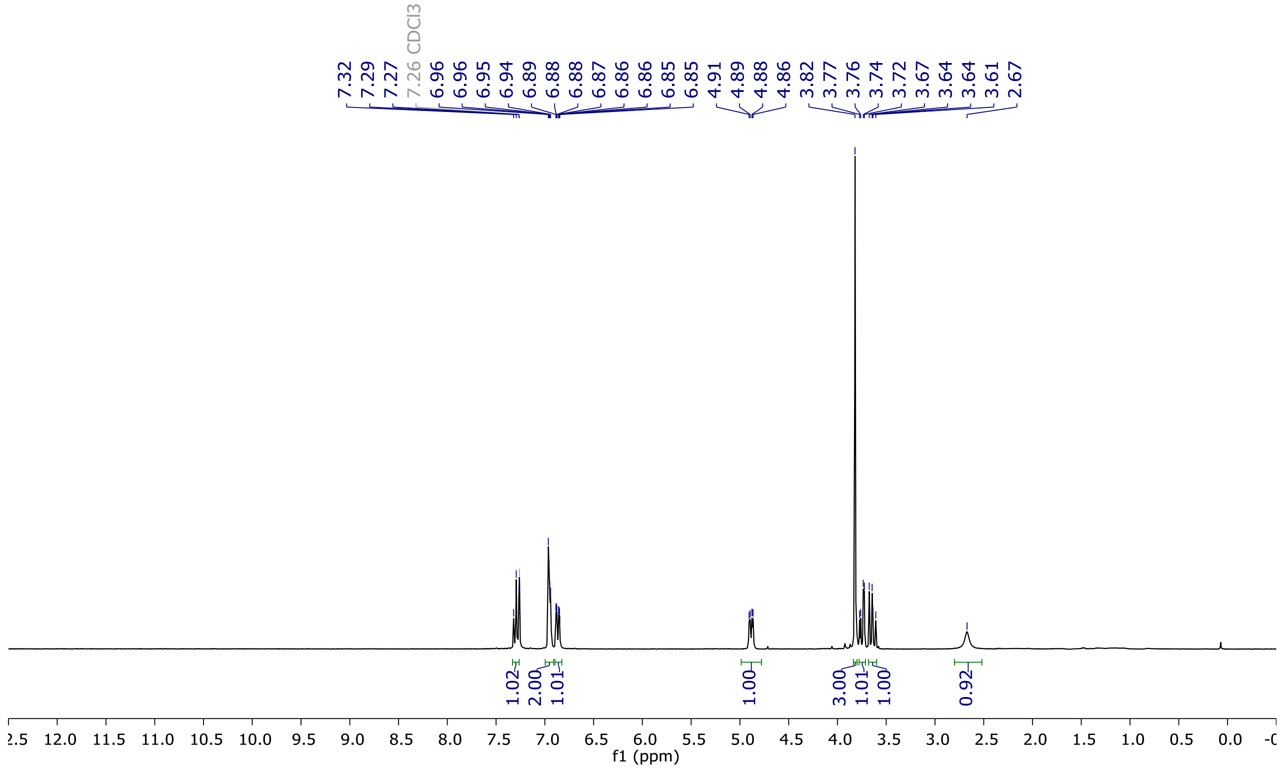

^{13}C-NMR (75 MHz, $CDCl_3$)

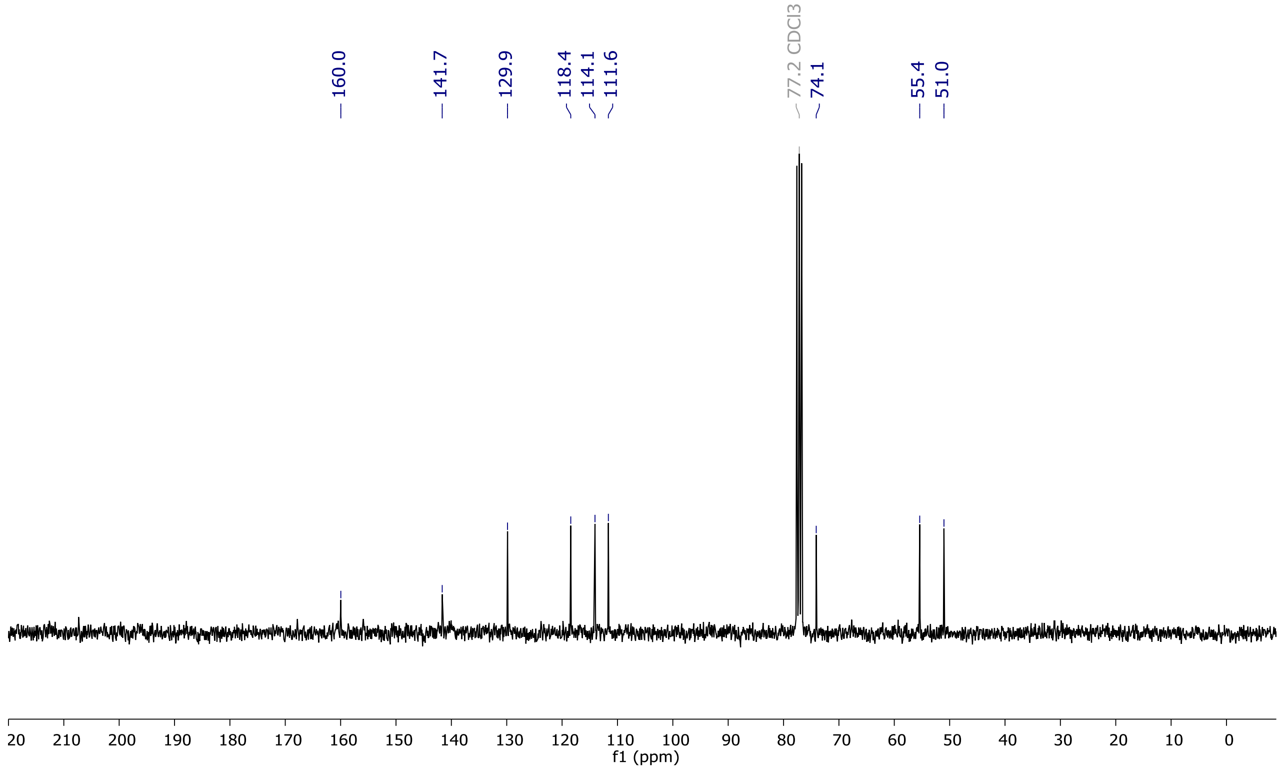

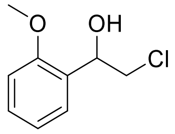

1p

[1]**H-NMR** (300 MHz, $CDCl_3$)

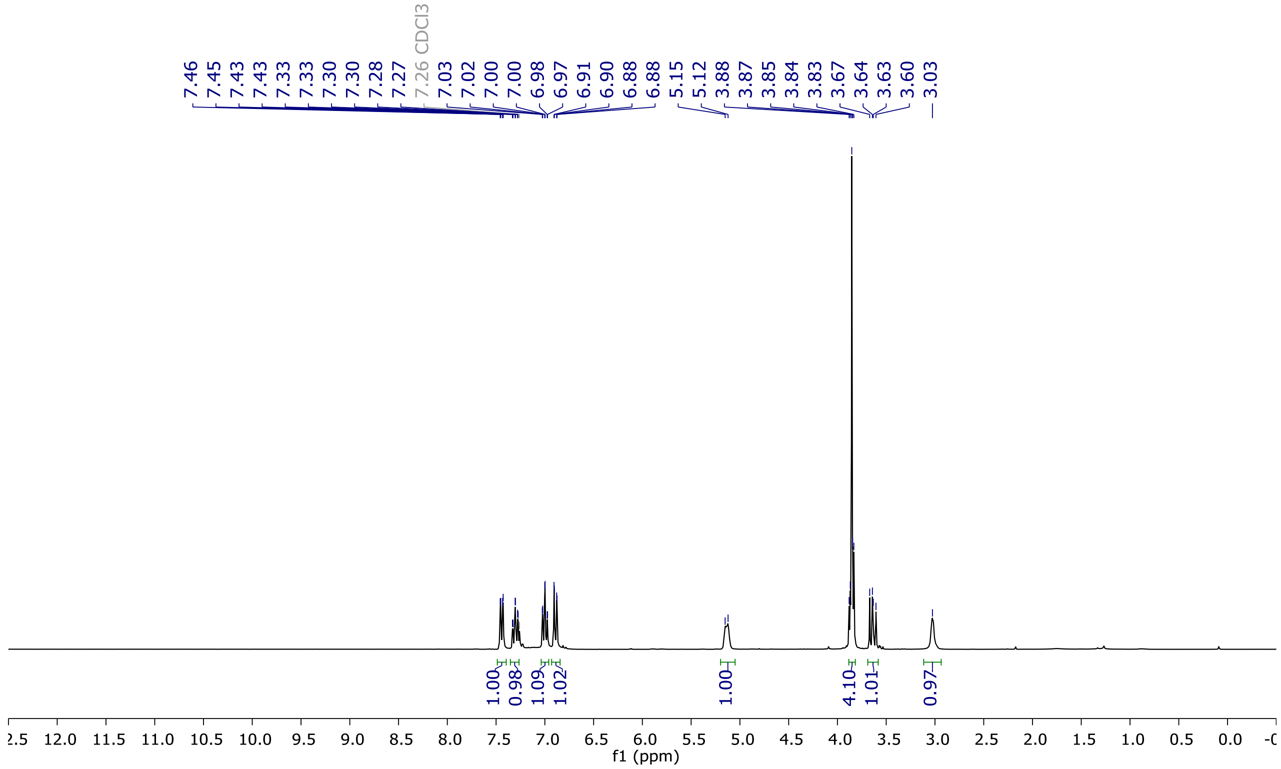

[13]**C-NMR** (75 MHz, $CDCl_3$)

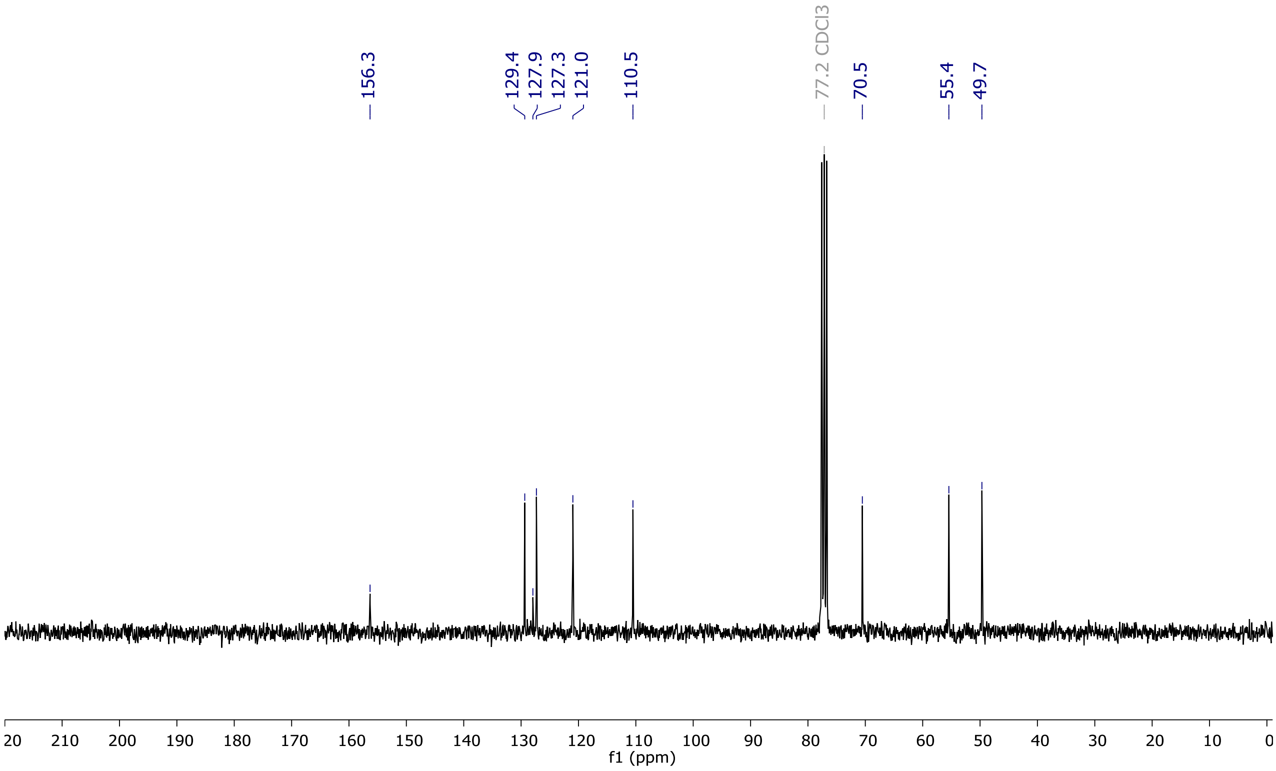

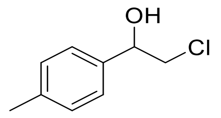

1q

^{1}H-NMR (300 MHz, $CDCl_3$)

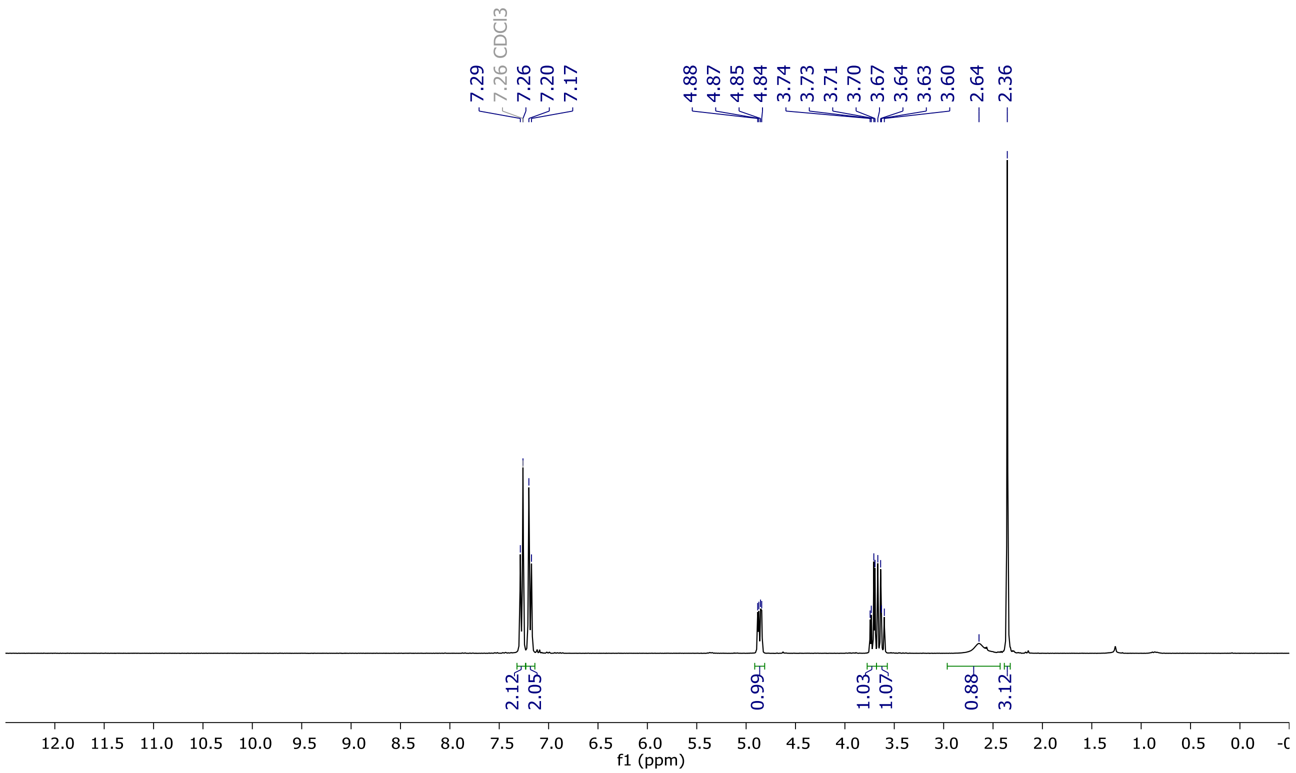

^{13}C-NMR (75 MHz, $CDCl_3$)

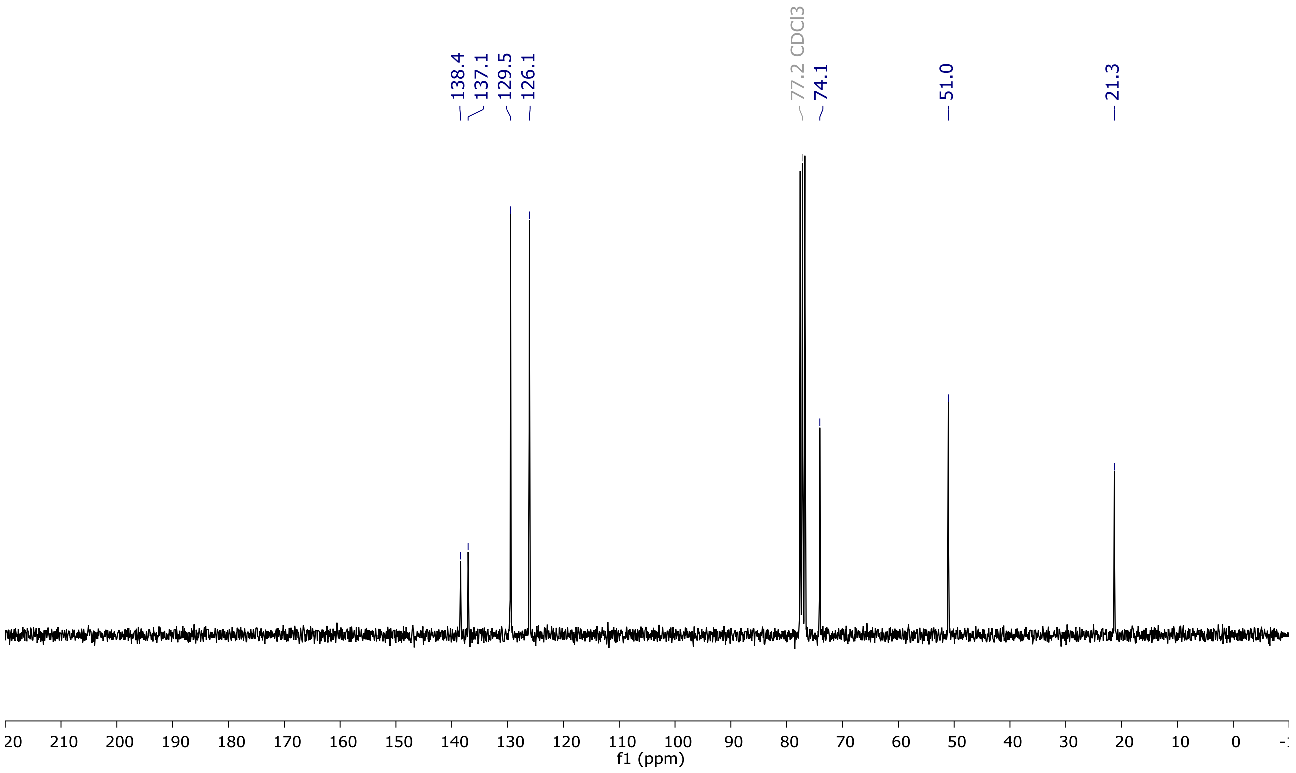

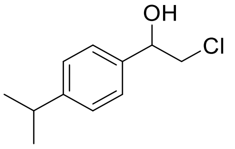

1r

[1]**H-NMR** (300 MHz, $CDCl_3$)

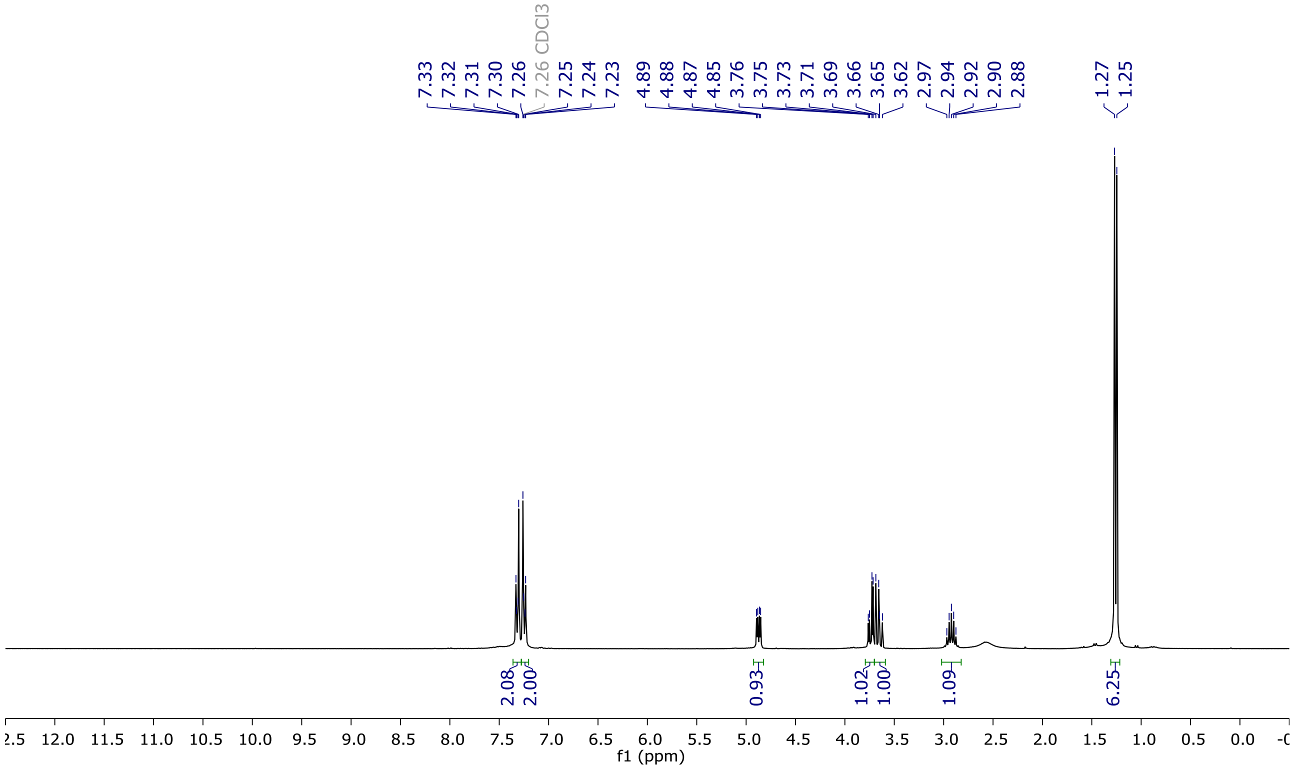

[13]**C-NMR** (75 MHz, $CDCl_3$)

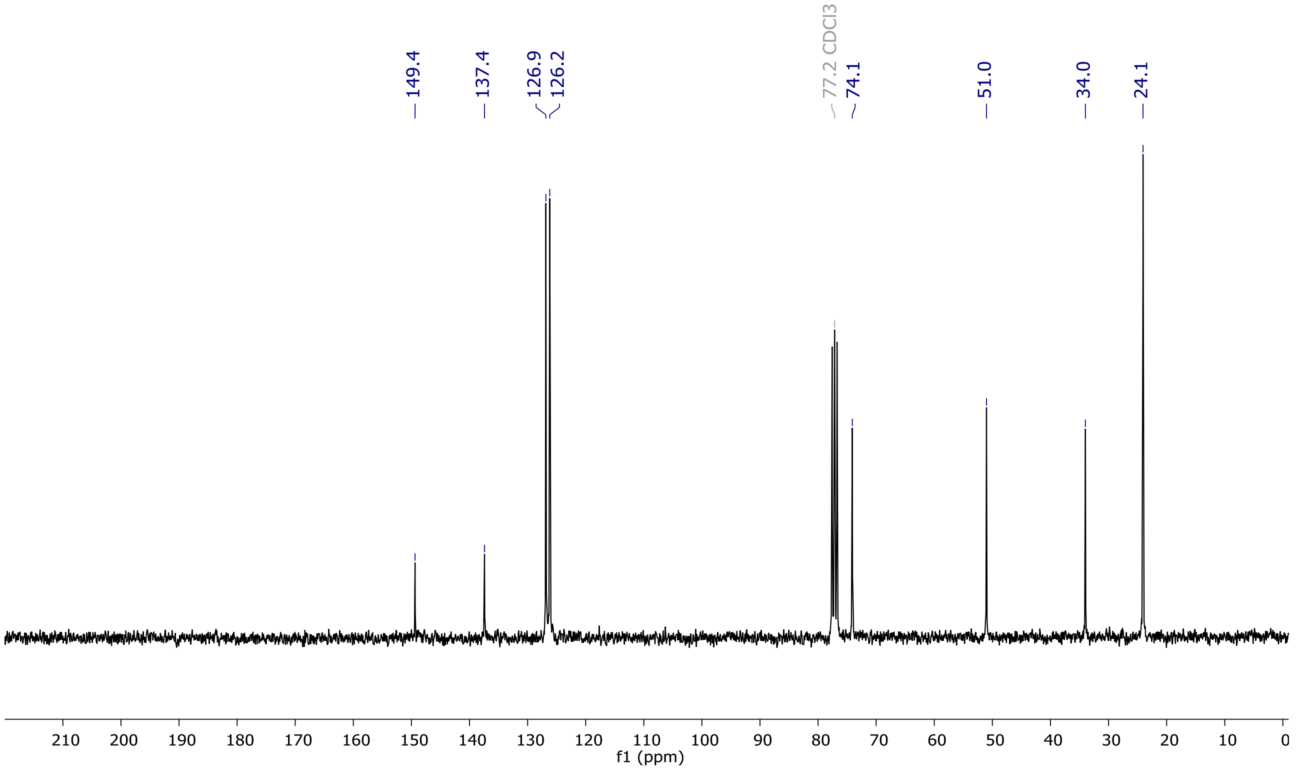

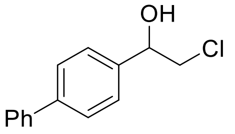

1s

^{1}H-NMR (300 MHz, $CDCl_3$)

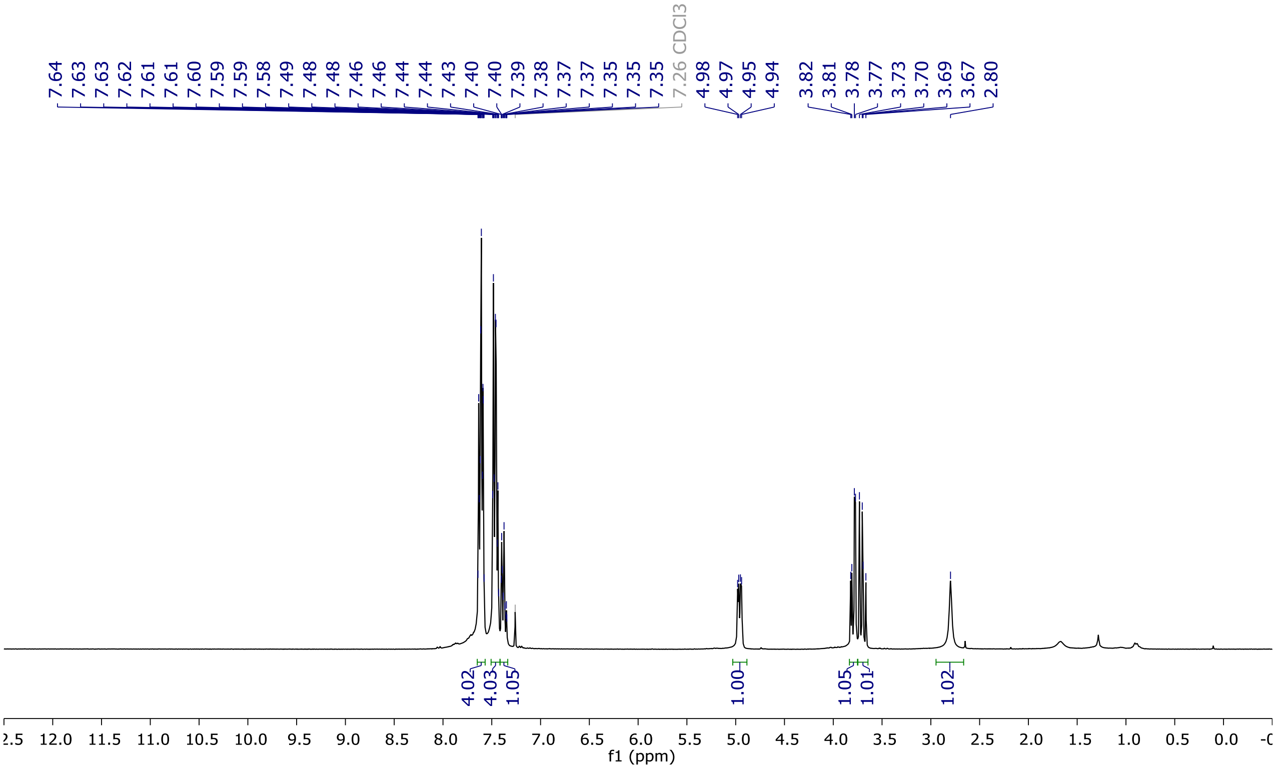

^{13}C-NMR (75 MHz, $CDCl_3$)

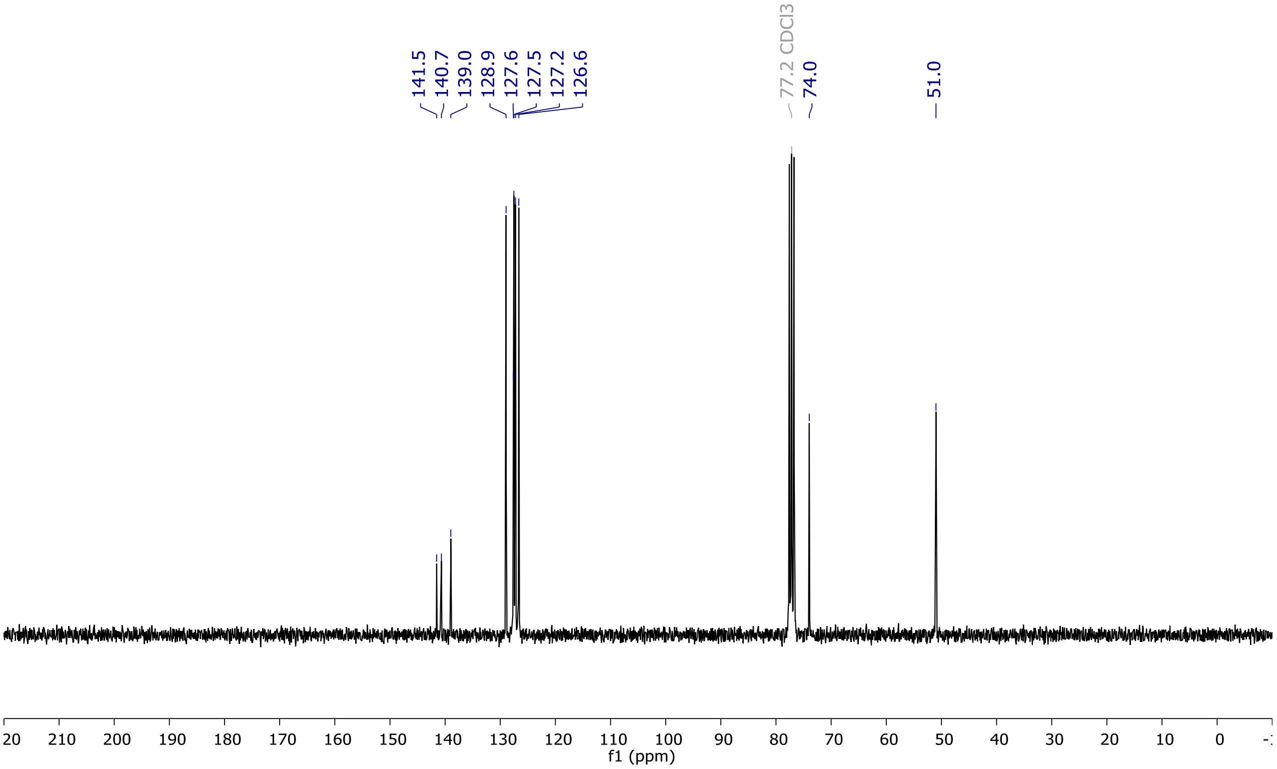

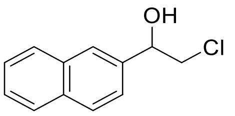

1t

[1]**H-NMR** (300 MHz, $CDCl_3$)

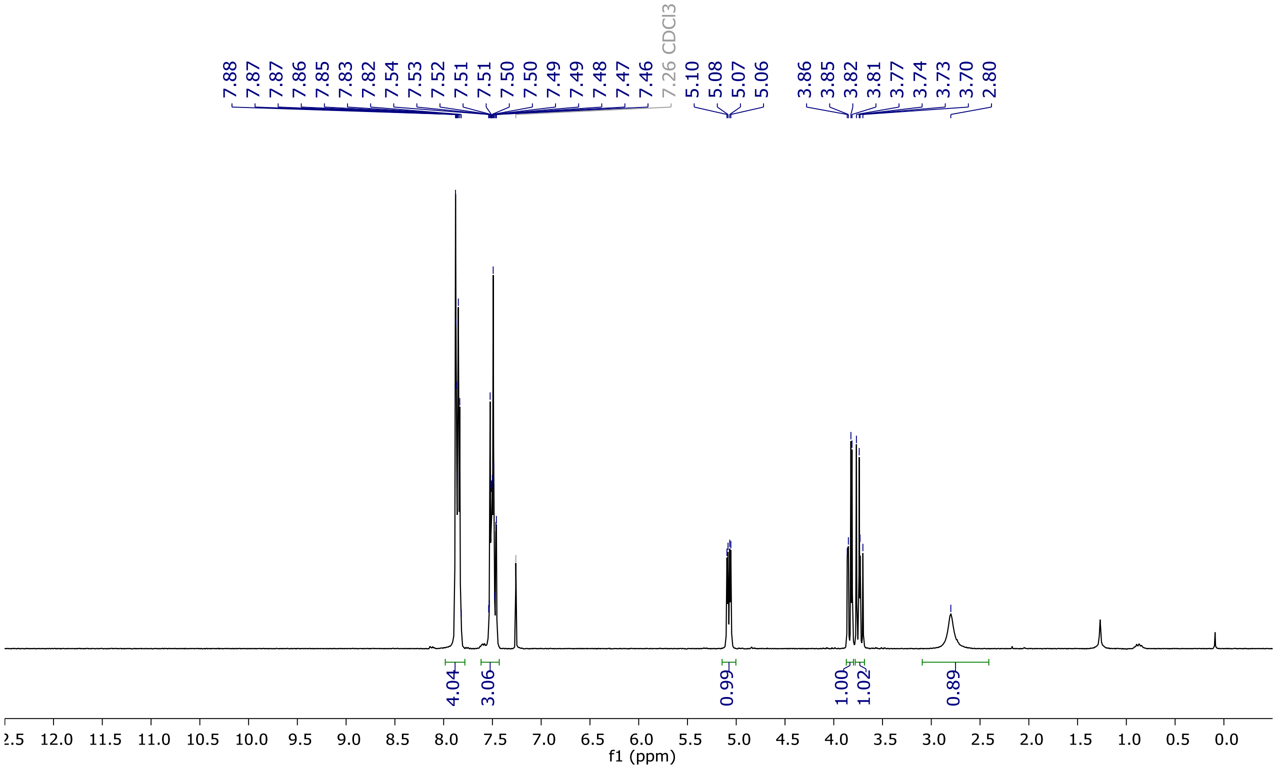

[13]**C-NMR** (75 MHz, $CDCl_3$)

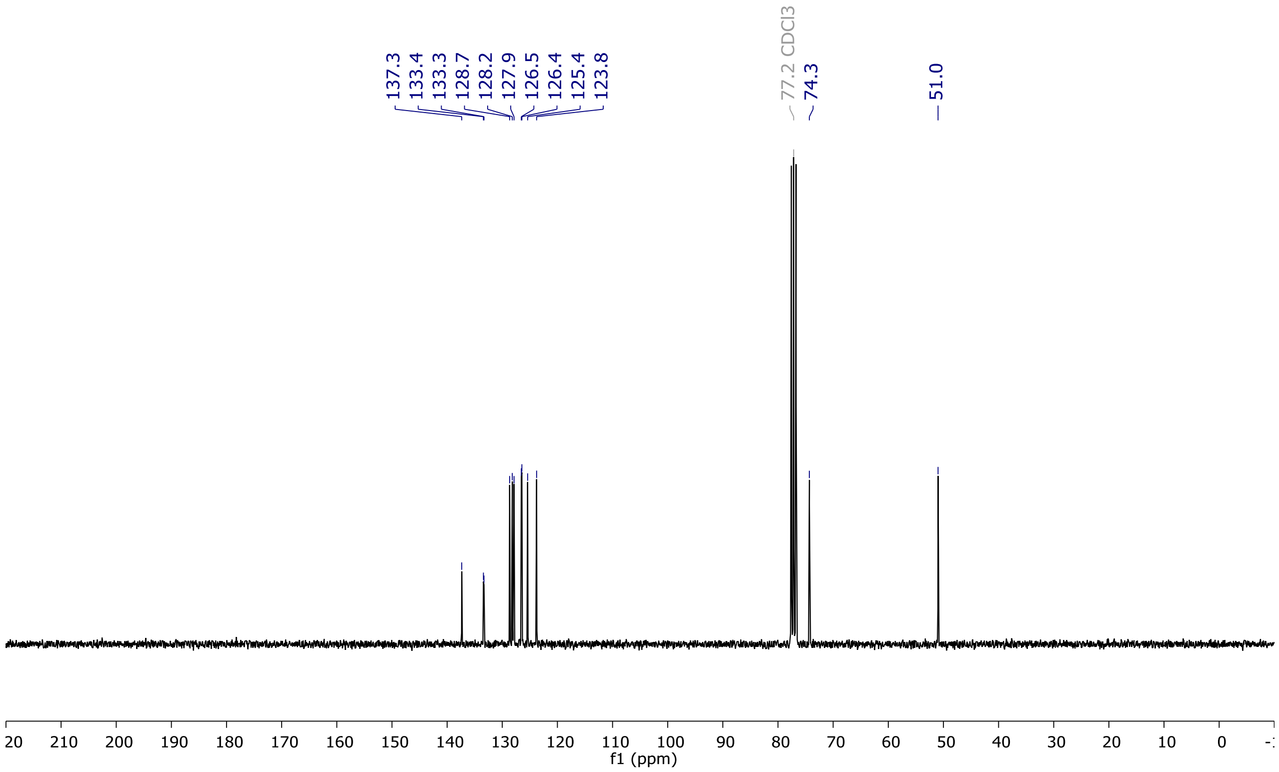

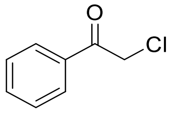

2a

^{1}H-NMR (300 MHz, $CDCl_3$)

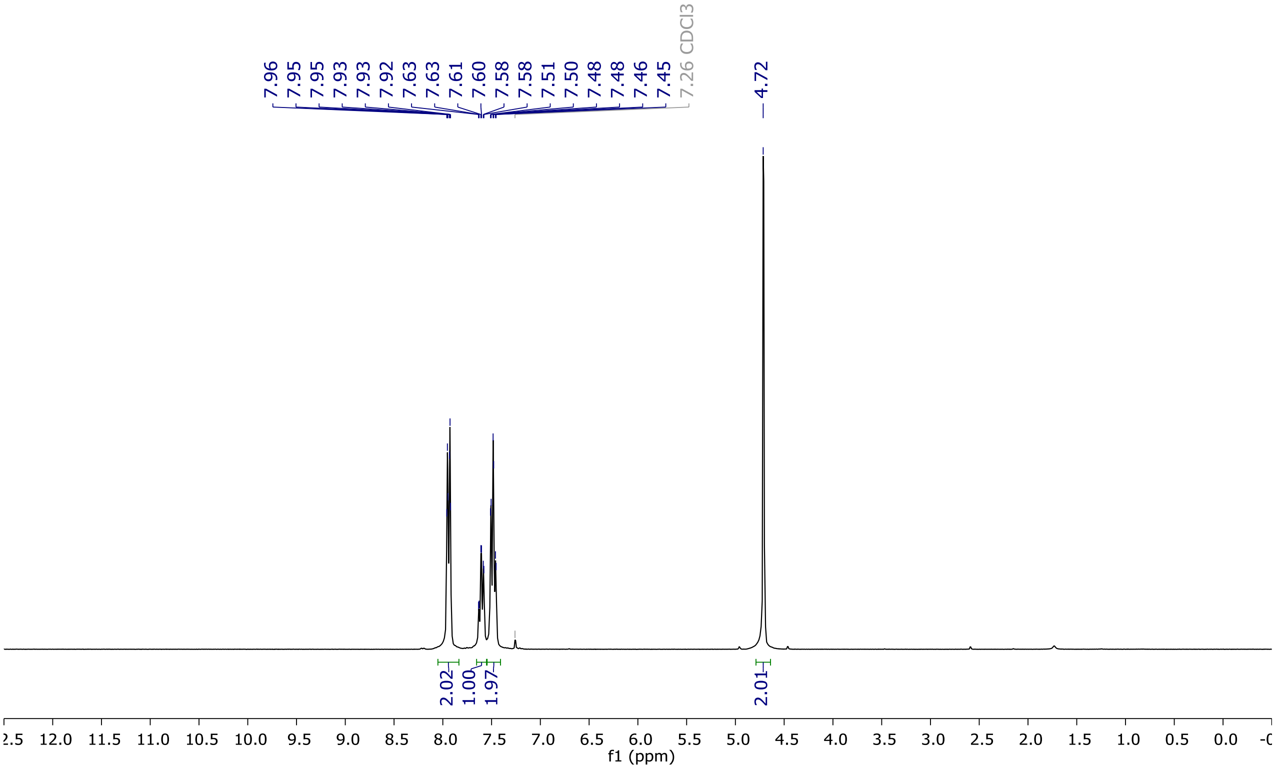

^{13}C-NMR (75 MHz, $CDCl_3$)

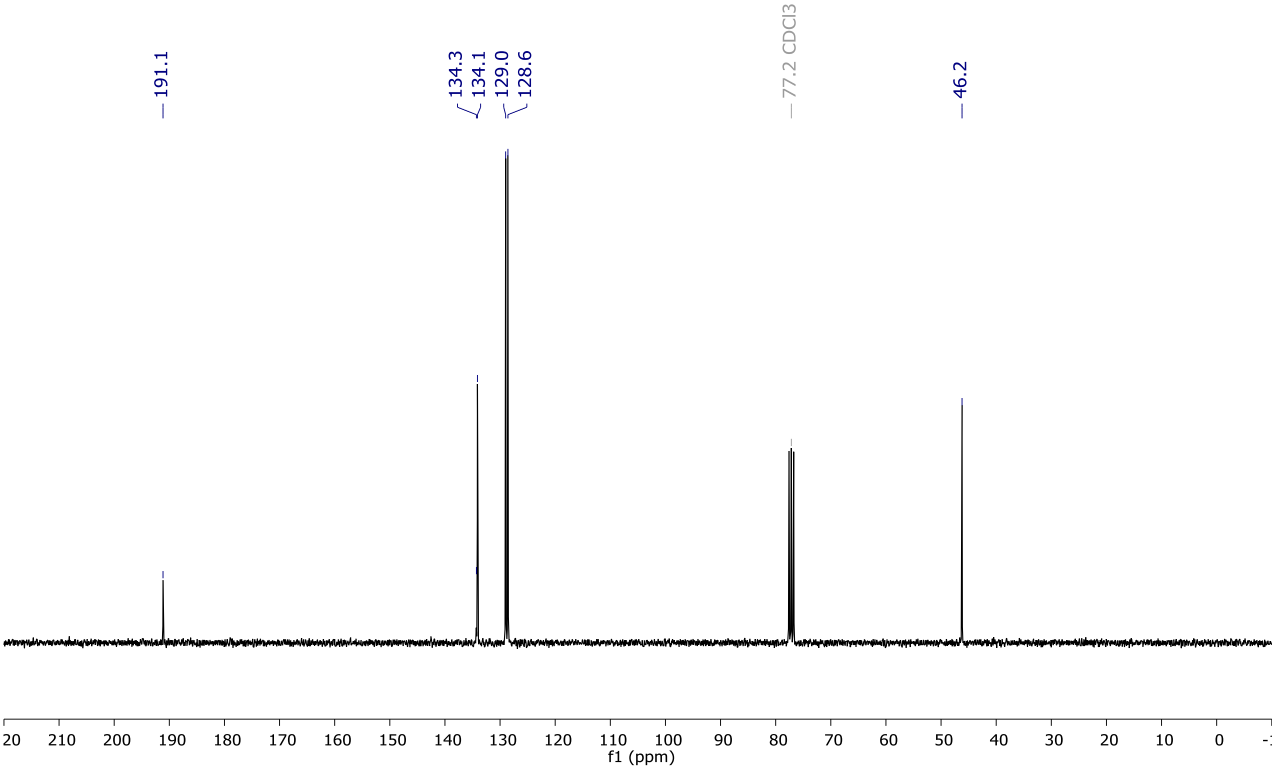

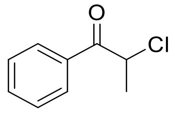

2d

^{1}H-NMR (300 MHz, $CDCl_3$)

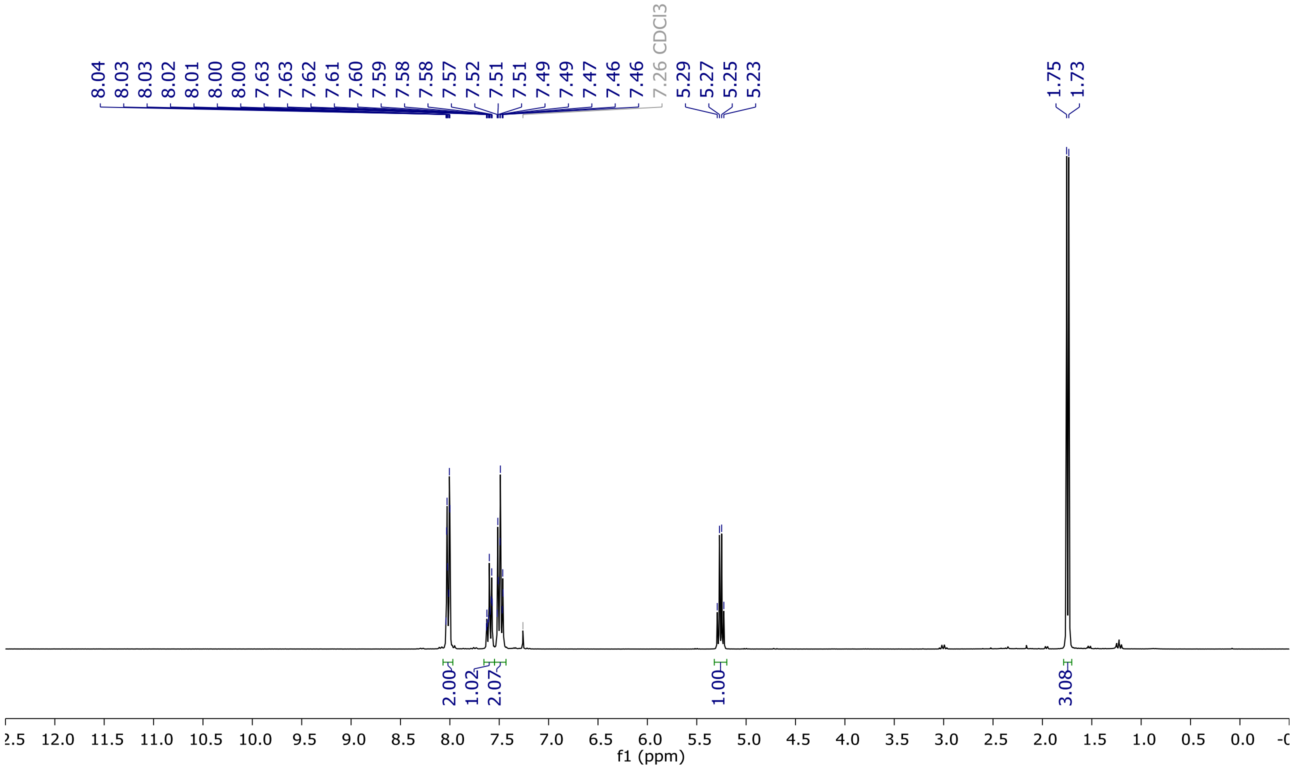

^{13}C-NMR (75 MHz, $CDCl_3$)

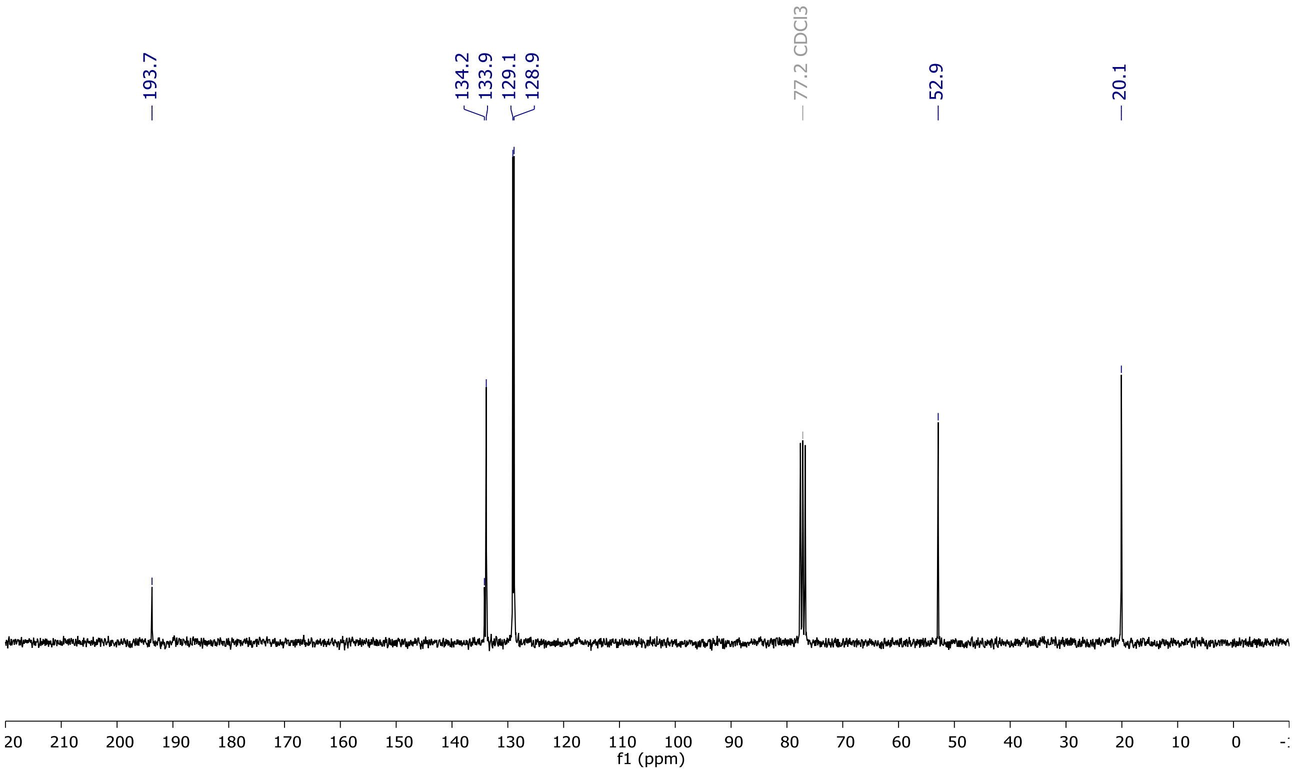

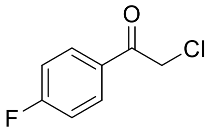

2e

^{1}H-NMR (300 MHz, $CDCl_3$)

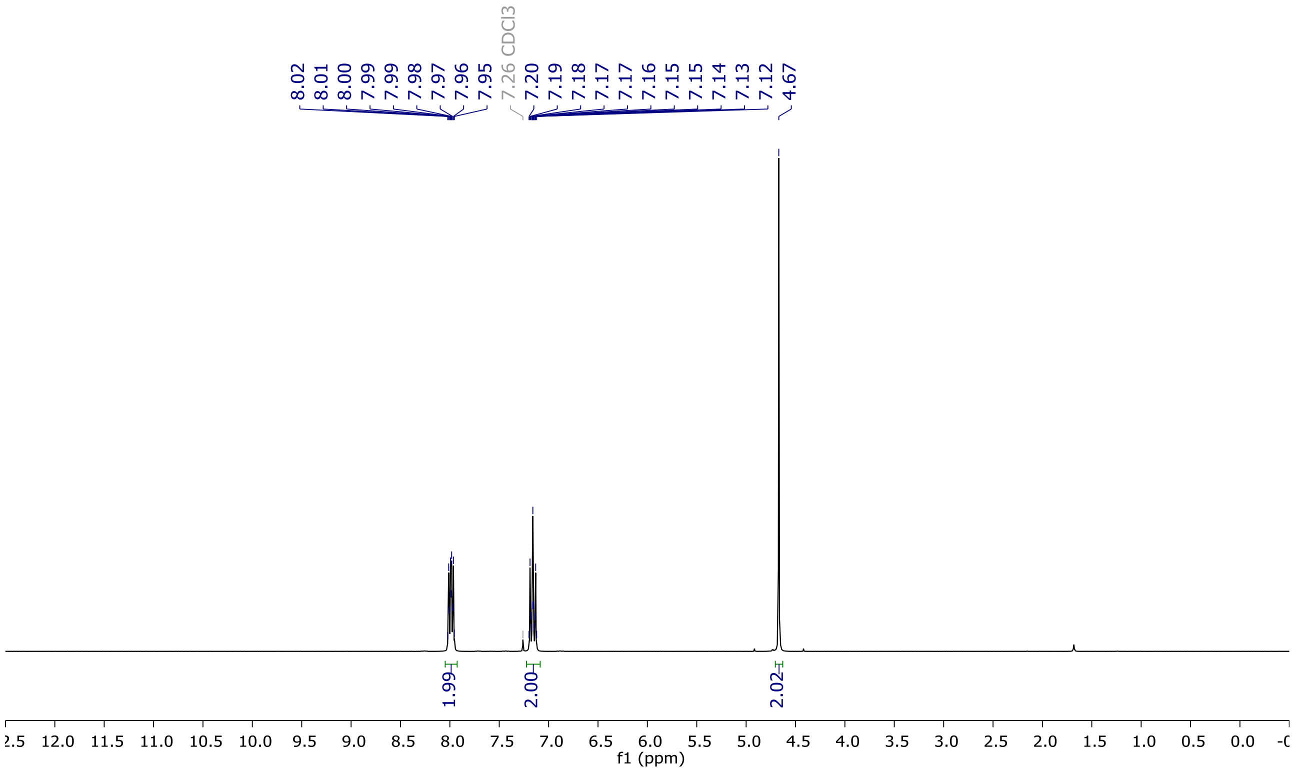

^{13}C-NMR (75 MHz, $CDCl_3$)

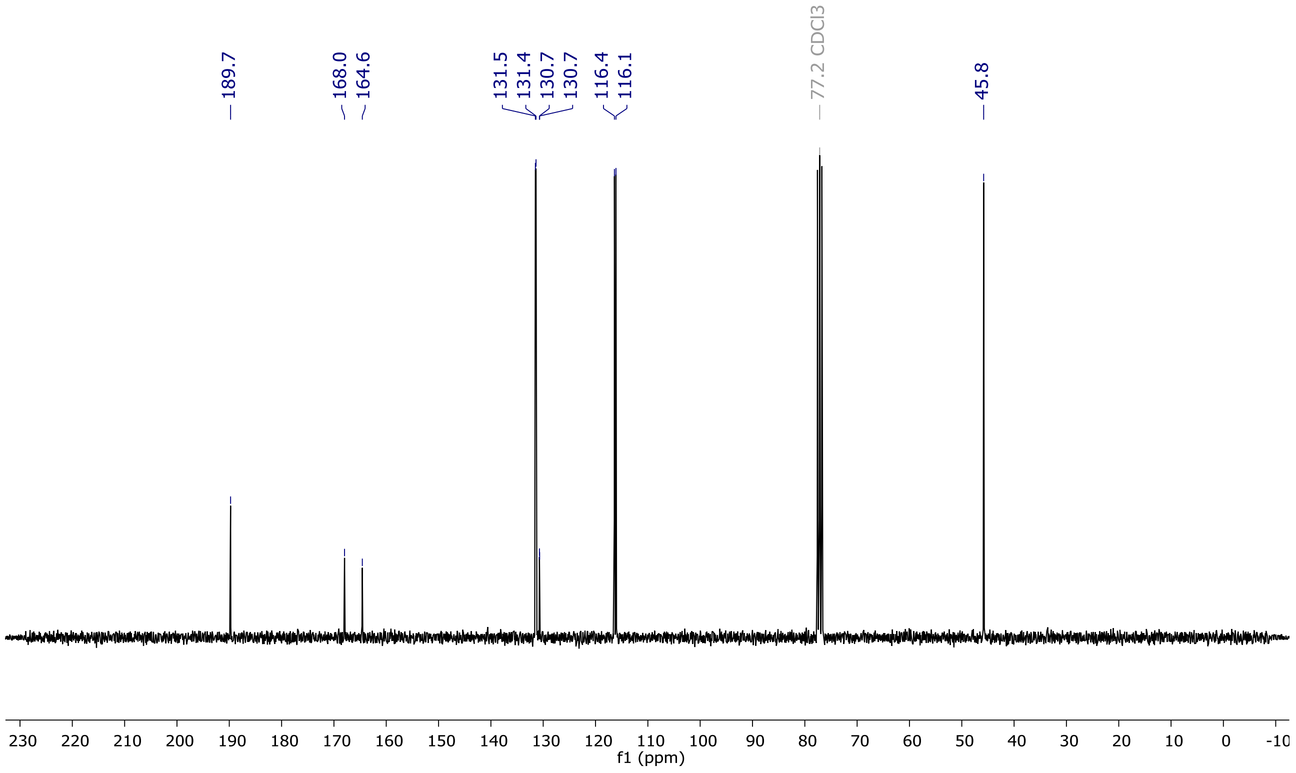

^{19}F{^{1}H}-NMR (282 MHz, $CDCl_3$)

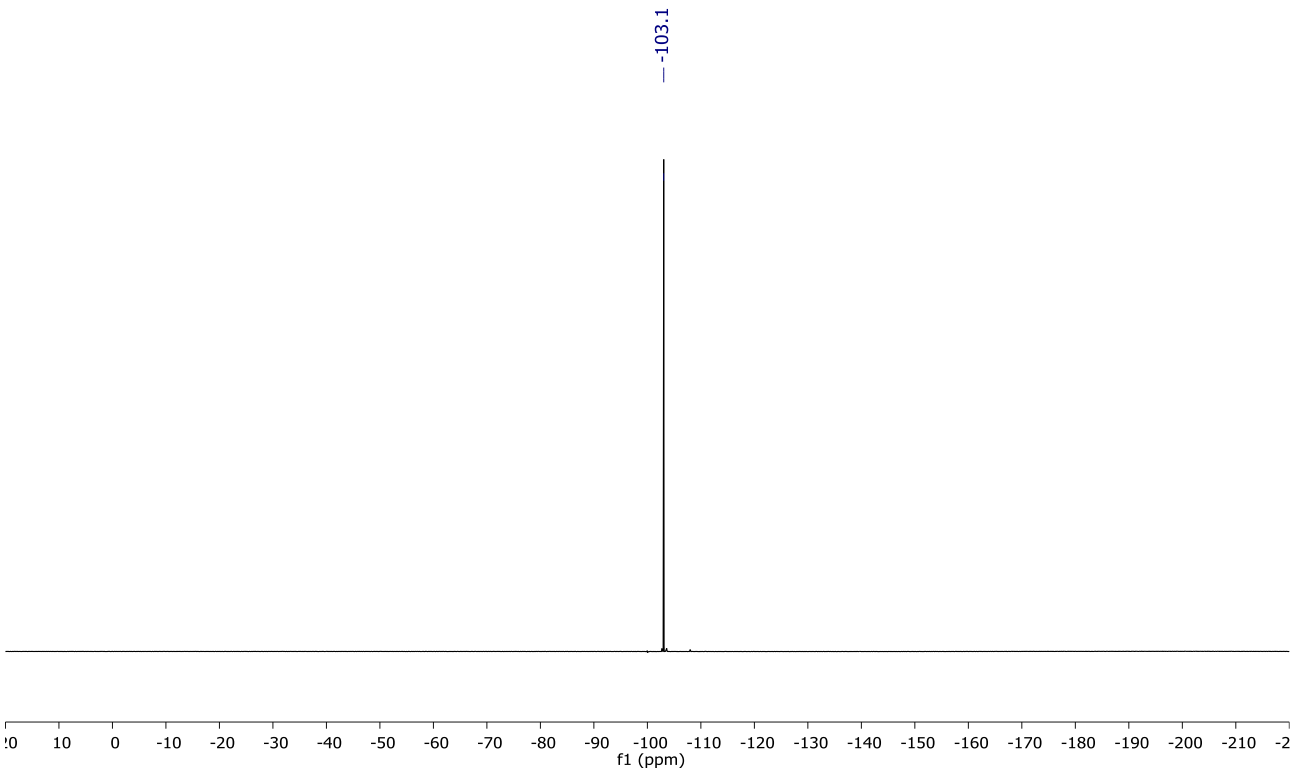

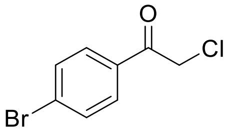

2f

^{1}H-NMR (300 MHz, $CDCl_3$)

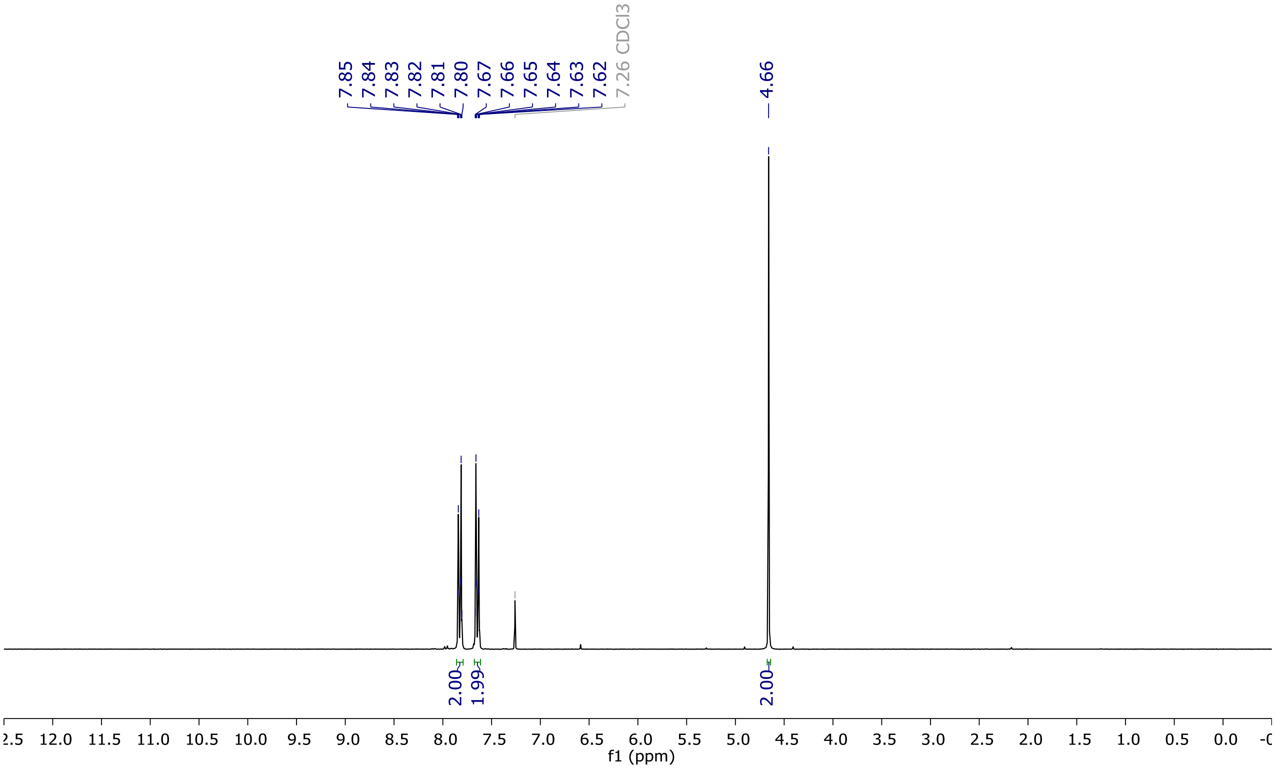

^{13}C-NMR (75 MHz, $CDCl_3$)

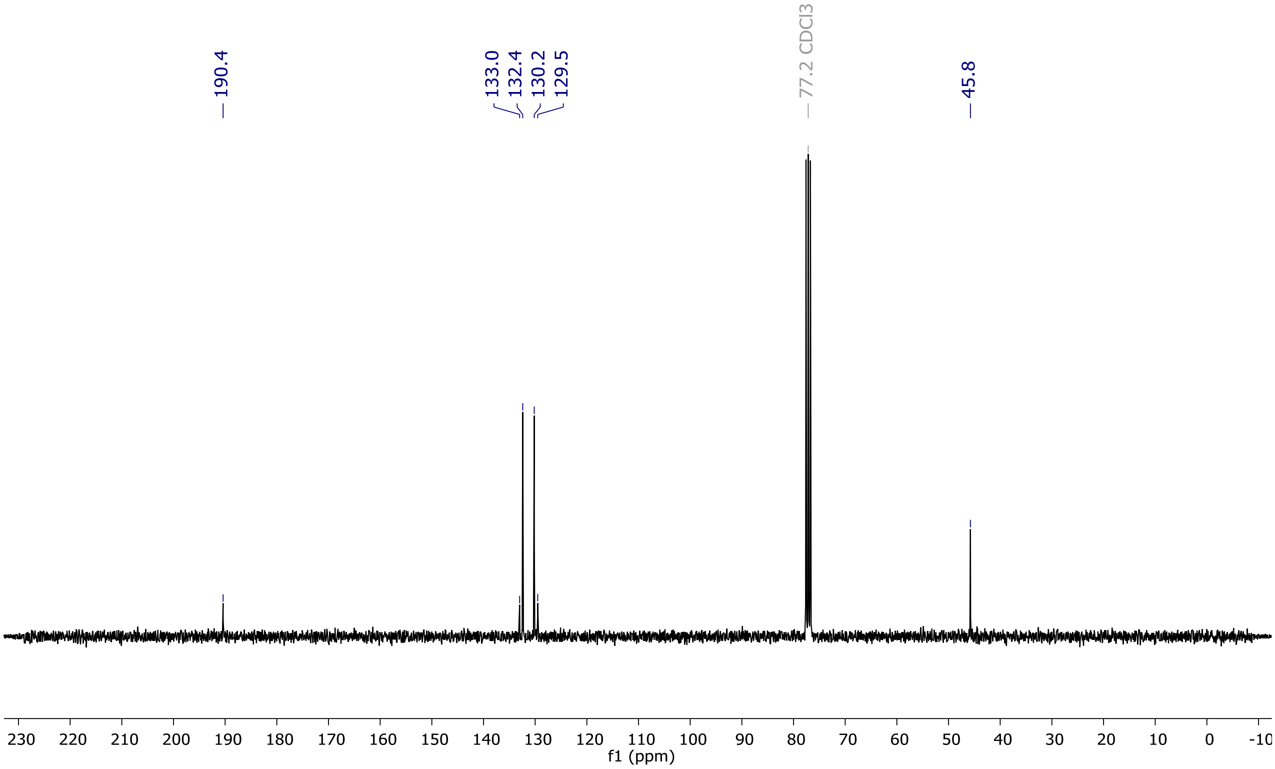

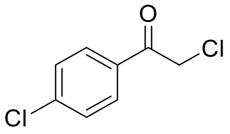

2g

^{1}H-NMR (300 MHz, $CDCl_3$)

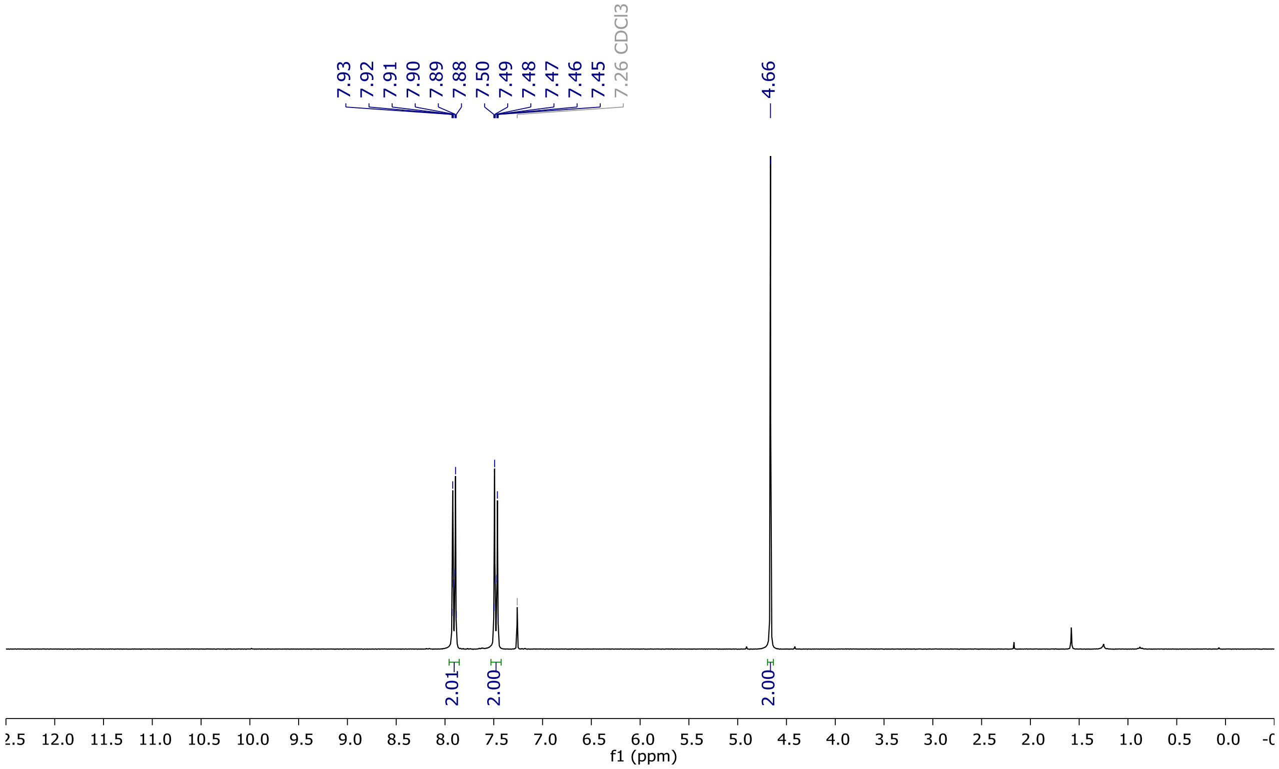

^{13}C-NMR (75 MHz, $CDCl_3$)

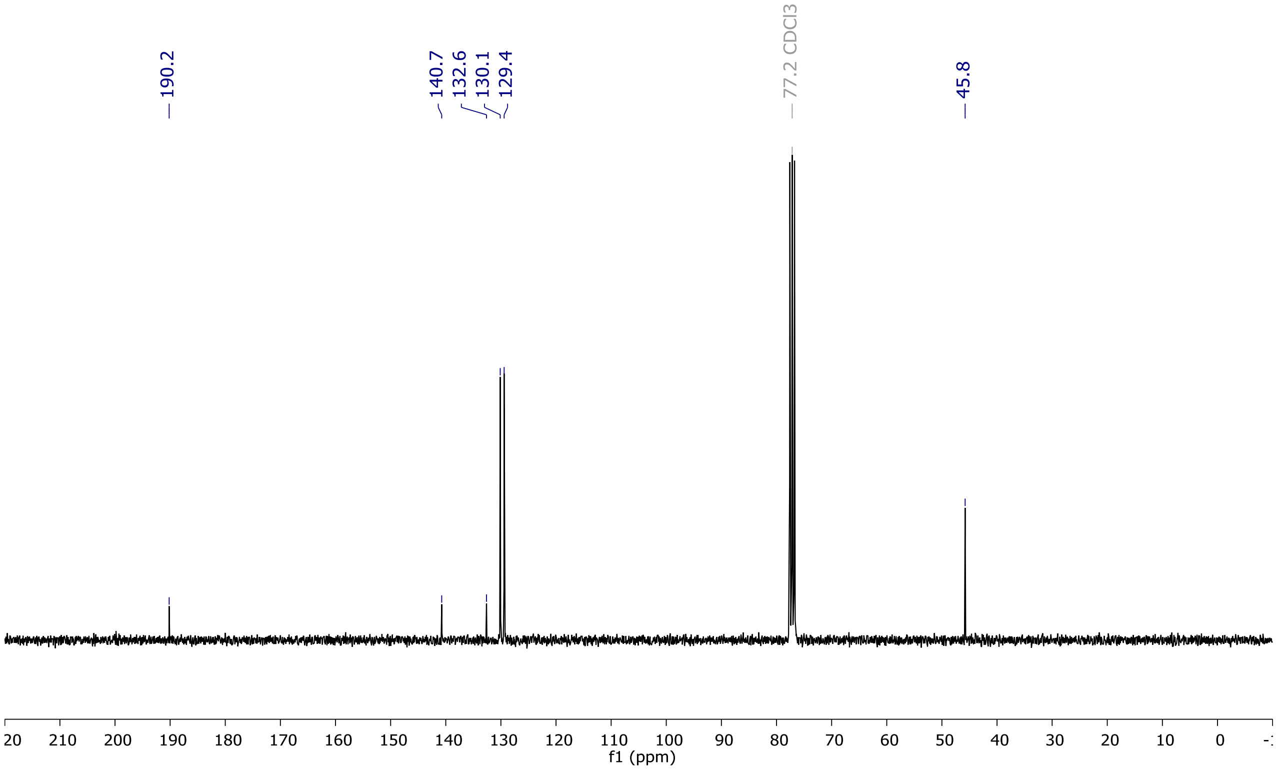

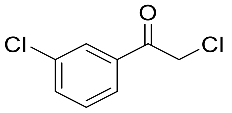

2h

^{1}H-NMR (300 MHz, $CDCl_3$)

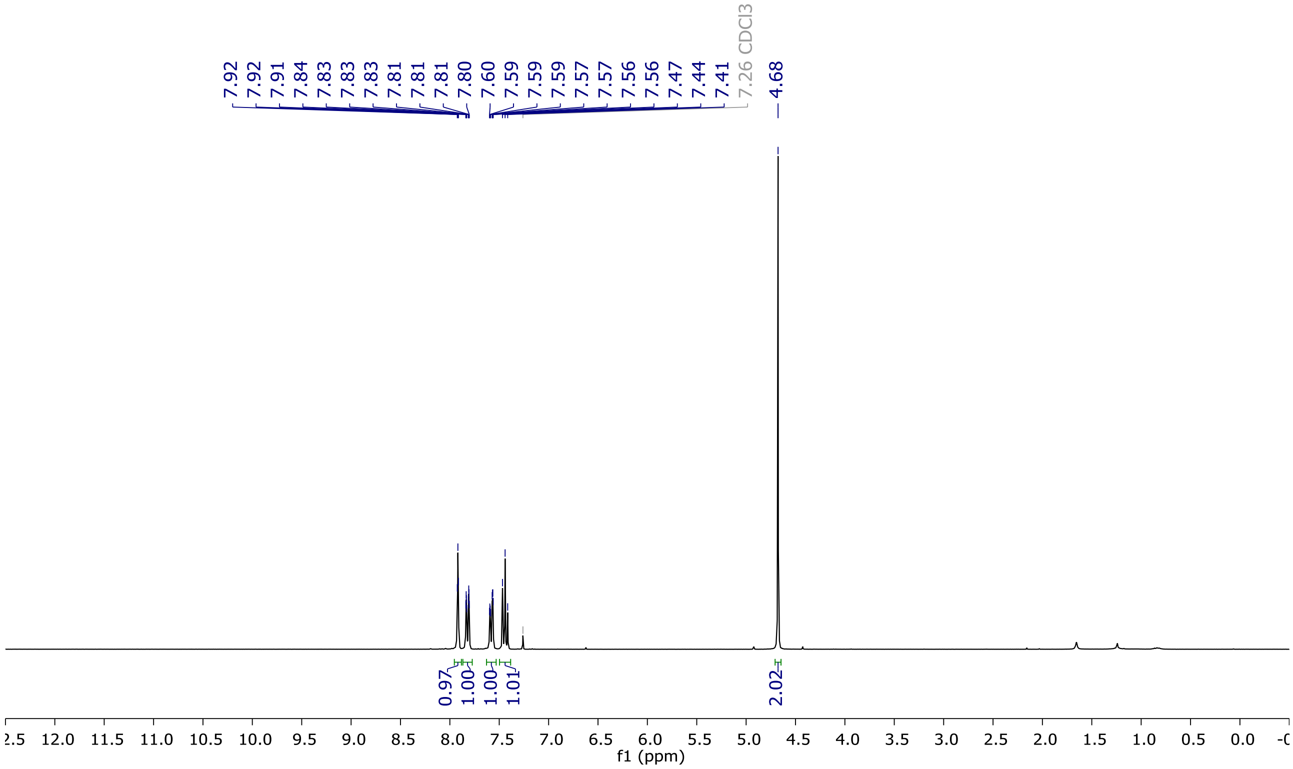

^{13}C-NMR (75 MHz, $CDCl_3$)

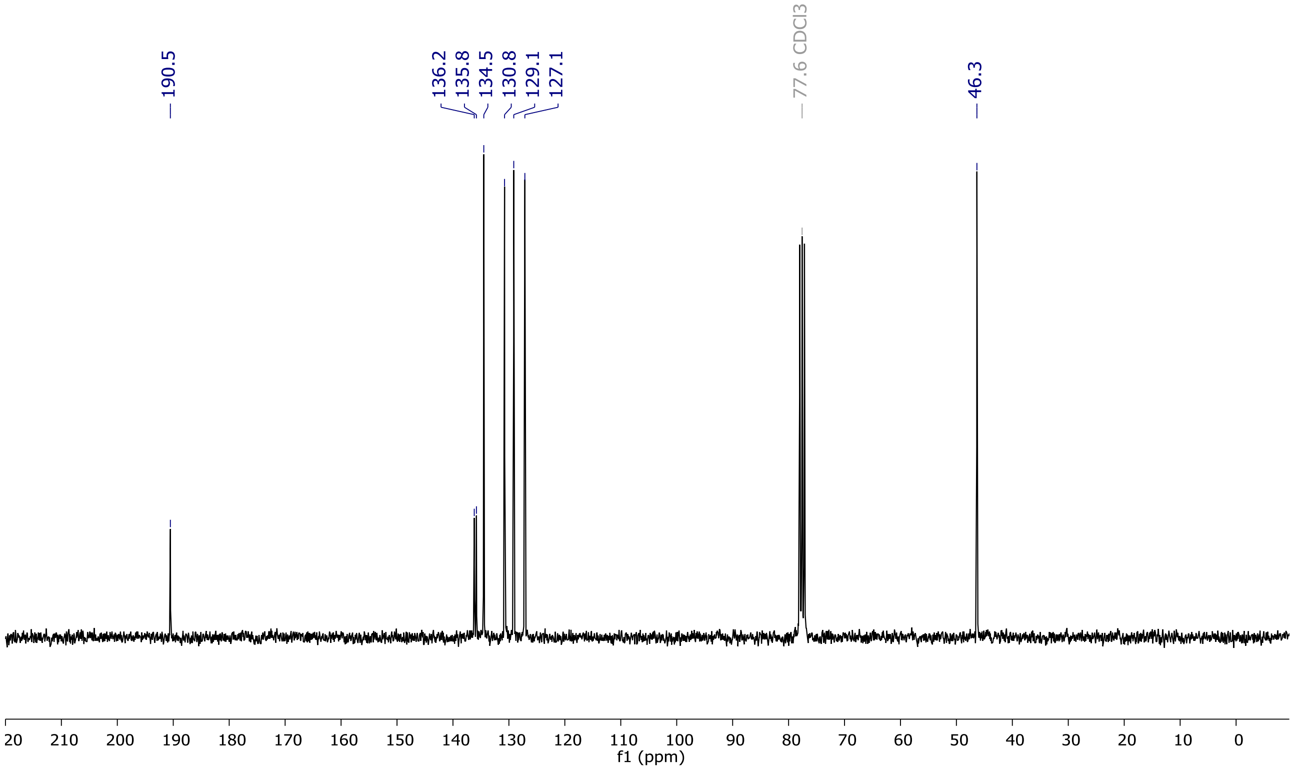

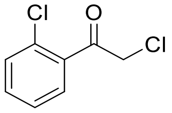

2i

^{1}H-NMR (300 MHz, $CDCl_3$)

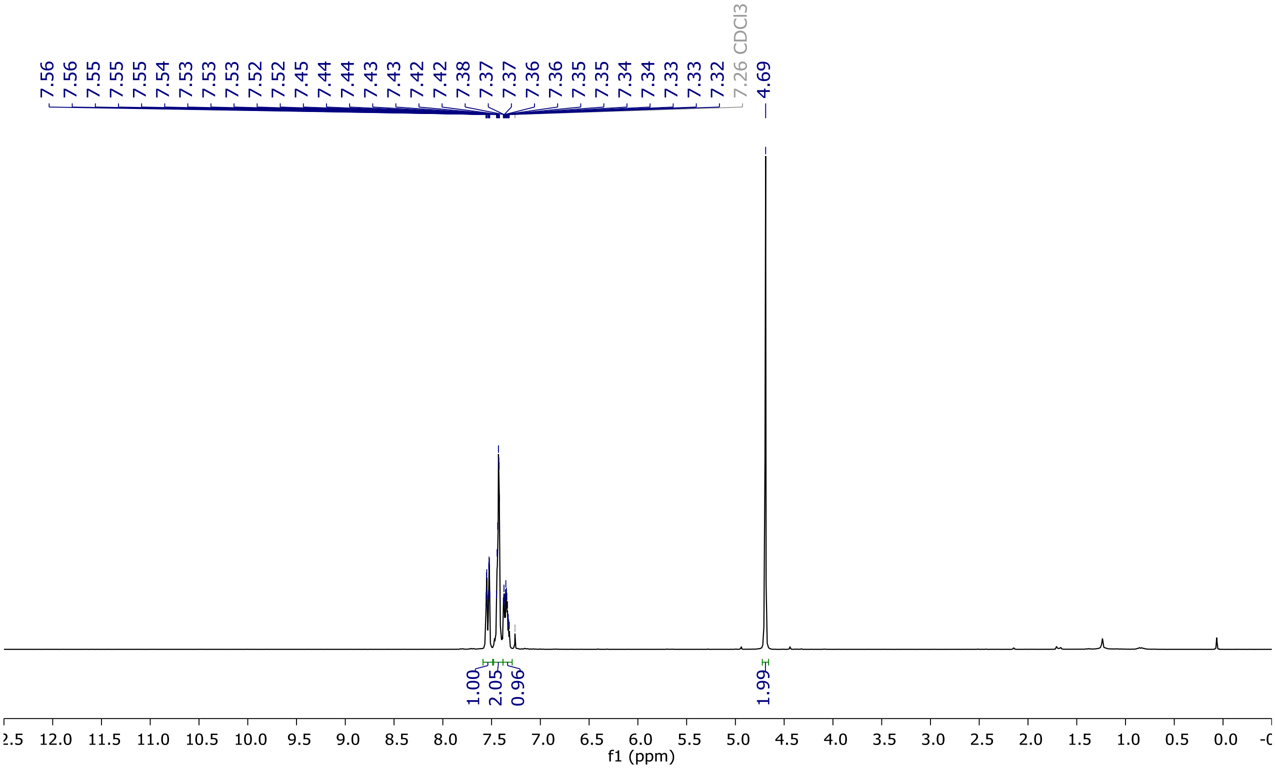

^{13}C-NMR (75 MHz, $CDCl_3$)

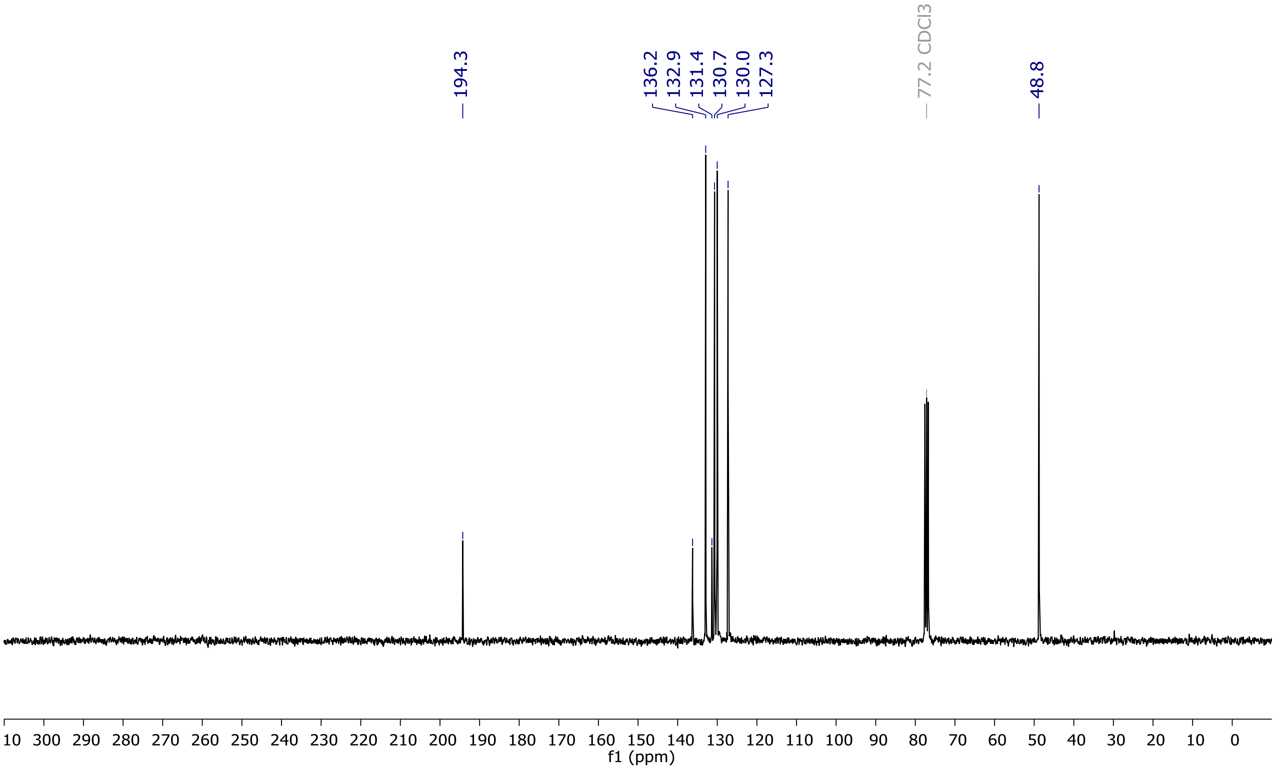

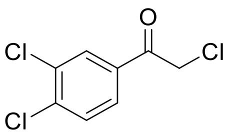

2j

^{1}H-NMR (300 MHz, $CDCl_3$)

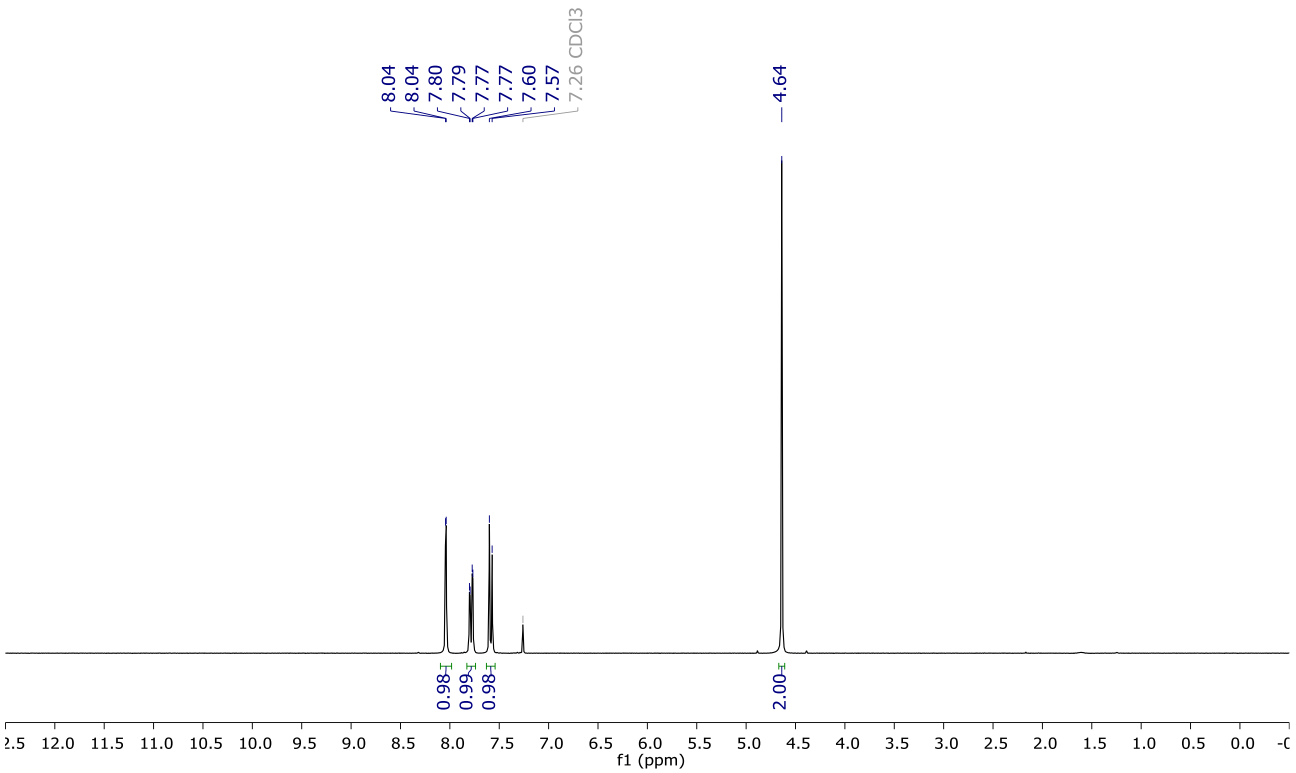

^{13}C-NMR (75 MHz, $CDCl_3$)

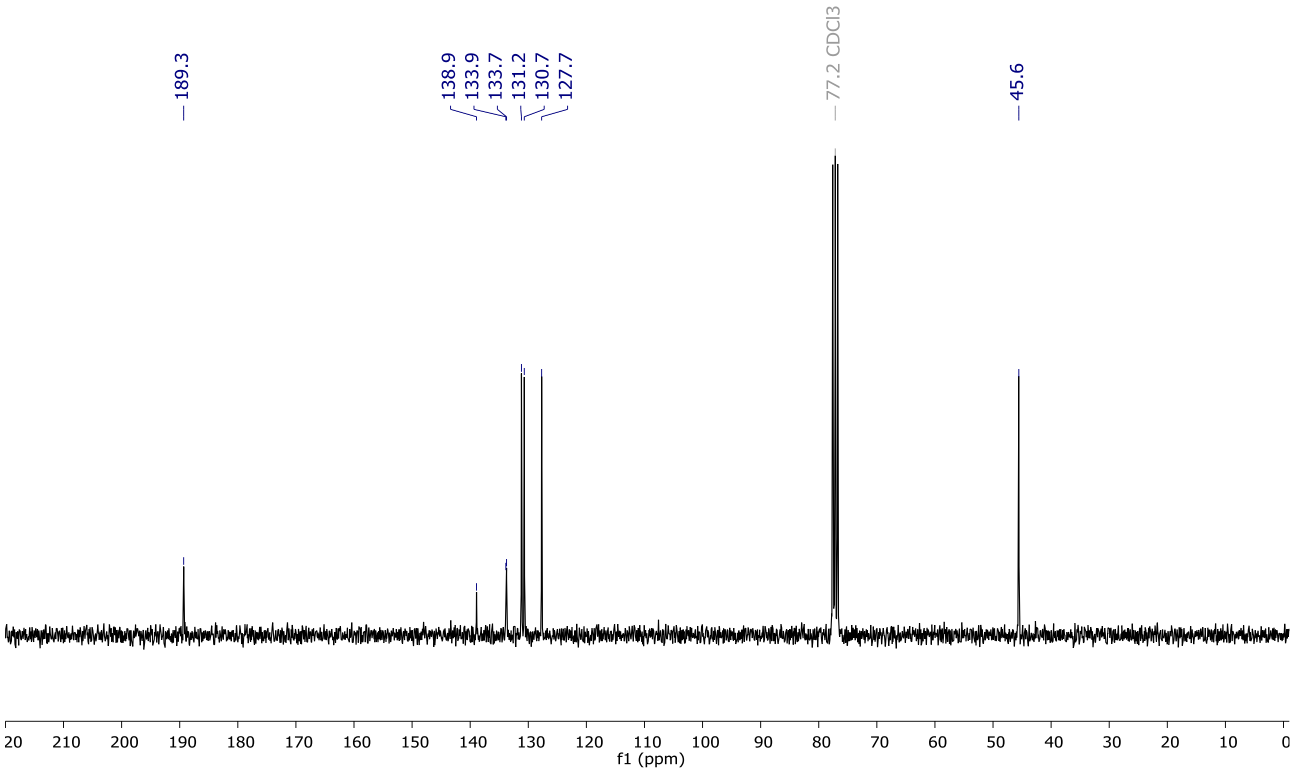

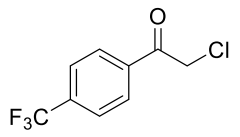

2l

^{1}H-NMR (300 MHz, $CDCl_3$)

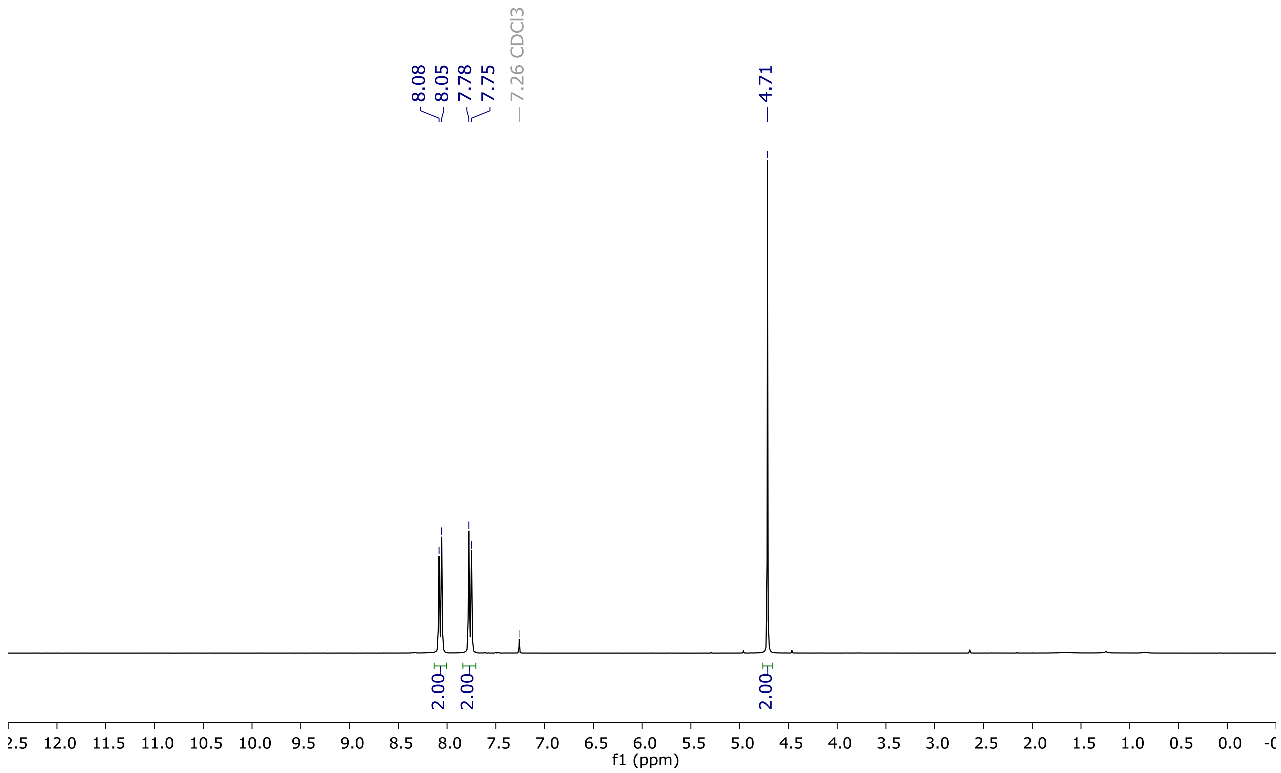

^{13}C-NMR (75 MHz, $CDCl_3$)

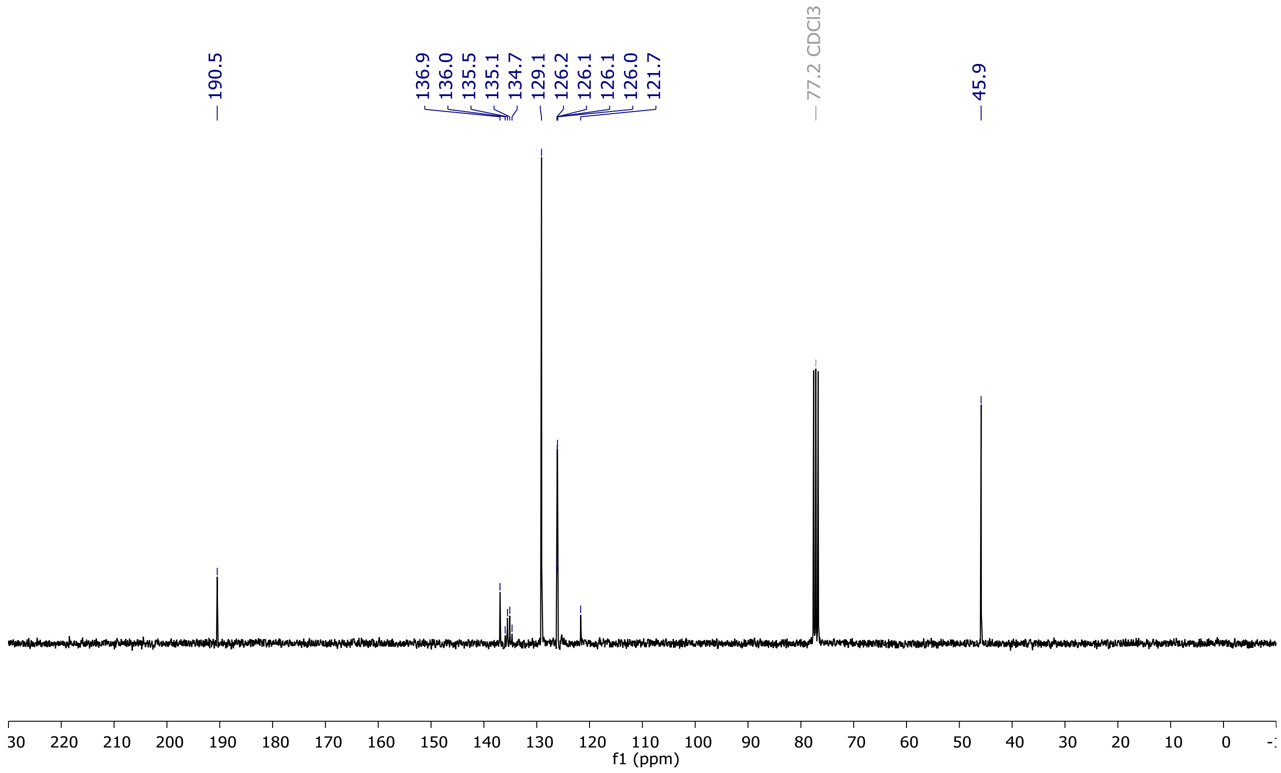

^{19}F{^{1}H}-NMR (282 MHz, $CDCl_3$)

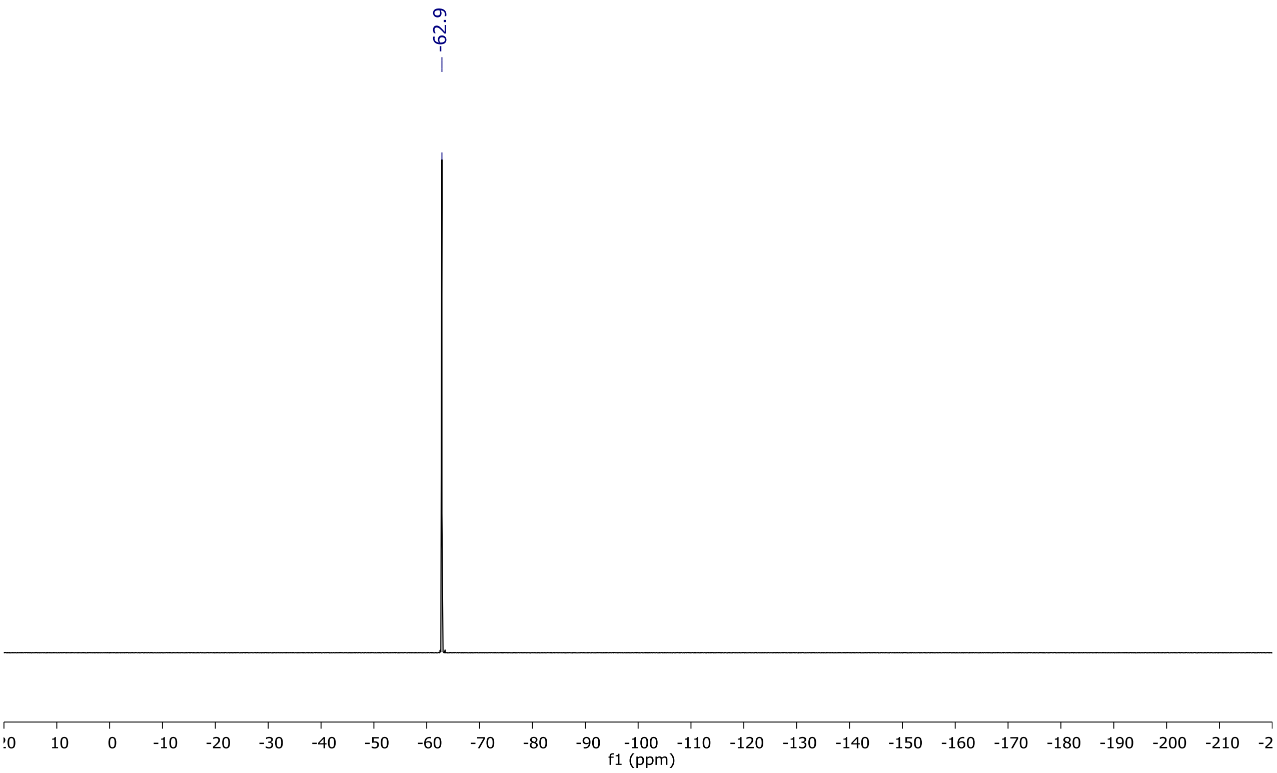

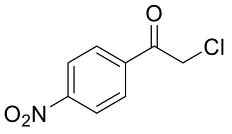

2m

^{1}H-NMR (300 MHz, $CDCl_3$)

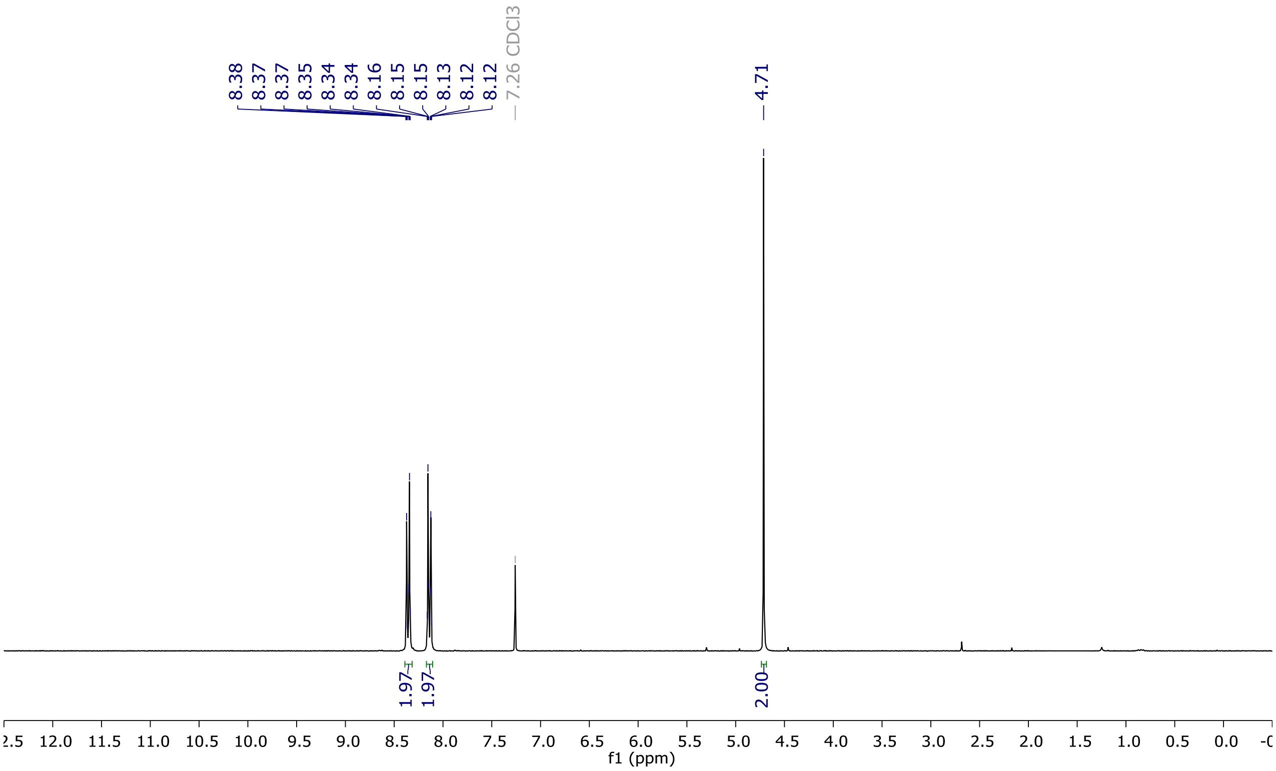

^{13}C-NMR (75 MHz, $CDCl_3$)

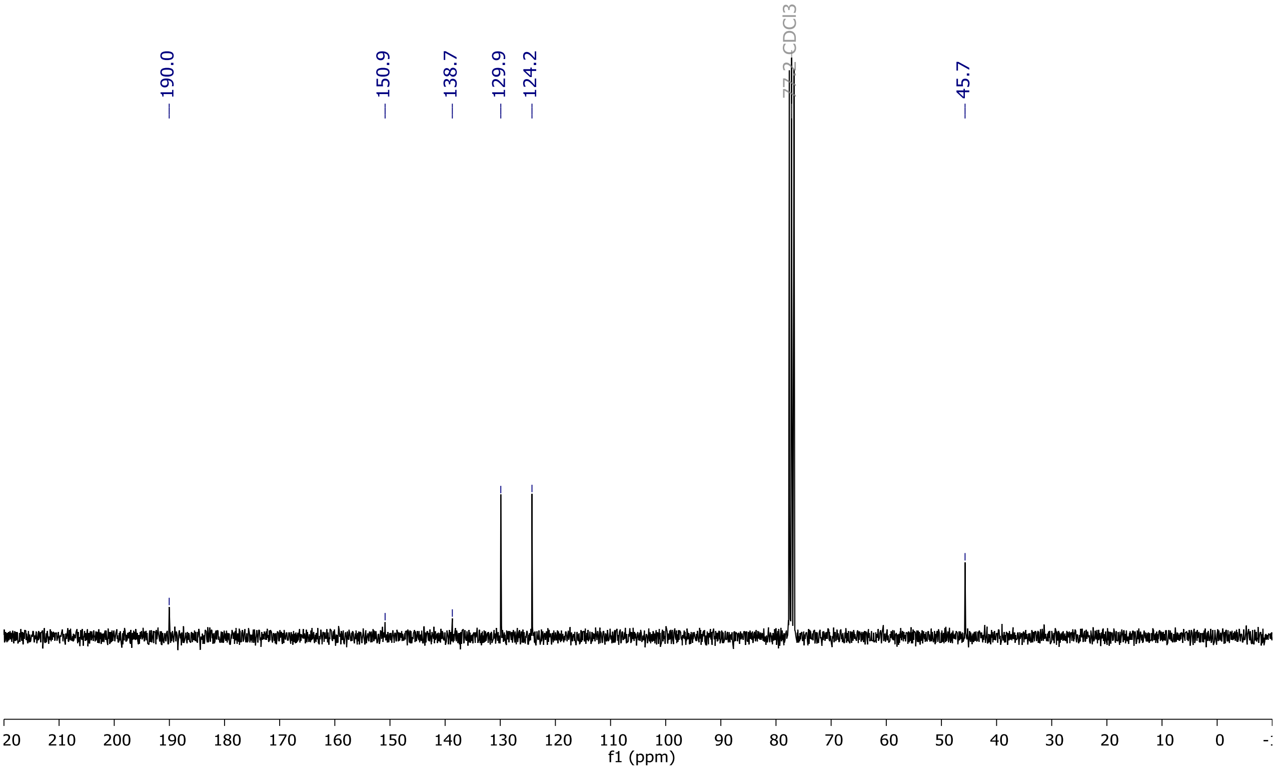

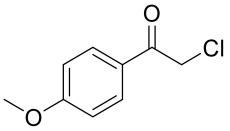

2n

^{1}H-NMR (300 MHz, $CDCl_3$)

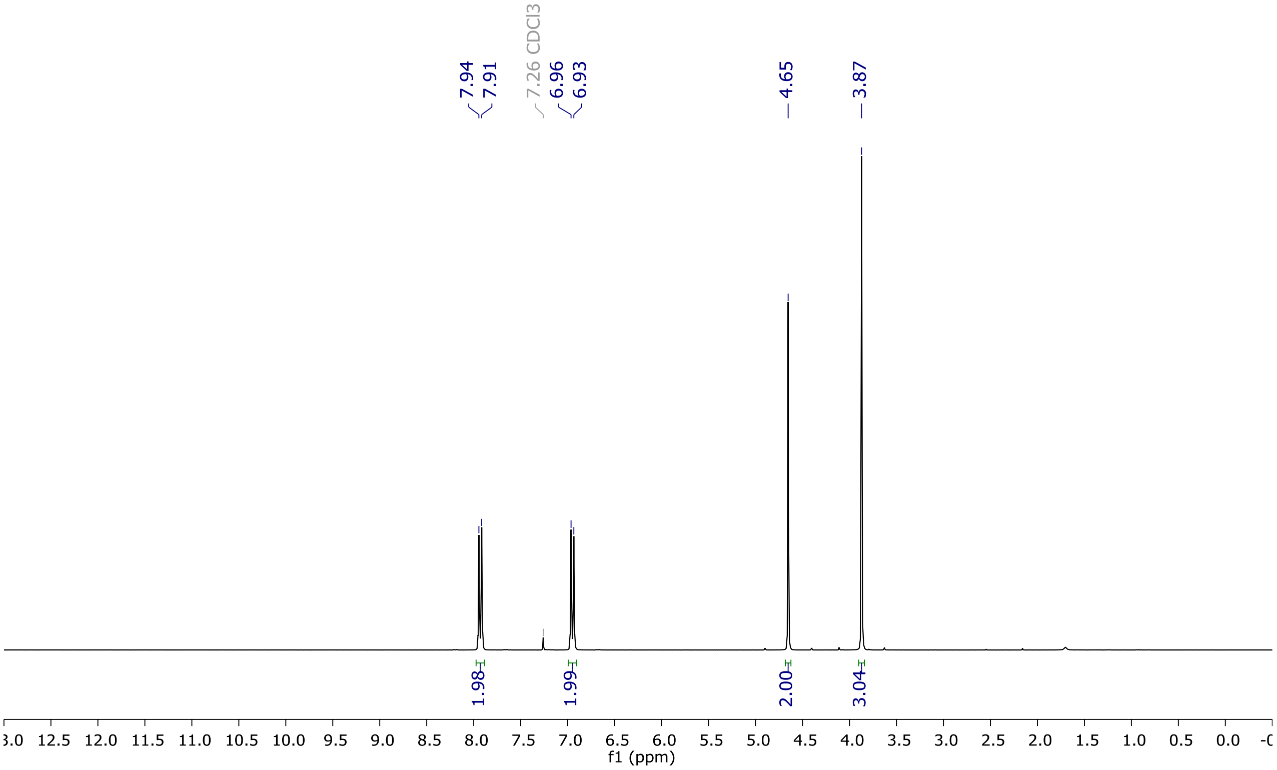

^{13}C-NMR (75 MHz, $CDCl_3$)

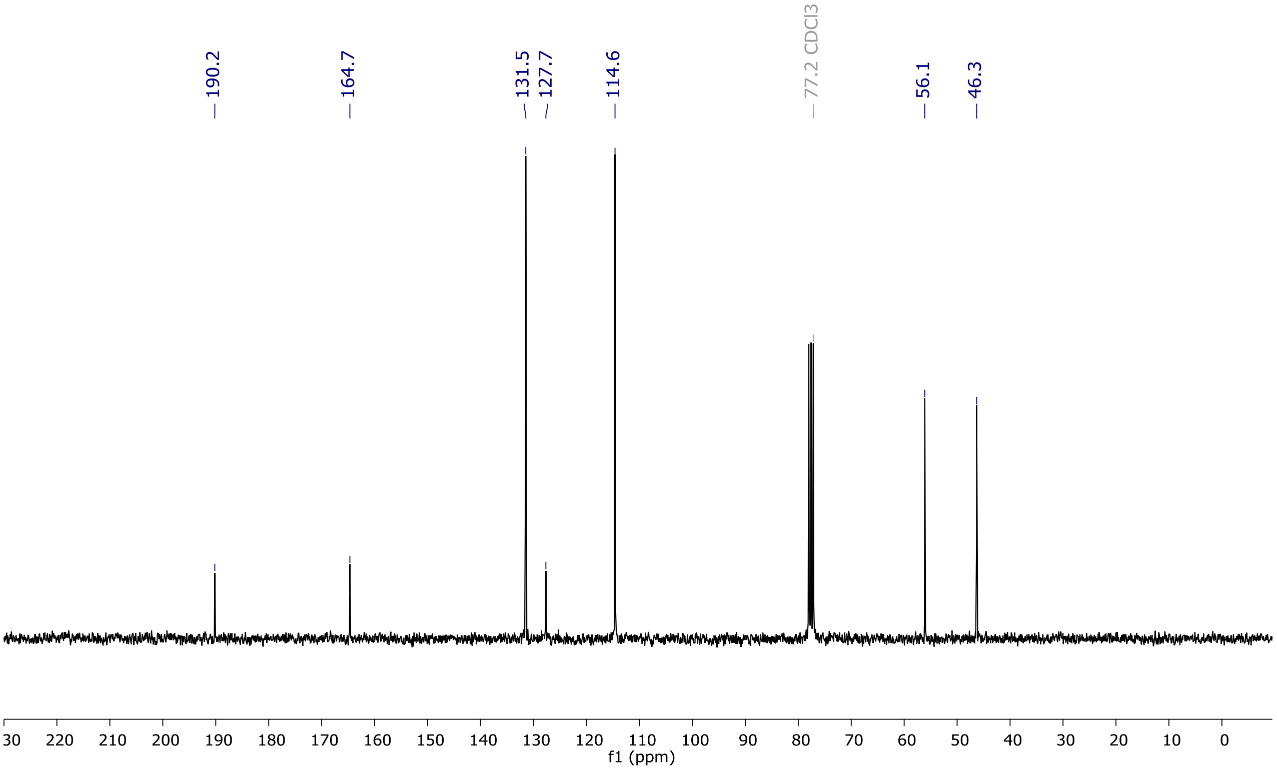

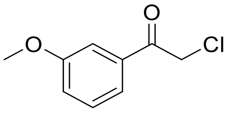

2o

^{1}H-NMR (300 MHz, $CDCl_3$)

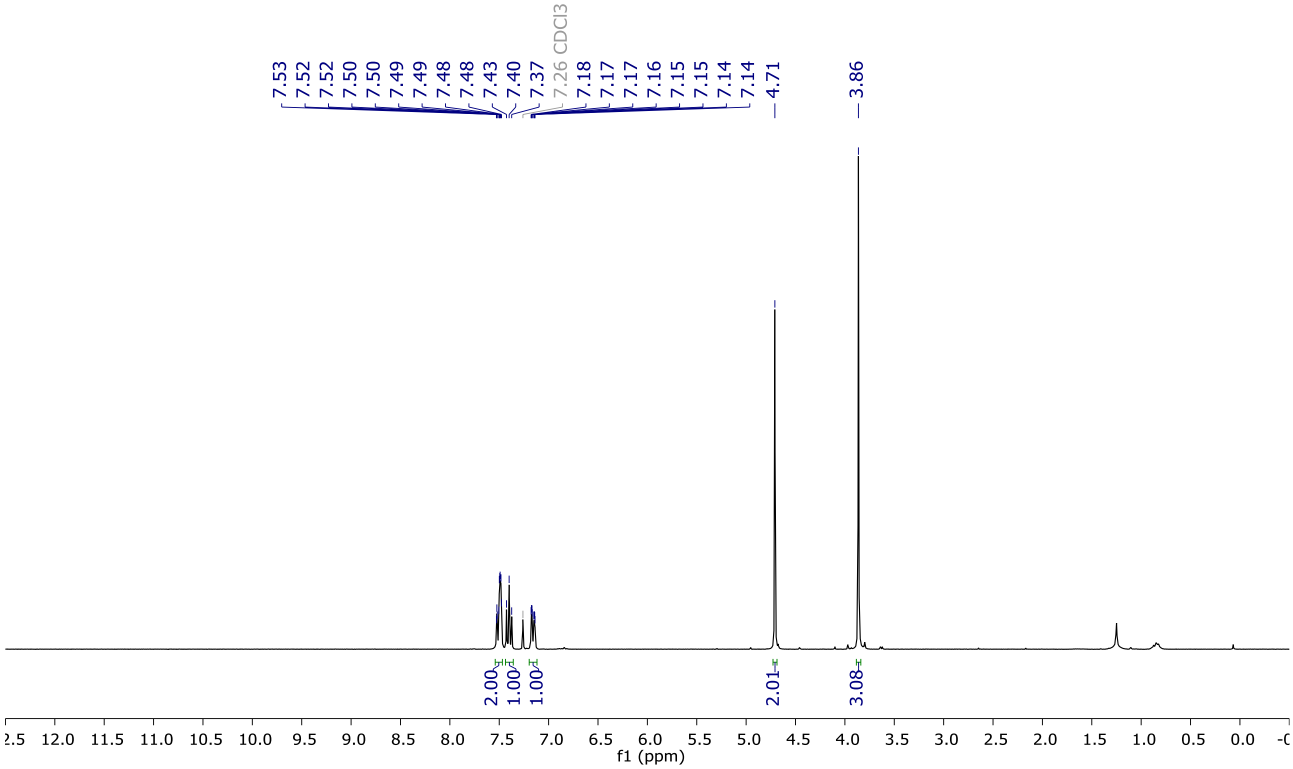

^{13}C-NMR (75 MHz, $CDCl_3$)

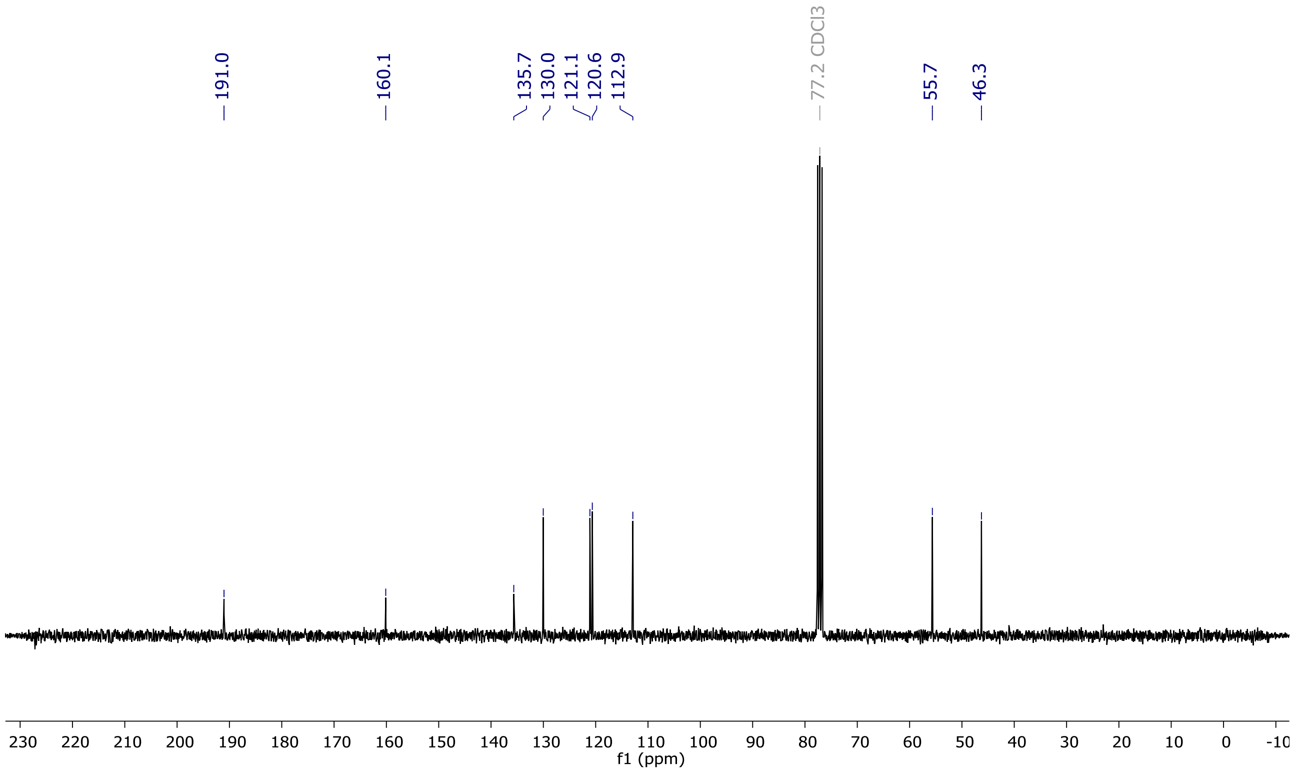

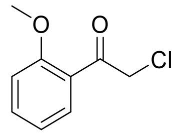

2p

^{1}H-NMR (300 MHz, $CDCl_3$)

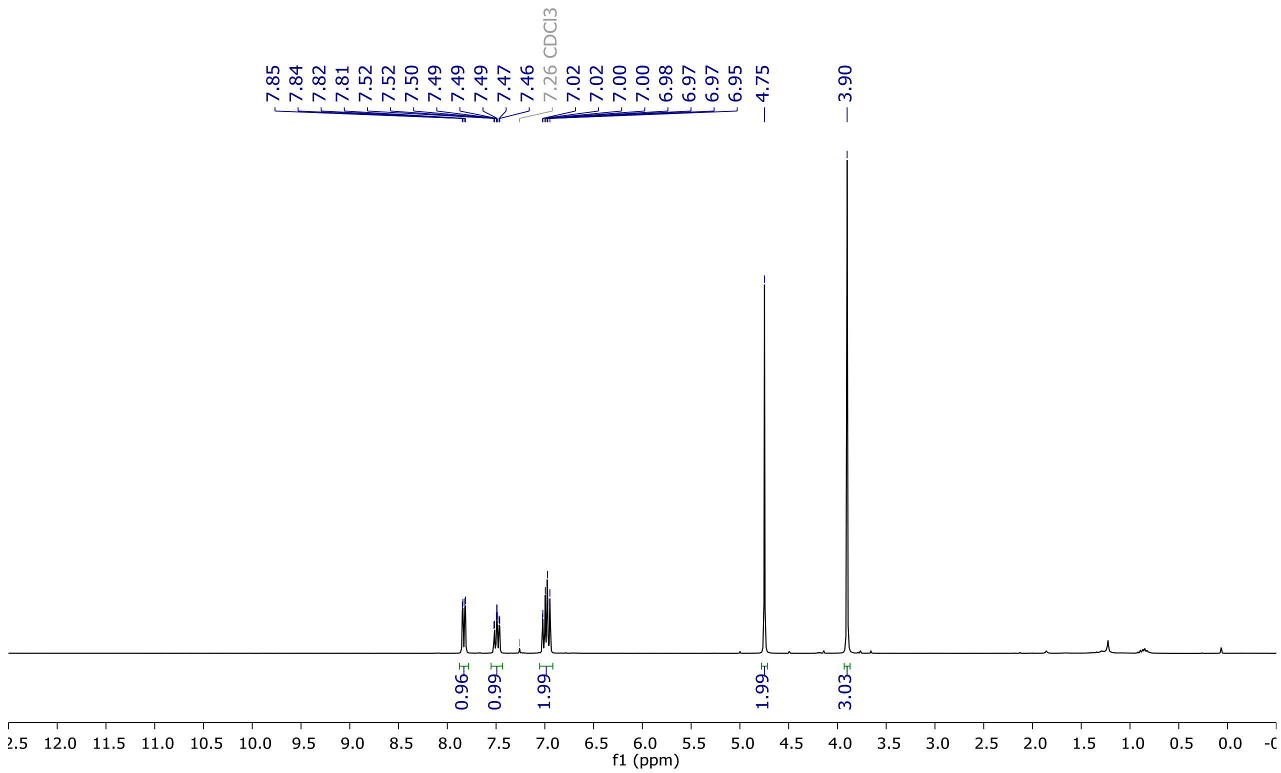

^{13}C-NMR (75 MHz, $CDCl_3$)

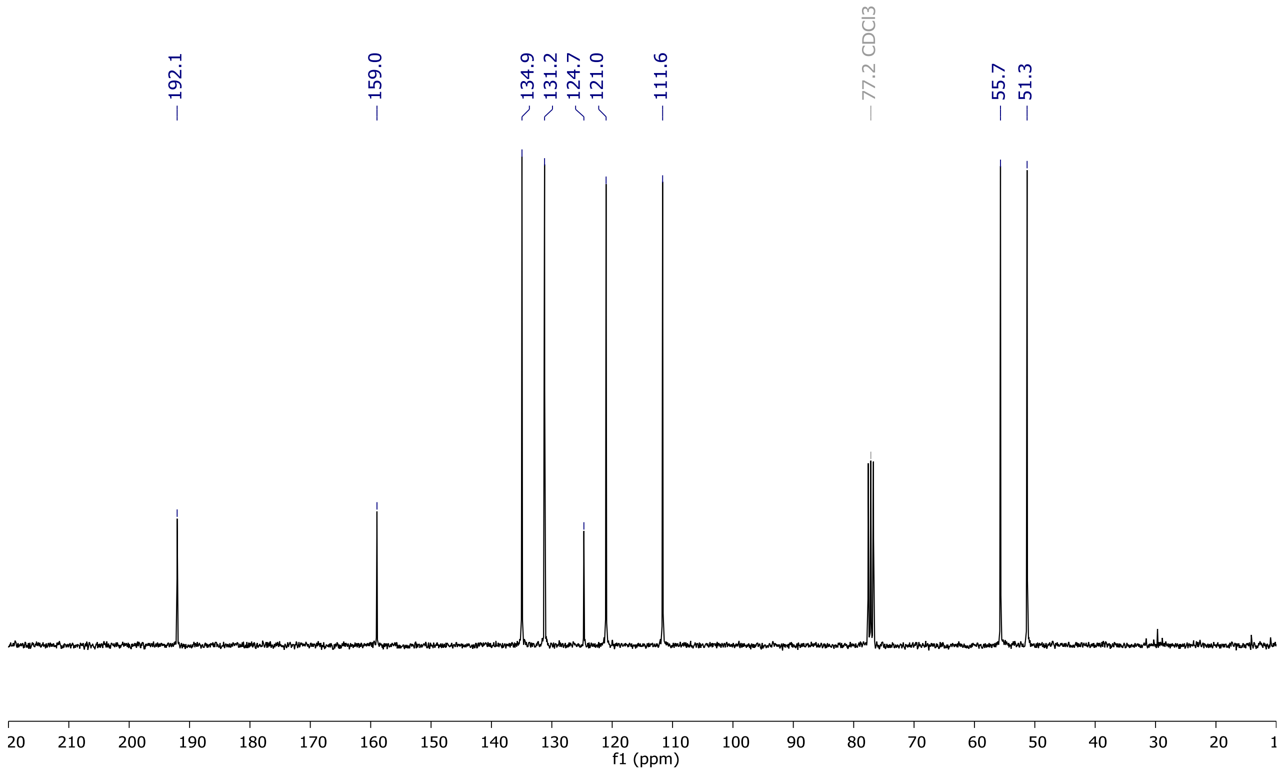

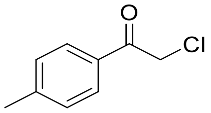

2q

^{1}H-NMR (300 MHz, $CDCl_3$)

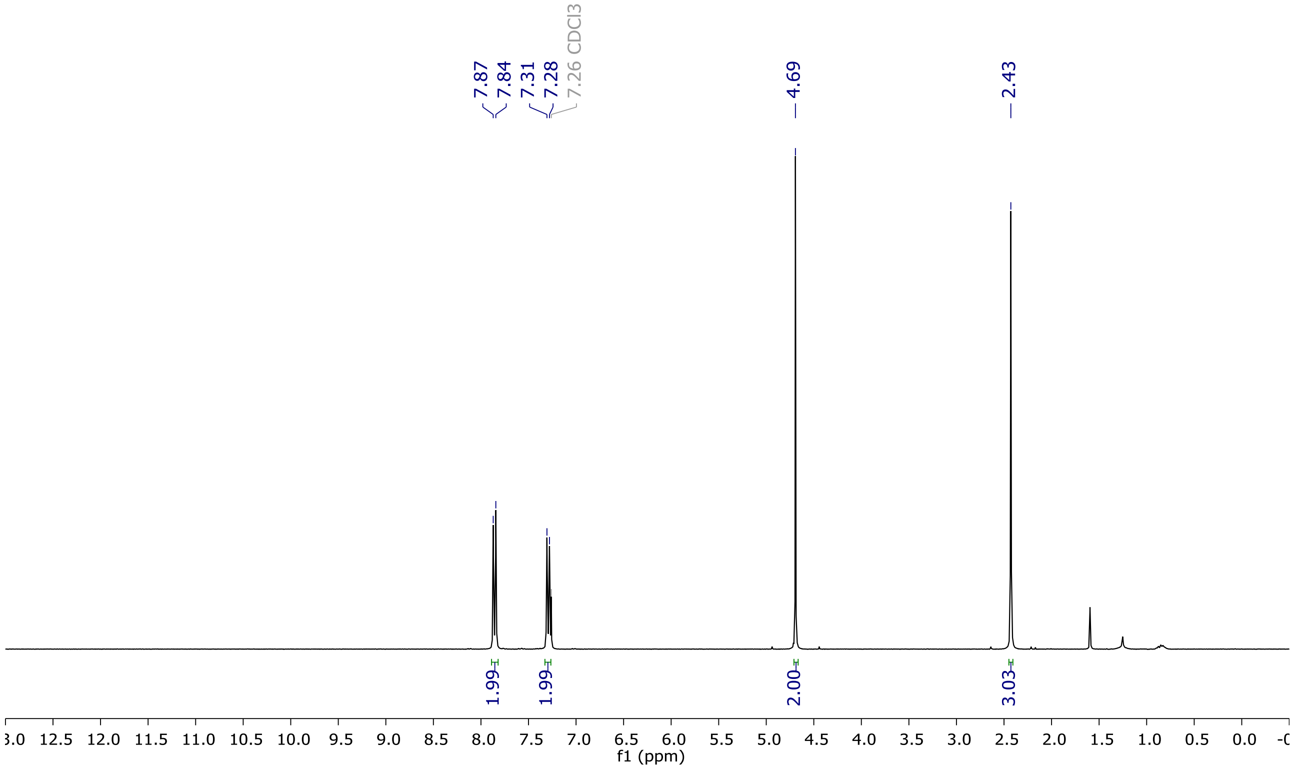

^{13}C-NMR (75 MHz, $CDCl_3$)

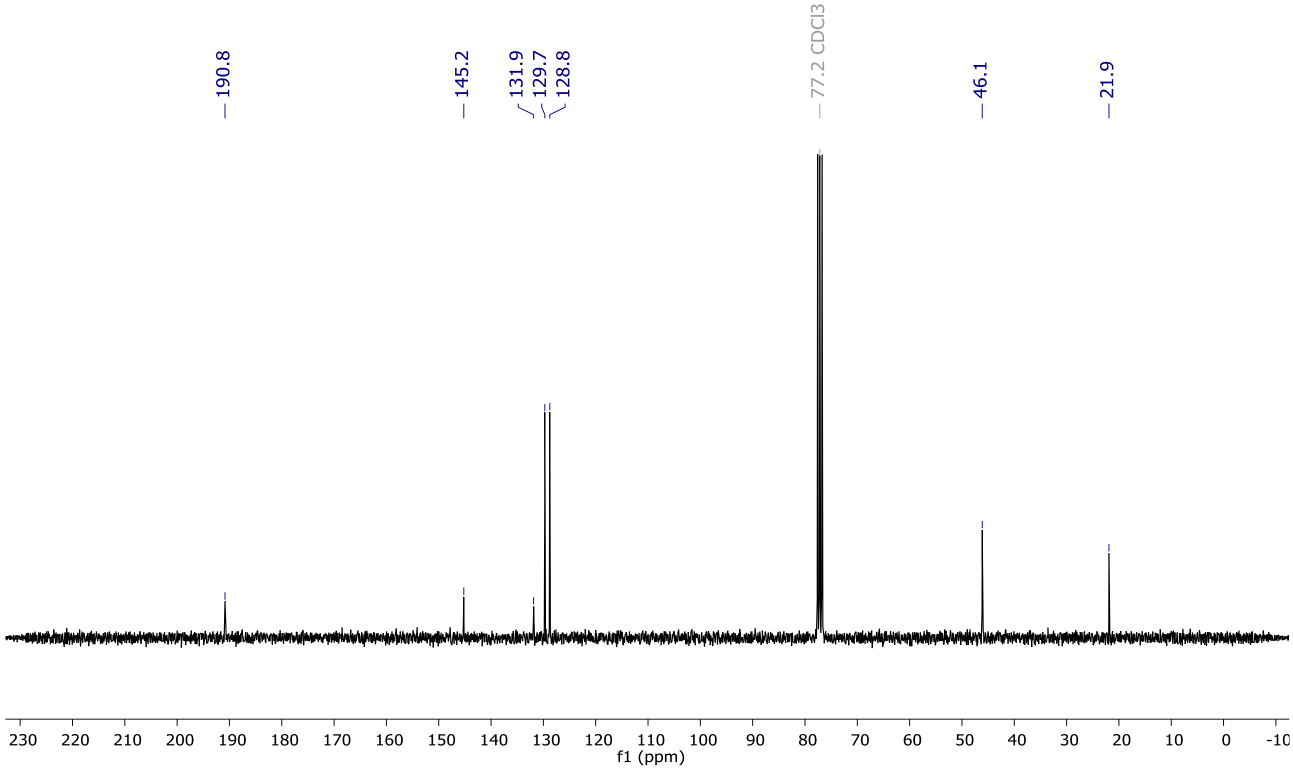

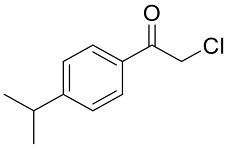

2r

[1]**H-NMR** (300 MHz, $CDCl_3$)

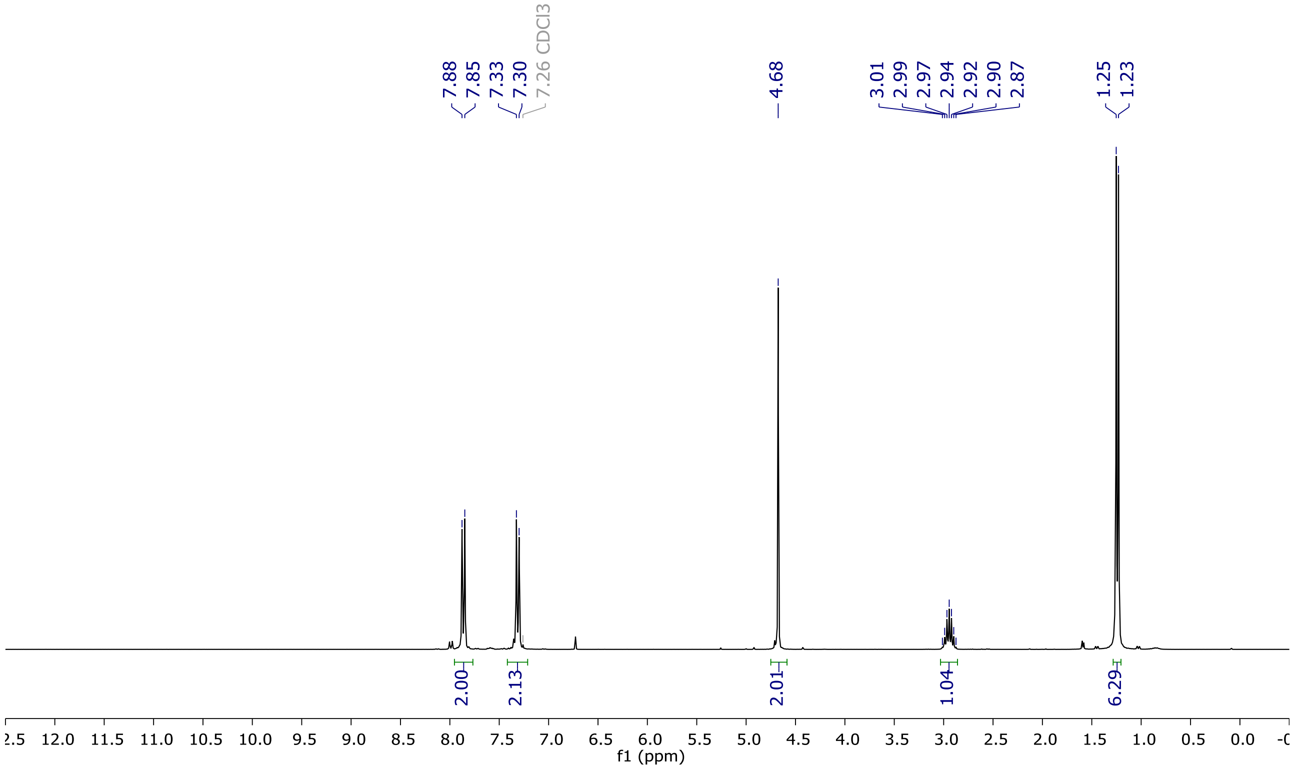

[13]**C-NMR** (75 MHz, $CDCl_3$)

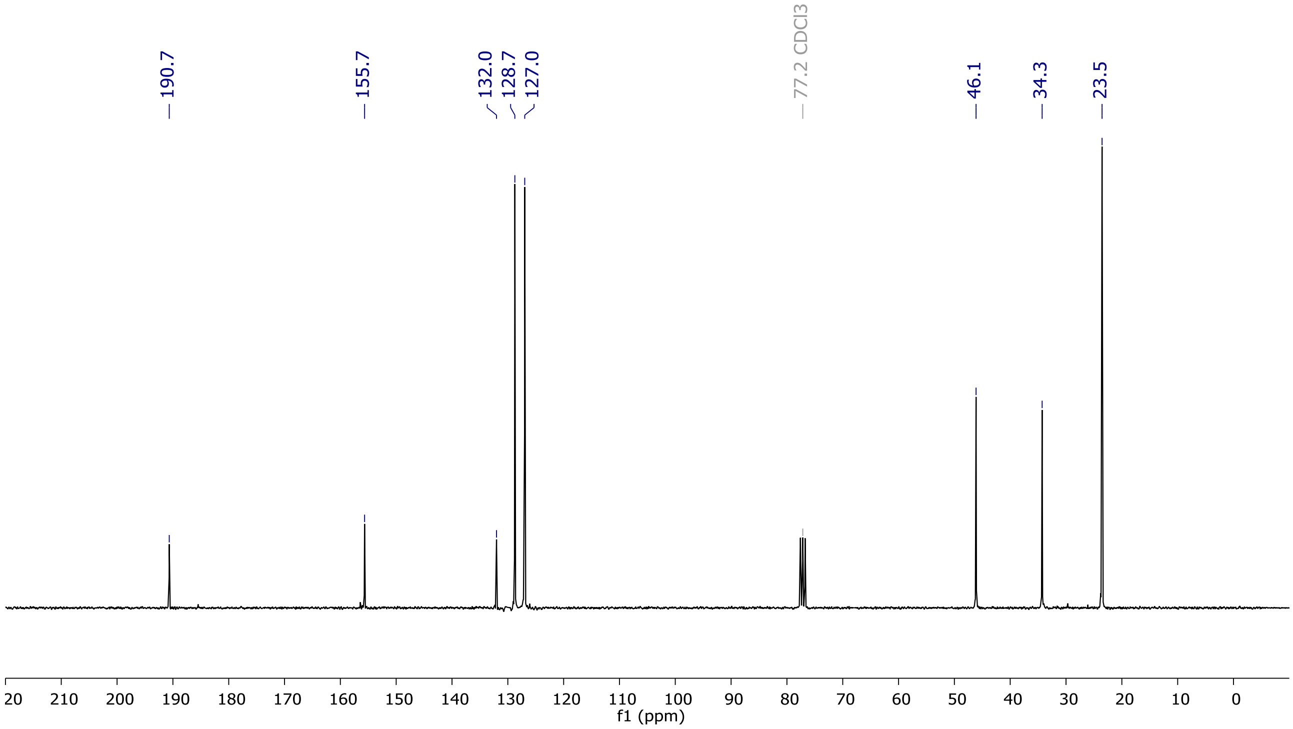

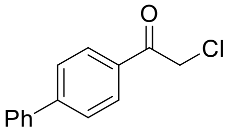

2s

^{1}H-NMR (300 MHz, $CDCl_3$)

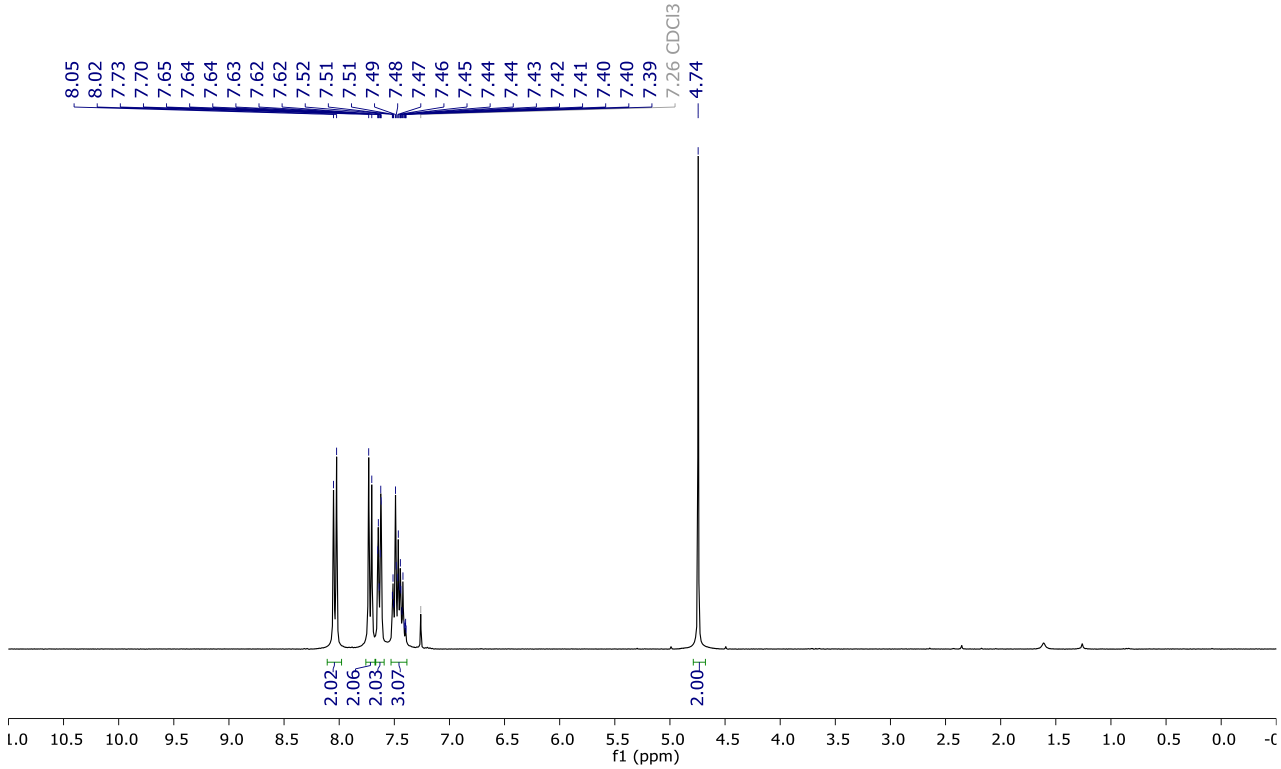

^{13}C-NMR (75 MHz, $CDCl_3$)

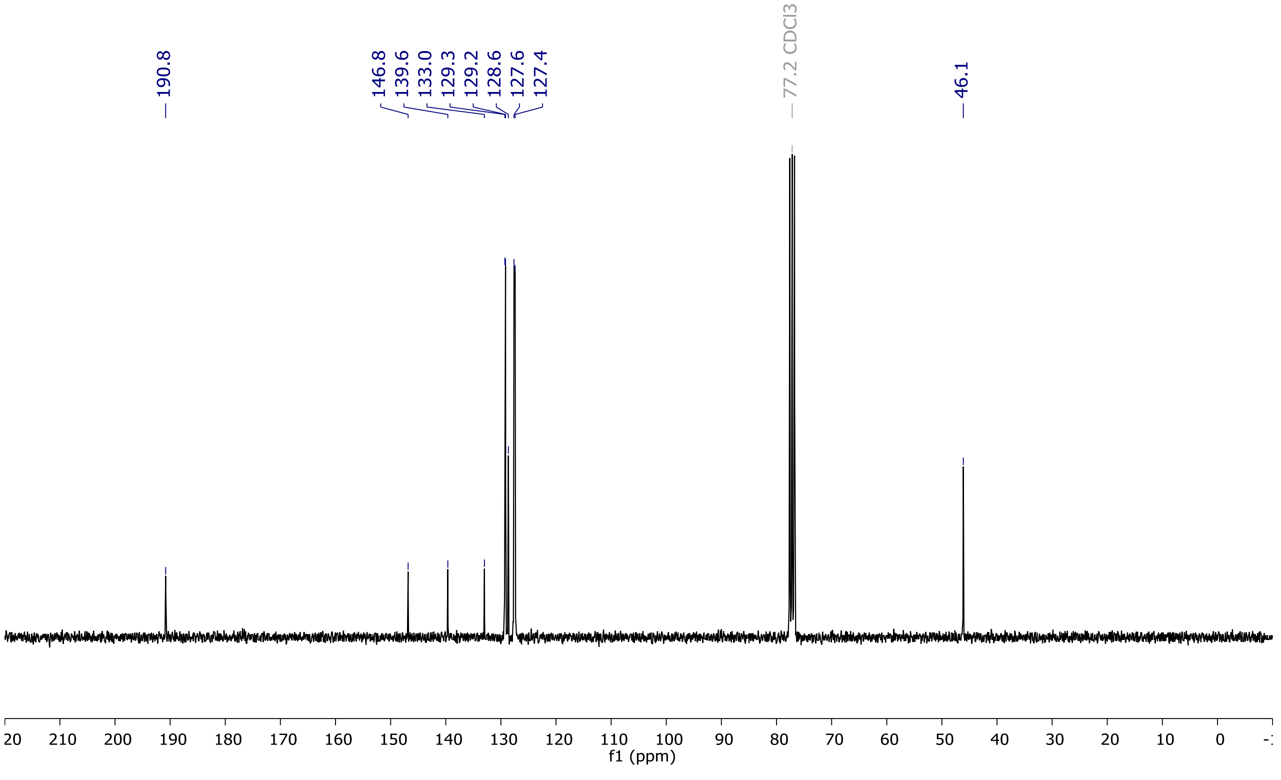

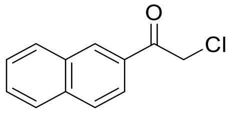

2t

^{1}H-NMR (300 MHz, $CDCl_3$)

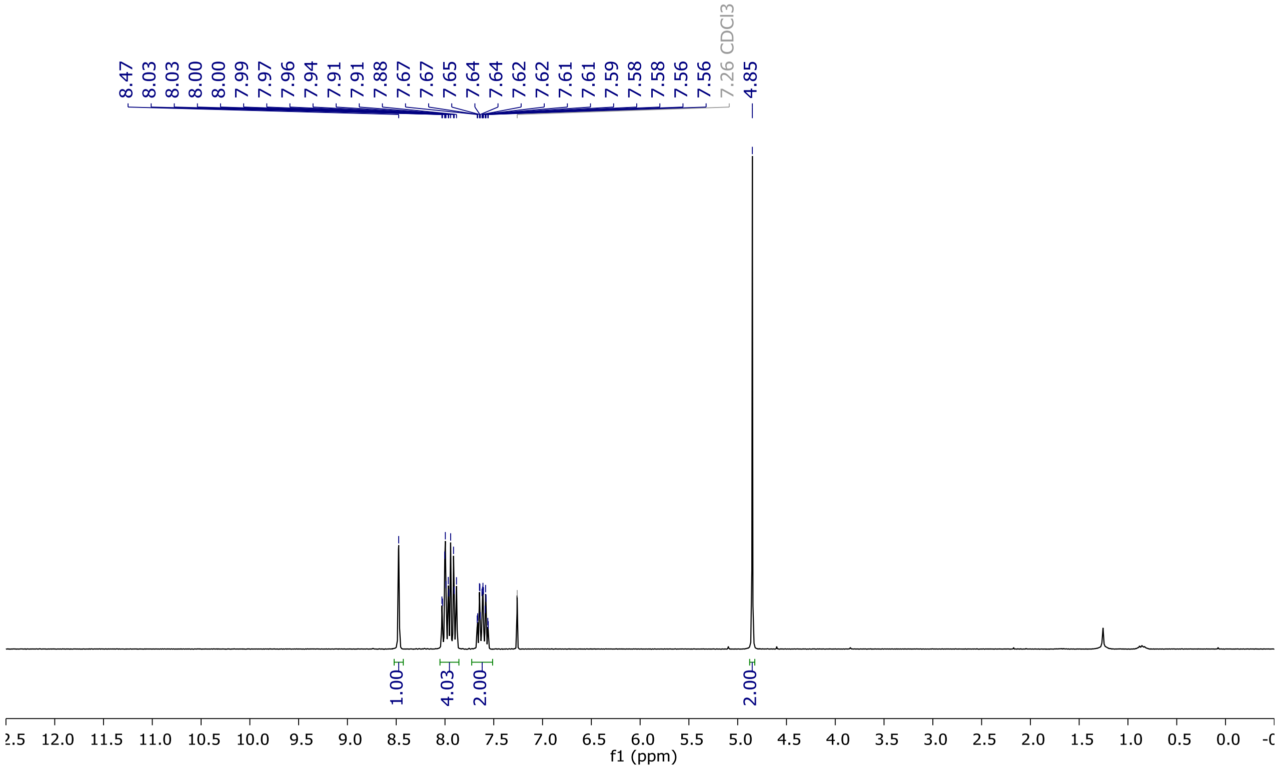

^{13}C-NMR (75 MHz, $CDCl_3$)

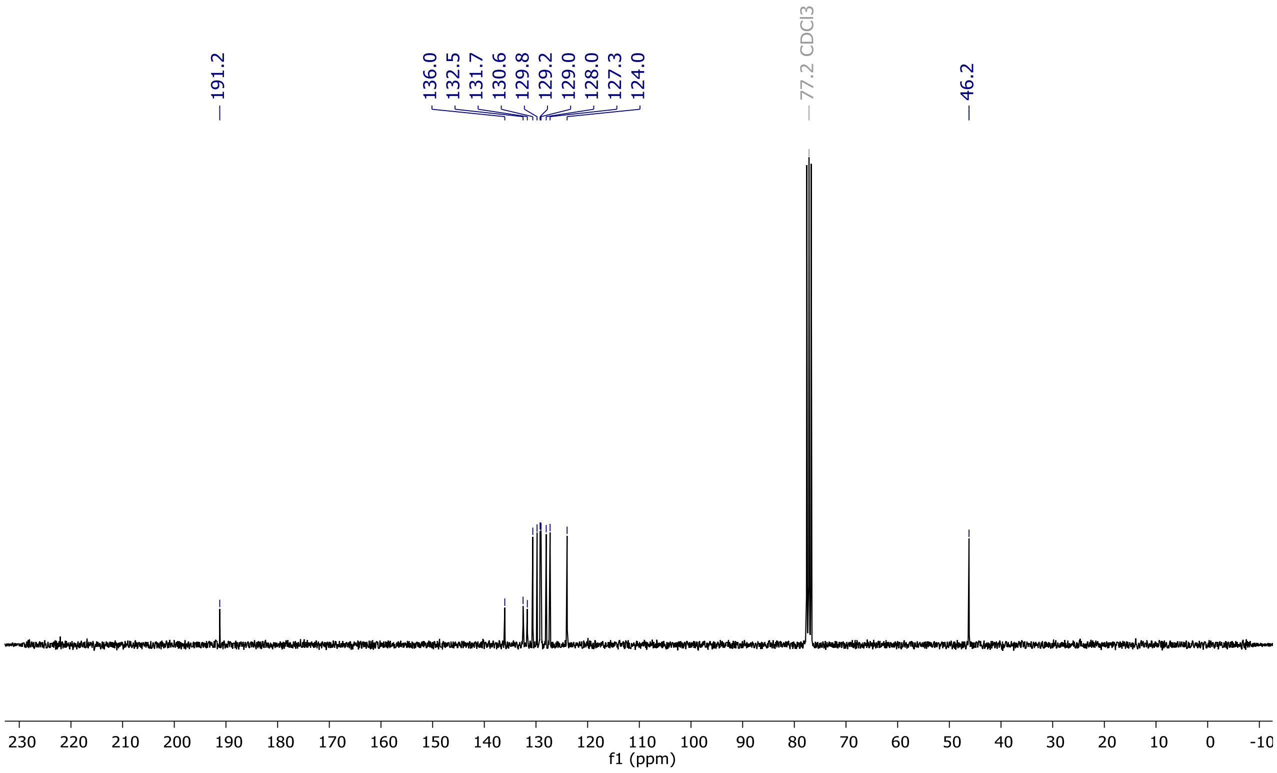

Supplement: Supplementary file 1 — Supplementary Material [file CSSC-18-e202500683-s001.pdf]
